# Deception detection with machine learning: A systematic review and statistical analysis

**DOI:** 10.1371/journal.pone.0281323

**Published:** 2023-02-09

**Authors:** Alex Sebastião Constâncio, Denise Fukumi Tsunoda, Helena de Fátima Nunes Silva, Jocelaine Martins da Silveira, Deborah Ribeiro Carvalho

**Affiliations:** 1 PPGGI, Universidade Federal do Paraná, Curitiba, State of Paraná, Brazil; 2 PPGPSI, Universidade Federal do Paraná, Curitiba, State of Paraná, Brazil; 3 PPGTS, Pontifícia Universidade Católica do Paraná, Curitiba, State of Paraná, Brazil; Sejong University, REPUBLIC OF KOREA

## Abstract

Several studies applying Machine Learning to deception detection have been published in the last decade. A rich and complex set of settings, approaches, theories, and results is now available. Therefore, one may find it difficult to identify trends, successful paths, gaps, and opportunities for contribution. The present literature review aims to provide the state of research regarding deception detection with Machine Learning. We followed the PRISMA protocol and retrieved 648 articles from ACM Digital Library, IEEE Xplore, Scopus, and Web of Science. 540 of them were screened (108 were duplicates). A final corpus of 81 documents has been summarized as mind maps. Metadata was extracted and has been encoded as Python dictionaries to support a statistical analysis scripted in Python programming language, and available as a collection of Jupyter Lab Notebooks in a GitHub repository. All are available as Jupyter Lab Notebooks. Neural Networks, Support Vector Machines, Random Forest, Decision Tree and K-nearest Neighbor are the five most explored techniques. The studies report a detection performance ranging from 51% to 100%, with 19 works reaching accuracy rate above 0.9. Monomodal, Bimodal, and Multimodal approaches were exploited and achieved various accuracy levels for detection. Bimodal and Multimodal approaches have become a trend over Monomodal ones, although there are high-performance examples of the latter. Studies that exploit language and linguistic features, 75% are dedicated to English. The findings include observations of the following: language and culture, emotional features, psychological traits, cognitive load, facial cues, complexity, performance, and Machine Learning topics. We also present a dataset benchmark. Main conclusions are that labeled datasets from real-life data are scarce. Also, there is still room for new approaches for deception detection with Machine Learning, especially if focused on languages and cultures other than English-based. Further research would greatly contribute by providing new labeled and multimodal datasets for deception detection, both for English and other languages.

## Introduction

We aim to find out which Machine Learning techniques perform best for automatic deception detection, what kind of data they process, what is the source of that data, and what theoretical framework they have used. We also seek to understand their limitations and merits, and what remains to be explored.

Therefore, this paper is not about Artificial Intelligence, Machine Learning, or deception detection. Instead, it is a literature review on deception detection with Machine Learning. Our intention is not to go deep into either deception detection or Machine Learning. Instead, our focus is on selecting and scrutinizing research papers on the application of Machine Learning for deception detection.

For this study, we define both “deceiving” and”lying” as the intentional act of making the interlocutor believe in something the deceiver considers false [1]; it is a conscious and deliberated act, perpetrated by the deceiver [2]. However, a false information believed to be true by the emitter is not considered deceptive.

Lying is a frequent and pervasive social phenomenon [3]. While some forms may be accepted as a “social lubricant” [4], others are socially harmful. Telling (and being told) lies is frequent but perceiving them is a major challenge for most people. The average person has a lie detection rate around 54% [5, 6], rarely reaching 60%, and sometimes falling below 50% [7].

Nevertheless, some individuals show a remarkable ability for spotting deceptions, with a detection accuracy above 90%. Referred to as “Wizards of deception detection” [5], these individuals demonstrate that lies can be detected. Such “wizards”, however, are not numerous.

Machine Learning has been successfully applied to a large number of fields and functions, such as document classification, computer vision, natural language processing, protein structure prediction, fraud and malware detection [8], medical diagnosis and data privacy [9], network and data transmission security [10], intrusion detection [11], generative molecular design [12], and recommendation systems, among others [13]. Also, it offers a vast set of techniques, providing several opportunities to approach various problems. Seeing Machine Learning applied to deception detection is not surprising.

We noticed many studies on deception detection aided by Machine Learning have been published in the last decade. Those report different approaches and results, a rich and bulky corpus of knowledge is available. The results, however, suffer from large variance, with a diversity of settings, techniques, complexities, and strategies based on several theoretical frameworks. Identifying trends, gaps, and research opportunities may be challenging.

Due to the diversity of studies and the difficulty of establishing a general state of technology on deception detection with Machine Learning, we felt stimulated to formulate the following research questions: a) What are the best-performing Machine Learning techniques applied to automatic deception detection? b) What are the datasets and features they consume? c) What level of performance have they reached recently?

This literature review aims to answer those questions and give a comprehensive overview of the application of Machine Learning for deception detection. We intend to report what researchers have exploited as techniques and approaches, their difficulties, what kind of data they have consumed, and what performance levels they have achieved.

To the best of our knowledge, this is the first literature review that scrutinizes the application of Machine Learning for deception detection. Trends, gaps, difficulties, results, and opportunities are highlighted to stimulate further studies and new developments in the area.

Our main contributions are as follows:

1. To identify the most frequent and performant Machine Learning techniques;
2. To enumerate the most explored feature modalities;
3. To compare the studies’ approaches with theoretical frameworks of deception detection;
4. To summarize articles in the form of mind maps;
5. To make the whole history and statistical analysis available as Jupyter Lab Notebooks.

The rest of this report is organized as follows: a) the “Theoretical background” section presents some basic knowledge on deception detection and Machine Learning; b) the “Materials and Methods” section presents the research process in detail; c) the “Data availability” section describes where all the data is located; d) the “Assessment of quality and the risk of bias” section describes the two tools used to asses quality and risk of bias in this report; the “Results” section presents the selected corpus and the answer to the research questions; e) the “Discussion” section comments various thematic dimensions emerged from the selected corpus; f) the “Current state and further research” section presents a general conclusion regarding the state of the field, and presents some opportunities for future work; g) the “Limitations and further work” section presents the limitations of this research and proposals for future extensions; h) the “Conclusions” section presents our final words and reflections in light of the findings.

### Theoretical background

The following sections present some background on the main topics explored in this review.

#### Deception detection

Deception detection is the act of deciding whether a certain communication carries the truth or not. It is an active and evidence-driven inference process [14]. High-stakes deceptions are believed to induce behavioral and physiological changes in the deceiver, yielding more evident indicators of lie-telling [1]. The task even more challenging because no clue alone is an indisputable predictor of deception [2, 4, 15].

The behavioral and physiological changes experienced by the deceiver work as deception cues [1, 2, 16]. An observer that notices these cues may be enabled to detect an attempt of deception. Such behavioral changes are what human lie detectors observe to make their judgment.

Deception cues can be verbal (effects on the deceiver’s speech) and non-verbal (impact on how the deceiver speaks and acts). The different sources of cues are usually called modalities or channels. For instance, the cues identified from a deceiver’s voice are said to be from the vocal channel or to belong to the vocal modality.

Examples of non-verbal cues include gestures such as self-adaptors (touching one’s own body, face, or hair) [4], manipulators (pinching, picking, scratching) [2], emblems (gestures that replace words) [2], or illustrators (gestures that accompany speech) [2, 4].

The need for better ways to detect deceptions stimulated the creation of aiding technology to increase the detection accuracy. The most famous example is the polygraph, introduced in the Berkeley Police Department by John Larson [17], in 1921. The current polygraph models can monitor several physiological responses from a subject and require a preliminary calibration step to establish a baseline for the operator.

It is essential to understand that the polygraph does not detect lies. Instead, it shows physiological alterations related to emotions [2]. It’s the operator that interprets and decides whether a given message is sincere or not. There are accounts of false positives who have been exonerated of criminal charges after further investigations proved the polygraph test mistakenly detected a deception [2]. Due to the polygraph’s limitations, other technological opportunities began to be considered. Positive results achieved by Machine Learning in several fields over the last 20 years worked to stimulate new research to respond to the challenges of deception detection.

#### Machine Learning

Machine Learning is a branch of Artificial Intelligence that allows computers to learn from data and acquire skills to work on a task without being programmed explicitly for it [18]. It is a multi-disciplinary field that includes contributions from Psychology, Neuroscience, Control Theory, and Philosophy, to name a few [19].

By consuming spreadsheet-like structures (datasets), Machine Learning algorithms produce a so-called model, a general representation of the patterns in data. Each row of the dataset is an example or individual and each column is a feature [13, 20].

It is usual to separate a part of the dataset for training and another for testing. Training is the phase of producing the model from the data, whereas testing consists of measuring the model’s performance and generality.

Machine Learning can be applied to many different tasks, such as Classification, Regression, Clustering, Association Rules, and Anomaly Detection. For each task, different techniques can achieve different performance levels.

Classification tasks rely on algorithms that assign a given class (or label) to a specific data example. Those classes are a limited number of categorical values [19]. So, they are not continuous values (while features can be).

Models for Classification problems (classifiers) frequently use a so-called supervised learning process. Each training data example already has a label (or class) assigned [21]. The Machine Learning algorithm will produce a model that relates specific examples to certain classes to predict the class for a new, unseen, data example. That’s why it is often also called a predictive model.

Predictive models are useful in many problems, such as price prediction, risk assessment, medical diagnosis, document classification [22], spam filtering, image classification, fraud detection, churn analysis, risk analysis [21], among others. For detection purposes, Classification models can be used for detecting diseases like Alzheimer’s disease [23] or skin pathologies [24], detecting physiological alterations [25, 26] and even traffic accidents [27].

Alternatively, unsupervised or self-supervised learning happens when the model training does not require labeled data [20].

There are several Machine Learning algorithms based on different theoretical frameworks and strategies [19], such as Decision Trees [28], Naïve Bayes [29], Support Vector Machines [30], K-Means [31], Random Forests [32] and Neural Networks [33].

No matter the problem, the area, or the algorithm, however, it is constant that the quality of data for training the models plays a crucial role in the success of any Machine Learning project.

#### Deep Learning

Deep Learning is a kind of Machine Learning that represents knowledge as a hierarchical structure, building complex and specific representations over simpler and broader ones [20].

Conventional Machine Learning methods are severely impacted by the features they consume. Wrong features may lead to incorrect or undesired results, which promotes an entire area of study known as feature engineering. However, Deep Learning methods can detect which features are relevant in raw data and extract them instead of others [20].

Deep Learning models are usually very complex, composed of multiple layers of representations, easily ranging from dozens to hundreds. Such stacked layers store the hierarchical representations that allow the model to encode complex data relationships usually found in challenging problems [34].

While Deep Learning has achieved remarkable results in many areas, it relies on large amounts of data for training. Furthermore, the number of parameters that make up a Deep Learning model can reach millions, which makes the training process extraordinarily demanding and may require the power of many Graphic Processing Units (GPUs) for several days.

## Materials and methods

We decided to store the history of our process in Jupyter Lab Notebooks. Those are digital documents that store and run Python code. Such notebooks: a) record our Research protocol, found in S3 File (Definition Jupyter Notebook); b) present the steps for selecting the corpus, found in S4 File (Corpus collection Jupyter Notebook); c) present the steps for analyzing the corpus, found in S5 File (Corpus analysis Jupyter Notebook); d) present all the steps for processing the extracted metadata, found in S6 File (Statistical analysis Jupyter Notebook) and; e) show all the mind maps built for each document in the selected corpus, found in S7 File (Mind maps Jupyter Notebook). Our research is reproducible, and its entire history is preserved.

This study is both a qualitative and quantitative review. An analysis consisting of statistical evaluations of the selected articles [35] comprises the quantitative portion and was performed to describe studies from a numerical and objective perspective.

All the metadata was extracted directly from the selected corpus and no value was, by any means, inferred or interpreted. Sometimes, the total number of features was summed when the text didn’t present it, but all the primitive values were there. Such metadata describes the source of training data, training strategy, Machine Learning methods, dataset sizes, predictors exploited, cues complexity, modality cardinality, performance levels, and performance metrics.

Such an analysis is not a meta-analysis since we designed the research to present a broad picture, not limited to evaluating only the final performance reported. The wide spectrum of factors stored into the metadata from each article naturally led to a high level of heterogeneity, which prevented any attempt to combine them.

However, the statistics rendered a rich, multidimensional profile of the topic and author’s approaches, highlighting their choices, limitations, expectations, and results. Those can be found in S6 File (Statistical analysis Jupyter Notebook).

As for the qualitative portion, our findings are discussed in light of the knowledge about deception detection techniques from a psychological perspective. Such discussions include themes that emerged from the selected corpus. They are more a finding than a choice and are all presented in the “Discussions” section.

The established design, supporting tools, research restrictions, and other details are presented in the following sections.

### Research goals and inclusion criteria

The main goal of this systematic review is to retrieve and study the most comprehensive collection of scientific production about Machine Learning applied to deception detection in our power. That allowed us to understand the current trends, difficulties, approaches, results, and general state of the field.

Our protocol and selection criteria best balances both our goals and limitations. We believe we could capture the studies that best met our research goals, regardless of the technique, strategy, or approach decisions.

Regarding PICO (Population-Intervention-Comparison-Outcome) components, this research design is as follows: a) Population: studies on deception detection; b) Intervention: Machine Learning techniques; c) Comparator: none; Outcome: performance level.

According to the research interests, we enforced the following selection restrictions: a) only studies that address deception detection; b) only studies that exploit Machine Learning; c) only studies that clearly state what data features were consumed; d) only studies that report a performance level; e) only methods and techniques that consume data from non-invasive sources.

By non-invasive, we mean methods that either do not touch the subjects or observe them by a device less mobile than a regular computer (e.g., a Magnetic Resonance Image machine, MRI). However, studies combining skin-level invasive, and non-invasive approaches were selected.

The period ranged from 2011 to 2021, inclusive, as we consider such years sufficiently recent for our purposes.

### Supporting tools

We exploited the following free resources to improve productivity, precision, and safety:

1. The Python programming language, chosen due to its familiarity to the authors and other research groups;
2. Python packages Pandas and MatPlotLib, for statistical analysis since they are richly featured and usually applied in data analysis;
3. Jupyter Lab, as a platform to run the statistical analysis scripts and generate charts, tables, and a process history;
4. FreeMind, to build summary mind maps from the deep screened papers.

In addition, BiblioAlly, a computer program written in Python, was built by one of the authors, because bibliographic managers, such as Mendeley, do not always interpret bibliographic citation files correctly, thus requiring some extra and time-consuming correction work. Moreover, they do not offer features to store metadata nor manage, track, and support the research workflow. In our case, such citation files were BibTeX files.

BiblioAlly can handle the differences existing in BibTeX files, manage and track the steps of the research protocol, and store the extracted metadata. It greatly optimized the entire process. BiblioAlly. is free and available at GitHub (http://github.com/gambit4348/biblioally) and at the Python Package Index (PyPI).

### Research protocol

Our research protocol is as follows:

1. Run queries on scientific document databases;
2. Export results as BibTeX files;
3. Import all BibTeX files into BiblioAlly;
4. Manually detect duplications not detected by BiblioAlly during import;
5. Pre-select articles by shallow screening:
  - Read title, keywords, and abstract for each paper;
  - Reject studies that violate research restrictions;
6. Retrieve the full text of pre-selected documents;
7. Select articles by deep screening:
  - Read full text;
  - Reject studies that violate research restrictions;
8. Extract relevant data from accepted documents:
  - Build FreeMind mental maps as summaries for the articles;
  - Store the metadata in BiblioAlly;
9. Perform a statistical analysis and generate charts and tables.

The protocol worked as a roadmap so the process can be rigorously and safely replicated.

### Search strategy

Due to previous experience with literature reviews, we expanded our search space by running queries on four different scientific search engines: Web of Science, Scopus, ACM Digital Library, and IEEE Xplore.

We ran two rounds of search. The first one was on March 2^nd^, 2021, and returned studies published from 2010 to 2020. The second run was on May 5^th^, 2022, and returned papers published in 2021. The year 2010 returned no papers that met the research protocol, therefore the period of interest is 2011–2021.

All queries were run using the syntax of each scientific search engine. As an additional filter, the period of interest was limited to 2010–2020 and 2021, depending on the run, as shown in Table 1.

**Table 1:** Search queries issued to different academic search engines.

**Search engine**	**Query strategy**
**Web of Science**	(("deception detection" OR "lie detection") AND ("machine learning" OR "artificial intelligence")) Refined by: Publication Years: (2021 OR 2020 OR 2019 OR 2018 OR 2017 OR 2016 OR 2015 OR 2014 OR 2013 OR 2012 OR 2011)
**Scopus**	TITLE-ABS-KEY (("deception detection" OR "lie detection") AND ("machine learning" OR "artificial intelligence")) AND (LIMIT-TO (PUBYEAR, 2021) OR LIMIT-TO (PUBYEAR, 2020) OR LIMIT-TO (PUBYEAR, 2019) OR LIMIT-TO (PUBYEAR, 2018) OR LIMIT-TO (PUBYEAR, 2017) OR LIMIT-TO (PUBYEAR, 2016) OR LIMIT-TO (PUBYEAR, 2015) OR LIMIT-TO (PUBYEAR, 2014) OR LIMIT-TO (PUBYEAR, 2013) OR LIMIT-TO (PUBYEAR, 2012) OR LIMIT-TO (PUBYEAR, 2011))
**ACM Digital Library**	[All: "deception detection"] OR [All: "lie detection"] AND [Publication Date: (01/01/2011 TO 12/31/2021)]
**IEEE Xplore**	(("All Metadata": "deception detection") OR "All Metadata": "lie deception") Filters Applied: 2011–2021

Source: The authors (2022)

Each scientific database allows the inclusion of extra metadata during the export to BibTeX format files. We included all these extra metadata.

### Data extraction

To perform the analysis with Pandas and MatPlotLib, the extracted metadata was encoded as Python dictionaries:

1. **document_id**: the document id in the BiblioAlly database;
2. **methods**: list of methods and tools, each item described as:
  - **classifier**: the classification algorithm in terms of:
    **kind**: when applicable, the sub-category of the method;
    **implementation**: software, package or library that provided the algorithm;
    **training**: training method;
    **performance**: classification performance as:
      **1. kind**: the performance measure;
      **2. value**: the performance level;
  - **support**: supporting tool for generic purposes;
3. **dataset**: description of the dataset used in the study:
  - **public**: True indicates a freely accessible dataset, False the opposite;
  - **mock**: True indicates a dataset collected from some fabricated setting, False indicates data collected from real-life circumstances;
  - **name**: name of the dataset, if any;
  - **size**: number of dataset rows;
  - **origin**: source of the data;
  - **target**: labels used for the target attribute;
  - **features**: list of feature kinds that make up the dataset:
    **kind**: the kind of detection cue features;
    **dimensions**: the number of features;
    **components**: list of feature components;
    **language**: list of languages, when applicable;
    **tool**: list of tools, when applicable;
4. **notes**: textual notes about the study;
5. **mindmap**: file name of the mind map document.

The dictionaries are readable by non-pythonists, provided a short explanation is given.

### Data access

For transparency, we made available all data collected and encoded in this research in a GitHub repository (http://github.com/gambit4348/deception-detection-review-2021). The Jupyter Lab Notebooks work as history for whole process. The BibTeX files and the BiblioAlly database can be used under the MIT License and are also available. FreeMind documents are also available.

For convenience, we included those Jupyter Lab Notebooks as additional documents of this review as Notebook 1 (Definition), Notebook 2 (Corpus collection), Notebook 3 (Corpus analysis), Notebook 4 (Statistical analysis), and Notebook 5 (Mindmaps) as PDF (Portable Document Format) files.

Not all the articles selected for the review were under open access, so we decided not to make any available to avoid any copyright violations.

### Assessment of quality and the risk of bias

The AMSTAR-2 (A MeaSurement Tool to Assess systematic Reviews) tool [36] (https://amstar.ca/Amstar_Checklist.php) was filled in to assess the quality parameters of this review. The assessment report is available in S1 File.

Additionally, an Excel spreadsheet was built and filled in to implement the PROBAST (Prediction model Risk Of Bias Assessment Tool) [37] assessment of the risk of bias (http://www.probast.org). The PROBAST model provides 20 questions across 4 domains (participants, predictors, outcome, and analysis) that produce individual risk of bias and applicability for each document of the review corpus. Such a spreadsheet file can also be accessed in the S2 File.

## Results

Our main goal is to comprehensively understand of the state of research regarding deception detection with Machine Learning. To do so, we surveyed, studied, and selected a collection of 81 documents out of 648 retrieved from four scientific databases. We report our findings in both quantitative and qualitative fashions.

From a quantitative perspective, we gathered a rich set of metadata to produce many charts and tables. Those numerically and objectively describe all the papers in the selected corpus (S5 and S6 Files).

From a corpus standpoint, a particular Jupyter Lab Notebook (S5 File) presents several charts and tables.

We offer a specific Jupyter Lab Notebook (S6 File) that renders charts and tables that go deep into research features (author decisions on how they approached the deception detection problem) and what kind of Machine Learning strategies were chosen to respond to the research challenges. Such a Jupyter Lab Notebook exposes the performance levels reported as boxplots.

All those Jupyter Lab Notebooks can be found at GitHub. Here we present only the discussion of our findings, as we consider that our greatest contribution.

From the qualitative perspective, we interpreted the statistical findings according to some theoretical frameworks on deception detection [2, 4, 5]. We discuss how the authors’ approaches align to those frameworks, where they agree and don’t, and what is still to be done. All those comments can be found in the “Discussion” section.

We have the following answers to the research questions:

a) **What are the best performing Machine Learning techniques applied to automatic deception detection?** The Machine Learning techniques that best performed were Decision Trees, Gradient Boosting, Neural Networks, Multi-view learning, Random Forest, and Support Vector Machines (SVM).
b) **What datasets and features do they consume?** Most studies trained their classifiers with mock data, but the adoption of real-life data is increasing; features include verbal and non-verbal cues, mainly facial expressions, gestures, body temperature, prosodic and vocal features, and linguistic patterns; 117 different kinds of features were exploited as deception cues, distributed in nine modalities.
c) **What performance level have they recently achieved?** Performance was measured mostly by accuracy, ranging from 0.51 to 1.0; other performance metrics were F1-score, Area Under the Curve (AUC), Unweighted Average Recall (UAR), Recall, and Precision.

Besides, deception detection was treated as a binary classification problem, except for one case. Many studies were dedicated to a single modality, but multimodal studies seem to become a trend.

### Distribution of retrieved documents

**Fig 1:**
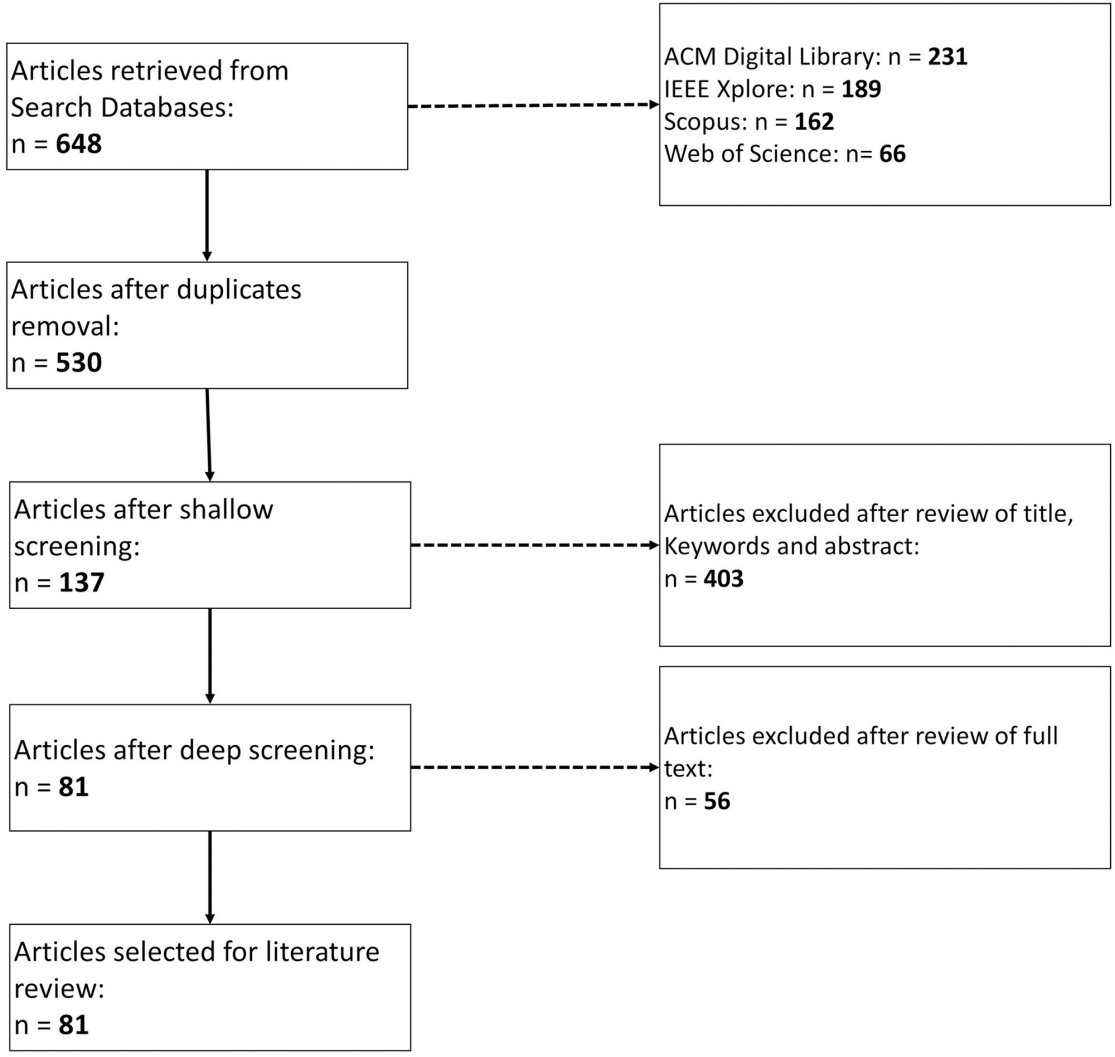
PRISMA flowchart. This PRISMA flowchart presents the main steps of literature selection, deduplication, shallow and deep screening, until the final collection is reached. Each step displays the amount of documents selected so far. Source: The authors (2022).

**Fig 2:**
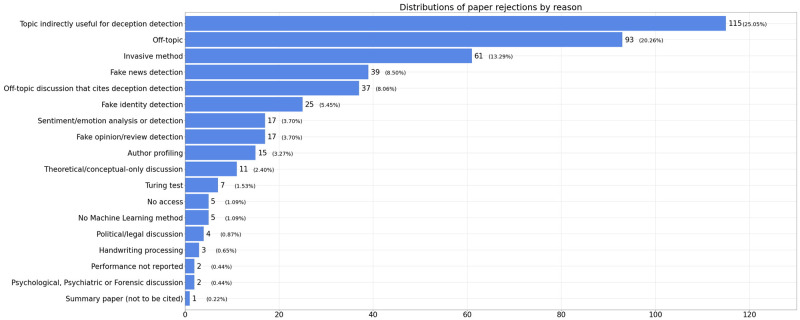
Rejection reasons. Papers that did not meet our selection criteria were rejected. The rejection reasons presented are those recorded for each article during the screening steps. This bar chart presents all those reasons and their frequency. Source: The authors (2022).

The PRISMA (Preferred Reporting Items for Systematic Reviews and Meta-Analyses) flowchart that summarizes the building of the review corpus can be seen in Fig 1. The exclusion reasons for 459 documents are shown in Fig 2.

We drawn mind map summaries from the full reading of the selected documents, as well as we extracted metadata especially intended for statistical analysis.

### The selected documents

Table 2 contains the list of all 81 selected documents. It can be used as a summary for the review. The table is sorted by descending performance and presents the best-performing technique (in some cases, more than one was reported) with the highest performance metric in the first 19 rows (again, in some cases more than one was reported).

**Table 2:** List of all 81 selected documents.

Title	Technique
[2021] **Use of Machine Learning for Deception Detection from Spectral and Cepstral Features of Speech Signals** [38]	Neural Network / Accuracy: 1.0
[2018] **Deception detection using artificial neural network and support vector machine** [39]	SVM / Accuracy: 1.0
[2020] **Automated Deception Detection of Males and Females from Non-Verbal Facial Micro-Gestures** [40]	Random Forest / Accuracy: 0.998
[2019] **Face-Focused Cross-Stream Network for Deception Detection in Videos** [41]	Neural Network / Area Under the Curve: 0.9978
[2018] **A Multi-View Learning Approach To Deception Detection** [42]	Multi-view Learning / Accuracy: 0.98
[2015] **A comparison of features for automatic deception detection in synchronous computer-mediated communication** [43]	Decision Tree / Accuracy: 0.98
[2019] **Robust Algorithm for Multimodal Deception Detection** [44]	Combined methods / Accuracy: 0.97
[2018] **Lie Detector with The Analysis Of The Change Of Diameter Pupil and The Eye Movement Use Method Gabor Wavelet Transform and Decision Tree** [45]	Decision Tree / Precision: 0.97
[2021] **LieNet: A Deep Convolution Neural Networks Framework for Detecting Deception** [46]	Neural Network / Accuracy: 0.967375
[2017] **Deep Learning Driven Multimodal Fusion for Automated Deception Detection** [47]	Neural Network / Accuracy: 0.964
[2019] **How smart your smartphone is in lie detection?** [48]	KNN / Precision: 0.95
[2012] **The Voice and Eye Gaze Behavior of an Imposter: Automated Interviewing and Detection for Rapid Screening at the Border** [49]	Decision Tree / Accuracy: 0.9447
[2020] **Building a Better Lie Detector with BERT: The Difference Between Truth and Lies** [50]	Neural Network / Accuracy: 0.936
[2021] **Deception detection in text and its relation to the cultural dimension of individualism/collectivism** [51]	Logistic Regression / Recall: 0.93
[2018] **Deception detection in videos** [52]	Logistic Regression / Area Under the Curve: 0.9221
[2021] **Development of Spectral Speech Features for Deception Detection Using Neural Networks** [53]	Neural Network / Accuracy: 0.9167
[2012] **Syntactic Stylometry for Deception Detection** [54]	SVM / Accuracy: 0.912
[2020] **Introducing Representations of Facial Affect in Automated Multimodal Deception Detection** [55]	AdaBoost / Area Under the Curve: 0.91
[2014] **Cues to Deception in Social Media Communications** [56]	Gradient Boosting / Accuracy: 0.91
[2020] Your eyes never lie: A robot magician can tell if you are lying [57]	Random Forest / Area Under the Curve: 0.897
[2017] Detecting Deceptive Behavior via Integration of Discriminative Features from Multiple Modalities [58]	Decision Tree / Accuracy: 0.8926
[2021] Affect-Aware Deep Belief Network Representations for Multimodal Unsupervised Deception Detection [59]	Neural Network / Precision: 0.88
[2020] Emotion Transformation Feature: Novel Feature For Deception Detection In Videos [60]	SVM / Accuracy: 0.8759
[2014] Thermal Facial Analysis for Deception Detection [61]	KNN / Accuracy: 0.8688
[2016] ReLiDSS: Novel lie detection system from speech signal [62]	SVM / Accuracy: 0.86375
[2021] Automatic Detection of Deceptive and Truthful Paralinguistic Information in Speech using Two-Level Machine Learning Model [63]	Combined methods / F1-score: 0.856
[2018] Toward End-to-End Deception Detection in Videos [64]	KNN / Accuracy: 0.8416
[2018] Interpretable Multimodal Deception Detection in Videos [65]	Neural Network / Accuracy: 0.8416
[2021] Deception in the eyes of deceiver: A computer vision and machine learning based automated deception detection [66]	SVM / Precision: 0.84
[2018] Detection of Deception Using Facial Expressions Based on Different Classification Algorithms [67]	Neural Network / Accuracy: 0.84
[2015] Distinguishing Deception from Non-Deception in Chinese Speech [68]	Decision Tree / Recall: 0.83555
[2013] Deception detection in speech using bark band and perceptually significant energy features [69]	Neural Network / Accuracy: 0.8333
[2019] Speech Deception Detection Algorithm Based on SVM and Acoustic Features [70]	SVM / Accuracy: 0.8247
[2015] Perinasal indicators of deceptive behavior [71]	Neural Network / Accuracy: 0.8
[2018] An Empirical Study on Detecting Deception and Cybercrime Using Artificial Neural Networks [72]	Neural Network / Area Under the Curve: 0.7999
[2018] Comparative Analysis of Classification Methods for Automatic Deception Detection in Speech [73]	Decision Tree / Unweighted Average Recall: 0.795
[2016] The Truth and Nothing but the Truth: Multimodal Analysis for Deception Detection [74]	SVM / Accuracy: 0.7895
[2018] Acoustic-Prosodic Indicators of Deception and Trust in Interview Dialogues [75]	Random Forest / Precision: 0.7837
[2018] Automated verbal credibility assessment of intentions: The model statement technique and predictive modeling [76]	SVM / Accuracy: 0.7742
[2019] Automatic Deception Detection in RGB Videos Using Facial Action Units [77]	SVM / Accuracy: 0.7684
[2021] How humans impair automated deception detection performance [78]	Random Forest / Recall: 0.76
[2011] Move, and I Will Tell You Who You Are: Detecting Deceptive Roles in Low-Quality Data [79]	SVM / F1-score: 0.76
[2021] Unsupervised Audio-Visual Subspace Alignment for High-Stakes Deception Detection [80]	KNN / Area Under the Curve: 0.75
[2018] Convolutional Bidirectional Long Short-Term Memory for Deception Detection with Acoustic Features [81]	Neural Network / Accuracy: 0.7487
[2019] Joint Learning of Conversational Temporal Dynamics and Acoustic Features for Speech Deception Detection in Dialog Games [82]	Neural Network / Unweighted Average Recall: 0.7471
[2018] Intelligent Deception Detection through Machine Based Interviewing [83]	Neural Network / Accuracy: 0.74605
[2019] Can a Robot Catch You Lying? A Machine Learning System to Detect Lies During Interactions [84]	Random Forest / Area Under the Curve: 0.74
[2013] Automatic Detection of Deceit in Verbal Communication [85]	SVM / Accuracy: 0.737
[2015] Deception Detection Using Real-Life Trial Data [86]	Random Forest / Accuracy: 0.7355
[2021] Detecting Lies is a Child (Robot)’s Play: Gaze-Based Lie Detection in HRI [87]	Random Forest / Area Under the Curve: 0.733
[2016] Deceptive Speech Detection based on sparse representation [88]	SVM / Accuracy: 0.7295
[2020] Multimodal Deception Detection using Real-Life Trial Data [89]	Neural Network / Accuracy: 0.7288
[2021] Identity Unbiased Deception Detection by 2D-to-3D Face Reconstruction [90]	Neural Network / Recall: 0.72
[2012] On the Use of Homogenous Sets of Subjects in Deceptive Language Analysis [91]	SVM / Precision: 0.7185
[2018] Linguistic cues to deception and perceived deception in interview dialogues [92]	Random Forest / Precision: 0.71685
[2019] Automatic Long-Term Deception Detection in Group Interaction Videos [93]	Combined methods / Area Under the Curve: 0.705
[2015] Detection of Deception in the Mafia Party Game [94]	Logistic Regression / Accuracy: 0.7026
[2013] Seeing through Deception: A Computational Approach to Deceit Detection in Written Communication [95]	SVM / F1-score: 0.702
[2014] Deception Detection Using a Multimodal Approach [96]	Decision Tree / Accuracy: 0.701
[2021] Multimodal Political Deception Detection [97]	Decision Tree / Accuracy: 0.7
[2021] Non-invasive Deception Detection in Videos Using Machine Learning Techniques [98]	SVM / Recall: 0.6972
[2015] Experiments in open domain deception detection [99]	SVM / Accuracy: 0.695
[2017] Deception detection in Russian texts [100]	Clustering / Accuracy: 0.683
[2019] High-Level Features for Multimodal Deception Detection in Videos [101]	BSSD / Area Under the Curve: 0.671
[2015] Is Interactional Dissynchrony a Clue to Deception? Insights From Automated Analysis of Nonverbal Visual Cues [Burgoon, J. K.; Yu, X.; Zhang, S.; Yan, Z.; Yang, F.; Huang, J.; Dunbar, N. E.; Jensen, M. L.; Metaxas, D. N.]	SVM / Precision: 0.668
[2018] Deception Detection and Analysis in Spoken Dialogues based on FastText [102]	Neural Network / Precision: 0.667
[2017] Gender-Based Multimodal Deception Detection [103]	Decision Tree / Accuracy: 0.664
[2019] Bag-of-Lies: A Multimodal Dataset for Deception Detection [104]	Combined methods / Accuracy: 0.6617
[2015] Cross-Cultural Production and Detection of Deception from Speech [105]	Random Forest / Accuracy: 0.6589
[2019] Detecting Concealed Information in Text and Speech [106]	Neural Network / F1-score: 0.65615
[2012] Discerning truth from deception: Human judgments and automation efforts [107]	Decision Tree / Accuracy: 0.65
[2011] Challenges in automated deception detection in computer-mediated communication [108]	SMO / Accuracy: 0.65
[2016] Automated detection of user deception in on-line questionnaires with focus on eye tracking use [109]	SVM / Precision: 0.64
[2017] Hybrid Acoustic-Lexical Deep Learning Approach for Deception Detection [110]	Neural Network / F1-score: 0.639
[2019] Improved semi-supervised autoencoder for deception detection [111]	Neural Network / Accuracy: 0.6278
[2021] Deception Detection and Remote Physiological Monitoring: A Dataset and Baseline Experimental Results [112]	SVM / Accuracy: 0.626
[2016] Analyzing Thermal and Visual Clues of Deception for a Non-Contact Deception Detection Approach [113]	Decision Tree / Accuracy: 0.6174
[2017] Construction and Analysis of Indonesian-Interviews Deception Corpus [114]	Random Forest / F1-score: 0.613
[2020] Multilingual Deception Detection by Autonomous Agents [115]	Neural Network / Accuracy: 0.6
[2018] Construction of a Liar Corpus and Detection of Lying Situations [116]	SVM / Accuracy: 0.5516
[2019] Detecting Deception in Political Debates Using Acoustic and Textual Features [117]	Neural Network / Accuracy: 0.5104

Source: The authors (2022)

However, the datasets and experiment setups are too diverse to be compared. Therefore, a direct benchmark of the studies’ performances is not reasonable. We include them here as another feature of those studies, but we do not claim that the specific research that achieved a higher accuracy than other is better. Those performance measures do not work here as a scale of success when approaching the problem, nor do they indicate that a particular approach is better than other. They can work, at best, as a baseline for further research designed under the same conditions.

The 19 studies that achieved accuracy above 0.9 show their title in bold. The threshold, 0.9, was chosen because it is the one Ekman considered as the accuracy level of distinctively performing lie-catchers [5].

However, we stress that a particular study reporting accuracy equals or above 0.9 is not equivalent to a highly accurate human deception detector. Human lie-catchers show their skills in very diverse situations and outside of any controlled environment. The conditions they are subjected to are far more complex than the ones Machine Learning solutions are at this moment.

We only decided to use 0.9 as threshold here because Ekman considered that as an indicator of high-standard performance.

## Discussion

As our contribution to the field, we present a discussion that unfolds in several themes (or dimensions) we consider suitable. Those themes were not chosen. Rather, they arise from the selected documents and represent a general summary of all the efforts analyzed. Those themes are findings themselves. They outline the main topics present in the selected studies regarding the theoretical foundations of deception detection. Authors attempted these approaches to answer to the deception detection problem.

Our goal is to present an abstract notion of the state of each of those dimensions so we could give researchers an insight about each theme. We hope that the following sections will help further studies to direct their effort to fill in the still existing gaps. We report what we have found and discuss it in comparison to deception detection theories, primarily to highlight research opportunities.

Many other conclusions were not included as we considered they do not contribute to answering the research questions. Those conclusions, however, can be found in the Jupyter Lab Notebooks. The following sections present and discuss those themes.

### Language and cultural coverage

We consider this theme important because it divides the studies into two distinct groups: one based on English and another based on other languages. Verbal cues depend heavily on language aspects. Thus, most of the knowledge found in English-based studies needs to be adapted or tested for other languages. Statistical details can be found in section 2 (Language analysis) in S6 File (Statistical Analysis Notebook).

The statistical analysis results make evident the lack of research regarding deception detection in non-English languages. The research on the topic was found only in nine other languages (Chinese, Dutch, Hebrew, Indonesian, Italian, Mandarin, Romanian, Russian, and Spanish), yet the volume is small (14 papers, 25%) when compared to studies dedicated to English (42 papers, 75%). We kept Chinese and Mandarin apart because they were referred to as such. Furthermore, most studies on the aforementioned languages are devoted to vocal cues [38, 63, 68, 70, 75, 80–82, 88, 97, 105, 111, 114, 115].

While facial expressions are universal, gestures are culture-specific [2]. Some visual cues related to gestures lack more experimentation for different cultures. As an example, the same gesture that means “Ok” for the American (connecting the thumb to the tip of the pointing finger) may represent an obscenity for the Brazilian. The messages and emotions related to that same gesture are pretty different. However, no study has taken advantage of this information.

From the 14 studies on non-English [46, 51, 68, 70, 75, 81, 82, 88, 91, 95, 100, 101, 105, 111, 114, 115], only one consumes visual features [101], and none include gestures such as self-adaptors (touching one’s own body, face, or hair) [4], manipulators (pinching, picking, scratching) [2], emblems (gestures that replace words) [2], or illustrators (gestures that accompany speech) [2, 4].

Regarding linguistic cues, one article presents a comprehensive study comparing five languages from different parts of the world [51]. Structural differences demonstrate the need for specific approaches for each language or, at least, a group of similar languages.

Therefore, there is still a large study gap for languages other than English, mainly focusing different modalities, features, tools, and techniques. The same can be said about gestures for non-American cultures.

### Emotional features

Emotional features are important because, according to some authors [2, 4], the act of deceiving triggers emotional states that induce the behavioral alterations that work as deception cues. Statistical details can be found in section 2 (Language analysis) in S6 File (Statistical Analysis Notebook).

Deception is said to be related to three different emotions: guilt, fear, and delight [2]. A deceiver may feel guilty because his/her conscience tells him/her that deceiving is morally wrong. Fear comes when a deceiver is afraid of being caught and having to account for his/her deception, eventually feeling ashamed or humiliated when exposed. However, a deceiver can feel delighted when the act of deceiving leads to the joy of fooling others [4].

Such emotions can cause several behavioral and physiological changes. Guilt may lead a deceiver to avoid eye contact. Fear may generate physiological arousal and result in eye blinking and the use of self-adaptors. It may also cause speech interferences (pauses, errors, repetitions, and hesitations) and influence the voice pitch. Negative emotions such as guilt and fear may also decrease the use of illustrators, whereas delight may cause smiling and increase the movements [4]. Despite emotions and deceiving being interrelated, not many studies exploited sentiments as a detection approach. Most studies use the behavioral alterations caused by the emotional fluctuations instead of emotions.

The selected papers explore several different modalities and features, but only two are directly related to emotions. One is a paper from 2020 that delves specifically into emotion transformation [60] combined with visual features (a bimodal approach, see section 3.4 in S6 File). An SVM classifier trained from visual cues (eye gazing, facial expressions, and hand motions) reached 0.8759 accuracy. Emotions are inferred from visual features. Visual cues were evaluated in space and time to detect both emotion transitions and deception.

The other study presents a monomodal approach (see section 3.3 in S6 File) based on emotional cues [48]. It exploits a mobile app that can monitor the emotion level by noticing the user’s shaking hands. This study reported 0.84 accuracy from a Random Forest classifier but did not measure the specific emotions related to deception.

Two other studies exploited sentiment extracted from textual cues [56] and visual cues [74], but report no particular findings regarding the influence of such feature on deception detection.

Statistical analysis suggests that scrutiny of the emotional effects experienced during deception is still a fertile ground for research.

### Psychological traits

Psychological traits are important because some specific, not-so-usual ones can influence how a deceiver behaves while telling lies. The expected behavioral shifts may not happen in individuals that show these traits. However, most studies do not delve deeply into this feature. Statistical details can be found in section 4.7 (Remaining features analysis) in S6 File (Statistical Analysis Notebook).

Certain people represent an exception to the emotional effects when they are deceiving. Machiavellian people usually look their accuser right in the eye when they are falsely denying something, which contradicts the notion of eye aversion [4, 15]. Thus, the deceiver’s psychological profile may influence their behavior and, consequently, over the cues they give away.

Three studies experimented on psychological features. One consumed NEO-FFI (Neuroticism-Extraversion-Openness Five-Factor Inventory) scores along with demographic and vocal cues [105]. NEO-FFI is a five-factor personality model based on an empirically developed taxonomy of personality traits. This model measures five personality components: Openness to experience, Conscientiousness, Extraversion, Agreeableness, and Neuroticism.

Certain studies confirm that some of the five NEO-FFI dimensions are related to Machiavellian individuals [118, 119] but the papers in question do not report this relationship as the reason for including such features in the experiments.

One paper reports correlations between Extraversion and Conscientiousness, and the ability to deceive, but does not relate it to Machiavellianism. Still, we consider NEO-FFI as a promising set of features for deception detection.

The second study combines the NEO-FFI score with demographic and textual features [92] that worked as features for training Random Forest, Logistic Regression, and SVM classifiers. The paper presents some discussion and conclusions on the textual features, but nothing about the psychological ones.

The latest study also includes NEO-FFI scores, added by traits such as NARS (Negative Attitude towards Robot Scale), Histrionic, and Narcissistic Machiavellianism, as well as visual features [84]. Although Machiavellianism was included in the study, it concludes that one’s psychological profile does not improve detection performance. It seems that psychological profiling is still both an opportunity for research and a point of doubt, as theoretical and experimental conclusions from Machine Learning do not align.

### Cognitive load

Cognitive load is important because it can disclose the unusual mental effort experienced by a deceiver when telling some elaborate lie, which can be exploited as a deception cue.

Besides emotional consequences, deceiving may lead to higher cognitive effort since fabricating an argument is usually more difficult than telling a recollection [1, 4]. Therefore, the cognitive load caused by lying, especially when the stakes are high, may produce behavioral shifts such as speaking slowly or taking too long to respond [120], as well as blinking less and hesitating during speech [4]. Higher cognitive demand also leads to body neglect, resulting in fewer body movements. In such scenarios there may be more gaze aversion, as looking at other people’s eyes can be distracting [16]. This extra mind work stems from the different areas of the brain related to remembering and fabricating a story.

Out of the 81 selected studies, 8 exploit cognitive load as a predictor in many different forms, such as pupil dilation [45, 49, 57, 84], eye blinks [84, 113], body motion [79, 86], time to respond [84], and hesitation [82]. Pupil dilation was reported to have high discriminant power for deception detection. Other features were not said to have any similar contribution.

Nevertheless, exceptions exist. Some people find telling a lie not such a demanding task, perhaps because they do it frequently and successfully [4]. This happens with people who are verbally skilled, or natural liars. No study among those selected has attempted to measure verbal skills and establish a relationship with deception detection, although there are papers that have explored syntax complexity [43, 54, 56, 58, 72, 92, 99, 101, 103, 106].

Vocal studies based on cognitive load use no more than silence gaps as predictor. Visual studies rely on special glasses to acquire images from the eyes to measure displacements of pupils.

Many verbal features are based on LIWC (Linguistic Inquiry Word Count), a text analyzer that provides psycholinguistic categories for words [121]. While those categories worked as features, no attempt to measure the subjects’ verbal skills was found in the selected corpus, making this another opportunity for study.

### Naturality

Naturality is important because those who are lying are not in a natural moment (at least for most people). Learning how this factor is addressed by research helps to understand its contribution to the field.

One consequence of deceiving is the deceiver’s attempt to control his or her own behavior. Liars may be aware of their interlocutors’ intention to detect deception and try to pose what they consider a natural truth-telling behavior [4]. This may lead to an artificial and rehearsed-like behavior, with unusual body rigidity. Moreover, their speech may also sound extremely fluent, with no hesitations or imprecisions, lacking spontaneity [15].

One study includes hesitation as a feature [82], while another explores speaking rate [105]. Neither of them, however, discusses the contribution of those features. No experimental results validate theoretical expectations so far.

### Source of data

The source of data is essential because Machine Learning is highly dependent on the quality and quantity of input data. To reduce bias, the data samples used as input for Machine Learning algorithms must represent the population as closely as possible. Statistical details can be found in section 7.2 (Dataset origin analysis) in S6 File (Statistical Analysis Notebook).

Deception cues are most noticeable when the deceiver is highly motivated to convince the victim [1, 15, 120, 122]. These are circumstances in which the deceiver foresees undesirable consequences.

Most studies based on mock data use positive motivations to stimulate deceptions (for instance, awarding a 20 dollars prize to the best deceiver). In contrast, studies that consume real-life data may use a negative motivation (facing punishments dictated by law). It has been demonstrated that the intensity of motivation is related to physiological changes during deception [120, 122].

The emotional component of deception detection makes the availability of real-life data labeled with ground truth even more critical. The prevalence of mock data (70.15%) over real-life data (29.85%) challenges some of the results since the influence of mock data over the results is unknown. Even so, such results should not be considered invalid. After all, the authors themselves comment that the lack of real-life data should be considered a research limitation. However, laboratory conditions offer control over variables, which can be leveraged in favor of research [4].

Thanks to the public release of the “Real-life Trial Deception Detection Dataset” in 2015 [86], the volume of research with real-life data increased, delivering reports of high-performance results. It is a multimodal dataset, although vocal and textual research are limited to English. Studies exploiting vocal and textual cues from other languages lack a version of the Real-life Trial dataset.

### Facial cues

Facial cues are important because the face is the primary vehicle to express someone’s emotional state. Also, they have been the focus of many studies, for current technology provides many tools for extracting facial features. Statistical details can be found in section 4.2 (Visual features analysis) of S6 File (Statistical Analysis Notebook).

Ekman reports that faking an emotion may be easier (especially for professional actors), but not demonstrating strong ones is almost impossible since some facial expressions involuntarily arise [2]. It is said that these emotions “leak out”, betraying the deceiver.

Facial expressions, micro-expressions, micro-gestures, and affect were analyzed as features in 32 (39.5%) out of the 81 selected papers, with a myriad of performance levels. The highest one reports 0.97 as accuracy [41, 44]. In general, Bimodal and Multimodal approaches show better results than Monomodal ones, a synergy between visual and non-visual cues. These findings demonstrate the importance of visual cues for deception detection.

Eye-related features (gaze, blinks and eye saccades) were exploited in 21 studies (22.78%). Such features were chosen because some studies [2] suggest that the gaze suffers the influence of some emotions, such as sadness or guilt. Since deception is associated with negative emotions and arousal of affect [3], those features seem promising. Likewise, eye blinks are usually increased with the arousal of emotions [4].

Head-related features (head pose and head motion) were exploited in 13 articles (17.58%). Those were chosen because some authors relate the head with deception cues [4]. Head shakes, nods, and head orientation may express emotional states while the lack of movement may indicate a state of self-conscience (self-monitoring) [3].

The face is a dual channel of Information. While there are involuntary actions that may work as clues for detection, the face is one of the body parts most monitored by the deceiver when concealing emotions and mislead the interlocutor [2]. Many studies have measured the importance of such features on detection performance, but none have been able to explain how they influence detection. This is because most of the exploited classifiers are black boxes (SVM and Neural Networks).

Two works [64, 65] proposed a method to interpret visual features. They used an LSTM (Long-Short Term Memory) Neural Network to extract features and a metric named Visual Attention to discriminate those face parts that contributed most to a certain classification. However, other techniques to explain how the results were inferred from the data (named a *post-hoc* explanation) were not exploited [123].

Two others used Decision Tree [113] and Random Forest [86] classifiers. The former surprisingly reports that facial expressions are not discriminant for deception and truth but could not explain why. Such a conclusion seems to contradict theoretical principles. In contrast, the latter reports facial cues as the most contributing set of features for discerning deception from the truth but provides no measure for the contribution of visual features. The first study consumed mock data, and the second real-life data. Consequently, while some results suggest the relevance of facial features, neither study made clear which features are more important and which could be discarded from the feature set, even though Decision Tree methods produce a human-interpretable output.

Given the already mentioned universality of facial expressions, researchers from non-English speaking cultures can take advantage of all these studies as a starting point for their endeavors. As a final comment relative to facial cues and their importance for deception detection, no experiments in the selected corpus tried to put their findings to the test by submitting them to actors.

### Complexity and performance

Complexity and performance are important because the processing power computers offer nowadays allows researchers to invest in more sophisticated and processor-demanding approaches. Some methods that run on a personal computer today were out of possibility 20 years ago.

Data gathered from the reviewed documents allows us to safely claim that there has been an increasing interest on deception detection with Machine Learning in the chosen period. In addition, statistical analysis discloses that the approach complexity also increased (see section 3.2 in S6 File) since different modalities were combined and explored to achieve higher performance levels in different scenarios and under various constraints.

Statistical analysis shows that Monomodal approaches achieved high-performance levels, especially considering that Monomodal studies constitute the majority of research on the topic (33 out of 81 studies, 40.74%). However, a deeper look into such data reveals the presence of outliers (see section 8 in S6 File).

This comes as a surprise since there is a particular group of lie-catchers that show consistently high performance for many kinds of deception in many different situations. These human deception detectors reported using verbal and non-verbal cues, particularly emphasizing the latter [5]. This suggests that Bimodal and Multimodal approaches should always perform better, but some reported results contradict these expectations. This raises questions about what bias could be interfering on such experiments.

Another discrepancy between what the high accuracy detectors said and what the results show is that non-verbal cues are their preference. The detection accuracy levels reported by Monomodal visual studies are not the best, except for some outliers. Monomodal vocal studies present higher accuracy than visual ones. Thus, under a different form, the results still seem to defy the theoretical framework. Reasons for that are yet to be understood.

Statistical analysis reveals that all those textual Monomodal approaches trained their classifiers with data extracted from non-real-life situations and online deception game sessions. Only a few visual and vocal Monomodal studies built their classifiers from real-life data [60, 73, 77].

Could the features extracted from real-life and mock settings be different enough to justify such surprising outcomes? No conclusion can be drawn at this moment, but this doubt raises essential questions that should be answered by future research.

### Machine Learning algorithms

Machine Learning algorithms are important because the area offers a wide range of possibilities for classification problems, not counting clustering and association rules, among others. Statistical details can be found in section 5 (Machine Learning analysis) in the S6 File (Statistical Analysis Notebook).

Authors exploited the Machine Learning arsenal by experimenting with 26 different algorithms. The top five (see section 5.1 in S6 File) is composed by Neural Networks (34 times, 30.09%), Support Vector Machines (SVM) (28 times, 24.78%), Random Forest (20 times, 17.70%), Decision Tree (21 times, 18.58%), and K-Nearest Neighbor (KNN) (10 times, 8.85%). Such algorithms are prevalent and a preference for them is not surprising. AdaBoost (6 papers, 14.63%), Naïve Bayes (6 papers, 14.63%), Logistic Regression (6 papers, 14.63%), and Sequential Minimal Optimization (SMO) (3 papers, 7.32%) come next as the second group of preference. Each of the other 16 (39.09%) algorithms had been exploited once (see section 5 in S6 File).

Four of the top five most exploited Machine Learning techniques present variations (see section 5.2 in S6 File). The variation choice gives an idea of how the authors understand the problem, how complex they expected it to be, and what are their hypotheses regarding the data.

Supervised learning was used in 80 studies since these modeled the deception detection problem as a binary classification task. However, one study [59] addressed the scarcity of labeled ground truth data by proposing an unsupervised model. Such work used a Deep Belief Network (DBN) trained from monomodal e multimodal features to elaborate a clustering system based on a metric they named “facial affect”.

The following sections discuss the most recurring Machine Learning models in the corpus.

#### Artificial Neural Networks and deep learning

Neural Networks were used 34 times (28.44%) in 81 papers. This technique was exploited in 10 different variations. Multi-layer Perceptron (MLP) appeared in 12 papers (35.29%), Long Short-Term Memory (LSTM) networks in 9 papers (26.47%), and Convolutional and Levenberg-Marquardt (MLN) networks in 3 papers (8.82%) each. Another 7 flavors had a single use, and include Autoencoder, BERT, Deep Belief Network, Deep Learning, Multi-input, Recurrent-Convolutional, and Virtual Generalizing RAM.

The MLP model is the older multilayer feed-forward model for Neural Networks. Its popularity come from the late 1980s when the backpropagation training algorithm was introduced [33]. It can model non-linear relationships in data, which seems to be the case for deception detection. One of its virtues is to be trained quickly in nowadays GPU-based computers (mostly, training lasts up to 5 minutes).

The performances of MLP models were measured by accuracy in 11 [39, 40, 43, 56, 66, 67, 71, 72, 83, 89, 102] out of the 12 studies. Those accuracy rates range from 0.6333 to 0.9665, with a mean at 0.7961 ± 0.1130. The other study [68] evaluated performance by F1-score, which measured 0.7633.

Levenberg-Marquardt Networks are a kind of MLP that uses a variation of the backpropagation algorithm aiming to accelerate its convergence. It is also a non-Deep Learning method. The three studies [38, 53, 69] that exploited this kind of Neural Network presented accuracies ranging from 0.7916 to 0.8750, with a mean at 0.8333 ± 0.0417.

On the other hand, LSTMs are Deep Learning recurrent networks that achieve excellent results against problems like time series and Natural Language Processing. This kind of network can model non-synchronic relationships in data. That was the reason for its choice in many studies. Authors hypothesize that the deception cues happen close to each other, but not necessarily simultaneously.

LSTM model performances were measured by accuracy in 5 [38, 50, 53, 65, 81] out the 9 studies. Those accuracies range from 0.7487 to 1.0, with a mean at 0.8886 ± 0.0965. In two cases [106, 110] the measure was F1-score, as 0.6390 and 0.6562. In one study [101] the performance was reported as Area Under the Curve, which measured 0.6650, and in the other [82] the Unweighted Average Recall measured 0.7471.

Another Deep Learning flavor is the Convolutional Neural Network (CNN), which has shown particular success for computer vision. In this model, high-dimensional data is compressed into fewer discriminating features, then processed by hidden layers in a manner similar to MLP.

The CNN model was measured by accuracy in all three studies [46, 47, 90]. These range from 0.6800 to 0.9674 with a mean at 0.8705 ± 0.1650.

Only MLP and MLN are not Deep Learning models, and together they appear in 15 articles (44.11%). All other flavors of Neural Networks (19 papers, 55.89%) use Deep Learning in several variations, revealing a trend of choice. We consider the trend natural given the level of excellence Deep Learning models have shown in the last decade. One of their virtues is that feature selection is automatic.

However, Deep Learning methods rely on large amounts of data to produce results free or with low bias. They also rely heavily on GPU power to be trained, and their architectures can become highly complex. This can be a problem for deception detection since labeled data in unconstrained circumstances is scarce. Then, some authors opted to exploit Autoencoders [80, 111], Deep Learning models that do not consume labeled data. Those are very recent works that may show a promising answer to the data scarcity problem.

The two studies that exploited Autoencoder present accuracies of 0.6278 and 0.6950. For more details, see section 5.3.1 in S6 File.

#### Support Vector Machines

Support Vector Machines (SVM) was the second most prevalent technique across all studies (28 times, 25.69%), and was mostly used with what is called a Linear kernel (23 times, 82.14%). The other choice was a Radial Basis Function (RBF) kernel (5 times, 17.86%).

SVM divides the feature space into optimum hyperplanes and uses them to make decisions [18]. The Linear kernel flavor is used when the data is believed to have linear relationships.

Nonlinear kernels are used when a linear solution is not possible. When working with RBF kernels (also called Gaussian kernels), the feature space is distorted to a higher-dimensional space where a hyperplane can be used to separate it [124].

Among the studies that use Linear SVM, 18 [39, 43, 44, 54, 56, 60, 62, 66, 67, 72, 74, 76, 85, 88, 91, 98, 99, 116] measured their performance by accuracy, ranging from 0.5516 to 1.0, with mean at 0.7752 ± 0.1121. Three studies [68, 79, 95] measured their performance by F1-score, which a range from 0.6012 to 0.7800 and mean at 0.7061 ± 0.0709. One study [52] reported the Area Under the Curve as 0.9034, and another [125] precision as 0.6680 and recall as 0.6590.

The five studies that used RBF SVM [70, 77, 89, 109, 112] measured their performances by accuracy, which ranges from 0.5650 to 0.8247, with mean at 0.6808 ± 0.1101. For more details, see section 5.3.2 in S6 File.

#### Random Forest

This technique is an example of an ensemble model. It combines several models to produce a better one [124]. Random Forest does not have different flavors. The final model is a composite of several randomly generated decision trees, all combined to make the final prediction [19]. Its main advantage over decision trees is that the final model usually overfits less.

Among the 20 studies that exploited Random Forest, 16 [40, 48, 56, 60, 66, 73, 75, 77, 78, 84, 86, 89, 92, 98, 105, 114] measured their performance by accuracy, which ranges from 0.5677 to 0.9980, with mean at 0.7301 ± 0.1182. Three other studies [52, 57, 87] measured their performance by Area Under the Curve, which values 0.7330, 0.8131, and 0.8970. One last paper [106] reported an F1-score of 0.5963. For more details, see section 5.3.2 in S6 File.

#### Decision Trees

Decision Trees models divide the feature space to build a series of partitions organized hierarchically into conditions [19]. The final model is human-readable, which makes this technique attractive because it helps to understand the relationships existing in data.

Decision Trees appeared 21 times (18.58%), among which 19 [40, 43, 45, 49, 58, 60, 71, 73, 86, 96–98, 103, 105, 107, 108, 113] in its vanilla flavor (the technique as originally proposed). Those measured performance by accuracy, which ranges from 0.5708 to 0.9800, with a mean at 0.5708 ± 0.1370. One study [52] measured the performance by Area Under the Curve as 0.8074, and the other [68] reported an F1-score of 0.8095.

One study [48] exploited a variation named Random tree and reported accuracy as 0.8200, and another [68] a Gradient-boosted version and reported an F1-score performance of 0.8207. For more details, see section 5.3.4 in S6 File.

#### K-Nearest Neighbor

K-Nearest Neighbor (KNN) uses an arbitrary number of *k* neighbor points to predict the new data class. A voting process is used, so the majority of neighbors will determine the class of the new data point [124].

KNN appeared 10 times (8.85%), among which eight [43, 60, 61, 67, 72, 73, 80, 98] in its vanilla flavor. Those measured performance by accuracy, which ranges from 0.5723 to 0.8688, with a mean at 0.7527 ± 0.1029.

One study [64] exploited a variation named Large Margin Nearest Neighbor and reported an accuracy as 0.8416. Another different version was IB1 [48], which reported accuracy as 0.8100. For more details, see section 5.3.5 in S6 File.

#### Heterogeneous approaches

Most multimodal works used the same algorithms for all the modalities, but four studies [44, 63, 93, 104] exploited combining different algorithms for different modalities and demonstrated that such a decision improved their results.

Those studies hypothesize that different algorithms better process different sets of features. Thus, they exploited a kind of ensemble classifier, having each algorithm dedicated to a specific modality.

### Dataset benchmark

A dataset benchmark is essential because Machine Learning dramatically depends on both data quality and volume to perform well.

The S6 File (Statistical analysis Jupyter Notebook) provides many charts and tables that describe the datasets in various facets, including a table that lists each paper with details about the dataset it consumes. Those details include cardinality, origin, access, modalities, features, and applied algorithms.

The “Real-life Trial Deception Detection Dataset” was used in 17 of the 81 studies. It is a well-balanced, 121-row dataset built from videos collected from YouTube. Each video section was labeled as true or deceptive based on police evidence. In four cases, the studies used a subset of the dataset, and in two others, a superset.

The largest real-life dataset has 6,733 instances, the shortest has 6 instances. Among the non-real-life datasets, the largest has 137,640 instances and the shortest has 40 instances.

### Current state and further research

Actors specialize in displaying fake emotions, and the face plays an essential role in this context. Would they be able to mislead an already trained Machine Learning Deception Detector? Ekman talks about how to detect false emotions [2], but not one study included that in their research.

It has been suggested that there is no relationship between detection accuracy and demographic aspects (age, gender, or profession) of the human lie-catcher [7], except for secret service agents, who show a correlation between accuracy, profession, and experience. High-accuracy catchers exploit different cues than those with lower performance, suggesting that specific cues carry important information about deception.

Although profession does not seem to be related to lie-catching skills, even high-performance catchers show a particular ability with certain kinds of deception, having a performance decrease when faced with other kinds [5, 15]. The conclusion is that certain kinds of lies produce different cues than others, and the experience on detecting a given kind of deception does not guarantee skills to detect others.

Moreover, situational and idiosyncratic factors can affect the subjects’ behavior and, therefore, which cues are leaked when deceiving. Such cues are more specific and challenging to detect [4]. Not considering these factors can decrease the detection accuracy—a situation referred to as the Brokaw hazard [2].

This finding works as a reasonable explanation for the variety of experiment results. While there are several reliable deception clues, exceptions exist because they may suffer from certain interferences, particularly the so-called Othello error [2]. The Othello error occurs when lie-catchers confuse emotions and motivations. The emotion is present, but it does not originate from deception.

This is a strong stimulus for further research and efforts to produce labeled datasets from actual data under more diverse circumstances. More cues could be identified and related to particular settings. Fake expressions from actors could be an important addition to the datasets.

Superior lie-catchers seem to acquire their ability from a personal desire to perform better on their job, no matter what it is [5]. It is like any other professional skill or talent, improved through effort, dedication, personal interest, technical knowledge, and training. Thus, such highly skilled lie-catchers result from intense dedication, which is a motivating factor for further research on deception detection. It is reasonable to believe that those levels of accuracy can be approximated or even replicated by a Machine Learning classifier given the correct cues are processed and interpreted.

The diversity of circumstances lie-catchers face improves and generalizes their abilities. This shows the importance of having labeled real-life data collected from diverse sources, including children and people under medical and psychological treatment, police interrogations, and witnesses in a trial. This creates another research gap to be filled.

The variety of different Machine Learning techniques suggests that the field is still being explored, although there are some high-performance results (Table 1). We believe that the variety of feature kinds exploited suggests that authors are still uncertain about which ones are the most informative. They are gauging the potential of certain cues as deception indicators. Different modality cardinalities and combinations and the plethora of features are evidence that the topic still offers room for research.

## Limitations and further work

This research aims to present a comprehensive overview of the state of knowledge about deception detection with Machine Learning, paying special attention to the performance level reported by each study and what data and features they consumed to achieve that performance. However, due to time restrictions and the number of researchers, the period of interest was limited to 2011–2021. It is known that some studies were excluded from the corpus, but we consider this acceptable since the last decade shows a great evolution of the field.

In addition, other scientific sources such as Google Scholar, Semantic Scholar, and Connected Papers were not queried for the same reasons above. They could have provided other papers to complement the current corpus.

We intend to expand our protocol to include those scientific sources, and improve BiblioAlly to handle them. Once new documents are included and analyzed, new versions of the metadata will be made available to provide a more complete panorama of the field.

## Conclusions

This literature review aims to comprehensively overview of the application of Machine Learning to deception detection by reporting on techniques, approaches, data, and performance levels. We searched, retrieved and selected papers, which were summarized as mind maps. We extracted metadata and encoded it as Python dictionaries. All are available for free access.

A total of 648 bibliographic references were retrieved, with 540 being screened (108 were duplicates). We selected only those that have results directly related to deception detection with Machine Learning. Such studies had to present the data features they consumed and the performance level achieved. Only non-invasive approaches were accepted. The final corpus (81 documents) reports the results of experiments on deception detection with Machine Learning. BiblioAlly was an important asset for conducting the study, helping to manage and track the steps of the process.

We could reach several conclusions from the findings of this review:

a) Authors modeled deception detection as a classification problem (supervised learning), except for one case that proposed a clustering-based solution (unsupervised learning);
b) The volume of production on the topic suggests a progressive increase of interest;
c) The preference for monomodal studies has changed to bimodal and multimodal, over time;
d) Features exploited are variated and include mostly language and culture, emotion exploitation, psychological traits, cognitive load, many facial cues, complexity, performance, and various Machine Learning algorithms;
e) The absolute majority of works that exploit verbal and vocal features are dedicated to English; there is a clear gap for other languages and cultures;
f) While the theory on deception detection strongly relates it to the subject’s emotional state, most studies did not approach the problem under this perspective, rather modeling the features from behavioral changes;
g) Machiavellianism is a psychological trait that can change the interpretation of detection cues, but authors did not exploited it;
h) Cognitive load was exploited mostly from the pupil dilation with promising results, but eye saccades, head motions, and syntax complexity also appeared;
i) Facial cues were exploited in many ways by many works with a variety of feature sets; OpenFace was the most used supporting tool for these works;
j) Vocal cues were almost exclusively provided by OpenSMILE and in general are reported as a highly discriminant feature set;
k) Naturality was exploited by hesitation and speaking rate, but not much was reported as the contribution of this sort of cue;
l) Most studies consumed mock data, but after the release of the “Real-life Trial Deception Detection Dataset” there is an increase of papers that consume it;
m) The scarcity of real-life labeled data with open access still stands as a major challenge for the field;
n) Neural networks, Support Vector Machines, Logistic Regression, K-Nearest Neighbor and Decision Trees were the most exploited Machine Learning algorithms;

The variation of size, source, and features of the data consumed is so high that it’s impossible compare works results. Although a multitude of distinct approaches had been tested with several performance levels for over a decade, the field still seems to be at initial stages of development.

Industry application looks premature at present. Most experiments are based on mock data, and even those operating on real-life data are restricted to particular cultures and circumstances.

We assess that the risk of bias is high since the datasets used are recurrent and neither large nor diverse enough to provide a highly general classifier. There are also no reports on methods tested under real-world scenarios. This perception is strengthened by the high risk of bias identified in many of the studies by applying PROBAST.

As a result, the overall conclusion is that there is still room for novel approaches, especially based on real-life data from non-English, and from different cultures. Results seem to be promising, as some experiments report a very high accuracy level.

Replicating the performance of human lie-catchers may be considered possible if the topic receives investment. Experiments with more data collected from real, every day, and diverse conditions would produce more robust solutions and raise results and techniques to a level of potential industrialization and commercialization.

## Supporting information

S1 File AMSTAR-2 tool for quality assessment. Source: The authors (2022).
(PDF)

S2 File PROBAST tool for risk of bias assessment. Source: The authors (2022).
(XLSX)

S3 File 1-Definition.pdf Jupyter Lab Notebook. Source: The authors (2022).
(PDF)

S4 File 2-Corpus collection.pdf Jupyter Lab Notebook. Source: The authors (2022).
(PDF)

S5 File 3-Corpus analysis.pdf Jupyter Lab Notebook. Source: The authors (2022).
(PDF)

S6 File 4-Statistical analysis Jupyter Lab Notebook. Source: The authors (2022).
(PDF)

S7 File 5-Mindmaps Jupyter Lab Notebook. Source: The authors (2022).
(PDF)

S1 Checklist PRISMA 2009 checklist. Source: The authors (2022).
(PDF)
