# Supplementary material for: Deception detection with machine learning: A systematic review and statistical analysis
Source: PLoS One. 2023 Feb 9;18(2):e0281323. doi: 10.1371/journal.pone.0281323 (PMC9910662; doi:10.1371/journal.pone.0281323)
Supplement: S3 File — Source: The authors (2022). (PDF) [file pone.0281323.s003.pdf]

Deception Detection supported by Machine Learning

Literature review

Introduction

This Jupyter Notebook is part of the Literature Review Project and aims to disclose the data collected along the process, and work as a memory of all the steps taken as well.

Currently, the manuscript of the scientific article that discusses the consequences and findings of the Literature Review was submitted to the Scientific Journal PLOS One (https://journals.plos.org/plosone/) and is waiting for a response from the peer reviewers.

Research team and contribution

Conceptualization

1. Alex Sebastião Constâncio
2. Denise Fukumi Tsunoda
3. Deborah Ribeiro Carvalho **Data curation**
4. Alex Sebastião Constâncio
5. Denise Fukumi Tsunoda **Formal analysis**
6. Alex Sebastião Constâncio **Investigation**
7. Alex Sebastião Constâncio
8. Denise Fukumi Tsunoda **Methodology**
9. Alex Sebastião Constâncio
10. Deborah Ribeiro Carvalho
11. Helena de Fátima Nunes Silva
12. Jocelaine Martins da Silveira **Software**
13. Alex Sebastião Constâncio **Writing – original draft**
14. Alex Sebastião Constâncio **Writing – review and editing**
15. Deborah Ribeiro Carvalho
16. Denise Fukumi Tsunoda
17. Helena de Fátima Nunes Silva
18. Jocelaine Martins da Silveira **Supervision**
19. Deborah Ribeiro Carvalho
20. Helena de Fátima Nunes Silva

Research scope and objectives

1. Research goals

The goal of this literature review is to capture a panoramic view of the state of research on **Deception Detection supported by Machine Learning**, in order to be able to understand trends, results and gaps on the field.

2. Research question

What are the best performing Machine Learning techniques applied on **automatic Deception Detection**, what features they consume and what level of performance have they recently achieved??

3. Research restrictions

1. Period of interest is 2011-2020;
2. Only non-invasive methods and techniques will be reviewed; by non-invasive, we mean methods that absolutely do not touch the subject nor submit him/her to be evaluated by an equipment less mobile then a regular computer;
3. Only studies that report some kind of performance level achieved.

Research protocol

1. Run queries on selected scientific document bases:
    - Web of Science:
        - `(("deception detection" OR "lie detection") AND ("machine learning" OR "artificial intelligence")) Refined by: Publication years: (2021 OR 2020 OR 2019 OR 2018 OR 2017 OR 2016 OR 2015 OR 2014 OR 2013 OR 2012 OR 2012 OR 2011)`
    - Scopus:
        - `TITLE-ABS-KEY (("deception detection" OR "lie detection") AND ("machine learning" OR "artificial intelligence")) AND (LIMIT-TO (PUBYEAR, 2021) OR LIMIT-TO (PUBYEAR, 2020) OR LIMIT-TO (PUBYEAR, 2019) OR LIMIT-TO (PUBYEAR, 2018) OR LIMIT-TO (PUBYEAR, 2017) OR LIMIT-TO (PUBYEAR, 2016) OR LIMIT-TO (PUBYEAR, 2015) OR LIMIT-TO (PUBYEAR, 2014) OR LIMIT-TO (PUBYEAR, 2013) OR LIMIT-TO (PUBYEAR, 2012) OR LIMIT-TO (PUBYEAR, 2011))`
    - ACM DL:
        - `[All: "deception detection"] OR [All: "lie detection"] AND [Publication Date: (01/01/2011 TO *)]`
    - IEEE Xplore:
        - `(("All Metadata":"deception detection") OR "All Metadata":"lie deception") Filters Applied: 2011 - 2021`
2. Export results as BibTeX files
3. Import all BibTeX files into BiblioAlly; those documents are tagged as "IMPORTED" or "DUPLICATE"
4. Manually detect duplications not detected during import and tag them as "DUPLICATE"
5. Pre-select articles by shallow screening:
    - Read title, keywords and abstract
    - Reject studies that violate research restrictions
    - Rejected ones are tagged "EXCLUDED", remaining ones are tagged "PRE-ACCEPTED"
6. Retrieve the full-text of pre-selected documents
7. Select articles by deep screening
    - Read full text
    - Reject studies that violate research restrictions
    - Rejected ones are tagged "EXCLUDED", remaining ones are untagged "PRE-ACCEPTED" and tagged "SELECTED"
8. Extract relevant data from accepted documents
    - Build the mental model representing the relevant summary of the paper as a FreeMind document
    - Register the meta-information that will serve for further meta-analysis
9. Run a meta-analysis and generate charts and tables
10. Assess Risk of Bias and research quality

Data extraction

After reading the full text of selected papers, each were summarized in two forms:

1. **Mind map**: a graphical summarized form of the study;
2. **Python dictionary**: an encoded version of the extracted meta-data of interest that can be further computed to produce statistics, charts and tables.

Details on each one below.

Mind maps

Mind maps are FreeMind documents, manually produced, since BiblioAlly still can't do it automatically (for now we can dream about it, right?). Those mind maps were built to serve as a quick and short summary of the entire article and helped during reading and reviewing their full text.

Those maps describe the study hypothesis, the contributions, the dataset, the feature modalities, the methods used, and the performance achieved.

Meta-data encoding

Each article was structured as follows:

1. `document_id`: the document id in the BiblioAlly database;
2. `methods`: list of methods and tools used in the paper, each item is described as `classifier` or `support`:
    0. `classifier`: describes the classification algorithm as:
        0. `kind`: when appliable, describes some kind or sub-category of the method;
        1. `implementation`: package used as algorithm implementor;
        2. `training`: training method used;
        3. `performance`: performance achieved by the classifier described as:
            0. `kind`: the performance measure used;
            1. `value`: the performance level achieved;
    1. `support`: describes supporting tools used for some generic purpose;
3. `dataset`: description of the dataset used in the study:
    0. `public`: True indicates a freely accessible dataset, False the opposite;
    1. `mock`: True indicates a dataset collected from some fabricated setting, False means data collected from real-life events;
    2. `name`: name of the dataset;
    3. `size`: number of rows listed in the dataset;
    4. `origin`: source of the data;
    5. `target`: labels used in the target attribute;
    6. `features`: list of feature kinds in the dataset:
        0. `kind`: the kind of detection cue features;
        1. `dimensions`: the number of features;
        2. `components`: list of feature components;
        3. `language`: list of languages, when appliable;
        4. `tool`: list of tools, when appliable;
4. `notes`: textual notes about the study;
5. `mindmap`: file name of the mind map document.
