# Supplementary material for: Deception detection with machine learning: A systematic review and statistical analysis
Source: PLoS One. 2023 Feb 9;18(2):e0281323. doi: 10.1371/journal.pone.0281323 (PMC9910662; doi:10.1371/journal.pone.0281323)
Supplement: S4 File — Source: The authors (2022). (PDF) [file pone.0281323.s004.pdf]

Deception Detection supported by Machine Learning

Literature review - Data collection and selection

Setup

Instantiating a BiblioAlly Catalog in a SQLite file **DeceptionDetection.db**

```
import BiblioAlly as ally

base_path = "./"
base_file = base_path + "DeceptionDetection.db"
base = ally.Catalog(base_file)
```

Step 1 - Running queries on scientific online search engines

This process is not yet supported by BiblioAlly. At this moment we have to use many search engines and run the queries ourselves.

Reminder: Many search engines provide an API that could be used to automate this step!

Step 2 - Export results as BibTeX files

This process is not yet supported by BiblioAlly and is only needed because BiblioAlly still can't run queries directly. So, we have to collect the results of our queries and save them as BibTex files that will further be imported into the BiblioAlly Catalog.

Step 3 - Import all BibTeX files into BiblioAlly

Importing BibTex files from Web of Science, Scopus, ACM Digital Library and IEEEXplore after they were exported from their original search engine pages. Here we start to load data into BiblioAlly.

All imported documents are tagged as **IMPORTED**. BiblioAlly will try to spot duplicates and tagged them as **DUPLICATE**. The duplicates are still imported, but they are appropriately marked and a reference to the existing document is set so this can be feed some statistics.

BiblioAlly strips all non-alphanumeric characters from the article's title, convert it to lowercase and calculates a CRC32. That CRC32 is used to detect the duplicates.

```
import BiblioAlly.acmdl as acm
import BiblioAlly.ieee as ieee
import BiblioAlly.scopus as scopus
import BiblioAlly.wos as wos

refs_path = base_path + 'Refs\\'
loadCount, fileCount, baseCount = base.import_from_file(wos.WebOfScience, refs_path + 'WoS\\refs.bib')
print(f"Web os Science: File={fileCount} Load={loadCount} Base={baseCount}")
loadCount, fileCount, baseCount = base.import_from_file(scopus.Scopus, refs_path + 'Scopus\\refs.bib')
print(f"Scopus        : File={fileCount} Load={loadCount} Base={baseCount}")
loadCount, fileCount, baseCount = base.import_from_file(acm.AcmDL, refs_path + 'AcmDL\\refs.bib')
print(f"ACM Dig Lib   : File={fileCount} Load={loadCount} Base={baseCount}")
loadCount, fileCount, baseCount = base.import_from_file(ieee.IeeeXplore, refs_path + 'IeeeXplore\\refs.bib')
print(f"IEEE Xplore   : File={fileCount} Load={loadCount} Base={baseCount}")
```

Step 4 - Manual duplicates detection

List all documents not marked as duplicate, sort them by title and manually inspect the list to spot remaining duplicates. After that, we decide which one is the duplicate, then manually mark the duplicates as such and update the base. Unfortunately, BiblioAlly still misses some duplicates.

Reminder: improve duplicate finder to avoid this step.

```
import pandas as pd
import re

pattern = re.compile('[\W_]+')
def code_title(title):
    return pattern.sub('', title).lower()

def only_duplicates(documents):
    doc_dict = dict()
    for doc in documents:
        if doc.code_title not in doc_dict:
            doc_dict[doc.code_title] = [doc]
        else:
            doc_dict[doc.code_title].append(doc)
    only_dups = []
    for docs in doc_dict.values():
        if len(docs)>1:
            only_dups += docs
    return only_dups

non_duplicates = base.documents_by(tagged_as=domain.TAG_IMPORTED, untagged_as=domain.TAG_DUPLICATE)
for doc in non_duplicates:
    doc.code_title = code_title(doc.title)

duplicates = only_duplicates(non_duplicates)
duplicates.sort(key=lambda document: document.code_title)

dups_dict = [{'id': d.id, 'key': d.external_key, 'year': d.year, 'title': d.title, 'authors': [a.author.long_name for a in
d.authors]} for d in duplicates]
dups_df = pd.DataFrame(dups_dict)
pd.set_option('display.max_rows', None)
pd.set_option('display.max_colwidth', None)
dups_df
```

```
# Now that we decided which is duplicate and which is not, we select the duplicates to mark them as such and
# reference the original documents.
doc_ori_dups = [('742412', 'VanDerWalt201641'), ('10.5555/2615731.261748', 'ISI:00037199040001'), ('vanDitmarsch202046',
'ISI:00051732150000'),
                ('ISI:00055057660002', 'Vogel201928'), ('ISI:00052556300000', 'Tortora202'), ('ISI:00055522520000', 'Sánchez-
Monedero202')]

for key_ori, key_dup in doc_ori_dups:
    doc_ori = base.document_by(external_key=key_ori)
    doc_dup = base.document_by(external_key=key_dup)
    base.tag(doc_dup, domain.TAG_DUPLICATE)
    doc_dup.original_document = doc_ori
base.commit()
```

Step 5 - Shallow screening

There is a simple user interface for this, so each **IMPORTED** document is shown so it can be evaluated based on "Title", "Keywords" and "Abstract". If a reference does not fit the research goals, it is marked as **EXCLUDED** and an exclusion reason is assigned to it so we can have some further statistics. On the other hand the paper is untagged **IMPORTED** and tagged as **PRE-ACCEPTED**.

```
import BiblioAlly.gui as gui

browser = gui.Browser(base)
browser.show()
```

Step 6 - Download PRE-ACCEPTED documents

Since BiblioAlly still cannot automatically download the documents, we go for them manually. We have to go for each of the accepted documents to read them entirely. We do this manually since at this moment BiblioAlly is unable to use any APIs to retrieve the documents automatically. So, we list all the pre-accepted documents and manually run some queries in IEEE Xplore, ACM Digital Library or Scholar Google to find and download the corresponding PDF files.

All downloaded documents are renamed to the following pattern: YEAR[AUTHOR]TITLE, where:

1. **YEAR**: publication year, so documents can be sorted by year in the file system;
2. **AUTHOR**: the surname of the first author;
3. **TITLE**: title of the document.

Let's list them in chuncks of 10 and get them by DOI or title, what works first.

NOTE: Because CAPES (Brazilian Scientific Research Regulator/Sponsor) enable us to have access to IEEE Xplore and ACM Digital Library content, in general we can download even those documents that are not published as Open Access.

```
pre_accepted_docs = base.documents_by(tagged_as=domain.TAG_PRE_ACCEPTED)
pre_accepted_docs.sort(key=lambda doc: [doc.year, doc.title])

print(f'{len(pre_accepted_docs)} documents to download and read:\n')
start = 1
for index in range(len(pre_accepted_docs)):
    doc = pre_accepted_docs[index]
    print(f'{index+1:3} [{doc.id:3}] {doc.year}: {doc.generator:20} {doc.title} ({doc.doi})')
```

Step 7 - Deep screening

Now we read all the PRE-ACCEPTED documents in full, decide either to keep or not each one and, for those ones that meet the research goals, a number of meta-data is extracted and a FreeMind mind map is built as a visual summary of the content.

```
import BiblioAlly.gui as gui

browser = gui.Browser(base)
browser.show()
```

Step 8 - Extract relevant data from selected papers

At this moment BiblioAlly does not have any features that make this step easier. We basically have to read the documents, build the mind maps and extract the relevant meta-data.

Step 9 - Summarize selected papers

Here we summarize all the readings so we can have some statistics for the review.

Details on this step are located in the 3-Meta-analysis notebook.

```
# Document references from IeeeXplore and Scopus do not have author's full name, so we will correct that one-by-one based on
# the names directly collected from the PDF files. The code below updates what is needed.

def update_long_names(document_id, names, correct_short_name=False):
    print(f'Document ID => {document_id}')
    document = base.document_by({'id': document_id}, return_first=True)
    if document is None:
        print('Document not found! ABORTING')
        return
    for author in document.authors:
        if correct_short_name:
            parts = author.longName.split()
            new_short_name = parts[-1]+', '+' '.join([p[0]+'.' for p in parts[0:len(parts)-1]])
        author.longName = names[author.shortName]
        if correct_short_name:
```

```
            author.shortName = new_short_name
        base.save_to_db(author)

def adjust_long_names(document_id):
    print(f'Document ID => {document_id}')
    document = base.document_by({'id': document_id}, return_first=True)
    if document is None:
        print('Document not found! ABORTING')
        return
    for author in document.authors:
        if author.longName is None:
            print(f'{author.shortName} has no long name! ABORTING')
            continue
        if ',' not in author.longName:
            continue
        parts = author.longName.split(',')
        new_long_name = parts[-1].strip()+' '+' '.join([p.strip() for p in parts[0:len(parts)-1]])
        author.longName = new_long_name
        base.save_to_db(author)

print('AJUSTING LONG NAMES')
document_ids = [13,19,24,26,28,31,33,37,40,53,61,76,81,107,116,119,130,149,172,174,178,179,180,181,184,189,194,196,199,204,208,
214,218,221,224,234,245,250,384,385,388,389,394,395,396,398,401,402,403,410,415,422,423,426,429,432,433,434,436,
439,448,451,458,463,467,479,483,493,501,504,527,546
]
for document_id in document_ids:
    adjust_long_names(document_id)


print('\nCORRECTING LONG NAMES')
names = {
    'Crockett, K.': 'Keeley Crockett',
    'OShea, J.': 'James O’Shea',
    'Khan, W.': 'Wasiq Khan',
}
update_long_names(53, names)

names = {
    'Mizanur R. M.': 'Md. Mizanur Rahman',
    'Shome, A.': 'Atanu Shome',
    'Chellappan, S.': 'Sriram Chellappan',
    'Alim A. I. A.': 'A. B. M. Alim Al Islam',
}
update_long_names(76, names)

names = {
    'Fu, H.': 'Hongliang Fu',
    'Lei, P.': 'Peizhi Lei',
    'Tao, H.': 'Huawei Tao',
    'Zhao, L.': 'Li Zhao',
    'Yang, J.': 'Jing Yang',
}
update_long_names(81, names)

names = {
    'Warnita, T.': 'Tiffani Warnita',
    'Lestari, D.': 'Dessi Puji Lestari',
}
update_long_names(107, names)

names = {
    'Wu, Z.': 'Zhe Wu',
    'Singh, B.': 'Bharat Singh',
    'Davis, L.': 'Larry S. Davis',
    'Subrahmanian, V.': 'V. S. Subrahmanian',
}
update_long_names(116, names)

names = {
    'Levitan, S.': 'Sarah Ita Levitan',
    'Maredia, A.': 'Angel Maredia',
    'Hirschberg, J.': 'Julia Hirschberg',
```

```
}
update_long_names(119, names)

names = {
    'Litvinova, O.': 'Olga Litvinova',
    'Litvinova, T.': 'Tatiana Litvinova',
    'Seredin, P.': 'Pavel Seredin',
    'Lyell, J.': 'John Lyell',
}
update_long_names(130, names)

names = {
    'Pérez-Rosas, V.': 'Verónica Pérez-Rosas',
    'Mihalcea, R.': 'Rada Mihalcea',
}
update_long_names(149, names)

names = {
    'Rubin, V.': 'Victoria L. Rubin',
    'Conroy, N.': 'Niall J. Conroy',
}
update_long_names(174, names)

names = {
    'Fornaciari, T.': 'Tommaso Fornaciari',
    'Poesio, M.': 'Massimo Poesio',
}
update_long_names(224, names)

names = {
    'S. V.': 'Sushma Venkatesh',
    'R. R.': 'Raghavendra Ramachandra',
    'P. B.': 'Patrick Bours',
}
update_long_names(384, names, True)

names = {
    'H. T.': 'Huawei Tao',
    'P. L.': 'Peizhi Lei',
    'M. W.': 'Mengzhe Wang',
    'J. W.': 'Jie Wang',
    'H. F.': 'Hongliang Fu',
}
update_long_names(385, names, True)

names = {
    'J. P.': 'Jinie Pak',
    'L. Z.': 'Lina Zhou',
}
document = base.document_by({'id': 388}, return_first=True)
update_long_names(388, names, True)

names = {
    'N. S.': 'Nidhi Srivastava',
    'S. D.': 'Sipi Dubey',
}
update_long_names(389, names, True)

names = {
    'H. N.': 'Hanen Nasri',
    'W. O.': 'Wael Ouarda',
    'A. M. A.': 'Adel M. Alimi',
}
update_long_names(394, names, True)

names = {
    'J. -. Y.': 'Jun-Teng Yang',
    'G. -. L.': 'Guei-Ming Liu',
    'S. C. .. -. H.': 'Scott C.-H Huang',
}
update_long_names(395, names, True)
```

```
names = {
    'S. S.': 'Sarun Sumriddetchkajorn',
    'A. S.': 'Armote Somboonkaew',
    'T. S.': 'Tawee Sodsong',
    'I. P.': 'Itthipol Promduang',
    'N. S.': 'Niti Sumriddetchkajorn',
    'T. P.': 'Thawatchai Prada-in',
}
document = base.document_by({'id': 396}, return_first=True)
update_long_names(396, names, True)

names = {
    'U. M. S.': 'M. Umut Sen',
    'V. P.': 'Verónica Pérez-Rosas',
    'B. Y.': 'Berrin Yanikoglu',
    'M. A.': 'Mohamed Abouelenien',
    'M. B.': 'Mihai Burzo',
    'R. M.': 'Rada Mihalcea',
}
update_long_names(398, names, True)

names = {
    'J. O.': 'James O’Shea',
    'K. C.': 'Keeley Crockett',
    'W. K.': 'Wasiq Khan',
    'P. K.': 'Philippos Kindynis',
    'A. A.': 'Athos Antoniades',
    'G. B.': 'Georgios Boultadakis',
}
update_long_names(401, names, True)

names = {
    'R. R.': 'Rodrigo Rill-García',
    'H. J. E.': 'Hugo Jair Escalante',
    'L. V.': 'Luis Villaseñor-Pineda',
    'V. R.': 'Verónica Reyes-Meza',
}
update_long_names(402, names, True)

names = {
    'H. K.': 'Hamid Karimi',
    'J. T.': 'Jiliang Tang',
    'Y. L.': 'Yanen Li',
}
update_long_names(403, names, True)

names = {
    'H. C.': 'Huang-Cheng Chou',
    'Y. L.': 'Yi-Wen Liu',
    'C. L.': 'Chi-Chun Lee',
}
update_long_names(410, names, True)

names = {
    'Y. X.': 'Yue Xie',
    'R. L.': 'Ruiyu Liang',
    'H. T.': 'Huawei Tao',
    'Y. Z.': 'Yue Zhu',
    'L. Z.': 'Li Zhao',
}
update_long_names(415, names, True)

names = {
    'C. B.': 'Chongyang Bai',
    'M. B.': 'Maksim Bolonkin',
    'J. B.': 'Judee D. Burgoon',
    'C. C.': 'Chao Chen',
    'N. D.': 'Norah Dunbar',
    'B. S.': 'Bharat Singh',
    'V. S. S.': 'V. S. Subrahmanian',
    'Z. W.': 'Zhe Wu',
}
```

```
update_long_names(422, names, True)

names = {
    'B. A. R.': 'Bashar A. Rajoub',
    'R. Z.': 'Reyer Zwiggelaar',
}
update_long_names(423, names, True)

names = {
    'M. S.': 'Muhammad Sanaullah',
    'K. G.': 'Kaliappan Gopalan',
}
update_long_names(426, names, True)

names = {
    'L. S.': 'Lin Su',
    'M. D. L.': 'Martin D. Levine',
}
update_long_names(429, names, True)

names = {
    'M. J.': 'Mimansa Jaiswal',
    'S. T.': 'Sairam Tabibu',
    'R. B.': 'Rajiv Bajpai',
}
update_long_names(432, names, True)

names = {
    'Y. A.': 'Yaniv Azar',
    'M. C.': 'Matthew Campisi',
}
update_long_names(433, names, True)

names = {
    'N. H.': 'Naoki Hosomi',
    'S. S.': 'Sakriani Sakti',
    'K. Y.': 'Koichiro Yoshino',
    'S. N.': 'Satoshi Nakamura',
}
update_long_names(434, names, True)

names = {
    'M. A.': 'Mohamed Abouelenien',
    'V. P.': 'Verónica Pérez-Rosas',
    'R. M.': 'Rada Mihalcea',
    'M. B.': 'Mihai Burzo',
}
update_long_names(436, names, True)

names = {
    'V. G.': 'Viresh Gupta',
    'M. A.': 'Mohit Agarwal',
    'T. C.': 'Tanmoy Chakraborty',
    'R. S.': 'Richa Singh',
    'M. V.': 'Mayank Vatsa',
}
# Faltou o Manik Arora
update_long_names(439, names, True)

names = {
    'X. Y.': 'Xiang Yu',
    'S. Z.': ', Shaoting Zhang',
    'Z. Y.': 'Zhennan Yan',
    'F. Y.': 'Fei Yang',
    'J. H.': 'Junzhou Huang',
    'N. E. D.': 'Norah E. Dunbar',
    'M. L. J.': 'Matthew L. Jensen',
    'J. K. B.': 'Judee K. Burgoon',
    'D. N. M.': 'Dimitris N. Metaxas',
}
update_long_names(448, names, True)
```

```
names = {
    'M. D.': 'Mingyu Ding',
    'A. Z.': 'An Zhao',
    'Z. L.': 'Zhiwu Lu',
    'T. X.': 'Tao Xiang',
    'J. W.': 'Ji-Rong Wen',
}
update_long_names(451, names, True)

names = {
    'D. K.': 'Daniel Kopev',
    'A. A.': 'Ahmed Ali',
    'I. K.': 'Ivan Koychev',
    'P. N.': 'Preslav Nakov',
}
update_long_names(458, names, True)

names = {
    'Y. A.': 'Yaniv Azar',
    'M. C.': 'Matthew Campisi',
}
update_long_names(463, names, True)

names = {
    'H. H. T.': 'Harith H. Thannoon',
    'W. H. A.': 'Wissam H. Ali',
    'I. A. H.': 'Ivan A. Hashim',
}
update_long_names(467, names, True)

names = {
    'M. D.': 'Malcolm Dcosta',
    'D. S.': 'Dvijesh Shastri',
    'R. V.': 'Ricardo Vilalta',
    'J. K. B.': 'Judee K. Burgoon',
    'I. P.': 'Ioannis Pavlidis',
}
update_long_names(483, names, True)

names = {
    'S. T.': 'Shohei Takabatake',
    'K. S.': 'Kazutaka Shimada',
    'T. S.': 'Takeshi Saitoh',
}
update_long_names(493, names, True)

names = {
    'Z. L.': 'Zuhrah Labibah',
    'M. N.': 'Muhammad Nasrun',
    'C. S.': 'Casi Setianingsih',
}
update_long_names(501, names, True)

names = {
    'E. J. B.': 'Erica J. Briscoe',
    'D. S. A.': 'D. Scott Appling',
    'H. H.': 'Heather Hayes',
}
update_long_names(504, names, True)

names = {
    'D. B.': 'Dan Barsever',
    'S. S.': 'Sameer Singh',
    'E. N.': 'Emre Neftci',
}
update_long_names(527, names, True)

names = {
    'M. R.': 'Metod Rybar',
    'M. B.': 'Maria Bielikova',
}
update_long_names(546, names, True)
```

```
print('\nDONE!\n')
```

Finnish!!!
