# Supplementary material for: Deception detection with machine learning: A systematic review and statistical analysis
Source: PLoS One. 2023 Feb 9;18(2):e0281323. doi: 10.1371/journal.pone.0281323 (PMC9910662; doi:10.1371/journal.pone.0281323)
Supplement: S5 File — Source: The authors (2022). (PDF) [file pone.0281323.s005.pdf]

Deception Detection supported by Machine Learning

Literature Review - Corpus analysis

This notebook is devoted to performing some analysis on the document corpus rather their content, both before and after screening phases. The charts and tables found below render a bibliographic profile of the corpus.

Particularly, we explore the rejections after deep screening and present the reasons. We use **Pandas** and **MatPlotLib**.

Documents statistics

The following chars present general statistics on all the documents somehow related to this effort, not particularly related to the content of the studies, but with the literature review workflow.

Articles distribution per source database

Articles were retrieved from four different article databases:

1. ACM Digital Library
2. IEEE Xplore
3. Scopus
4. Web of Science

Here we can see what database most contributed to our initial base of articles, before the selection process.

Those article databases where chosen based on our previous experience on literature reviews on different topics.

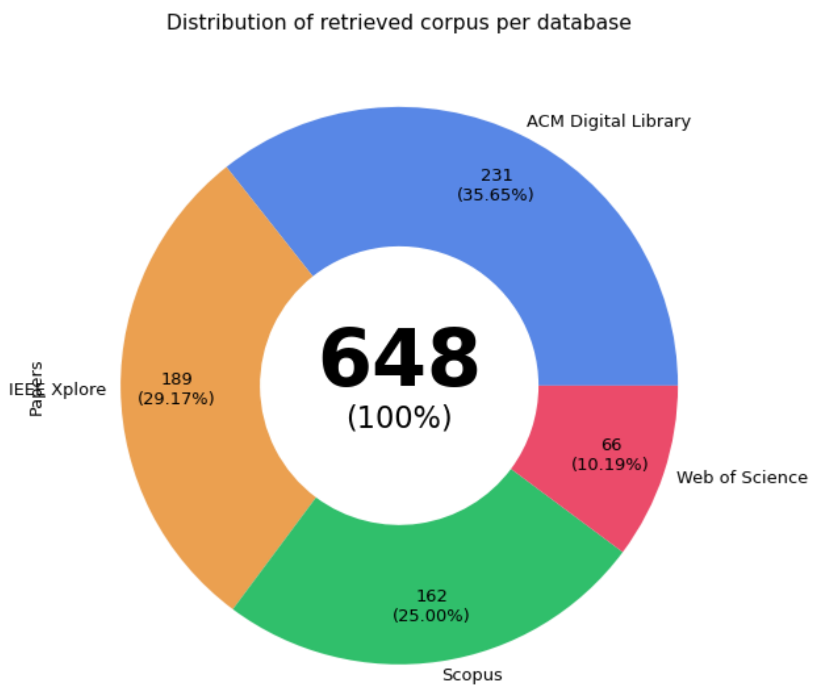

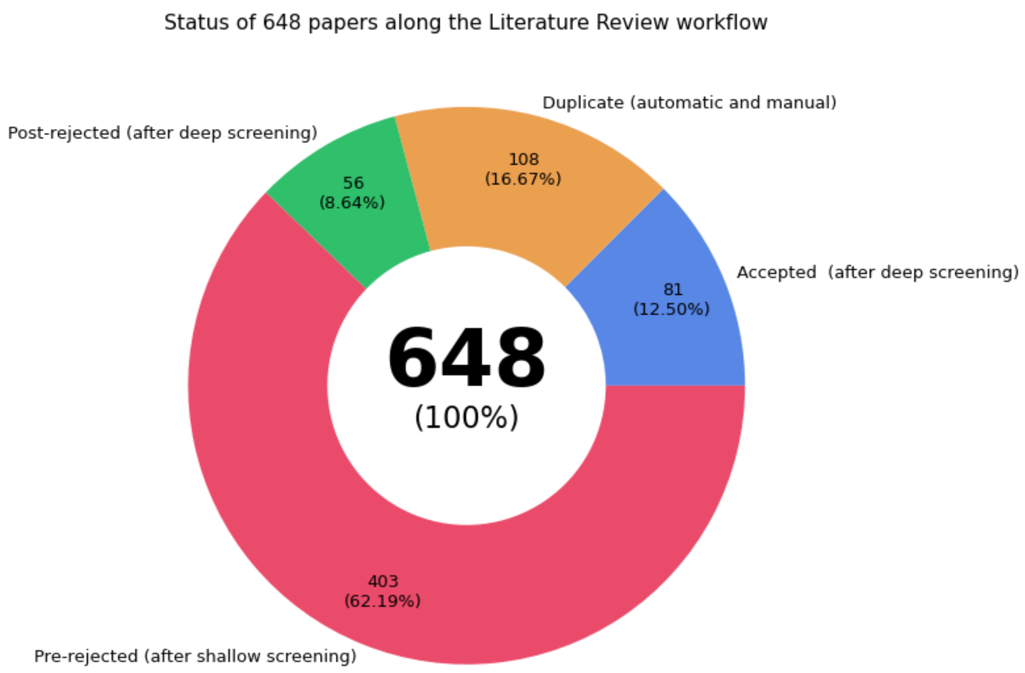

Rejection reasons

Papers were rejected by a number of different reasons (both before and after the deep screening) and their frequencies are reported below.

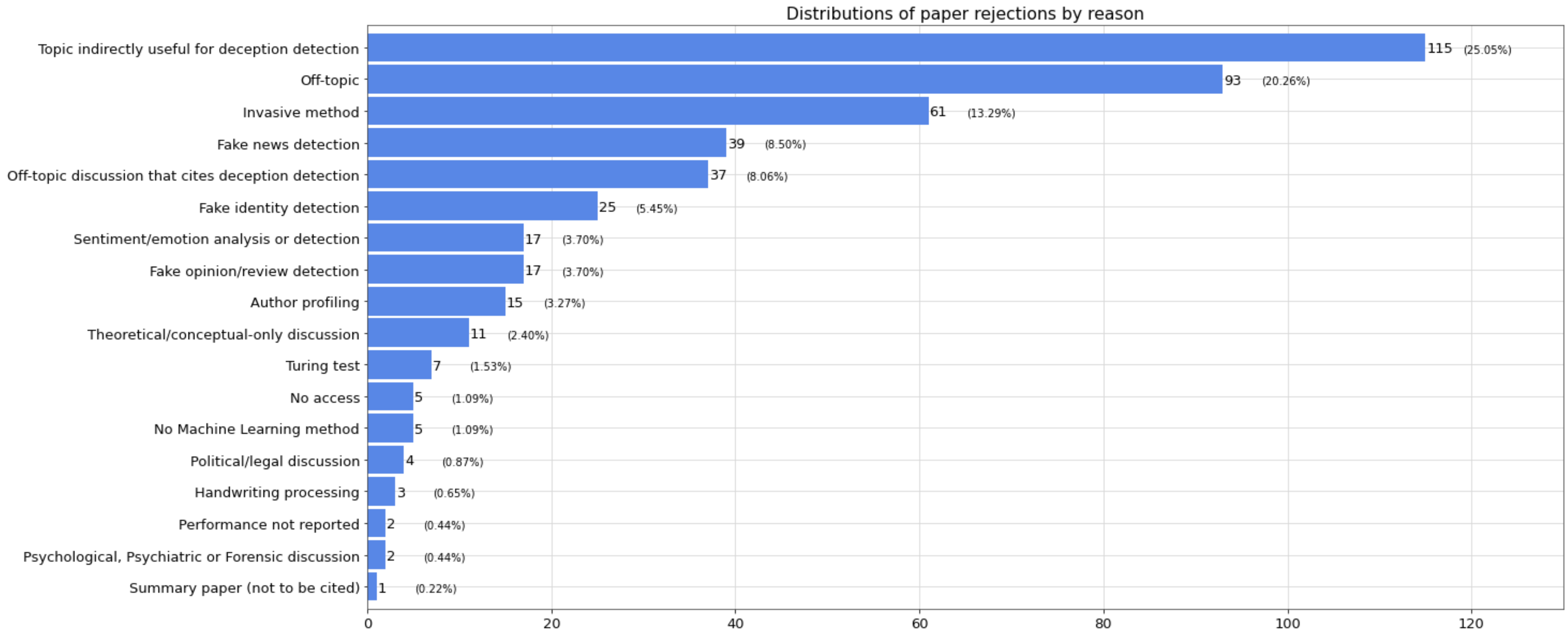

Document rejection reasons after deep screening

Shallow screening produced a corpus that was then deep screened (139 documents). After the full text reading some of those (56 documents) were rejected because they violated some of the selection criteria. Those are all listed below.

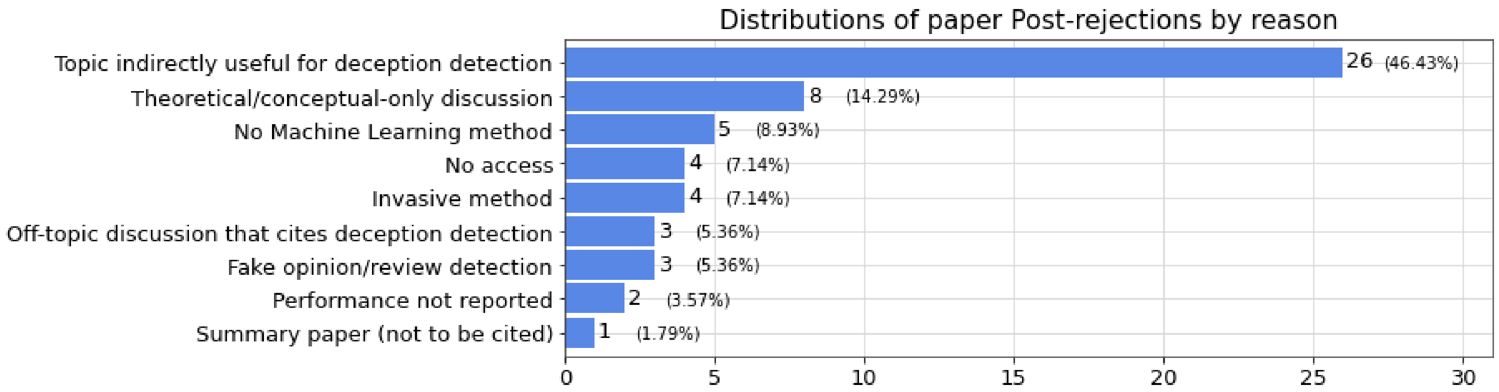

Selected corpus analysis

From this point on all analysis will be made only on the corpus of those papers that satisfy the selection criteria of the review.

Selected Articles distribution per source database

Here we show the source distribution of the selected articles only. While ACM Digital Library returned a large number of potential articles, IEEE Xplore holds the majority of the selected ones.

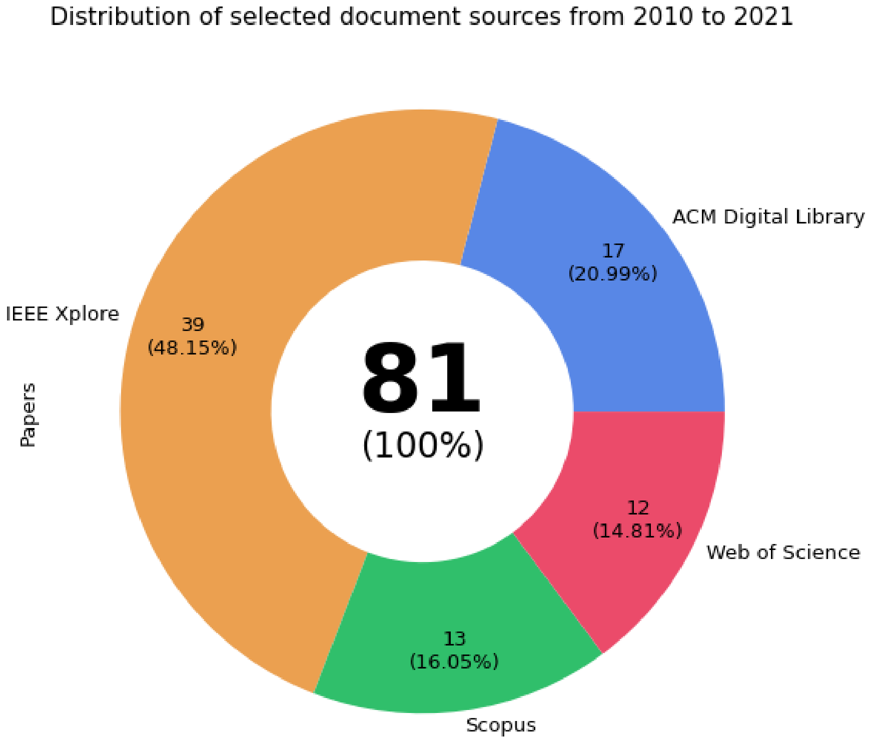

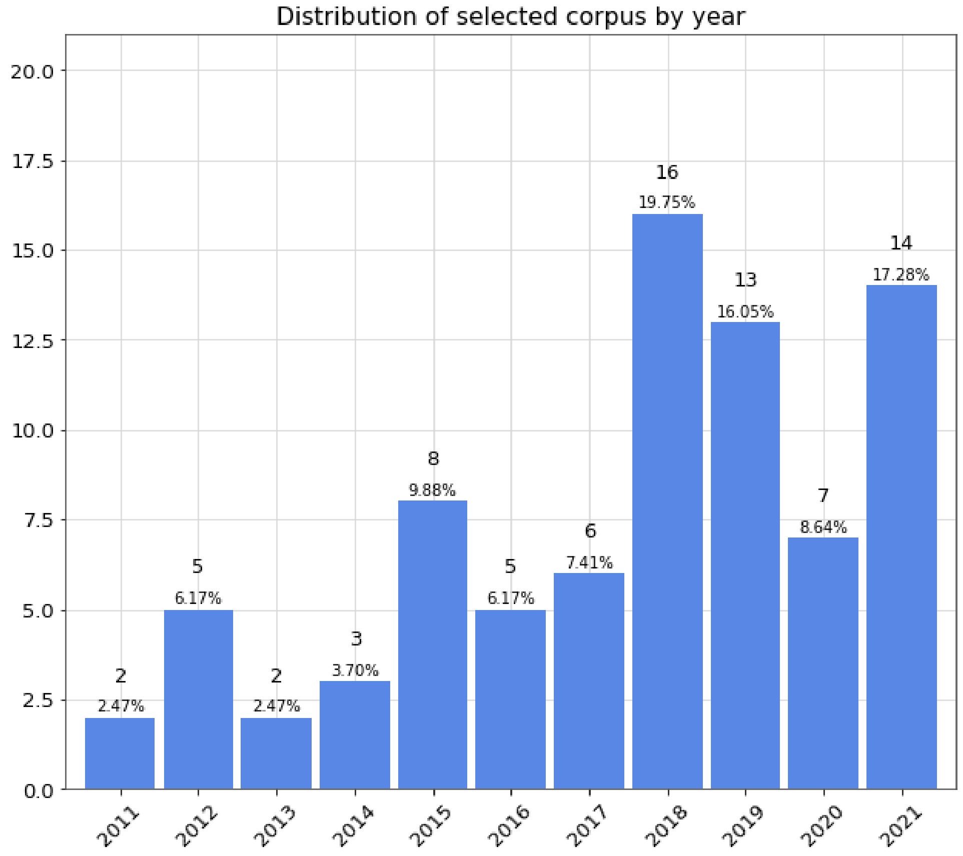

Papers per journal

Journal	Volume
Proceedings of the Workshop on Computational Approaches to Deception Detection	3
2019 IEEE/CVF Conference on Computer Vision and Pattern Recognition Workshops (CVPRW)	2
IEEE Access	2

IEEE Transactions on Information Forensics and Security	2
Proceedings of the 2015 ACM on International Conference on Multimodal Interaction	2
15th Conference of the European Chapter of the Association for Computational Linguistics, EACL 2017 - Proceedings of the Student Research Workshop	1
18TH ANNUAL CONFERENCE OF THE INTERNATIONAL SPEECH COMMUNICATION ASSOCIATION (INTERSPEECH 2017), VOLS 1-6: SITUATED INTERACTION	1
19TH ANNUAL CONFERENCE OF THE INTERNATIONAL SPEECH COMMUNICATION ASSOCIATION (INTERSPEECH 2018), VOLS 1-6: SPEECH RESEARCH FOR EMERGING MARKETS IN MULTILINGUAL SOCIETIES	1
2013 IEEE 56th International Midwest Symposium on Circuits and Systems (MWSCAS)	1
2014 47th Hawaii International Conference on System Sciences	1
2015 11th IEEE International Conference and Workshops on Automatic Face and Gesture Recognition (FG)	1
2015 IEEE International Conference on Intelligence and Security Informatics (ISI)	1
2015 SIXTH INTERNATIONAL CONFERENCE ON INTELLIGENT CONTROL AND INFORMATION PROCESSING (ICICIP)	1
2016 11th International Workshop on Semantic and Social Media Adaptation and Personalization (SMAP)	1
2016 IEEE 12th International Colloquium on Signal Processing Its Applications (CSPA)	1
2016 IEEE 16th International Conference on Data Mining Workshops (ICDMW)	1
2016 IEEE/ACS 13th International Conference of Computer Systems and Applications (AICCSA)	1
2017 20TH CONFERENCE OF THE ORIENTAL CHAPTER OF THE INTERNATIONAL COORDINATING COMMITTEE ON SPEECH DATABASES AND SPEECH I/O SYSTEMS AND ASSESSMENT (O-COCOSDA)	1
2017 IEEE SYMPOSIUM SERIES ON COMPUTATIONAL INTELLIGENCE (SSCI)	1
2018 Asia-Pacific Signal and Information Processing Association Annual Summit and Conference (APSIPA ASC)	1
2018 IEEE International Conference on Big Data (Big Data)	1
2018 IEEE International Conference on Internet of Things and Intelligence System (IOTAIS)	1
2018 International Joint Conference on Neural Networks (IJCNN)	1
2018 Joint 10th International Conference on Soft Computing and Intelligent Systems (SCIS) and 19th International Symposium on Advanced Intelligent Systems (ISIS)	1
2018 Second International Conference on Electronics, Communication and Aerospace Technology (ICECA)	1
2018 Third Scientific Conference of Electrical Engineering (SCEE)	1
2019 Asia-Pacific Signal and Information Processing Association Annual Summit and Conference (APSIPA ASC)	1
2019 IEEE 7th International Conference on Computer Science and Network Technology (ICCSNT)	1
2019 IEEE Automatic Speech Recognition and Understanding Workshop (ASRU)	1
2019 IEEE Conference on Multimedia Information Processing and Retrieval (MIPR)	1
2019 IEEE International Conference on Multimedia and Expo (ICME)	1
2019 IEEE/CVF Conference on Computer Vision and Pattern Recognition (CVPR)	1
2020 IEEE International Conference on Image Processing (ICIP)	1
2020 International Joint Conference on Neural Networks (IJCNN)	1
32nd AAAI Conference on Artificial Intelligence, AAAI 2018	1
57TH ANNUAL MEETING OF THE ASSOCIATION FOR COMPUTATIONAL LINGUISTICS (ACL 2019)	1
ACM International Conference Proceeding Series	1
ACM/IEEE International Conference on Human-Robot Interaction	1
ACTA PSYCHOLOGICA	1
APPLIED COGNITIVE PSYCHOLOGY	1
Companion Proceedings of the Web Conference 2020	1
Conference Proceedings - EMNLP 2015: Conference on Empirical Methods in Natural Language Processing	1
EXPERT SYSTEMS WITH APPLICATIONS	1

FRONTIERS IN ROBOTICS AND AI	1
First Monday	1
IEEE MultiMedia	1
IEEE Transactions on Affective Computing	1
IEEE Transactions on Cognitive and Developmental Systems	1
IEEE Transactions on Cybernetics	1
International Journal of Social Robotics	1
Komp'juternaja Lingvistika i Intellektual'nye Tehnologii	1
NAACL HLT 2018 - 2018 Conference of the North American Chapter of the Association for Computational Linguistics: Human Language Technologies - Proceedings of the Conference	1
Natural Language Engineering	1
PLoS ONE	1
PROCEEDINGS 2018 13TH IEEE INTERNATIONAL CONFERENCE ON AUTOMATIC FACE \& GESTURE RECOGNITION (FG 2018)	1
Proceedings of the 13th International Conference on Distributed Smart Cameras	1
Proceedings of the 13th International Conference on Multimodal Interfaces	1
Proceedings of the 15th ACM on International Conference on Multimodal Interaction	1
Proceedings of the 16th International Conference on Multimodal Interaction	1
Proceedings of the 2015 ACM on Workshop on Multimodal Deception Detection	1
Proceedings of the 2020 International Conference on Multimodal Interaction	1
Proceedings of the 20th ACM International Conference on Multimodal Interaction	1
Proceedings of the 2nd International Conference on Compute and Data Analysis	1
Proceedings of the 50th Annual Meeting of the Association for Computational Linguistics: Short Papers - Volume 2	1
Proceedings of the 9th ACM International Conference on PErvasive Technologies Related to Assistive Environments	1
Proceedings of the ASIST Annual Meeting	1
Proceedings of the International Joint Conference on Neural Networks	1
Proceedings of the Symposium on Applied Computing	1
SPEECH AND COMPUTER (SPECOM 2018)	1
