# Supplementary material for: Deception detection with machine learning: A systematic review and statistical analysis
Source: PLoS One. 2023 Feb 9;18(2):e0281323. doi: 10.1371/journal.pone.0281323 (PMC9910662; doi:10.1371/journal.pone.0281323)
Supplement: S6 File — Source: The authors (2022). (PDF) [file pone.0281323.s006.pdf]

Deception Detection supported by Machine Learning

Literature Review - Statistical analysis

This notebook is devoted to performing some statistical analysis on the selected corpus.

By statistical analysis we mean generating some tables and charts from the collected metadata on the documents selected by the Deep Screening (Step 7).

Metadata for each paper is encoded as a Python dictionary and saved in the BiblioAlly Catalog. All that was done in the **Step 9** of our research protocol (see the **2-Data Collection** notebook).

Those dictionaries are retrieved here and handled so data can be used to produce charts and tables.

We use **Pandas** and **MatPlotLib**.

1. Selected corpus summary

Below there is a list of all selected articles. Those are summarized in terms of **Technique**, **Performance**, **Features**, **Language**, and **Dataset**, since those give a general notion of each study. All those factors are directly related to the research question stated in notebook 1.

When a certain article explored more than one **Technique**, we present the one with the best performance. Some studies may present more than one **Performance** metric for each technique. In such a case we choose the metric with higher value to represent the performance.

1. **Features** describe what kind of data was consumed by the Machine Learning algorithm.
2. **Language** lists what languages were processed by the study, not in what language the study report is written (all are written in English).
3. **Dataset** tell us the name or its origin, and size in terms of instances.

Summary of all studies retrieved in the period of interest

	Year	Title	Technique	Dataset	Features	Languages
**72**	2021	Affect-Aware Deep Belief Network Representations for Multimodal Unsupervised Deception Detection [Mathur, Leena; Matarić, Maja J]	Neural Network / Precision: 0.88	Subset of Real-life Trial Deception Detection Dataset / 108 rows	Visual+Vocal / Eye gaze, Facial arousal, Facial expressions, Facial valence, Head pose, MFCC, Prosody, Spectral parameters, Voice energy, Voice quality	English
**78**	2021	Automatic Detection of Deceptive and Truthful Paralinguistic Information in Speech using Two-Level Machine Learning Model [Автоматическое определение ложной и истинной паралингвистической информации в речи человека с применением двухуровневой модели машинного обучения] [Velichko, A.N.; Karpov, A.A.]	Combined methods / F1-score: 0.856	Merging of Real-life Trial Deception Detection Dataset and Deceptive Speech Database / 1680 rows	Vocal / INTERSPEECH 2009, INTERSPEECH 2013, INTERSPEECH 2016	English
**74**	2021	Deception Detection and Remote Physiological Monitoring: A Dataset and Baseline Experimental Results [Speth, Jeremy; Vance, Nathan; Czajka, Adam; Bowyer, Kevin W.; Wright, Diane; Flynn, Patrick]	SVM / Accuracy: 0.626	Deception Detection and Physiological Monitoring Dataset / 1680 rows	Physiological+Visual / Eye saccades, Facial micro-expressions, Head pose, Heart rate, Thermal video	N/A

**80**	2021	Deception detection in text and its relation to the cultural dimension of individualism/collectivism [Papantoniou, K.; Papadakos, P.; Patkos, T.; Flouris, G.; Androutsopoulos, I.; Plexousakis, D.]	Logistic Regression / Recall: 0.93	Eleven multidomain and multicultural datasets. / 7024 rows	Demographical+Textual / BERT embeddings, Culture, Language, Lexical measures, N-grams, Phonemes, Pronouns, Relativity, Sentiment, Syntax complexity	English, Spanish, Romanian, Russian, Dutch
**76**	2021	Deception in the eyes of deceiver: A computer vision and machine learning based automated deception detection [Khan, Wasiq; Hussain, Abir; Crockett, Keeley; OShea, James; Khan, Bilal M.]	SVM / Precision: 0.84	Video recordings of true and false declarations on an interview taken from volunteers. / 255026 rows	Demographical+Visual / Ethnicity, Eye saccades, Facial micro-expressions, Gender	N/A
**79**	2021	Detecting Lies is a Child (Robot)'s Play: Gaze-Based Lie Detection in HRI [Gonzalez-Billandon, J.; Sciutti, A.; Sandini, G.; Rea, F.; Pasquali, D.; Aroyo, A.M.]	Random Forest / Area Under the Curve: 0.733	Pupil size measures taken during a card game with a iCub robot. / 37 rows	Visual / Pupil size	N/A
**69**	2021	Development of Spectral Speech Features for Deception Detection Using Neural Networks [Ullah, Muhammad S.; Fernandes, Sinead V.]	Neural Network / Accuracy: 0.9167	Three sessions of a police interrogation on a suspect. / 12 rows	Vocal / Bark	English
**77**	2021	How humans impair automated deception detection performance [Kleinberg, Bennett; Verschuere, Bruno]	Random Forest / Recall: 0.76	True and deceptive statements collected by a web application from volunteers. / 1640 rows	Textual / LIWC categories, POS tags	N/A
**68**	2021	Identity Unbiased Deception Detection by 2D-to-3D Face Reconstruction [Ngô, Lê Minh; Wang, Wei; Mandira, Burak; Karaoğlu, Sezer; Bouma, Henri; Dibeklioğlu, Hamdi; Gevers, Theo]	Neural Network / Recall: 0.72	Real-life Trial Deception Detection Dataset / 121 rows	Demographical+Visual / Age, Face image, Gender	N/A
**75**	2021	LieNet: A Deep Convolution Neural Networks Framework for Detecting Deception [Karnati, Mohan; Seal, Ayan; Yazidi, Anis; Krejcar, Ondrej]	Neural Network / Accuracy: 0.967375	Combination of three deception detection datasets / 766 rows	Physiological+Visual+Vocal / EEG channels, Face image, Voice signal	English
**73**	2021	Multimodal Political Deception Detection [Abouelenien, Mohamed; Hessler, Christian; Kamboj, Manvi; Asnani, Priyanka; Riani, Kais]	Decision Tree / Accuracy: 0.7	Videos with political debates with deceptions checked by PolitiFact.org / 180 rows	Textual+Visual+Vocal / Eye gaze, Facial emotion, Facial expressions, GloVe embeddings, Head pose, INTERSPEECH 2009, INTERSPEECH 2013, LIWC categories, POS tags, Sentiment, Unigrams	English
**67**	2021	Non-invasive Deception Detection in Videos Using Machine Learning Techniques [Islam, Siam; Saha, Popin; Chowdhury, Touhidul; Sorowar, Asif; Rab, Raqeebir]	SVM / Recall: 0.6972	Subset of features from Bag-of-lies / 325 rows	Visual / Facial expressions	N/A
**71**	2021	Unsupervised Audio-Visual Subspace Alignment for High-Stakes Deception Detection [Mathur, Leena; Matarić, Maja J.]	KNN / Area Under the Curve: 0.75	Subset of UR Lying Database / 107 rows	Visual+Vocal / Eye gaze, Facial expressions, Head pose, MFCC, Prosody, Statistical measures, Statistical measures, Voice quality, eGeMAPd	English
**70**	2021	Use of Machine Learning for Deception Detection From Spectral and Cepstral Features of Speech Signals [Ullah, Muhammad S.; Fernandes, Sinead V.]	Neural Network / Accuracy: 1.0	Three sessions of a police interrogation on a suspect. / 12 rows	Vocal / Delta cepstrum, Delta energy, Time difference cepstrum, Time difference energy	English
**10**	2020	Automated Deception Detection of Males and Females from Non-Verbal Facial Micro-Gestures [Crockett, K.; OShea, J.; Khan, W.]	Random Forest / Accuracy: 0.998	Interview video recordings / 86584 rows	Demographical+Visual / Eye gaze, Facial micro-gestures, Gender, Head pose	N/A
**65**	2020	Building a Better Lie Detector with BERT: The Difference Between Truth and Lies [Barsever, D.; Singh, S.; Neftci, E.]	Neural Network / Accuracy: 0.936	Ott Deceptive Opinion Spam Corpus / 1600 rows	Textual / BERT embeddings	English
**42**	2020	Emotion Transformation Feature: Novel Feature For Deception Detection In Videos [Yang, J. -T.; Liu, G. -M.; Huang, S. C. . -H.]	SVM / Accuracy: 0.8759	Superset of Real-life Trial Deception Detection Dataset / 190 rows	Emotional+Visual / Emotion Transformation, Eye gaze, Facial expressions, Hand motion	N/A
**26**	2020	Introducing Representations of Facial Affect in Automated Multimodal Deception Detection [Mathur, Leena; Mataric, Maja J.]	AdaBoost / Area Under the Curve: 0.91	Real-life Trial Deception Detection Dataset / 121 rows	Textual+Visual+Vocal / Eye gaze, Facial affect, Facial expressions, Head motion, LIWC	English

					categories, MFCC, Spectral parameters, Voice pitch, Voice quality	
**20**	2020	Multilingual Deception Detection by Autonomous Agents [Azaria, Amos; Hershkovitch Neiterman, Evgeny; Bitan, Moshe]	Neural Network / Accuracy: 0.6	Computer-based card game played by volunteers / 637 rows	Demographical+Vocal / Native language, Voice spectrogram	English, Hebrew
**43**	2020	Multimodal Deception Detection using Real-Life Trial Data [Mihalcea, R.; Abouelenien, M.; Burzo, M.; Sen, U. M.; Perez-Rosas, V.; Yanikoglu, B.]	Neural Network / Accuracy: 0.7288	Subset of Real-life Trial Deception Detection Dataset / 59 rows	Demographical+Textual+Visual+Vocal / Eye gaze, Facial expressions, Gender, Hand motion, Head motion, LIWC categories, Silence gaps, Unigrams, Voice histogram, Voice pitch	English
**11**	2020	Your eyes never lie: A robot magician can tell if you are lying [Gonzalez-Billandon, J.; Sciutti, A.; Sandini, G.; Rea, F.; Pasquali, D.; Aroyo, A.M.]	Random Forest / Area Under the Curve: 0.897	Card discovery game played by volunteers / 126 rows	Visual / Pupil dilation, Pupil dilation latency	N/A
**27**	2019	Automatic Deception Detection in RGB Videos Using Facial Action Units [Avola, Danilo; Cinque, Luigi; Foresti, Gian Luca; Pannone, Daniele]	SVM / Accuracy: 0.7684	Real-life Trial Deception Detection Dataset / 121 rows	Visual / Eye gaze, Facial expressions, Head motion	N/A
**49**	2019	Automatic Long-Term Deception Detection in Group Interaction Videos [Bai, C.; Wu, Z.; Singh, B.; Burgoon, J.; Bolonkin, M.; Chen, C.; Dunbar, N.; Subrahmanian, V. S.]	Combined methods / Area Under the Curve: 0.705	Online Resistance game / 285 rows	Visual+Vocal / Eye gaze, Facial expressions, Head motion, MFCC	N/A
**55**	2019	Bag-of-Lies: A Multimodal Dataset for Deception Detection [Gupta, V.; Agarwal, M.; Arora, M.; Chakraborty, T.; Singh, R.; Vatsa, M.]	Combined methods / Accuracy: 0.6617	Bag-of-lies / 325 rows	Physiological+Visual+Vocal / EEG channels, Eye blinks, Eye gaze, MFCC, Pupil size, Spectral parameters, Zero-crossing	N/A
**0**	2019	Can a Robot Catch You Lying? A Machine Learning System to Detect Lies During Interactions [Gonzalez-Billandon, Jonas; Aroyo, Alexander M.; Tonelli, Alessia; Pasquali, Dario; Sciutti, Alessandra; Gori, Monica; Sandini, Giulio; Rea, Francesco]	Random Forest / Area Under the Curve: 0.74	Questionnaire answered by volunteers / 1054 rows	Psychological+Visual / Eloquence, Eye blinks, Eye gaze, Histronic, NARS, NEO-FFI scores, Narcisistic Machiavellianism, Pupil dilation, Response time	N/A
**1**	2019	Detecting Concealed Information in Text and Speech [Hu, Shengli]	Neural Network / F1-score: 0.65615	Blind wine-tasting game / 12392 rows	Demographical+Textual+Vocal / Gender, GloVe embeddings, INTERSPEECH 2009, LIWC categories, N-grams, POS tags, Syntax complexity, Voice energy, Voice pitch, Voice quality	N/A
**58**	2019	Detecting Deception in Political Debates Using Acoustic and Textual Features [Kopev, D.; Ali, A.; Koychev, I.; Nakov, P.]	Neural Network / Accuracy: 0.5104	Political claims existing in the CT-FCC-18 corpus / 286 rows	Demographical+Textual+Vocal / BERT embeddings, Claim author, INTERSPEECH 2013, LIWC categories, N-grams, TF-IDF, i-vector features	English
**57**	2019	Face-Focused Cross-Stream Network for Deception Detection in Videos [Ding, M.; Zhao, A.; Lu, Z.; Xiang, T.; Wen, J.]	Neural Network / Area Under the Curve: 0.9978	Real-life Trial Deception Detection Dataset / 121 rows	Textual+Visual+Vocal / Facial expressions, Head motion, Spectral parameters, Unigrams	English
**45**	2019	High-Level Features for Multimodal Deception Detection in Videos [Rill-García, R.; Escalante, H. J.; Villaseñor-Pineda, L.; Reyes-Meza, V.]	BSSD / Area Under the Curve: 0.671	Real-life Trial Deception Detection Dataset / 121 rows	Textual+Visual+Vocal / Eye gaze, Facial expressions, Head pose, LIWC categories, MFCC, N-grams, POS tags, Syntax complexity, Voice pitch	English, Spanish
**12**	2019	How smart your smartphone is in lie detection? [Mizanur Rahman, Md.; Shome, A.; Chellappan, S.; Alim Al Islam, A.B.M.]	KNN / Precision: 0.95	Questionnaire answered by recruts / 121 rows	Emotional / Hand shaking	N/A
**13**	2019	Improved semi-supervised autoencoder for deception detection [Fu, H.; Lei, P.; Tao, H.; Zhao, L.; Yang, J.]	Neural Network / Accuracy: 0.6278	Video recordings of Werewolf online game / 987 rows	Vocal / INTERSPEECH 2009	Chinese
**47**	2019	Joint Learning of Conversational Temporal Dynamics and Acoustic Features for Speech Deception Detection in Dialog Games [Chou, H.; Liu, Y.; Lee, C.]	Neural Network / Unweighted Average Recall: 0.7471	Daily Deceptive Dialogues Corpus of Mandarin / 7504 rows	Dynamical+Vocal / Hesitation duration, MFCC, Silence count, Spectral parameters, Turn duration, Utterance duration, Voice energy, Voice pitch, Zero-crossing	Mandarin
**37**	2019	Robust Algorithm for Multimodal Deception Detection [Venkatesh, S.; Ramachandra, R.; Bours, P.]	Combined methods / Accuracy: 0.97	Real-life Trial Deception Detection Dataset / 121 rows	Textual+Visual+Vocal / Body motion, Facial micro-expressions, MFCC, N-grams	English
**38**	2019	Speech Deception Detection Algorithm Based on SVM and Acoustic Features [Fu, H.; Lei, P.; Tao, H.; Wang, M.; Wang, J.]	SVM / Accuracy: 0.8247	KWOLF / 388 rows	Vocal / MFCC, Voice energy, Voice pitch, Zero-crossing	Chinese

**5**	2018	A Multi-View Learning Approach To Deception Detection [Carissimi, Nicolo; Beyan, Cigdem; Murino, Vittorio]	Multi-view Learning / Accuracy: 0.98	Superset of Real-life Trial Deception Detection Dataset / 121 rows	Textual+Visual / Bigrams, Eye gaze, Facial expressions, Hand motion, Head motion, Unigrams	English
**4**	2018	Acoustic-Prosodic Indicators of Deception and Trust in Interview Dialogues [Levitan, Sarah Ita; Maredia, Angel; Hirschberg, Julia]	Random Forest / Precision: 0.7837000000000001	Columbia X-Cultural Dataset / 49106 rows	Demographical+Vocal / Gender, Native language, Voice energy, Voice pitch, Voice quality	English, Mandarin
**36**	2018	An Empirical Study on Detecting Deception and Cybercrime Using Artificial Neural Networks [Mbaziira, Alex V.; Murphy, Diane R.]	Neural Network / Area Under the Curve: 0.7999999999999999	Combination of four publicly available dataset / 300 rows	Textual / Syntax complexity	English
**2**	2018	Automated verbal credibility assessment of intentions: The model statement technique and predictive modeling [Kleinberg, Bennett; van der Toolen, Yaloe; Vrij, Aldert; Arntz, Arnoud; Verschuere, Bruno]	SVM / Accuracy: 0.7742	Interviews on weekand plans collected from volunteers / 147 rows	Textual / LIWC categories, Named entities, Psychological processes	English
**3**	2018	Comparative Analysis of Classification Methods for Automatic Deception Detection in Speech [Velichko, Alena; Budkov, Viktor; Kagirov, Ildar; Karpov, Alexey]	Decision Tree / Unweighted Average Recall: 0.795	Superset of Real-life Trial Deception Detection Dataset / 195 rows	Vocal / INTERSPEECH 2013	English
**62**	2018	Construction of a Liar Corpus and Detection of Lying Situations [Takabatake, S.; Shimada, K.; Saitoh, T.]	SVM / Accuracy: 0.5516	Computer-based question-answer interview / 540 rows	Visual / Facial micro-expressions	N/A
**48**	2018	Convolutional Bidirectional Long Short-Term Memory for Deception Detection With Acoustic Features [Tao, H.; Zhao, L.; Xie, Y.; Liang, R.; Zhu, Y.]	Neural Network / Accuracy: 0.7487	Question-answer experiment answered by selected participants / 7867 rows	Vocal / Spectral parameters, Voice pitch	Chinese
**53**	2018	Deception Detection and Analysis in Spoken Dialogues based on FastText [Hosomi, N.; Sakti, S.; Yoshino, K.; Nakamura, S.]	Neural Network / Precision: 0.667	CSC Deceptive Speech / 4100 rows	Textual+Vocal / FastText embedding, INTERSPEECH 2009	English
**14**	2018	Deception detection in videos [Subrahmanian, V.S.; Wu, Z.; Singh, B.; Davis, L.S.]	Logistic Regression / Area Under the Curve: 0.9221	Subset of Real-life Trial Deception Detection Dataset / 104 rows	Textual+Visual+Vocal / Facial micro-expressions, GloVe embeddings, MFCC	English
**40**	2018	Deception detection using artificial neural network and support vector machine [Srivastava, N.; Dubey, S.]	SVM / Accuracy: 1.0	15-question questionnaire answered by 50 people / 750 rows	Physiological+Vocal / Blood pressure, Heart rate, MFCC, Respiration rate, Voice energy, Voice pitch, Zero-crossing	N/A
**59**	2018	Detection of Deception Using Facial Expressions Based on Different Classification Algorithms [Thannoon, H. H.; Ali, W. H.; Hashim, I. A.]	Neural Network / Accuracy: 0.84	Questionnaire answered by volunteers / 448 rows	Demographical+Visual / Facial expressions, Gender	N/A
**44**	2018	Intelligent Deception Detection through Machine Based Interviewing [Crockett, K.; Khan, W.; O'Shea, J.; Kindynis, P.; Antoniades, A.; Boultadakis, G.]	Neural Network / Accuracy: 0.74605	Questionnaire answered by volunteers / 400 rows	Demographical+Visual / Ethnicity, Eye gaze, Facial expressions, Gender, Head pose	N/A
**22**	2018	Interpretable Multimodal Deception Detection in Videos [Karimi, Hamid]	Neural Network / Accuracy: 0.8416	Real-life Trial Deception Detection Dataset / 121 rows	Visual+Vocal / Facial expressions, INTERSPEECH 2009, INTERSPEECH 2013	English
**63**	2018	Lie Detector With The Analysis Of The Change Of Diameter Pupil and The Eye Movement Use Method Gabor Wavelet Transform and Decision Tree [Labibah, Z.; Nasrun, M.; Setianingsih, C.]	Decision Tree / Precision: 0.97	Questionnaire answered by volunteers / 40 rows	Visual / Eye gaze, Pupil dilation	N/A
**15**	2018	Linguistic cues to deception and perceived deception in interview dialogues [Levitan, S.I.; Maredia, A.; Hirschberg, J.]	Random Forest / Precision: 0.71685	Columbia X-Cultural Dataset / 4056 rows	Demographical+Psychological+Textual / Gender, LIWC categories, Lexical measures, NEO-FFI scores, Native language, Pauses, Syntax complexity	English
**46**	2018	Toward End-to-End Deception Detection in Videos [Karimi, H.; Tang, J.; Li, Y.]	KNN / Accuracy: 0.8416	Real-life Trial Deception Detection Dataset / 121 rows	Visual+Vocal / Facial expressions, INTERSPEECH 2009, INTERSPEECH 2013	English

**8**	2017	Construction and Analysis of Indonesian-Interviews Deception Corpus [Warnita, Tifani; Lestari, Dessi Puji]	Random Forest / F1-score: 0.613	Indonesian Deception Corpus / 5542 rows	Textual+Vocal / INTERSPEECH 2010, LIWC categories, Silence gaps, Voice energy, Voice pitch	Indonesian
**16**	2017	Deception detection in Russian texts [Litvinova, O.; Litvinova, T.; Seredin, P.; Lyell, J.]	Clustering / Accuracy: 0.683	Russian Deception Bank / 226 rows	Demographical+Textual / Age, Education, Gender, LIWC categories, POS tags	Russian
**6**	2017	Deep Learning Driven Multimodal Fusion For Automated Deception Detection [Gogate, Mandar; Adeel, Ahsan; Hussain, Amir]	Neural Network / Accuracy: 0.964	Superset of Real-life Trial Deception Detection Dataset / 121 rows	Textual+Visual+Vocal / Facial expressions, GloVe embeddings, Hand motion, INTERSPEECH 2013	English
**54**	2017	Detecting Deceptive Behavior via Integration of Discriminative Features From Multiple Modalities [Pérez-Rosas, V.; Mihalcea, R.; Abouelenien, M.; Burzo, M.]	Decision Tree / Accuracy: 0.8926	Mock crime game and a questionnaire about two sensitive themes answered by 30 volunteers / 149 rows	Physiological+Textual+Thermal / Blood volume, Cheeks, Forehead, Heart rate, LIWC categories, Nose, POS tags, Periorbital region, Respiration volume, Skin conductance, Syntax complexity, TF-IDF	English
**21**	2017	Gender-Based Multimodal Deception Detection [Perez-Rosas, Veronica; Mihalcea, Rada; Abouelenien, Mohamed; Zhao, Bohan; Burzo, Mihai]	Decision Tree / Accuracy: 0.664	Mock crime game and a questionnaire about two sensitive themes answered by 104 volunteers / 520 rows	Demographical+Physiological+Textual+Thermal / Face region, Gender, Heart rate, LIWC categories, POS tags, Respiration rate, Skin conductance, Syntax complexity, Unigrams	English
**7**	2017	Hybrid Acoustic-Lexical Deep Learning Approach for Deception Detection [Levitan, Sarah Ita; Hirschberg, Julia; Mendels, Gideon; Lee, Kai-Zhan]	Neural Network / F1-score: 0.639	Columbia X-Cultural Dataset / 49106 rows	Textual+Vocal / GloVe embeddings, INTERSPEECH 2009, INTERSPEECH 2013, MFCC, N-grams	English
**28**	2016	Analyzing Thermal and Visual Clues of Deception for a Non-Contact Deception Detection Approach [Mihalcea, Rada; Abouelenien, Mohamed; Burzo, Mihai]	Decision Tree / Accuracy: 0.6174	A trivia game answered by volunteers / 149 rows	Thermal+Visual / Entire face, Eye blinks, Facial expressions, Head motion	N/A
**66**	2016	Automated detection of user deception in on-line questionnaires with focus on eye tracking use [Rybar, M.; Bielikova, M.]	SVM / Precision: 0.64	Gaze and pupil data collected during questionnaire answering by volunteers / 50 rows	Visual / Eye saccades, Pupil dilation, Response time	N/A
**60**	2016	Deceptive Speech Detection based on sparse representation [Fan, Cheng; Zhao, Heming; Chen, Xueqin; Fan, Xiaohe; Chen, Shuxi]	SVM / Accuracy: 0.7295	Soochow Deceptive Speech Detection Corpus / 4143 rows	Demographical+Vocal / Gender, MFCC, Spectral parameters, Zero-crossing	Chinese
**41**	2016	ReLiDSS: Novel lie detection system from speech signal [Nasri, H.; Ouarda, W.; Alimi, A. M.]	SVM / Accuracy: 0.86375	ReliDDB / 137640 rows	Vocal / MFCC, Voice pitch	N/A
**52**	2016	The Truth and Nothing But the Truth: Multimodal Analysis for Deception Detection [Jaiswal, M.; Tabibu, S.; Bajpai, R.]	SVM / Accuracy: 0.7895	Subset of Real-life Trial Deception Detection Dataset / 100 rows	Textual+Visual+Vocal / Facial expressions, MFCC, POS tags, Prosody, Sentiment, Unigrams, Voice energy	English
**39**	2015	A comparison of features for automatic deception detection in synchronous computer-mediated communication [Pak, J.; Zhou, L.]	Decision Tree / Accuracy: 0.98	Communications during sessions of the online mafia game / 142 rows	Textual / LIWC categories, Syntax complexity, Unigrams	English
**29**	2015	Cross-Cultural Production and Detection of Deception from Speech [Hirschberg, Julia; Mendels, Gideon; Levitan, Sarah I.; An, Guzhen; Wang, Mandi; Levine, Michelle; Rosenberg, Andrew]	Random Forest / Accuracy: 0.6589	Statements provided by volunteers in a mock fake resume game / 154 rows	Demographical+Psychological+Vocal / Ethnicity, Gender, NEO-FFI scores, Native language, Speaking rate, Voice energy, Voice pitch, Voice quality	English, Mandarin
**24**	2015	Deception Detection Using Real-Life Trial Data [Perez-Rosas, Veronica; Mihalcea, Rada; Abouelenien, Mohamed; Burzo, Mihai]	Random Forest / Accuracy: 0.7355	Real-life Trial Deception Detection Dataset / 121 rows	Textual+Visual / Bigrams, Body motion, Facial expressions, Head motion, Unigrams	English
**35**	2015	Detection of Deception in the Mafia Party Game [Demyanov, Sergey; Bailey, James; Ramamohanarao, Kotagiri; Leckie, Christopher]	Logistic Regression / Accuracy: 0.7026	Mafia DB / 6733 rows	Visual / Facial expressions	N/A
**9**	2015	Distinguishing Deception from Non-Deception in Chinese Speech [Fan, Cheng; Zhao, Heming; Chen, Xueqin; Fan, Xiaohe; Chen, Shuxi]	Decision Tree / Recall: 0.83555	Soochow Deceptive Speech Detection Corpus / 3787 rows	Demographical+Vocal / Duration, Formant, Gender, Voice energy, Voice pitch	Mandarin

**17**	2015	Experiments in open domain deception detection [Pérez-Rosas, V.; Mihalcea, R.]	SVM / Accuracy: 0.695	Open domain sentences collected from volunteers / 7168 rows	Demographical+Textual / Age, Education, Gender, LIWC categories, Nationality, POS tags, Syntax complexity, Unigrams	English
**56**	2015	Is Interactional Dissynchrony a Clue to Deception? Insights From Automated Analysis of Nonverbal Visual Cues [Burgoon, J. K.; Yu, X.; Zhang, S.; Yan, Z.; Yang, F.; Huang, J.; Dunbar, N. E.; Jensen, M. L.; Metaxas, D. N.]	SVM / Precision: 0.668	From a trivia game with volunteers / 100 rows	Visual / Facial expressions, Head motion, Interactional synchrony	N/A
**61**	2015	Perinasal indicators of deceptive behavior [Burgoon, J. K.; Dcosta, M.; Shastri, D.; Vilalta, R.; Pavlidis, I.]	Neural Network / Accuracy: 0.8	From a mock crime game answered by volunteers / 40 rows	Thermal / Perinasal region	N/A
**64**	2014	Cues to Deception in Social Media Communications [Briscoe, E. J.; Appling, D. S.; Hayes, H.]	Gradient Boosting / Accuracy: 0.91	Statements provided by volunteers in a mock chat room / 254 rows	Textual / Emoticons, Informality, Sentiment, Syntax complexity	English
**23**	2014	Deception Detection Using a Multimodal Approach [Perez-Rosas, Veronica; Mihalcea, Rada; Abouelenien, Mohamed; Burzo, Mihai]	Decision Tree / Accuracy: 0.701	Statements from volunteers in an opinion game / 120 rows	Physiological+Textual+Thermal / Blood volume, Entire face, LIWC categories, Respiration volume, Skin conductance, Unigrams	English
**50**	2014	Thermal Facial Analysis for Deception Detection [Rajoub, B. A.; Zwiggelaar, R.]	KNN / Accuracy: 0.8688	Statements from volunteers in an interview game / 492 rows	Thermal / Periorbital region	N/A
**32**	2013	Automatic Detection of Deceit in Verbal Communication [Perez-Rosas, Veronica; Mihalcea, Rada; Burzo, Mihai]	SVM / Accuracy: 0.737	Video recordings from volunteers / 140 rows	Textual / Unigrams	English
**51**	2013	Deception detection in speech using bark band and perceptually significant energy features [Sanaullah, M.; Gopalan, K.]	Neural Network / Accuracy: 0.8333	Utterances from recordings of police interrogations / 6 rows	Vocal / Bark, Significant energy	English
**18**	2012	Discerning truth from deception: Human judgments and automation efforts [Rubin, V.L.; Conroy, N.]	Decision Tree / Accuracy: 0.65	Stories written by volunteers / 90 rows	Textual / LIWC categories, Lexical measures	English
**33**	2012	On the Use of Homogenous Sets of Subjects in Deceptive Language Analysis [Fornaciari, Tommaso; Poesio, Massimo]	SVM / Precision: 0.7185	DeCour corpus / 3015 rows	Textual / LIWC categories, Lexical measures, N-grams, POS tags	Italian
**31**	2012	Seeing through Deception: A Computational Approach to Deceit Detection in Written Communication [Almela, Angela; Valencia-Garcia, Rafael; Cantos, Pascual]	SVM / F1-score: 0.702	Opinions from volunteers on three themes / 600 rows	Textual / LIWC categories	Spanish
**25**	2012	Syntactic Stylometry for Deception Detection [Feng, Song; Banerjee, Ritwik; Choi, Yejin]	SVM / Accuracy: 0.912	Reviews of 35 Italian restaurants / 2692 rows	Textual / Bigrams, POS tags, Syntax complexity, Unigrams	English
**30**	2012	The Voice and Eye Gaze Behavior of an Imposter: Automated Interviewing and Detection for Rapid Screening at the Border [Elkins, Aaron C.; Derrick, Douglas C.; Gariup, Monica]	Decision Tree / Accuracy: 0.9447	Participants of an experiment in UE border control / 259 rows	Thermal+Vocal / Eye gaze, Pupil dilation, Voice energy, Voice pitch, Voice quality	N/A
**19**	2011	Challenges in automated deception detection in computer-mediated communication [Conroy, N.J.; Rubin, V.L.]	SMO / Accuracy: 0.65	Stories written by volunteers / 90 rows	Textual / LIWC categories, Lexical measures	English
**34**	2011	Move, and i Will Tell You Who You Are: Detecting Deceptive Roles in Low-Quality Data [Raiman, Nimrod; Hung, Hayley; Englebienne, Gwenn]	SVM / F1-score: 0.76	Wolf-database / 72 rows	Visual+Vocal / Body motion, Non-silent	N/A

2. Language analysis

The following charts present a summarization on the **operated language** in papers. The operated language is not the language which the paper is written. Rather, it is the language of the textual and vocal modality features explored in studies present in the selected corpus.

This theme is important because it divides the studies in two distinctive groups: one based on English and the other based on other languages. Naturally, verbal cues are greatly dependent to language aspects, so most of the knowledge found on English-based studies need to be adapted or tested on other languages.

We can see this theme in two forms:

1. A general distribution of operated languages;
2. A distribution of operated languages by years in the period of interest.

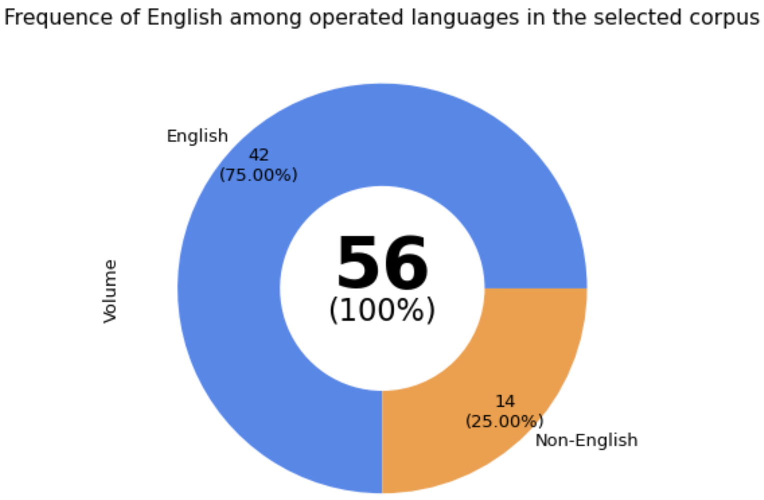

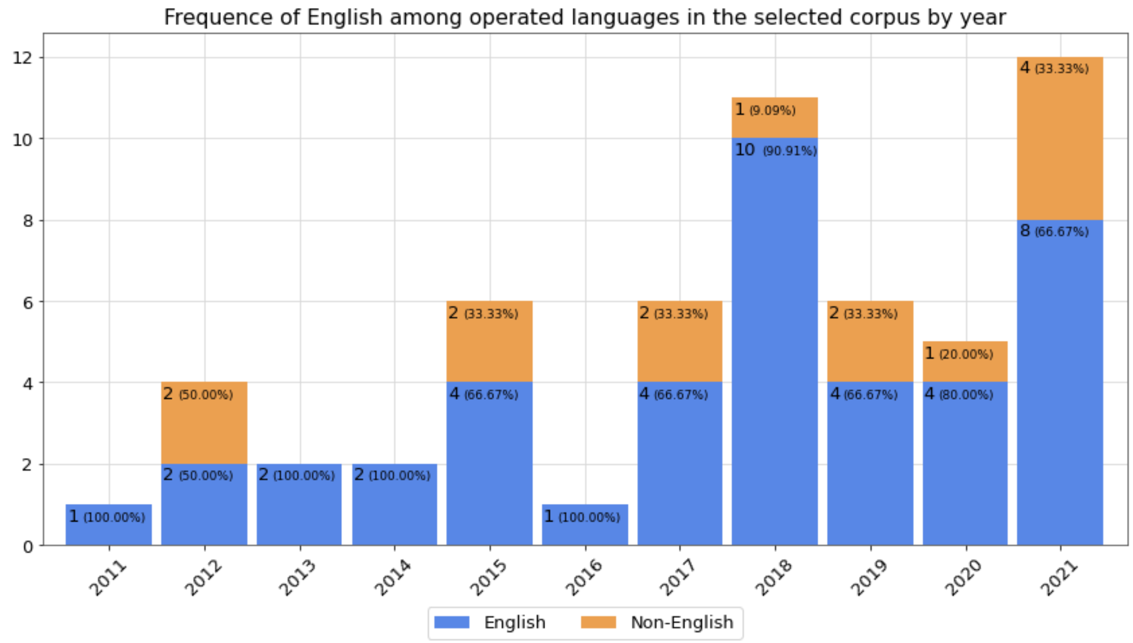

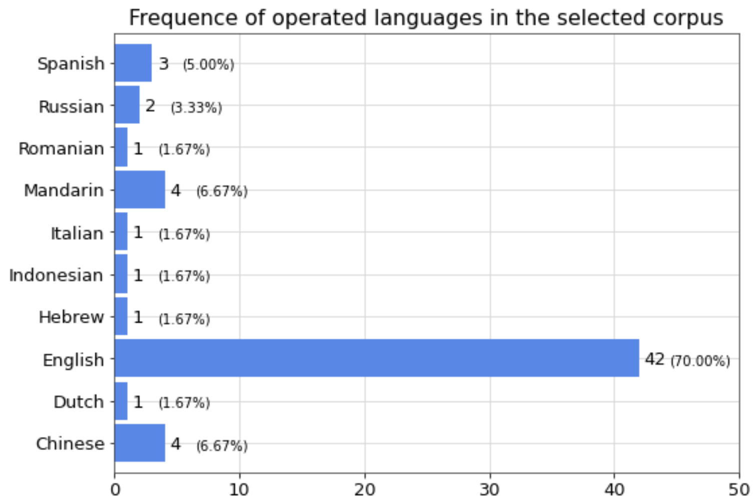

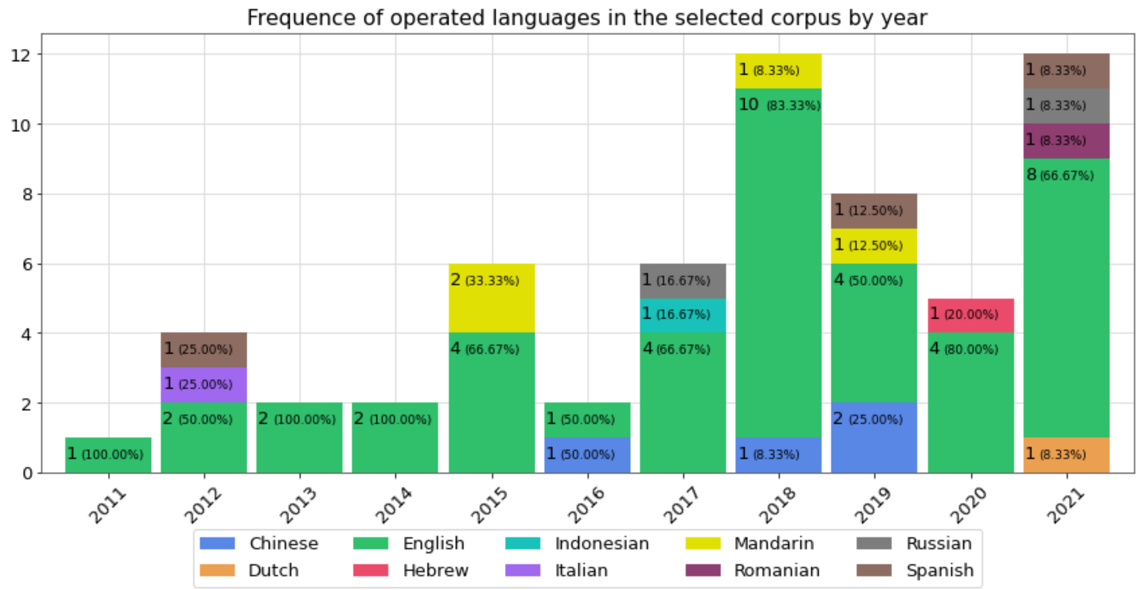

3. Modality analysis

The following charts present the distribution of modality kinds, modality cardinalities and modality combinations.

Modality kind describes the information nature (or source of data) exploited for feature extraction (textual, visual, vocal, demographic, and so on).

Modality cardinality is the number of modality kinds combined in studies and can be one of the following:

1. **Monomodal**: only one modality is used, for instance, vocal features;
2. **Bimodal**: the combination of two modalities;
3. **Multimodal**: the combination of three or more modalities.

3.1. Feature modality analysis

Different modalities were exploited all over the period of interest. This section shows which ones and when they were studies.

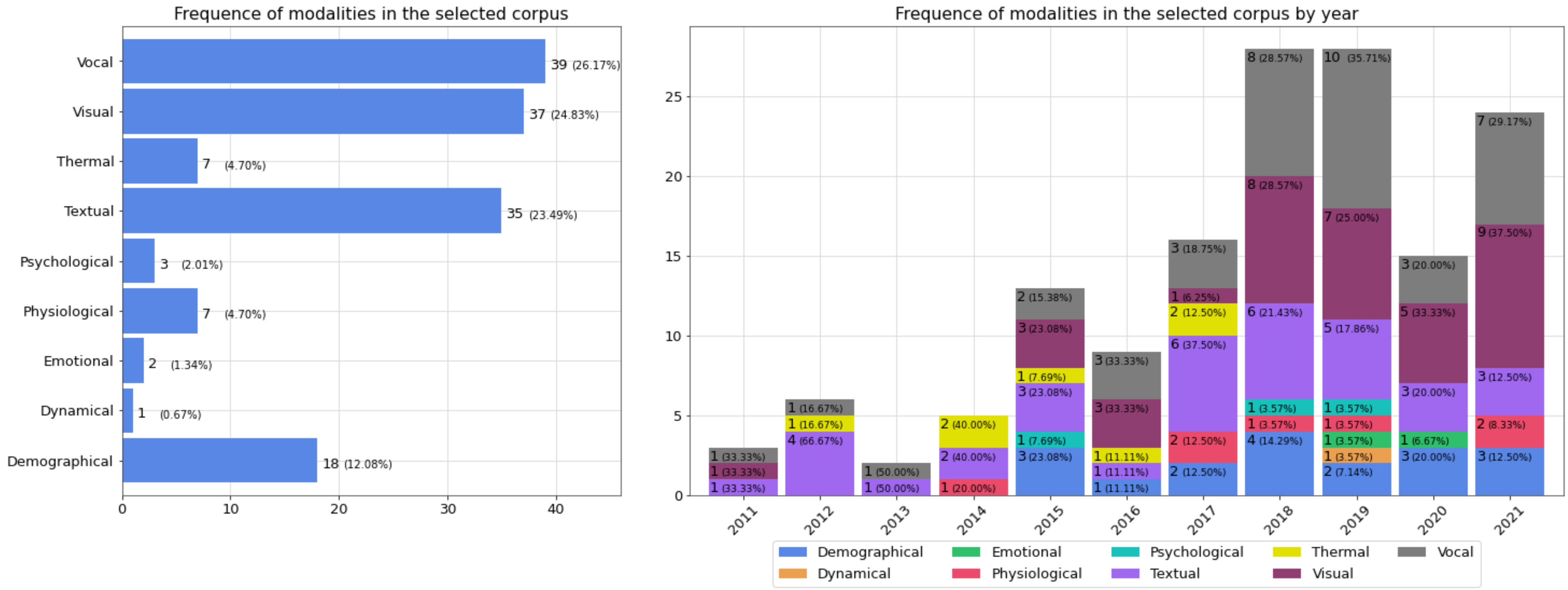

3.2. Modality cardinality analysis

Different modalities were exploited alone (monomodal approach) or in combination with other modalities (bimodal and multimodal approaches). This section shows how modality cardinalities are distributed in the period of interest.

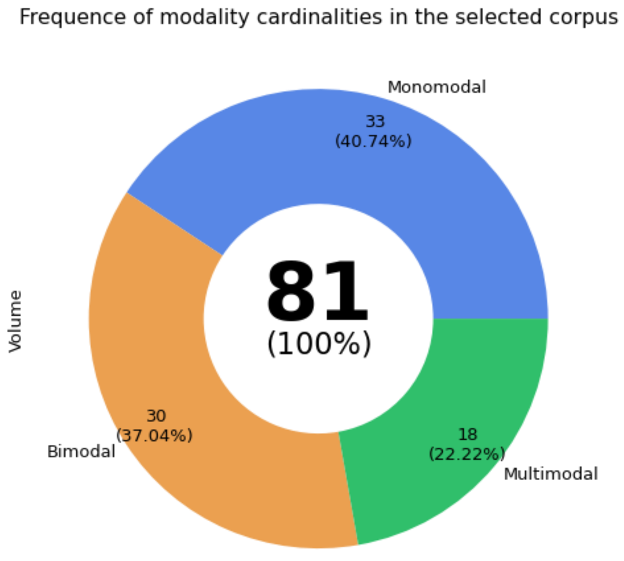

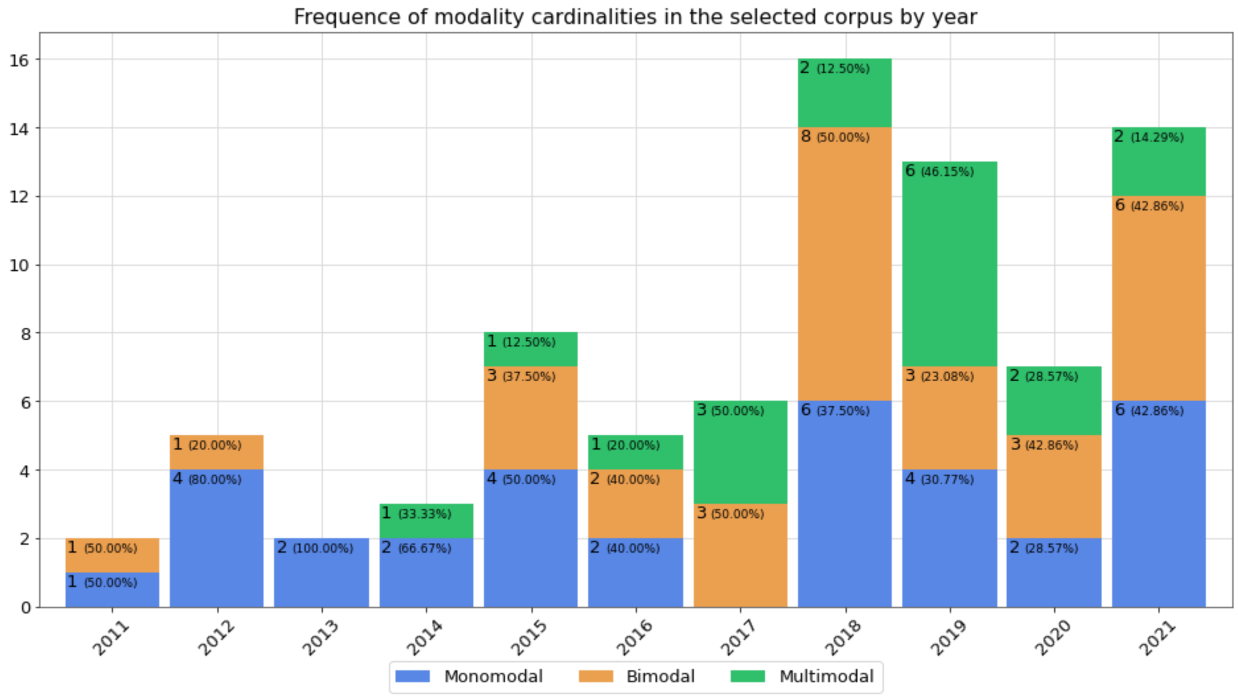

3.3. Monomodal studies analysis

Monomodal approach still poses as the most frequent. However, there are different modalities in monomodal studies. This section presents how those modalities are distributed in the period of interest for monomodal studies.

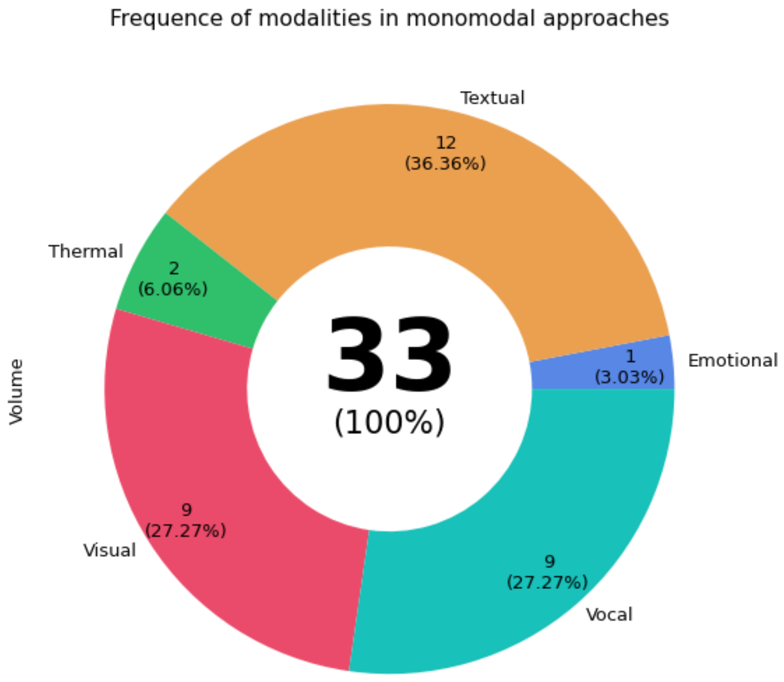

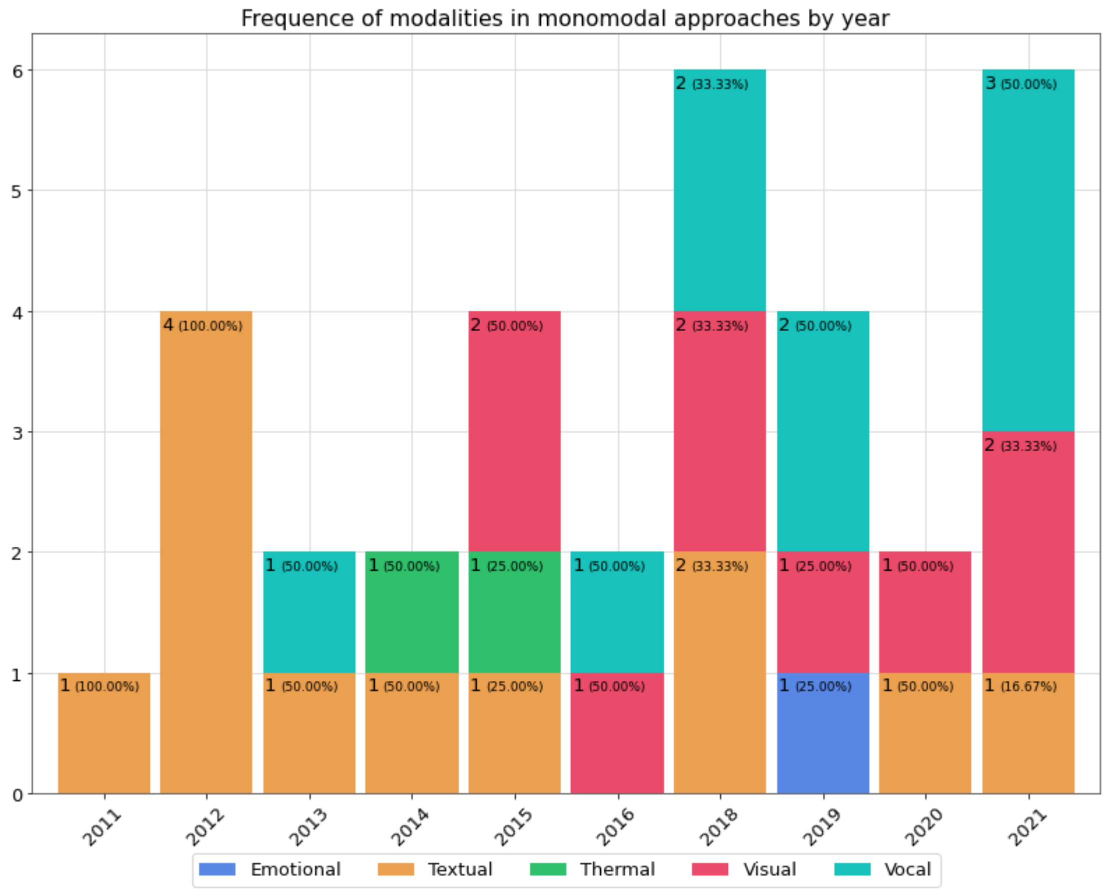

3.4. Bimodal studies analysis

Bimodal approach takes the second position in terms of preference. There are different distributions of modalities combinations in bimodal studies. This section presents how those modalities are combined and distributed in the period of interest for bimodal studies.

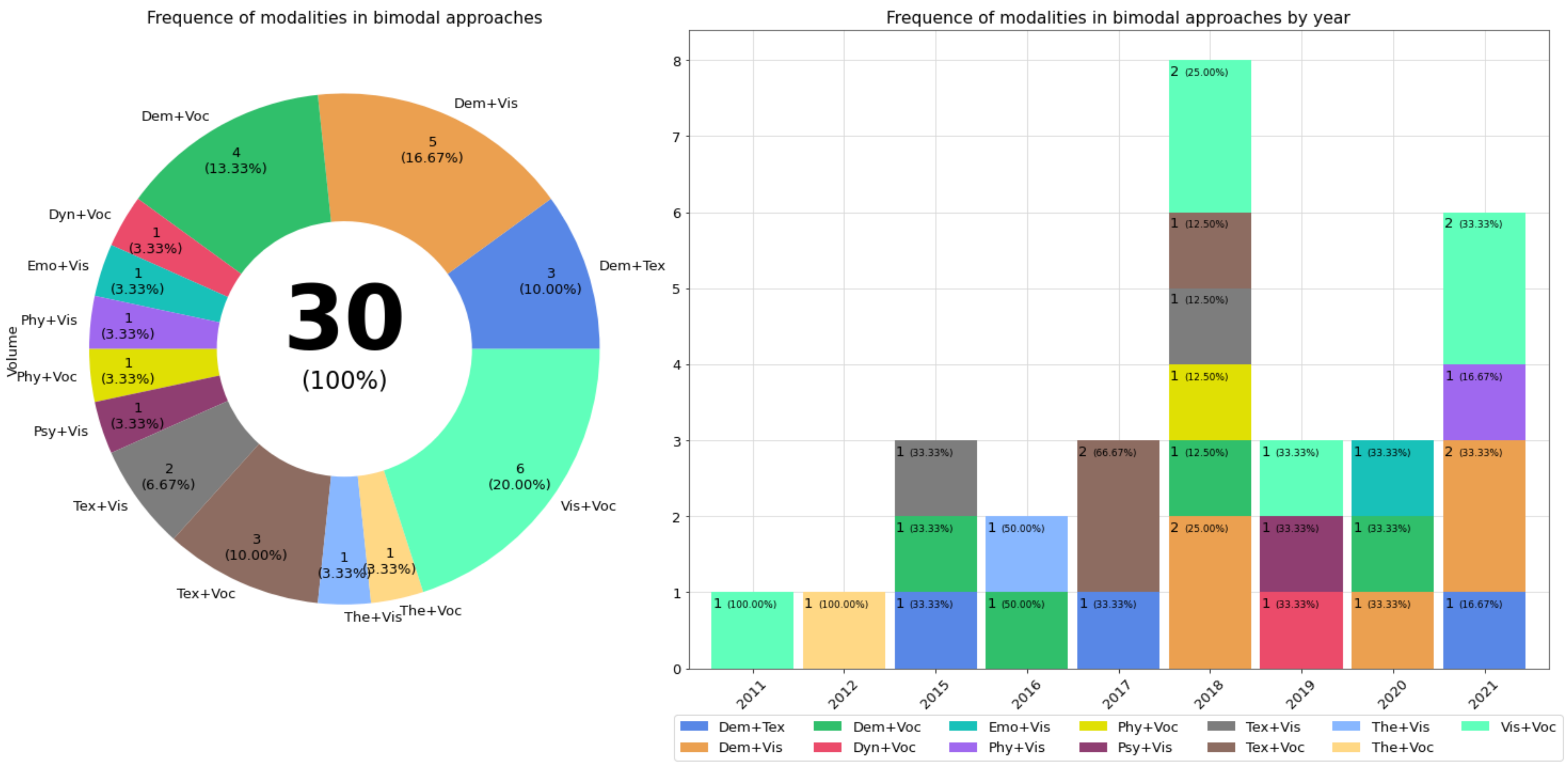

3.5. Multimodal studies analysis

Multimodal approach is least preferred one among studies. However, there are different distributions of modalities combinations in multimodal studies. This section presents how those modalities are combined and distributed in the period of interest for multimodal studies.

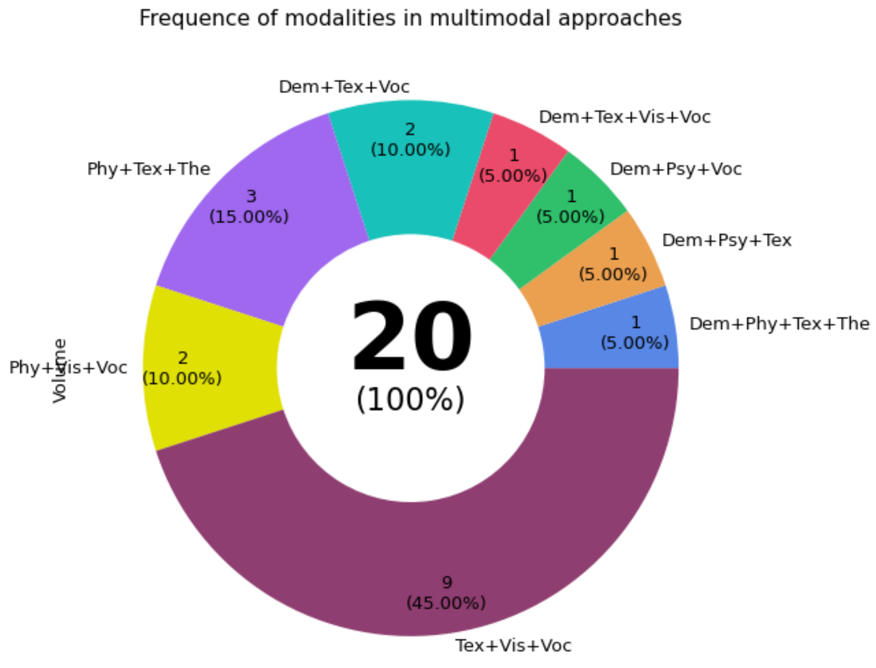

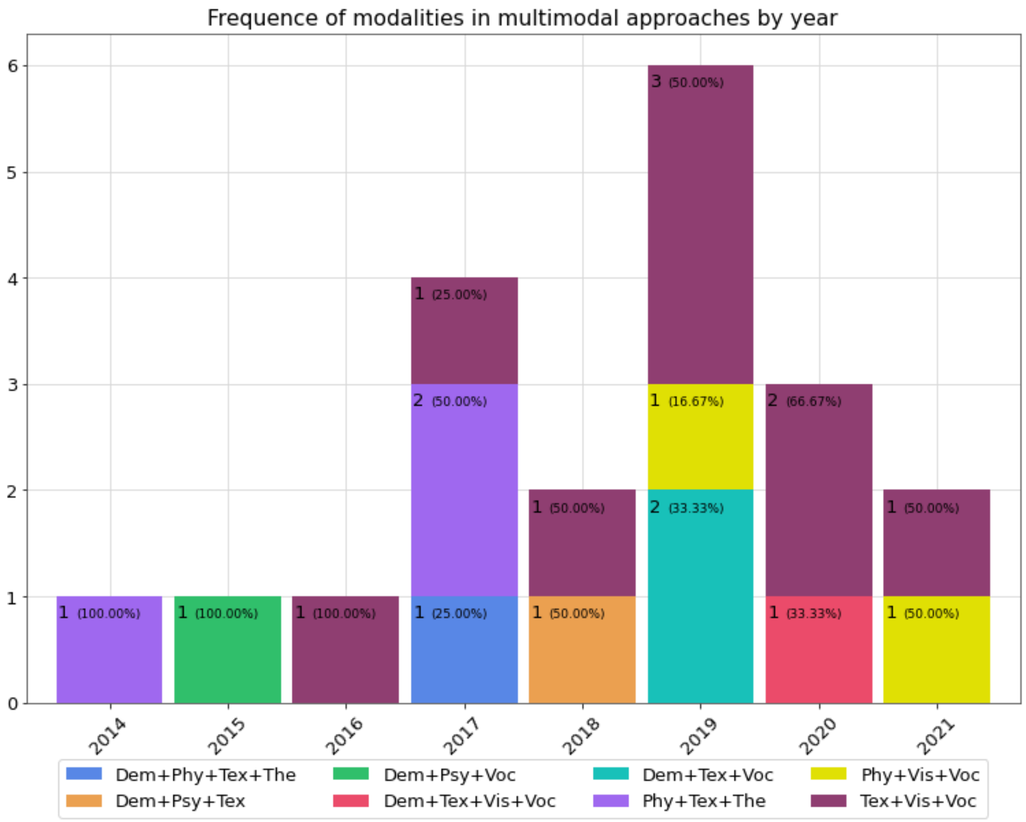

4. Features analysis

A feature is one data item used to describe a certain aspect that characterizes the collected behavior and are used as cues for deception detection.

The next charts summarize the metadata that describe the deception detection modality features used in the selected documents.

4.1 Textual features analysis

Textual features are verbal cues directly extracted from textual data, existing in written messages or transcribed from recordings.

According to theory, the behavioral changes that happen when someone is deceiving also affects the way such a person verbalizes, promoting variations of different kinds on what is written or said.

Those changes may be represented by various features extracted from text (written or transcribed).

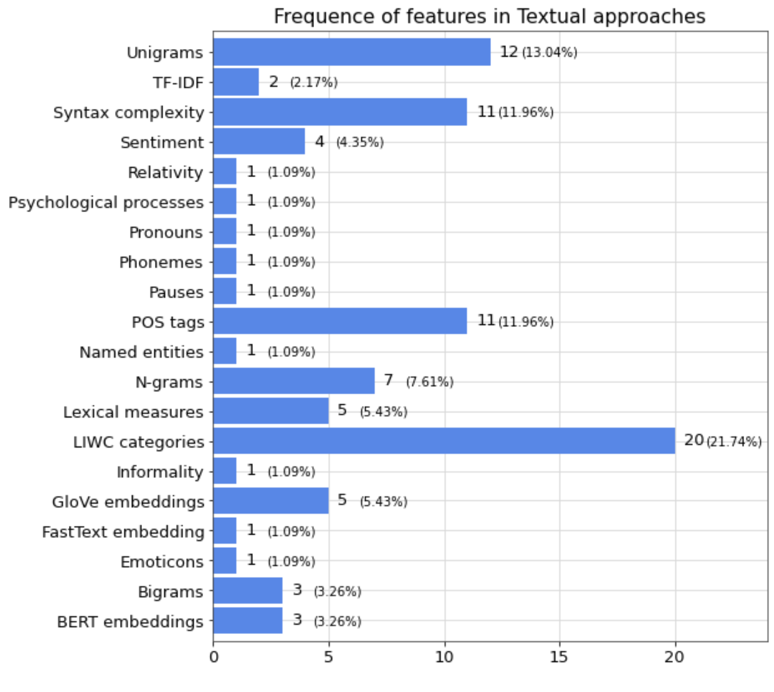

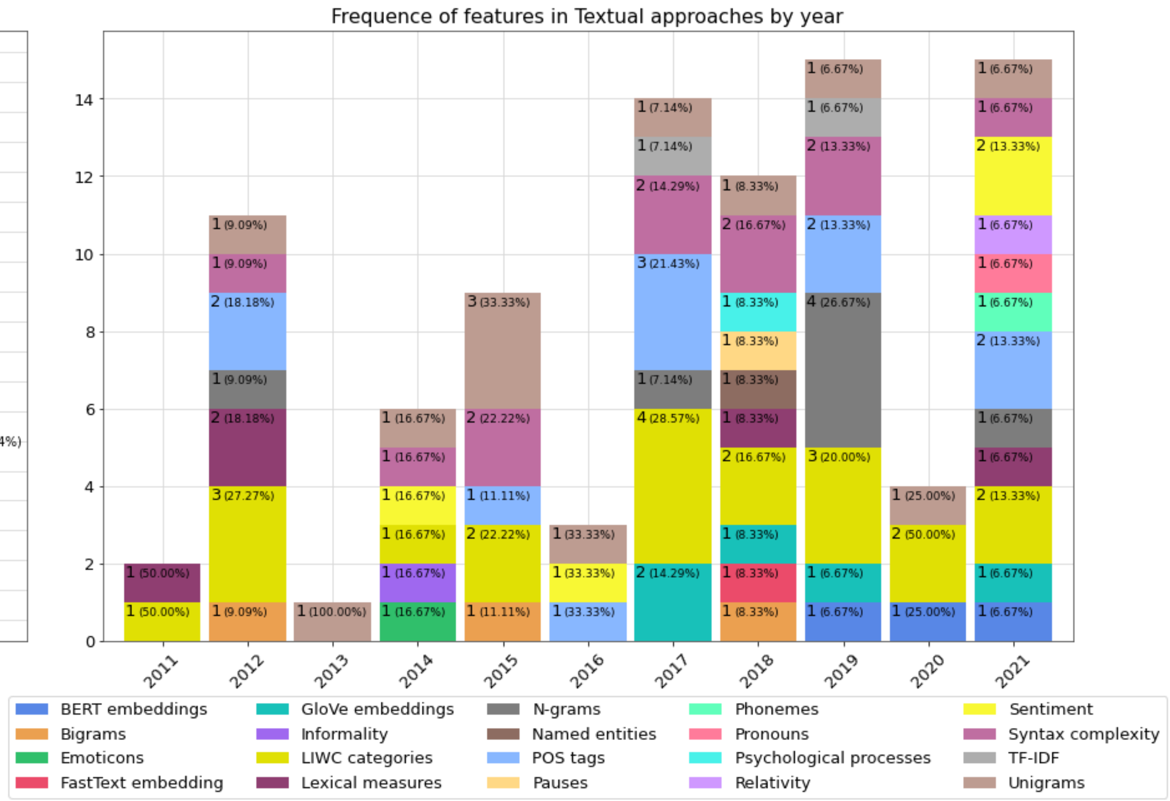

4.2. Visual features analysis

Visual features are non-verbal cues extracted from video recordings.

According to theory, the behavioral changes that happen when someone is deceiving also affects the way such a person moves or poses the body, members or the head, including facial expressions, eye gaze and blinks.

Those changes may be represented by various features extracted from video recordings.

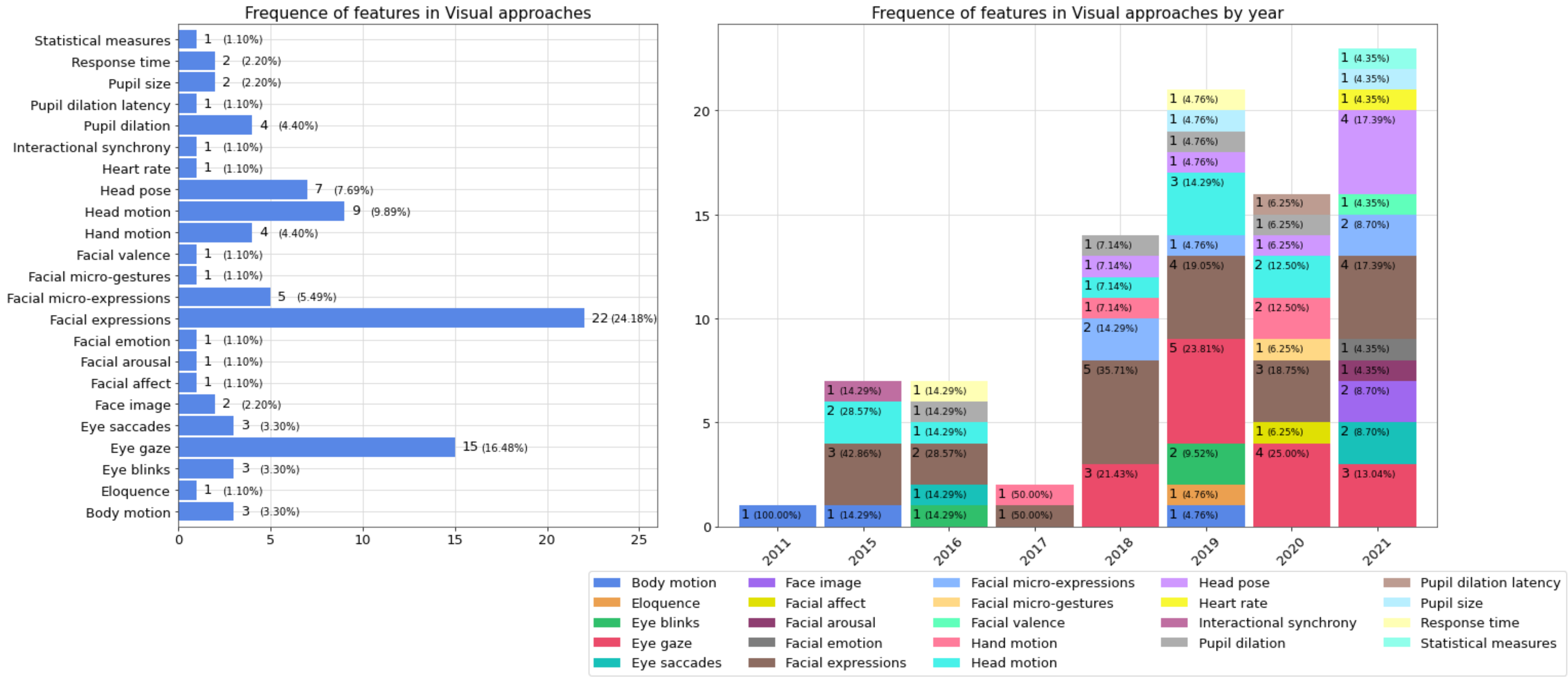

4.3. Vocal features analysis

Vocal features are non-verbal cues extracted from video or audio recordings.

According to theory, the behavioral changes that happen when someone is deceiving also affects the voice of such a person, in an acoustic sense.

Those changes may be represented by various features extracted from video ou audio recordings.

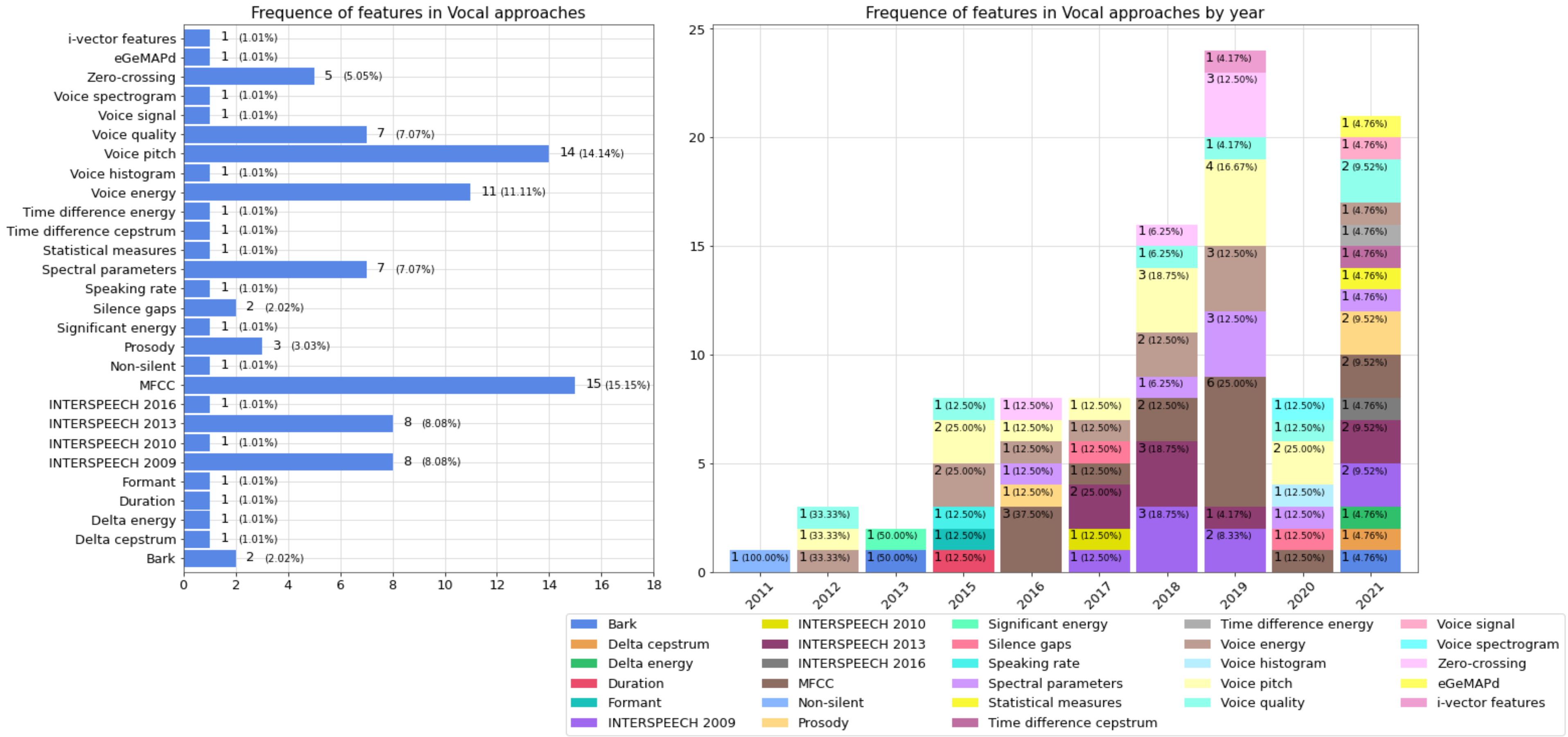

4.4. Thermal feature analysis

Thermal features are non-verbal cues extracted from thermal video recordings.

According to theory, the body changes that happen when someone is deceiving also affects the physiology of such a person, affecting the blood flow that alter the temperature of body parts.

Those changes may be represented by various features extracted from thermal video recordings.

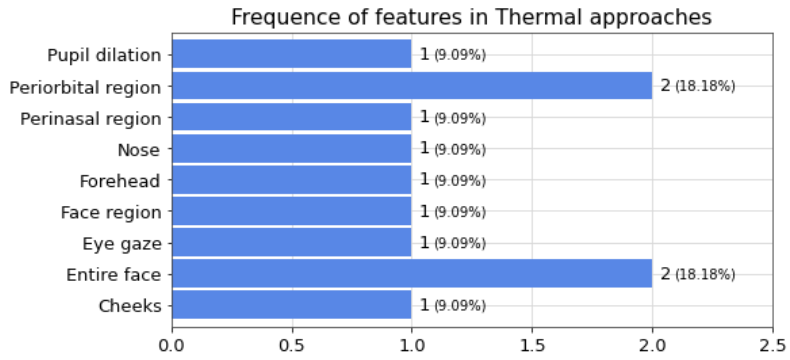

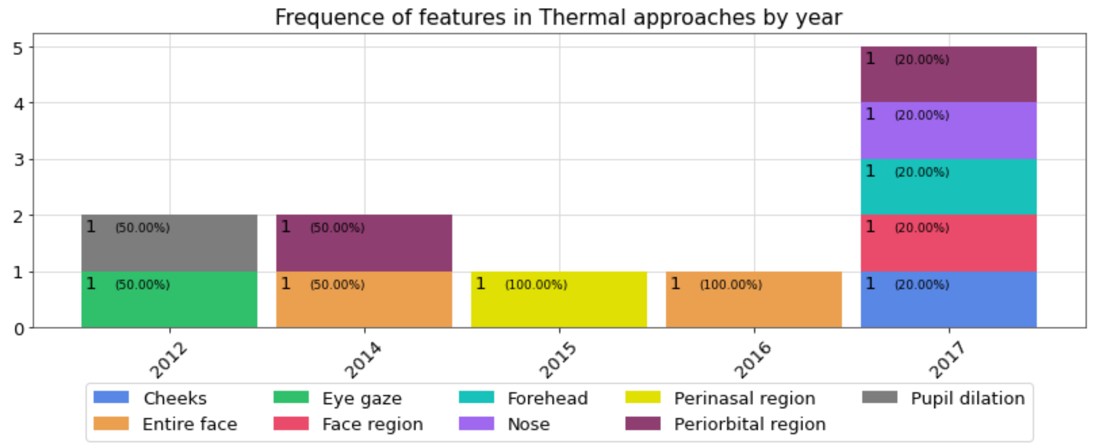

4.5. Demographical feature analysis

Demographical features are not cues for deception detection but are additional data that may establish behavior differences that may affect the detection process.

Theory suggests that deception cues may vary based on gender.

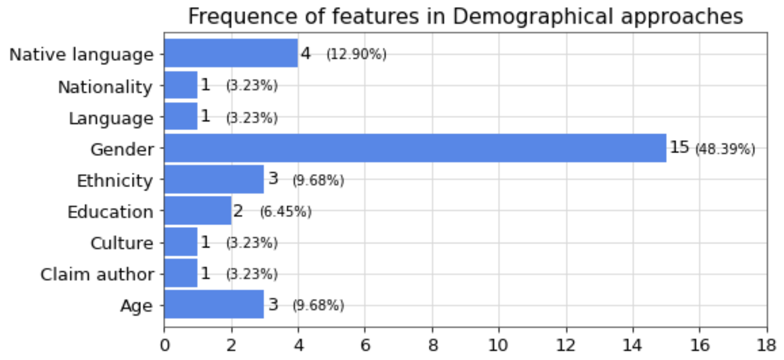

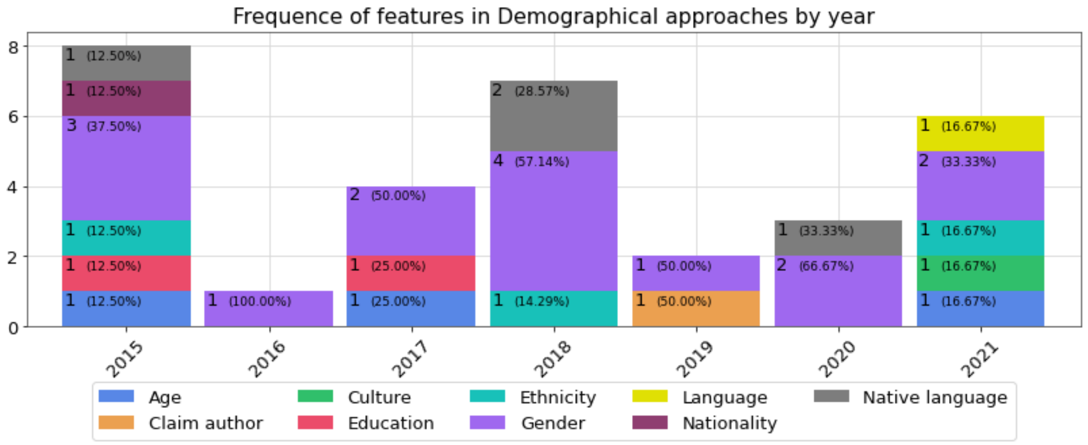

4.6. Physiological features analysis

Physiological features are non-verbal cues extracted from special sensors.

According to theory, the body changes that happen when someone is deceiving also affects the physiology of such a person, affecting a number of physiological functions.

Those changes may be represented by various features extracted from sensor readings.

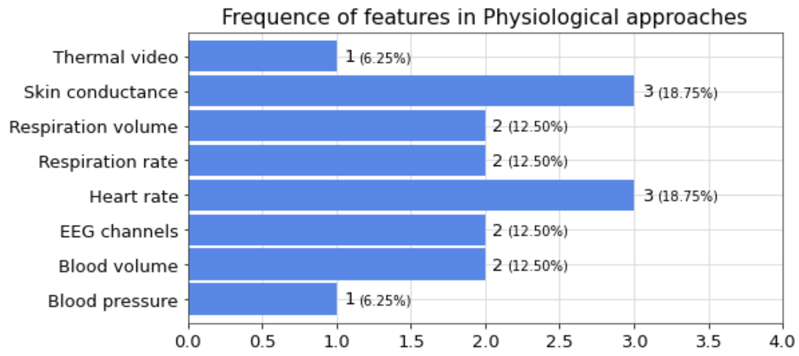

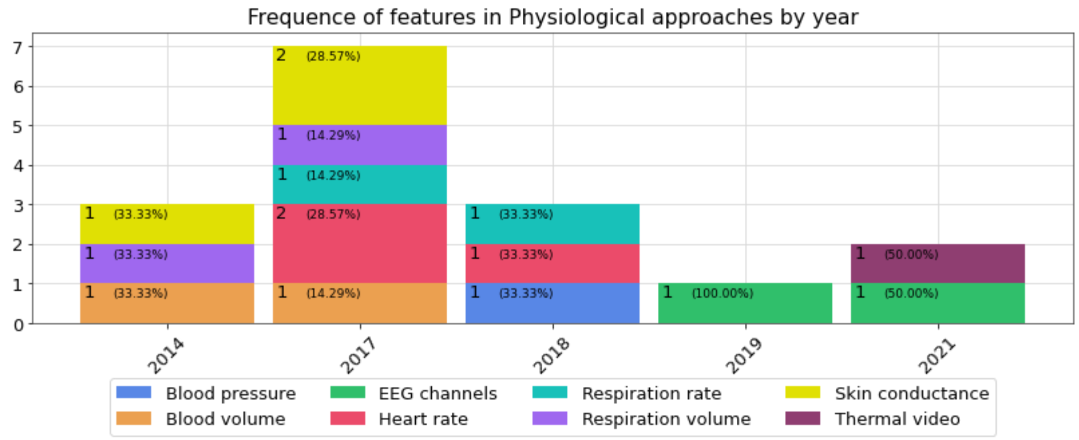

4.7. Remaining features analysis

Besides the previous modality-specific features, below there are some remaining modalities that were exploited in just a few studies, not justifying dedicated charts.

Remaining features exploited in studies

	year	title	features
**0**	2019	Joint Learning of Conversational Temporal Dynamics and Acoustic Features for Speech Deception Detection in Dialog Games	['Hesitation duration', 'Silence count', 'Turn duration', 'Utterance duration']
**0**	2019	How smart your smartphone is in lie detection?	['Hand shaking']
**1**	2020	Emotion Transformation Feature: Novel Feature For Deception Detection In Videos	['Emotion Transformation']
**0**	2019	Can a Robot Catch You Lying? A Machine Learning System to Detect Lies During Interactions	['Histronic', 'NARS', 'NEO-FFI scores', 'Narcisistic Machiavellianism']
**1**	2018	Linguistic cues to deception and perceived deception in interview dialogues	['NEO-FFI scores']
**2**	2015	Cross-Cultural Production and Detection of Deception from Speech	['NEO-FFI scores']

5. Machine Learning techniques analysis

The area of Machine Learning offers a wide range of possibilities for classification problems, not counting for clustering and association rules, among others. Authors exploited the Machine Learning arsenal by experimenting 26 different algorithms.

The next charts present some findings about the Machine Learning techniques used by the studies in the selected corpus.

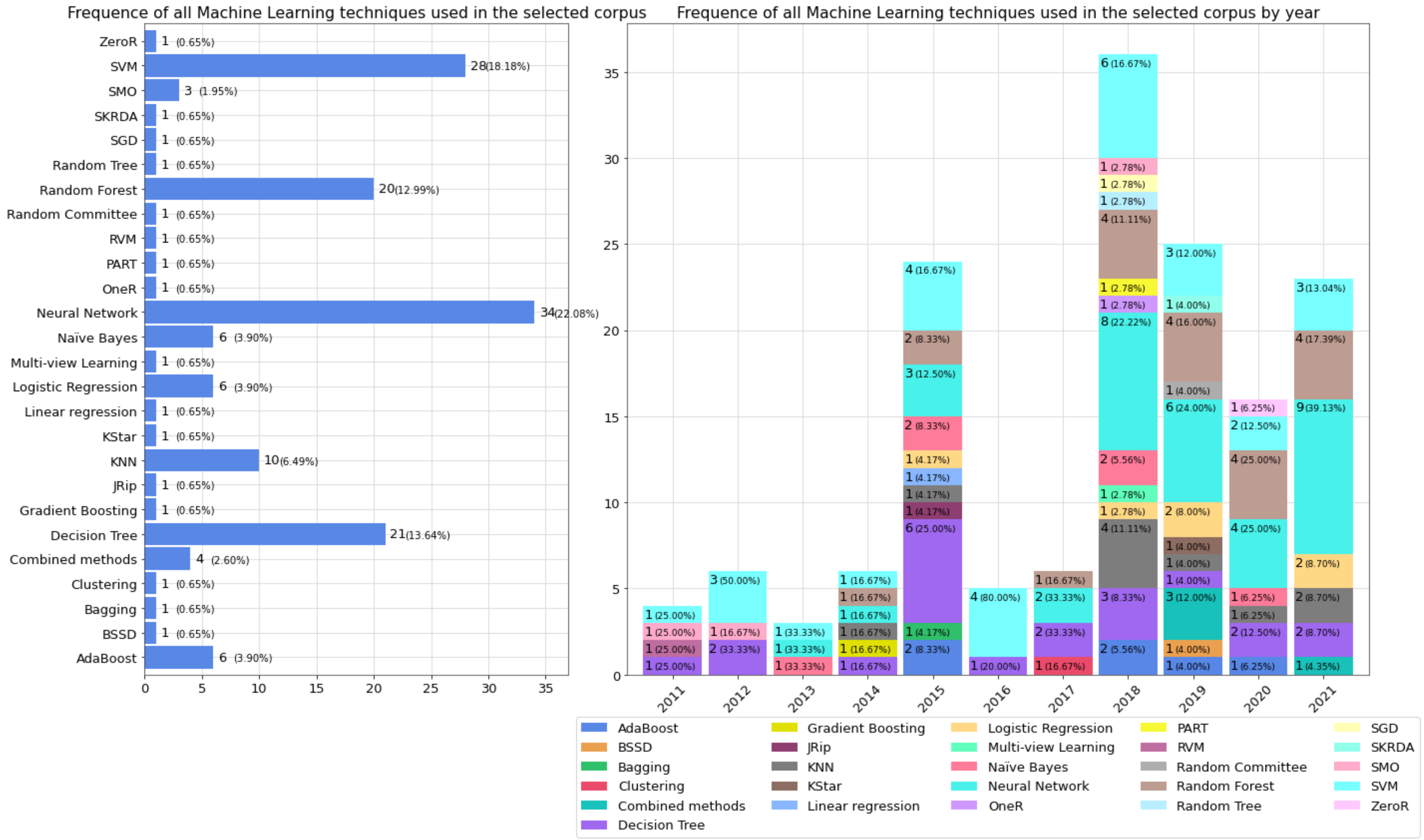

5.1. Rank of Machine Learning techniques

Some Machine Learning techniques were exploited by many different authors in diverse conditions. Others had less recurrence and some have one single case of use.

The charts belos present a rank of the top five most used techniques and the frequency of the remaining ones.

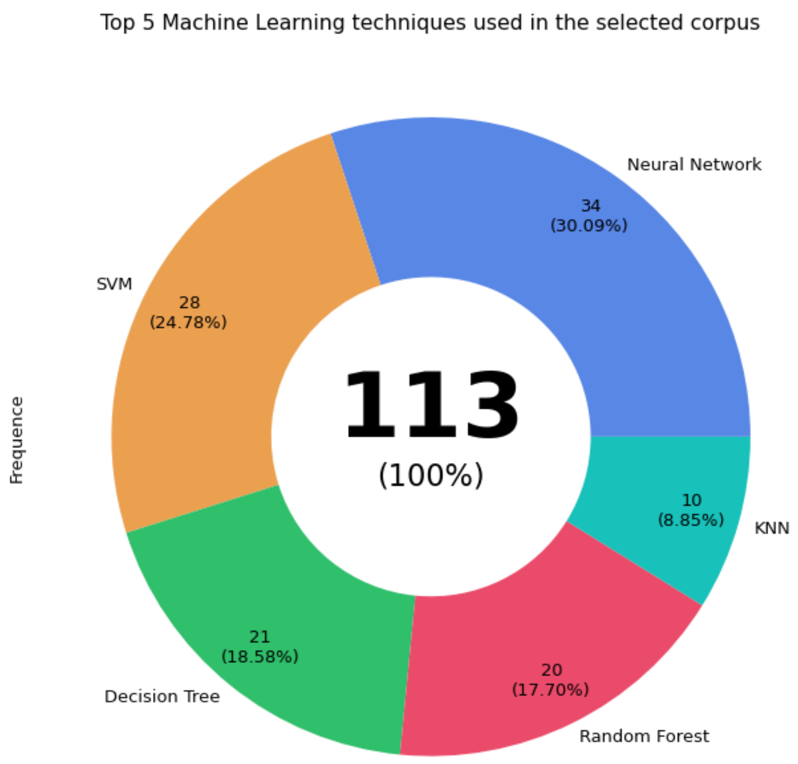

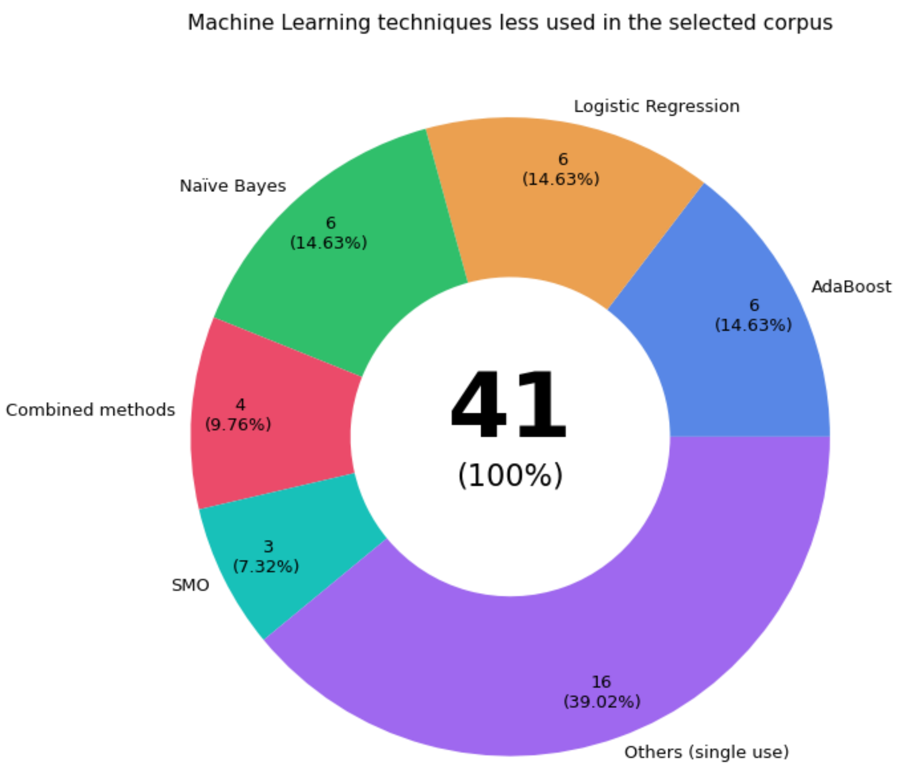

5.2. Machine Learning flavors

Some Machine Learning techniques have variations and sub models that are better suited for some kinds of problems or some levels of complexity. This section details those different flavors of the top 5 Machine Learning techniques. Only **Random Forest** does not present any variations.

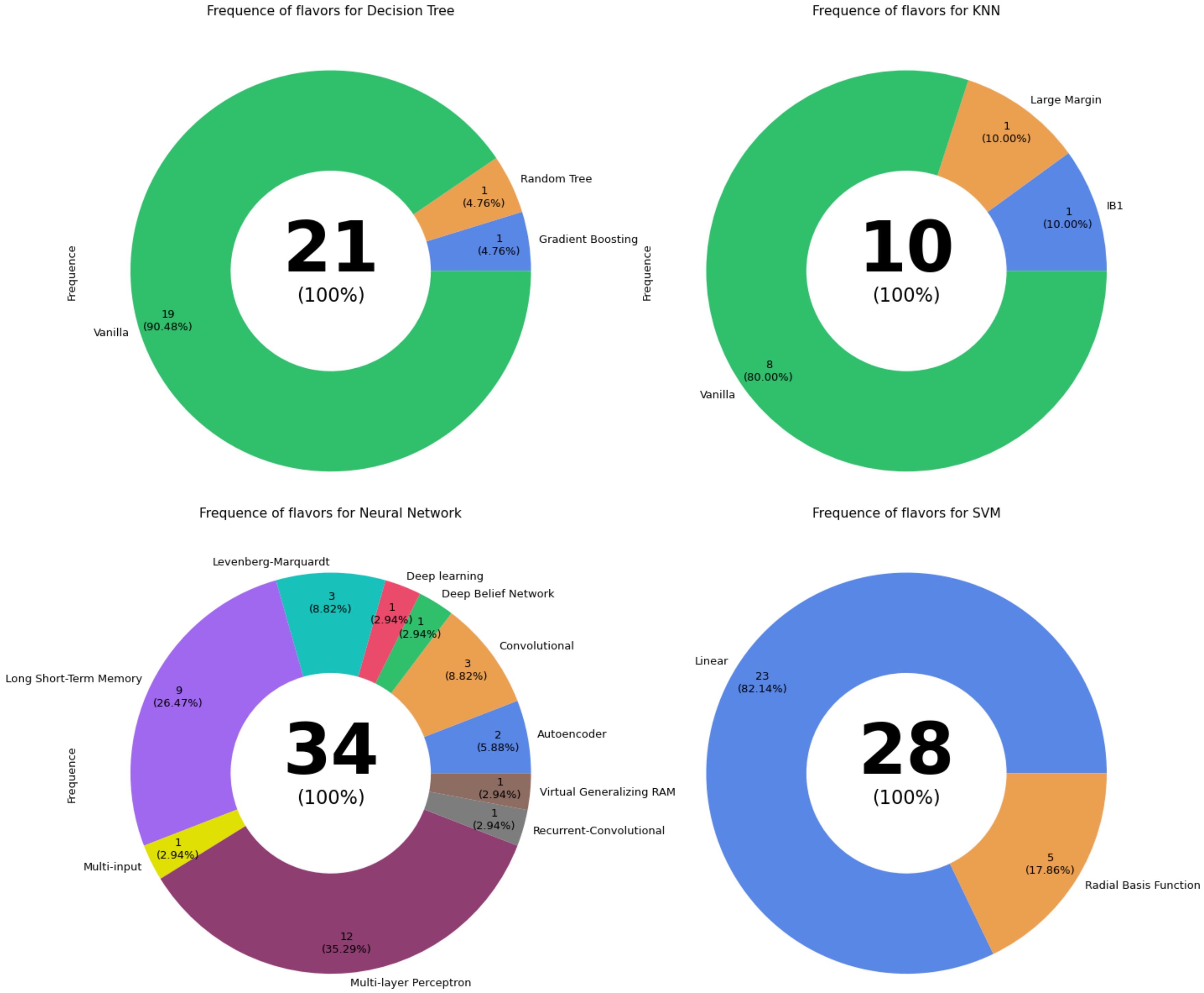

Frequence of flavors for Decision Tree
Random Tree
1
(4.76%)
Gradient Boosting
1
(4.76%)
21
(100%)
Frequence
19
(90.48%)
Vanilla
Frequence of flavors for KNN
Large Margin
1
(10.00%)
IB1
1
(10.00%)
10
(100%)
Frequence
8
(80.00%)
Vanilla
Frequence of flavors for Neural Network
Levenberg-Marquardt
3
(8.82%)
Deep learning
1
(2.94%)
Deep Belief Network
1
(2.94%)
Convolutional
3
(8.82%)
Long Short-Term Memory
9
(26.47%)
Autoencoder
2
(5.88%)
34
(100%)
Frequence
Virtual Generalizing RAM
1
(2.94%)
Recurrent-Convolutional
1
(2.94%)
Multi-input
1
(2.94%)
12
(35.29%)
Multi-layer Perceptron
Frequence of flavors for SVM
Linear
23
(82.14%)
28
(100%)
5
(17.86%)
Radial Basis Function

5.3. Performances of Machine Learning Techniques

The tables below list the performance of studies according to the technique used (Neural Network, Support Vector Machines, Random Forest, Decision Tree and K-Nearest Neighbor) and their most frequent variations (flavors).

The *Vanilla* flavor is used When a certain technique is used just as it was originally proposed.

Different flavors and all the performance metrics reported are showed.

5.3.1. Neural Networks

Performances achieved in studies based on Neural Network

	flavor	year	title	Accuracy	Area Under the Curve	F1-score	Precision	Recall	Unweighted Average Recall
28	Long Short-Term Memory	2021	Use of Machine Learning for Deception Detection From Spectral and Cepstral Features of Speech Signals	1.000000	nan	nan	nan	nan	nan
18	Recurrent-Convolutional	2019	Face-Focused Cross-Stream Network for Deception Detection in Videos	0.970000	0.997800	nan	nan	nan	nan
31	Convolutional	2021	LieNet: A Deep Convolution Neural Networks Framework for Detecting Deception	0.967375	nan	nan	nan	nan	nan
1	Convolutional	2017	Deep Learning Driven Multimodal Fusion For Automated Deception Detection	0.964000	nan	0.950000	0.960000	0.950000	nan
4	Multi-layer Perceptron	2020	Automated Deception Detection of Males and Females from Non-Verbal Facial Micro-Gestures	0.956500	nan	nan	nan	nan	nan
24	Long Short-Term Memory	2020	Building a Better Lie Detector with BERT: The Difference Between Truth and Lies	0.936000	nan	nan	nan	nan	nan
10	Multi-layer Perceptron	2018	Deception detection using artificial neural network and support vector machine	0.933300	nan	nan	nan	nan	nan
9	Multi-layer Perceptron	2015	A comparison of features for automatic deception detection in synchronous computer-mediated communication	0.920000	nan	nan	nan	nan	nan
26	Long Short-Term Memory	2021	Development of Spectral Speech Features for Deception Detection Using Neural Networks	0.916700	nan	nan	nan	nan	nan
29	Levenberg-Marquardt	2021	Use of Machine Learning for Deception Detection From Spectral and Cepstral Features of Speech Signals	0.875000	nan	nan	nan	nan	nan
23	Multi-layer Perceptron	2014	Cues to Deception in Social Media Communications	0.850000	nan	nan	nan	nan	nan
7	Long Short-Term Memory	2018	Interpretable Multimodal Deception Detection in Videos	0.841600	nan	nan	nan	nan	nan
21	Virtual Generalizing RAM	2018	Detection of Deception Using Facial Expressions Based on Different Classification Algorithms	0.840000	nan	nan	nan	nan	nan
16	Levenberg-Marquardt	2013	Deception detection in speech using bark band and perceptually significant energy features	0.833300	nan	nan	nan	nan	nan
20	Multi-layer Perceptron	2018	Detection of Deception Using Facial Expressions Based on Different Classification Algorithms	0.830000	nan	nan	nan	nan	nan
22	Multi-layer Perceptron	2015	Perinasal indicators of deceptive behavior	0.800000	nan	nan	nan	nan	nan
27	Levenberg-Marquardt	2021	Development of Spectral Speech Features for Deception Detection Using Neural Networks	0.791600	nan	nan	nan	nan	nan
15	Long Short-Term Memory	2018	Convolutional Bidirectional Long Short-Term Memory for Deception Detection With Acoustic Features	0.748700	nan	nan	nan	nan	nan
12	Multi-layer Perceptron	2018	Intelligent Deception Detection through Machine Based Interviewing	0.746050	nan	nan	nan	nan	nan

**11**	Multi-layer Perceptron	2020	Multimodal Deception Detection using Real-Life Trial Data	0.728800	nan	nan	nan	nan	nan
**32**	Multi-layer Perceptron	2021	Deception in the eyes of deceiver: A computer vision and machine learning based automated deception detection	0.720000	nan	0.740000	0.740000	0.700000	nan
**30**	Deep Belief Network	2021	Affect-Aware Deep Belief Network Representations for Multimodal Unsupervised Deception Detection	0.700000	0.800000	nan	0.880000	nan	nan
**33**	Autoencoder	2021	Deception detection in text and its relation to the cultural dimension of individualism/collectivism	0.695000	0.736667	0.726667	0.691667	0.770000	nan
**25**	Convolutional	2021	Identity Unbiased Deception Detection by 2D-to-3D Face Reconstruction	0.680000	nan	nan	0.660000	0.720000	nan
**17**	Multi-layer Perceptron	2018	Deception Detection and Analysis in Spoken Dialogues based on FastText	0.640000	nan	0.609000	0.667000	0.560000	nan
**8**	Multi-layer Perceptron	2018	An Empirical Study on Detecting Deception and Cybercrime Using Artificial Neural Networks	0.633333	0.800000	0.733333	0.733333	0.733333	nan
**5**	Autoencoder	2019	Improved semi-supervised autoencoder for deception detection	0.627800	nan	nan	nan	nan	nan
**6**	Deep learning	2020	Multilingual Deception Detection by Autonomous Agents	0.600000	nan	nan	0.520000	0.420000	nan
**19**	Multi-input	2019	Detecting Deception in Political Debates Using Acoustic and Textual Features	0.510400	nan	0.450700	nan	nan	nan
**0**	Long Short-Term Memory	2019	Detecting Concealed Information in Text and Speech	nan	nan	0.656150	nan	nan	nan
**2**	Long Short-Term Memory	2017	Hybrid Acoustic-Lexical Deep Learning Approach for Deception Detection	nan	nan	0.639000	nan	nan	nan
**3**	Multi-layer Perceptron	2015	Distinguishing Deception from Non-Deception in Chinese Speech	nan	nan	0.763300	0.761350	0.766800	nan
**13**	Long Short-Term Memory	2019	High-Level Features for Multimodal Deception Detection in Videos	nan	0.665000	nan	nan	nan	nan
**14**	Long Short-Term Memory	2019	Joint Learning of Conversational Temporal Dynamics and Acoustic Features for Speech Deception Detection in Dialog Games	nan	nan	nan	nan	nan	0.747100

Performances achieved in studies based on Multi-layer Perceptron

	**year**	**title**	**Accuracy**	**Area Under the Curve**	**F1-score**	**Precision**	**Recall**	**Unweighted Average Recall**
**4**	2020	Automated Deception Detection of Males and Females from Non-Verbal Facial Micro-Gestures	0.956500	nan	nan	nan	nan	nan
**10**	2018	Deception detection using artificial neural network and support vector machine	0.933300	nan	nan	nan	nan	nan
**9**	2015	A comparison of features for automatic deception detection in synchronous computer-mediated communication	0.920000	nan	nan	nan	nan	nan
**23**	2014	Cues to Deception in Social Media Communications	0.850000	nan	nan	nan	nan	nan
**20**	2018	Detection of Deception Using Facial Expressions Based on Different Classification Algorithms	0.830000	nan	nan	nan	nan	nan
**22**	2015	Perinasal indicators of deceptive behavior	0.800000	nan	nan	nan	nan	nan
**12**	2018	Intelligent Deception Detection through Machine Based Interviewing	0.746050	nan	nan	nan	nan	nan
**11**	2020	Multimodal Deception Detection using Real-Life Trial Data	0.728800	nan	nan	nan	nan	nan
**32**	2021	Deception in the eyes of deceiver: A computer vision and machine learning based automated deception detection	0.720000	nan	0.740000	0.740000	0.700000	nan
**17**	2018	Deception Detection and Analysis in Spoken Dialogues based on FastText	0.640000	nan	0.609000	0.667000	0.560000	nan
**8**	2018	An Empirical Study on Detecting Deception and Cybercrime Using Artificial Neural Networks	0.633333	0.800000	0.733333	0.733333	0.733333	nan
**3**	2015	Distinguishing Deception from Non-Deception in Chinese Speech	nan	nan	0.763300	0.761350	0.766800	nan

Descriptive statistics of Multi-layer Perceptron performances

	**Accuracy**	**Area Under the Curve**	**F1-score**	**Precision**	**Recall**	**Unweighted Average Recall**
**count**	11.000000	1.000000	4.000000	4.000000	4.000000	0.000000
**mean**	0.796180	0.800000	0.711408	0.725421	0.690033	nan
**std**	0.112986	nan	0.069470	0.040739	0.090877	nan

**min**	0.633333	0.800000	0.609000	0.667000	0.560000	nan
**25%**	0.724400	0.800000	0.702250	0.716750	0.665000	nan
**50%**	0.800000	0.800000	0.736667	0.736667	0.716667	nan
**75%**	0.885000	0.800000	0.745825	0.745337	0.741700	nan
**max**	0.956500	0.800000	0.763300	0.761350	0.766800	nan

Performances achieved in studies based on Long Short-Term Memory

	year	title	Accuracy	Area Under the Curve	F1-score	Precision	Recall	Unweighted Average Recall
**28**	2021	Use of Machine Learning for Deception Detection From Spectral and Cepstral Features of Speech Signals	1.000000	nan	nan	nan	nan	nan
**24**	2020	Building a Better Lie Detector with BERT: The Difference Between Truth and Lies	0.936000	nan	nan	nan	nan	nan
**26**	2021	Development of Spectral Speech Features for Deception Detection Using Neural Networks	0.916700	nan	nan	nan	nan	nan
**7**	2018	Interpretable Multimodal Deception Detection in Videos	0.841600	nan	nan	nan	nan	nan
**15**	2018	Convolutional Bidirectional Long Short-Term Memory for Deception Detection With Acoustic Features	0.748700	nan	nan	nan	nan	nan
**0**	2019	Detecting Concealed Information in Text and Speech	nan	nan	0.656150	nan	nan	nan
**2**	2017	Hybrid Acoustic-Lexical Deep Learning Approach for Deception Detection	nan	nan	0.639000	nan	nan	nan
**13**	2019	High-Level Features for Multimodal Deception Detection in Videos	nan	0.665000	nan	nan	nan	nan
**14**	2019	Joint Learning of Conversational Temporal Dynamics and Acoustic Features for Speech Deception Detection in Dialog Games	nan	nan	nan	nan	nan	0.747100

Descriptive statistics of Long Short-Term Memory performances

	Accuracy	Area Under the Curve	F1-score	Precision	Recall	Unweighted Average Recall
**count**	5.000000	1.000000	2.000000	0.000000	0.000000	1.000000
**mean**	0.888600	0.665000	0.647575	nan	nan	0.747100
**std**	0.096472	nan	0.012127	nan	nan	nan
**min**	0.748700	0.665000	0.639000	nan	nan	0.747100
**25%**	0.841600	0.665000	0.643288	nan	nan	0.747100
**50%**	0.916700	0.665000	0.647575	nan	nan	0.747100
**75%**	0.936000	0.665000	0.651863	nan	nan	0.747100
**max**	1.000000	0.665000	0.656150	nan	nan	0.747100

Performances achieved in studies based on Levenberg-Marquardt

	year	title	Accuracy	Area Under the Curve	F1-score	Precision	Recall	Unweighted Average Recall
**29**	2021	Use of Machine Learning for Deception Detection From Spectral and Cepstral Features of Speech Signals	0.875000	nan	nan	nan	nan	nan
**16**	2013	Deception detection in speech using bark band and perceptually significant energy features	0.833300	nan	nan	nan	nan	nan
**27**	2021	Development of Spectral Speech Features for Deception Detection Using Neural Networks	0.791600	nan	nan	nan	nan	Nan

Descriptive statistics of Levenberg-Marquardt performances

	Accuracy	Area Under the Curve	F1-score	Precision	Recall	Unweighted Average Recall
**count**	3.000000	0.000000	0.000000	0.000000	0.000000	0.000000
**mean**	0.833300	nan	nan	nan	nan	nan
**std**	0.041700	nan	nan	nan	nan	nan
**min**	0.791600	nan	nan	nan	nan	nan
**25%**	0.812450	nan	nan	nan	nan	nan

**50%**	0.833300	nan	nan	nan	nan	nan
**75%**	0.854150	nan	nan	nan	nan	nan
**max**	0.875000	nan	nan	nan	nan	Nan

Performances achieved in studies based on Convolutional Neural Networks

	year	title	Accuracy	Area Under the Curve	F1-score	Precision	Recall	Unweighted Average Recall
**31**	2021	LieNet: A Deep Convolution Neural Networks Framework for Detecting Deception	0.967375	nan	nan	nan	nan	nan
**1**	2017	Deep Learning Driven Multimodal Fusion For Automated Deception Detection	0.964000	nan	0.950000	0.960000	0.950000	nan
**25**	2021	Identity Unbiased Deception Detection by 2D-to-3D Face Reconstruction	0.680000	nan	nan	0.660000	0.720000	Nan

Descriptive statistics of Convolutional Neural Networks performances

	Accuracy	Area Under the Curve	F1-score	Precision	Recall	Unweighted Average Recall
**count**	3.000000	0.000000	1.000000	2.000000	2.000000	0.000000
**mean**	0.870458	nan	0.950000	0.810000	0.835000	nan
**std**	0.164950	nan	nan	0.212132	0.162635	nan
**min**	0.680000	nan	0.950000	0.660000	0.720000	nan
**25%**	0.822000	nan	0.950000	0.735000	0.777500	nan
**50%**	0.964000	nan	0.950000	0.810000	0.835000	nan
**75%**	0.965688	nan	0.950000	0.885000	0.892500	nan
**max**	0.967375	nan	0.950000	0.960000	0.950000	Nan

Performances achieved in studies based on Autoencoders

	year	title	Accuracy	Area Under the Curve	F1-score	Precision	Recall	Unweighted Average Recall
**33**	2021	Deception detection in text and its relation to the cultural dimension of individualism/collectivism	0.695000	0.736667	0.726667	0.691667	0.770000	nan
**5**	2019	Improved semi-supervised autoencoder for deception detection	0.627800	nan	nan	nan	nan	Nan

Descriptive statistics of Autoencoders performances

	Accuracy	Area Under the Curve	F1-score	Precision	Recall	Unweighted Average Recall
**count**	2.000000	1.000000	1.000000	1.000000	1.000000	0.000000
**mean**	0.661400	0.736667	0.726667	0.691667	0.770000	nan
**std**	0.047518	nan	nan	nan	nan	nan
**min**	0.627800	0.736667	0.726667	0.691667	0.770000	nan
**25%**	0.644600	0.736667	0.726667	0.691667	0.770000	nan
**50%**	0.661400	0.736667	0.726667	0.691667	0.770000	nan
**75%**	0.678200	0.736667	0.726667	0.691667	0.770000	nan
**max**	0.695000	0.736667	0.726667	0.691667	0.770000	Nan

5.3.2. Support Vector Machines (SVM)

Performances achieved in studies based on Support Vector Machines

	flavor	year	title	Accuracy	Area Under the Curve	F1-score	Precision	Recall
**0**	Linear	2018	Automated verbal credibility assessment of intentions: The model statement technique and predictive modeling	0.774200	nan	nan	nan	nan
**1**	Linear	2015	Distinguishing Deception from Non-Deception in Chinese Speech	nan	nan	0.774250	0.752250	0.803850

**2**	Linear	2018	Deception detection in videos	nan	0.903350	nan	nan	nan
**3**	Linear	2015	Experiments in open domain deception detection	0.695000	nan	nan	nan	nan
**4**	Linear	2012	Syntactic Stylometry for Deception Detection	0.912000	nan	nan	nan	nan
**5**	Radial Basis Function	2019	Automatic Deception Detection in RGB Videos Using Facial Action Units	0.768400	nan	nan	nan	nan
**6**	Linear	2012	Seeing through Deception: A Computational Approach to Deceit Detection in Written Communication	nan	nan	0.702000	nan	nan
**7**	Linear	2013	Automatic Detection of Deceit in Verbal Communication	0.737000	nan	nan	nan	nan
**8**	Linear	2012	On the Use of Homogenous Sets of Subjects in Deceptive Language Analysis	0.659600	nan	0.601200	0.718500	0.625600
**9**	Linear	2011	Move, and i Will Tell You Who You Are: Detecting Deceptive Roles in Low-Quality Data	nan	nan	0.760000	nan	nan
**10**	Linear	2018	An Empirical Study on Detecting Deception and Cybercrime Using Artificial Neural Networks	0.700000	0.696667	0.696667	0.700000	0.696667
**11**	Linear	2019	Robust Algorithm for Multimodal Deception Detection	0.760000	nan	nan	nan	nan
**12**	Radial Basis Function	2019	Speech Deception Detection Algorithm Based on SVM and Acoustic Features	0.824700	nan	nan	nan	nan
**13**	Linear	2015	A comparison of features for automatic deception detection in synchronous computer-mediated communication	0.780000	nan	nan	nan	nan
**14**	Linear	2018	Deception detection using artificial neural network and support vector machine	1.000000	nan	nan	nan	nan
**15**	Linear	2016	ReLiDSS: Novel lie detection system from speech signal	0.863750	nan	nan	nan	nan
**16**	Linear	2020	Emotion Transformation Feature: Novel Feature For Deception Detection In Videos	0.875900	nan	nan	nan	nan
**17**	Radial Basis Function	2020	Multimodal Deception Detection using Real-Life Trial Data	0.565000	nan	nan	nan	nan
**18**	Linear	2016	The Truth and Nothing But the Truth: Multimodal Analysis for Deception Detection	0.789500	nan	nan	nan	nan
**19**	Linear	2015	Is Interactional Dissynchrony a Clue to Deception? Insights From Automated Analysis of Nonverbal Visual Cues	nan	nan	nan	0.668000	0.659000
**20**	Linear	2018	Detection of Deception Using Facial Expressions Based on Different Classification Algorithms	0.830000	nan	nan	nan	nan
**21**	Linear	2016	Deceptive Speech Detection based on sparse representation	0.729500	nan	nan	nan	nan
**22**	Linear	2018	Construction of a Liar Corpus and Detection of Lying Situations	0.551600	nan	nan	0.551550	0.551450
**23**	Linear	2014	Cues to Deception in Social Media Communications	0.910000	nan	nan	nan	nan
**24**	Radial Basis Function	2016	Automated detection of user deception in on-line questionnaires with focus on eye tracking use	0.620000	nan	nan	0.640000	0.630000
**25**	Linear	2021	Non-invasive Deception Detection in Videos Using Machine Learning Techniques	0.615400	nan	0.628500	0.575800	0.697200
**26**	Radial Basis Function	2021	Deception Detection and Remote Physiological Monitoring: A Dataset and Baseline Experimental Results	0.626000	nan	nan	nan	nan
**27**	Linear	2021	Deception in the eyes of deceiver: A computer vision and machine learning based automated deception detection	0.770000	nan	0.780000	0.840000	0.690000

Performances achieved in studies based on Linear SVM

	year	title	Accuracy	Area Under the Curve	F1-score	Precision	Recall
**14**	2018	Deception detection using artificial neural network and support vector machine	1.000000	nan	nan	nan	nan
**4**	2012	Syntactic Stylometry for Deception Detection	0.912000	nan	nan	nan	nan
**23**	2014	Cues to Deception in Social Media Communications	0.910000	nan	nan	nan	nan
**16**	2020	Emotion Transformation Feature: Novel Feature For Deception Detection In Videos	0.875900	nan	nan	nan	nan
**15**	2016	ReLiDSS: Novel lie detection system from speech signal	0.863750	nan	nan	nan	nan
**20**	2018	Detection of Deception Using Facial Expressions Based on Different Classification Algorithms	0.830000	nan	nan	nan	nan
**18**	2016	The Truth and Nothing But the Truth: Multimodal Analysis for Deception Detection	0.789500	nan	nan	nan	nan
**13**	2015	A comparison of features for automatic deception detection in synchronous computer-mediated communication	0.780000	nan	nan	nan	nan
**0**	2018	Automated verbal credibility assessment of intentions: The model statement technique and predictive modeling	0.774200	nan	nan	nan	nan
**27**	2021	Deception in the eyes of deceiver: A computer vision and machine learning based automated deception detection	0.770000	nan	0.780000	0.840000	0.690000
**11**	2019	Robust Algorithm for Multimodal Deception Detection	0.760000	nan	nan	nan	nan

**7**	2013	Automatic Detection of Deceit in Verbal Communication	0.737000	nan	nan	nan	nan
**21**	2016	Deceptive Speech Detection based on sparse representation	0.729500	nan	nan	nan	nan
**10**	2018	An Empirical Study on Detecting Deception and Cybercrime Using Artificial Neural Networks	0.700000	0.696667	0.696667	0.700000	0.696667
**3**	2015	Experiments in open domain deception detection	0.695000	nan	nan	nan	nan
**8**	2012	On the Use of Homogenous Sets of Subjects in Deceptive Language Analysis	0.659600	nan	0.601200	0.718500	0.625600
**25**	2021	Non-invasive Deception Detection in Videos Using Machine Learning Techniques	0.615400	nan	0.628500	0.575800	0.697200
**22**	2018	Construction of a Liar Corpus and Detection of Lying Situations	0.551600	nan	nan	0.551550	0.551450
**1**	2015	Distinguishing Deception from Non-Deception in Chinese Speech	nan	nan	0.774250	0.752250	0.803850
**2**	2018	Deception detection in videos	nan	0.903350	nan	nan	nan
**6**	2012	Seeing through Deception: A Computational Approach to Deceit Detection in Written Communication	nan	nan	0.702000	nan	nan
**9**	2011	Move, and i Will Tell You Who You Are: Detecting Deceptive Roles in Low-Quality Data	nan	nan	0.760000	nan	nan
**19**	2015	Is Interactional Dissynchrony a Clue to Deception? Insights From Automated Analysis of Nonverbal Visual Cues	nan	nan	nan	0.668000	0.659000

Descriptive statistics of Linear SVM performances					
	**Accuracy**	**Area Under the Curve**	**F1-score**	**Precision**	**Recall**
**count**	18.000000	2.000000	7.000000	7.000000	7.000000
**mean**	0.775192	0.800008	0.706088	0.686586	0.674824
**std**	0.112046	0.146147	0.070878	0.099916	0.077191
**min**	0.551600	0.696667	0.601200	0.551550	0.551450
**25%**	0.707375	0.748337	0.662583	0.621900	0.642300
**50%**	0.772100	0.800008	0.702000	0.700000	0.690000
**75%**	0.855313	0.851679	0.767125	0.735375	0.696933
**max**	1.000000	0.903350	0.780000	0.840000	0.803850

Performances achieved in studies based on RBF SVM							
	**year**	**title**	**Accuracy**	**Area Under the Curve**	**F1-score**	**Precision**	**Recall**
**12**	2019	Speech Deception Detection Algorithm Based on SVM and Acoustic Features	0.824700	nan	nan	nan	nan
**5**	2019	Automatic Deception Detection in RGB Videos Using Facial Action Units	0.768400	nan	nan	nan	nan
**26**	2021	Deception Detection and Remote Physiological Monitoring: A Dataset and Baseline Experimental Results	0.626000	nan	nan	nan	nan
**24**	2016	Automated detection of user deception in on-line questionnaires with focus on eye tracking use	0.620000	nan	nan	0.640000	0.630000
**17**	2020	Multimodal Deception Detection using Real-Life Trial Data	0.565000	nan	nan	nan	Nan

Descriptive statistics of RBF SVM performances					
	**Accuracy**	**Area Under the Curve**	**F1-score**	**Precision**	**Recall**
**count**	5.000000	0.000000	0.000000	1.000000	1.000000
**mean**	0.680820	nan	nan	0.640000	0.630000
**std**	0.110103	nan	nan	nan	nan
**min**	0.565000	nan	nan	0.640000	0.630000
**25%**	0.620000	nan	nan	0.640000	0.630000
**50%**	0.626000	nan	nan	0.640000	0.630000
**75%**	0.768400	nan	nan	0.640000	0.630000
**max**	0.824700	nan	nan	0.640000	0.630000

5.3.3. Random Forest

Performances achieved in studies based on Random Forest								
	**year**	**title**	**Accuracy**	**Area Under the Curve**	**F1-score**	**Precision**	**Recall**	**Unweighted Average Recall**

**5**	2020	Automated Deception Detection of Males and Females from Non-Verbal Facial Micro-Gestures	0.998000	nan	nan	nan	nan	nan
**15**	2014	Cues to Deception in Social Media Communications	0.890000	nan	nan	nan	nan	nan
**7**	2019	How smart your smartphone is in lie detection?	0.830000	nan	0.850000	0.940000	0.780000	nan
**13**	2020	Emotion Transformation Feature: Novel Feature For Deception Detection In Videos	0.816600	nan	nan	nan	nan	nan
**2**	2018	Comparative Analysis of Classification Methods for Automatic Deception Detection in Speech	0.794000	nan	nan	nan	nan	0.793000
**17**	2021	Deception in the eyes of deceiver: A computer vision and machine learning based automated deception detection	0.780000	nan	0.800000	0.840000	0.720000	nan
**3**	2018	Acoustic-Prosodic Indicators of Deception and Trust in Interview Dialogues	0.745300	nan	0.716850	0.783700	0.660450	nan
**10**	2015	Deception Detection Using Real-Life Trial Data	0.735500	nan	nan	nan	nan	nan
**9**	2018	Linguistic cues to deception and perceived deception in interview dialogues	0.716350	nan	0.716000	0.716850	0.716050	nan
**18**	2021	How humans impair automated deception detection performance	0.690000	0.750000	nan	0.600000	0.760000	nan
**12**	2015	Cross-Cultural Production and Detection of Deception from Speech	0.658900	nan	nan	nan	nan	nan
**14**	2020	Multimodal Deception Detection using Real-Life Trial Data	0.632800	nan	nan	nan	nan	nan
**0**	2019	Can a Robot Catch You Lying? A Machine Learning System to Detect Lies During Interactions	0.630000	0.740000	nan	nan	nan	nan
**4**	2017	Construction and Analysis of Indonesian-Interviews Deception Corpus	0.612600	nan	0.613000	nan	nan	nan
**11**	2019	Automatic Deception Detection in RGB Videos Using Facial Action Units	0.584200	nan	nan	nan	nan	nan
**16**	2021	Non-invasive Deception Detection in Videos Using Machine Learning Techniques	0.567700	nan	0.585400	0.544300	0.648800	nan
**1**	2019	Detecting Concealed Information in Text and Speech	nan	nan	0.596300	nan	nan	nan
**6**	2020	Your eyes never lie: A robot magician can tell if you are lying	nan	0.897000	nan	0.833000	0.833000	nan
**8**	2018	Deception detection in videos	nan	0.873100	nan	nan	nan	nan
**19**	2021	Detecting Lies is a Child (Robot)'s Play: Gaze-Based Lie Detection in HRI	nan	0.733000	0.711000	nan	nan	Nan

Descriptive statistics of Random Forest performances

	Accuracy	Area Under the Curve	F1-score	Precision	Recall	Unweighted Average Recall
**count**	16.000000	5.000000	8.000000	7.000000	7.000000	1.000000
**mean**	0.730122	0.798620	0.698569	0.751121	0.731186	0.793000
**std**	0.118168	0.079580	0.096066	0.140314	0.065478	nan
**min**	0.567700	0.733000	0.585400	0.544300	0.648800	0.793000
**25%**	0.632100	0.740000	0.608825	0.658425	0.688250	0.793000
**50%**	0.725925	0.750000	0.713500	0.783700	0.720000	0.793000
**75%**	0.799650	0.873100	0.737637	0.836500	0.770000	0.793000
**max**	0.998000	0.897000	0.850000	0.940000	0.833000	0.793000

5.3.4. Decision Tree

Performances achieved in studies based on Decision Tree

	flavor	year	title	Accuracy	Area Under the Curve	F1-score	Precision	Recall	Unweighted Average Recall
**14**	Vanilla	2015	A comparison of features for automatic deception detection in synchronous computer-mediated communication	0.980000	nan	nan	nan	nan	nan
**3**	Vanilla	2020	Automated Deception Detection of Males and Females from Non-Verbal Facial Micro-Gestures	0.970000	nan	nan	nan	nan	nan
**18**	Vanilla	2018	Lie Detector With The Analysis Of The Change Of Diameter Pupil and The Eye Movement Use Method Gabor Wavelet Transform and Decision Tree	0.950000	nan	nan	0.970000	0.940000	nan
**13**	Vanilla	2012	The Voice and Eye Gaze Behavior of an Imposter: Automated Interviewing and Detection for Rapid Screening at the Border	0.944700	nan	nan	nan	nan	nan
**16**	Vanilla	2017	Detecting Deceptive Behavior via Integration of Discriminative Features From Multiple Modalities	0.892600	nan	nan	nan	0.892300	nan
**4**	Random Tree	2019	How smart your smartphone is in lie detection?	0.820000	nan	0.830000	0.910000	0.770000	nan

**17**	Vanilla	2015	Perinasal indicators of deceptive behavior	0.800000	nan	nan	nan	nan	nan
**15**	Vanilla	2020	Emotion Transformation Feature: Novel Feature For Deception Detection In Videos	0.765600	nan	nan	nan	nan	nan
**9**	Vanilla	2014	Deception Detection Using a Multimodal Approach	0.701000	nan	nan	nan	nan	nan
**20**	Vanilla	2021	Multimodal Political Deception Detection	0.700000	nan	nan	0.690000	nan	nan
**0**	Vanilla	2018	Comparative Analysis of Classification Methods for Automatic Deception Detection in Speech	0.696000	nan	nan	nan	nan	0.795000
**10**	Vanilla	2015	Deception Detection Using Real-Life Trial Data	0.685900	nan	nan	nan	nan	nan
**8**	Vanilla	2017	Gender-Based Multimodal Deception Detection	0.664000	nan	nan	nan	nan	nan
**7**	Vanilla	2011	Challenges in automated deception detection in computer-mediated communication	0.650000	nan	nan	nan	nan	nan
**6**	Vanilla	2012	Discerning truth from deception: Human judgments and automation efforts	0.650000	nan	nan	nan	nan	nan
**12**	Vanilla	2015	Cross-Cultural Production and Detection of Deception from Speech	0.648900	nan	nan	nan	nan	nan
**11**	Vanilla	2016	Analyzing Thermal and Visual Clues of Deception for a Non-Contact Deception Detection Approach	0.617400	nan	nan	nan	nan	nan
**19**	Vanilla	2021	Non-invasive Deception Detection in Videos Using Machine Learning Techniques	0.570800	nan	0.593200	0.532900	0.674200	nan
**1**	Gradient Boosting	2015	Distinguishing Deception from Non-Deception in Chinese Speech	nan	nan	0.820650	0.834600	0.819650	nan
**2**	Vanilla	2015	Distinguishing Deception from Non-Deception in Chinese Speech	nan	nan	0.809500	0.793400	0.835550	nan
**5**	Vanilla	2018	Deception detection in videos	nan	0.807400	nan	nan	nan	Nan

Performances achieved in studies based on Vanilla Decision Tree

	year	title	Accuracy	Area Under the Curve	F1-score	Precision	Recall	Unweighted Average Recall
**14**	2015	A comparison of features for automatic deception detection in synchronous computer-mediated communication	0.980000	nan	nan	nan	nan	nan
**3**	2020	Automated Deception Detection of Males and Females from Non-Verbal Facial Micro-Gestures	0.970000	nan	nan	nan	nan	nan
**18**	2018	Lie Detector With The Analysis Of The Change Of Diameter Pupil and The Eye Movement Use Method Gabor Wavelet Transform and Decision Tree	0.950000	nan	nan	0.970000	0.940000	nan
**13**	2012	The Voice and Eye Gaze Behavior of an Imposter: Automated Interviewing and Detection for Rapid Screening at the Border	0.944700	nan	nan	nan	nan	nan
**16**	2017	Detecting Deceptive Behavior via Integration of Discriminative Features From Multiple Modalities	0.892600	nan	nan	nan	0.892300	nan
**17**	2015	Perinasal indicators of deceptive behavior	0.800000	nan	nan	nan	nan	nan
**15**	2020	Emotion Transformation Feature: Novel Feature For Deception Detection In Videos	0.765600	nan	nan	nan	nan	nan
**9**	2014	Deception Detection Using a Multimodal Approach	0.701000	nan	nan	nan	nan	nan
**20**	2021	Multimodal Political Deception Detection	0.700000	nan	nan	0.690000	nan	nan
**0**	2018	Comparative Analysis of Classification Methods for Automatic Deception Detection in Speech	0.696000	nan	nan	nan	nan	0.795000
**10**	2015	Deception Detection Using Real-Life Trial Data	0.685900	nan	nan	nan	nan	nan
**8**	2017	Gender-Based Multimodal Deception Detection	0.664000	nan	nan	nan	nan	nan
**7**	2011	Challenges in automated deception detection in computer-mediated communication	0.650000	nan	nan	nan	nan	nan
**6**	2012	Discerning truth from deception: Human judgments and automation efforts	0.650000	nan	nan	nan	nan	nan
**12**	2015	Cross-Cultural Production and Detection of Deception from Speech	0.648900	nan	nan	nan	nan	nan
**11**	2016	Analyzing Thermal and Visual Clues of Deception for a Non-Contact Deception Detection Approach	0.617400	nan	nan	nan	nan	nan
**19**	2021	Non-invasive Deception Detection in Videos Using Machine Learning Techniques	0.570800	nan	0.593200	0.532900	0.674200	nan
**2**	2015	Distinguishing Deception from Non-Deception in Chinese Speech	nan	nan	0.809500	0.793400	0.835550	nan
**5**	2018	Deception detection in videos	nan	0.807400	nan	nan	nan	Nan

Descriptive statistics of Vanilla Decision Tree performances

	Accuracy	Area Under the Curve	F1-score	Precision	Recall	Unweighted Average Recall

**count**	17.000000	1.000000	2.000000	4.000000	4.000000	1.000000
**mean**	0.758053	0.807400	0.701350	0.746575	0.835512	0.795000
**std**	0.137027	nan	0.152947	0.183457	0.115707	nan
**min**	0.570800	0.807400	0.593200	0.532900	0.674200	0.795000
**25%**	0.650000	0.807400	0.647275	0.650725	0.795212	0.795000
**50%**	0.700000	0.807400	0.701350	0.741700	0.863925	0.795000
**75%**	0.892600	0.807400	0.755425	0.837550	0.904225	0.795000
**max**	0.980000	0.807400	0.809500	0.970000	0.940000	0.795000

5.3.5. K-Nearest Neighbor (KNN)

Performances achieved in studies based on K-Nearest Neighbor

	flavor	year	title	Accuracy	Area Under the Curve	F1-score	Precision	Recall	Unweighted Average Recall
**6**	Vanilla	2014	Thermal Facial Analysis for Deception Detection	0.868800	nan	nan	nan	nan	nan
**5**	Large Margin	2018	Toward End-to-End Deception Detection in Videos	0.841600	nan	nan	nan	nan	nan
**7**	Vanilla	2018	Detection of Deception Using Facial Expressions Based on Different Classification Algorithms	0.840000	nan	nan	nan	nan	nan
**4**	Vanilla	2020	Emotion Transformation Feature: Novel Feature For Deception Detection In Videos	0.824900	nan	nan	nan	nan	nan
**1**	IB1	2019	How smart your smartphone is in lie detection?	0.810000	nan	0.830000	0.950000	0.760000	nan
**3**	Vanilla	2015	A comparison of features for automatic deception detection in synchronous computer-mediated communication	0.780000	nan	nan	nan	nan	nan
**0**	Vanilla	2018	Comparative Analysis of Classification Methods for Automatic Deception Detection in Speech	0.763000	nan	nan	nan	nan	0.763000
**9**	Vanilla	2021	Unsupervised Audio-Visual Subspace Alignment for High-Stakes Deception Detection	0.740000	0.750000	0.730000	nan	nan	nan
**2**	Vanilla	2018	An Empirical Study on Detecting Deception and Cybercrime Using Artificial Neural Networks	0.633333	0.723333	0.680000	0.690000	0.680000	nan
**8**	Vanilla	2021	Non-invasive Deception Detection in Videos Using Machine Learning Techniques	0.572300	nan	0.493000	0.577900	0.442900	Nan

Performances achieved in studies based on Vanilla KNN

	year	title	Accuracy	Area Under the Curve	F1-score	Precision	Recall	Unweighted Average Recall
**6**	2014	Thermal Facial Analysis for Deception Detection	0.868800	nan	nan	nan	nan	nan
**7**	2018	Detection of Deception Using Facial Expressions Based on Different Classification Algorithms	0.840000	nan	nan	nan	nan	nan
**4**	2020	Emotion Transformation Feature: Novel Feature For Deception Detection In Videos	0.824900	nan	nan	nan	nan	nan
**3**	2015	A comparison of features for automatic deception detection in synchronous computer-mediated communication	0.780000	nan	nan	nan	nan	nan
**0**	2018	Comparative Analysis of Classification Methods for Automatic Deception Detection in Speech	0.763000	nan	nan	nan	nan	0.763000
**9**	2021	Unsupervised Audio-Visual Subspace Alignment for High-Stakes Deception Detection	0.740000	0.750000	0.730000	nan	nan	nan
**2**	2018	An Empirical Study on Detecting Deception and Cybercrime Using Artificial Neural Networks	0.633333	0.723333	0.680000	0.690000	0.680000	nan
**8**	2021	Non-invasive Deception Detection in Videos Using Machine Learning Techniques	0.572300	nan	0.493000	0.577900	0.442900	Nan

Descriptive statistics of Vanilla K-Nearest Neighbor performances

	Accuracy	Area Under the Curve	F1-score	Precision	Recall	Unweighted Average Recall

**count**	8.000000	2.000000	3.000000	2.000000	2.000000	1.000000
**mean**	0.752792	0.736667	0.634333	0.633950	0.561450	0.763000
**std**	0.102900	0.018856	0.124925	0.079267	0.167655	nan
**min**	0.572300	0.723333	0.493000	0.577900	0.442900	0.763000
**25%**	0.713333	0.730000	0.586500	0.605925	0.502175	0.763000
**50%**	0.771500	0.736667	0.680000	0.633950	0.561450	0.763000
**75%**	0.828675	0.743333	0.705000	0.661975	0.620725	0.763000
**max**	0.868800	0.750000	0.730000	0.690000	0.680000	0.763000

6. Support tool analysis

Authors reported the use of many different tools used to run many kinds of processing on data. In this section we present charts that show which tools were used and how frequent.

The next charts present some findings about the supporting tools used by the studies in the selected corpus.

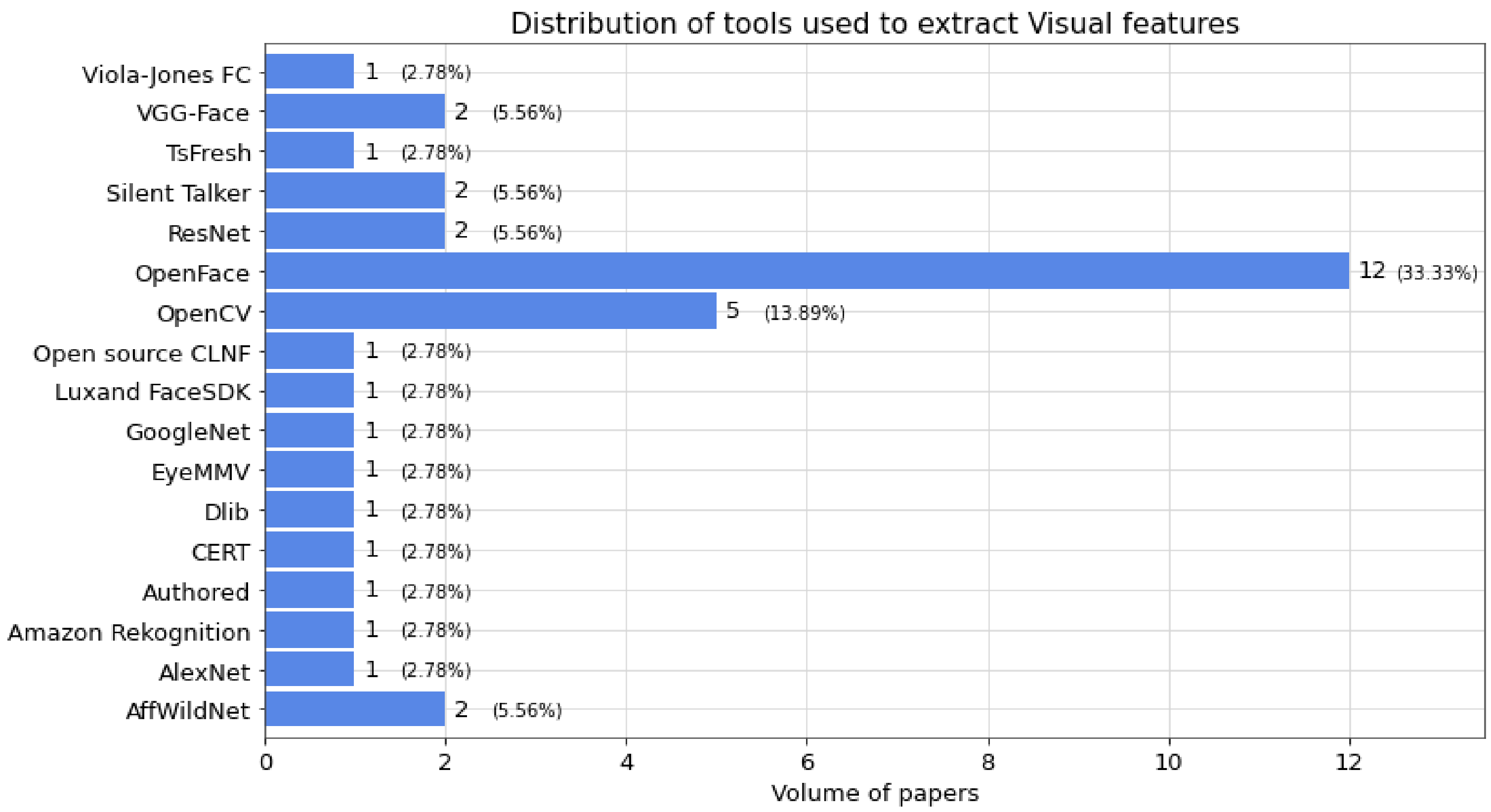

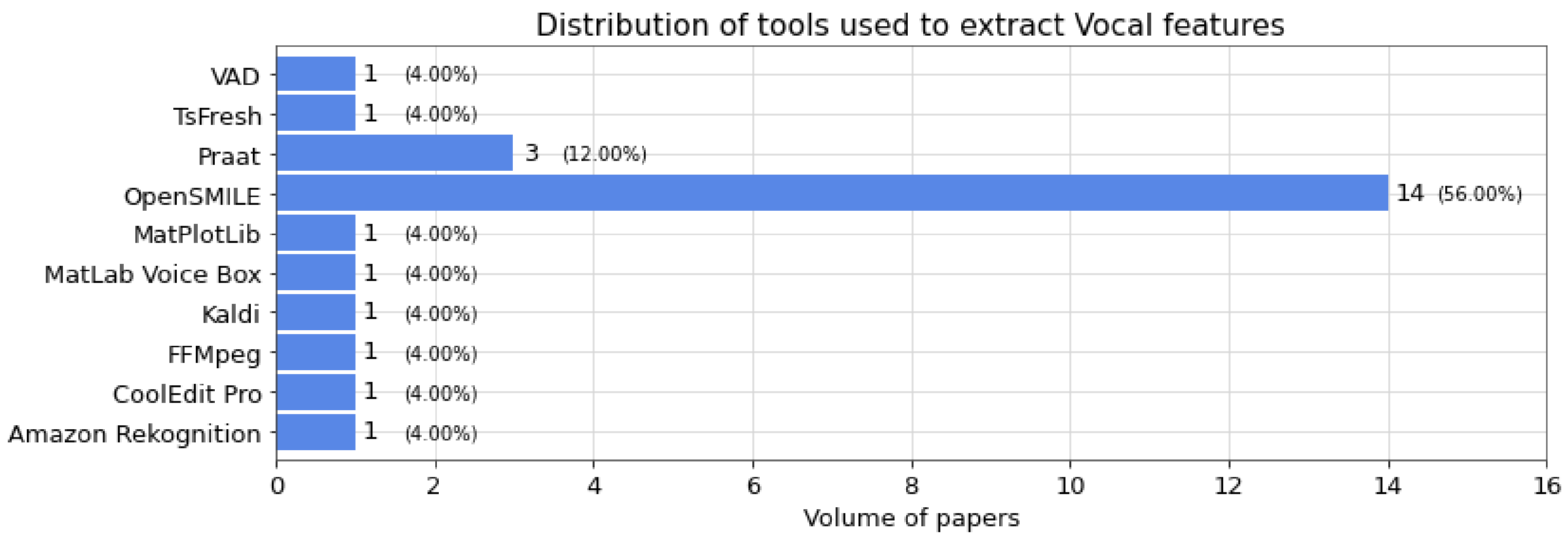
Distribution of tools used to extract Vocal features
VAD
TsFresh
Praat
OpenSMILE
MatPlotLib
MatLab Voice Box
Kaldi
FFMpeg
CoolEdit Pro
Amazon Rekognition
1 (4.00%)
1 (4.00%)
3 (12.00%)
14 (56.00%)
1 (4.00%)
1 (4.00%)
1 (4.00%)
1 (4.00%)
1 (4.00%)
1 (4.00%)
0
2
4
6
8
10
12
14
16
Volume of papers

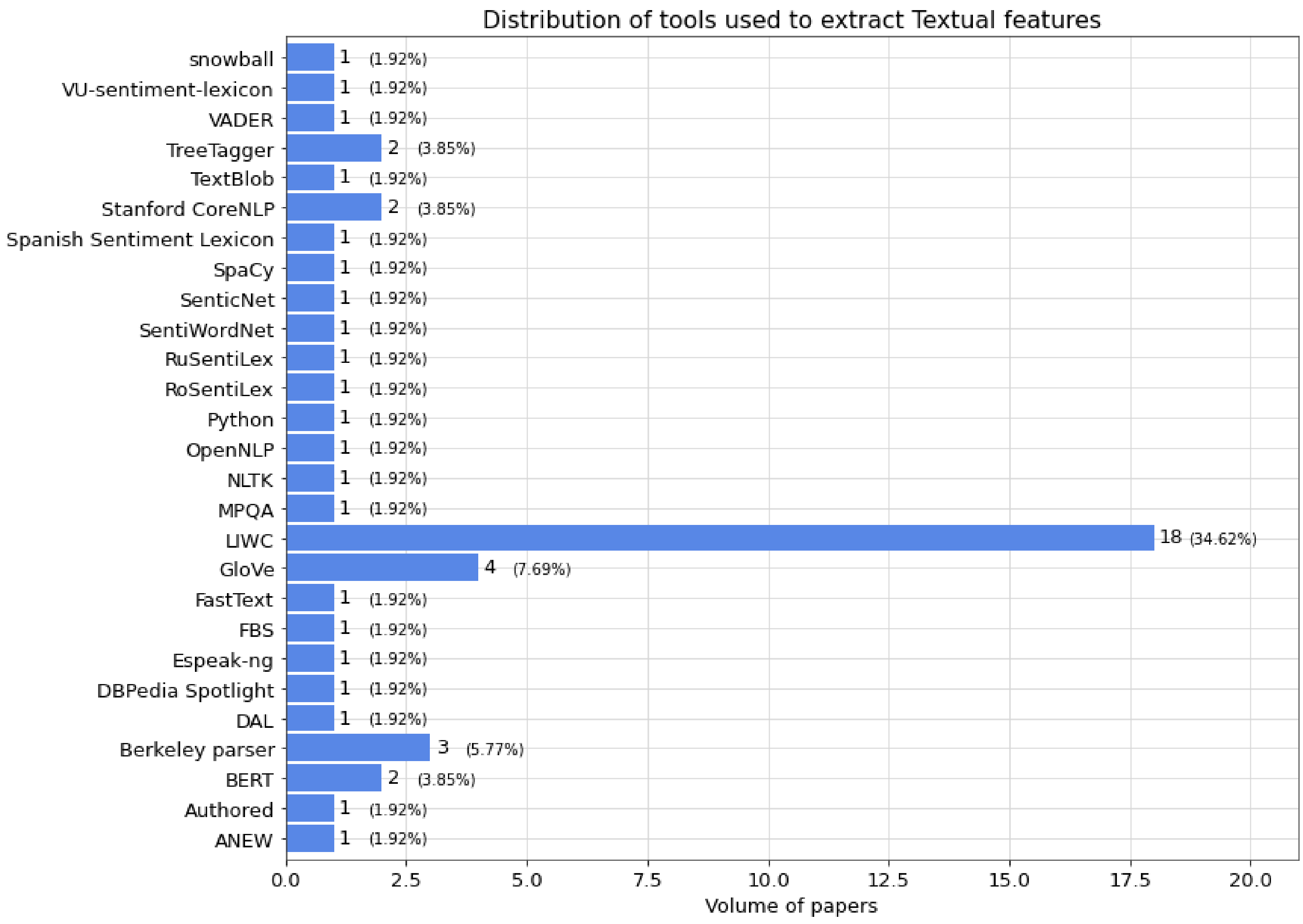

Distribution of tools used to extract Textual features
snowball 1 (1.92%)
VU-sentiment-lexicon 1 (1.92%)
VADER 1 (1.92%)
TreeTagger 2 (3.85%)
TextBlob 1 (1.92%)
Stanford CoreNLP 2 (3.85%)
Spanish Sentiment Lexicon 1 (1.92%)
SpaCy 1 (1.92%)
SenticNet 1 (1.92%)
SentiWordNet 1 (1.92%)
RuSentiLex 1 (1.92%)
RoSentiLex 1 (1.92%)
Python 1 (1.92%)
OpenNLP 1 (1.92%)
NLTK 1 (1.92%)
MPQA 1 (1.92%)
LIWC 18 (34.62%)
GloVe 4 (7.69%)
FastText 1 (1.92%)
FBS 1 (1.92%)
Espeak-ng 1 (1.92%)
DBPedia Spotlight 1 (1.92%)
DAL 1 (1.92%)
Berkeley parser 3 (5.77%)
BERT 2 (3.85%)
Authored 1 (1.92%)
ANEW 1 (1.92%)
0.0 2.5 5.0 7.5 10.0 12.5 15.0 17.5 20.0
Volume of papers

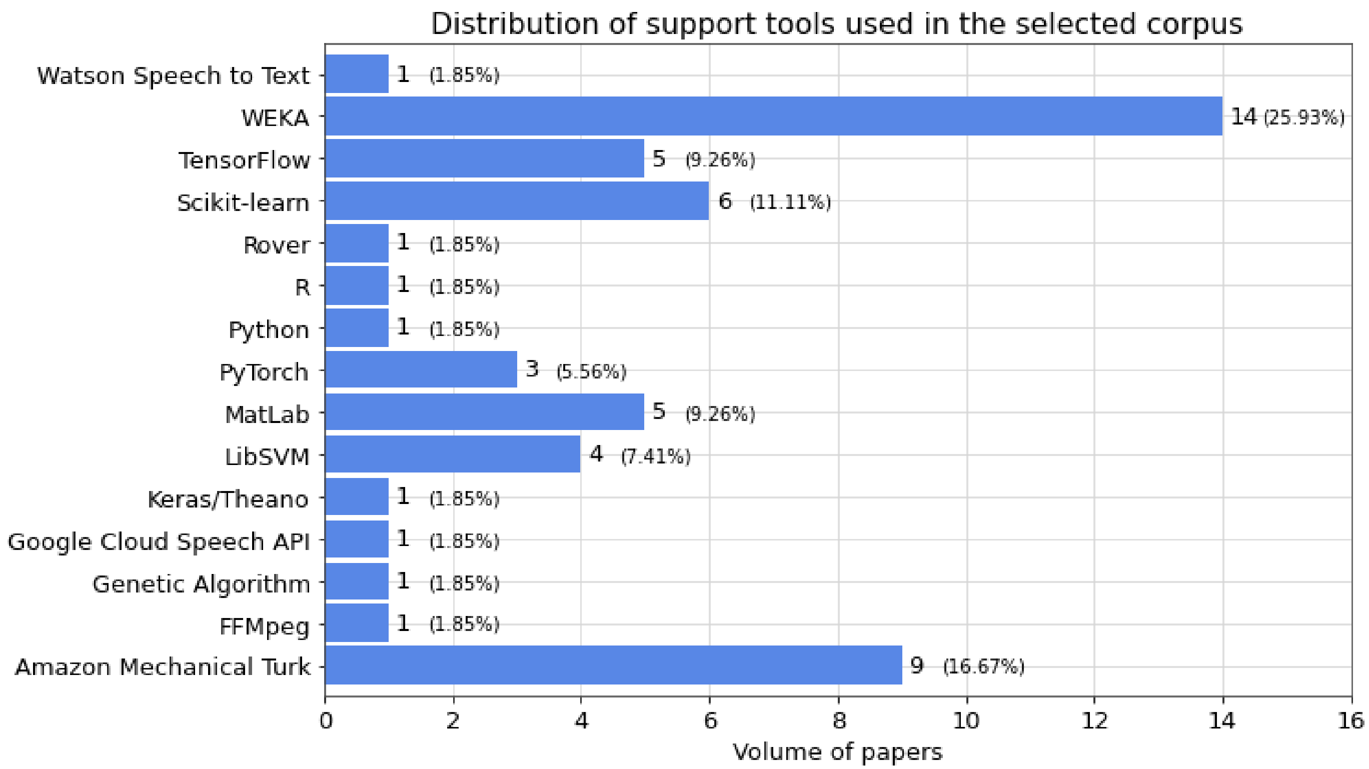

Distribution of support tools used in the selected corpus
Watson Speech to Text
WEKA
TensorFlow
Scikit-learn
Rover
R
Python
PyTorch
MatLab
LibSVM
Keras/Theano
Google Cloud Speech API
Genetic Algorithm
FFMpeg
Amazon Mechanical Turk
1 (1.85%)
14 (25.93%)
5 (9.26%)
6 (11.11%)
1 (1.85%)
1 (1.85%)
1 (1.85%)
3 (5.56%)
5 (9.26%)
4 (7.41%)
1 (1.85%)
1 (1.85%)
1 (1.85%)
1 (1.85%)
9 (16.67%)
0
2
4
6
8
10
12
14
16
Volume of papers

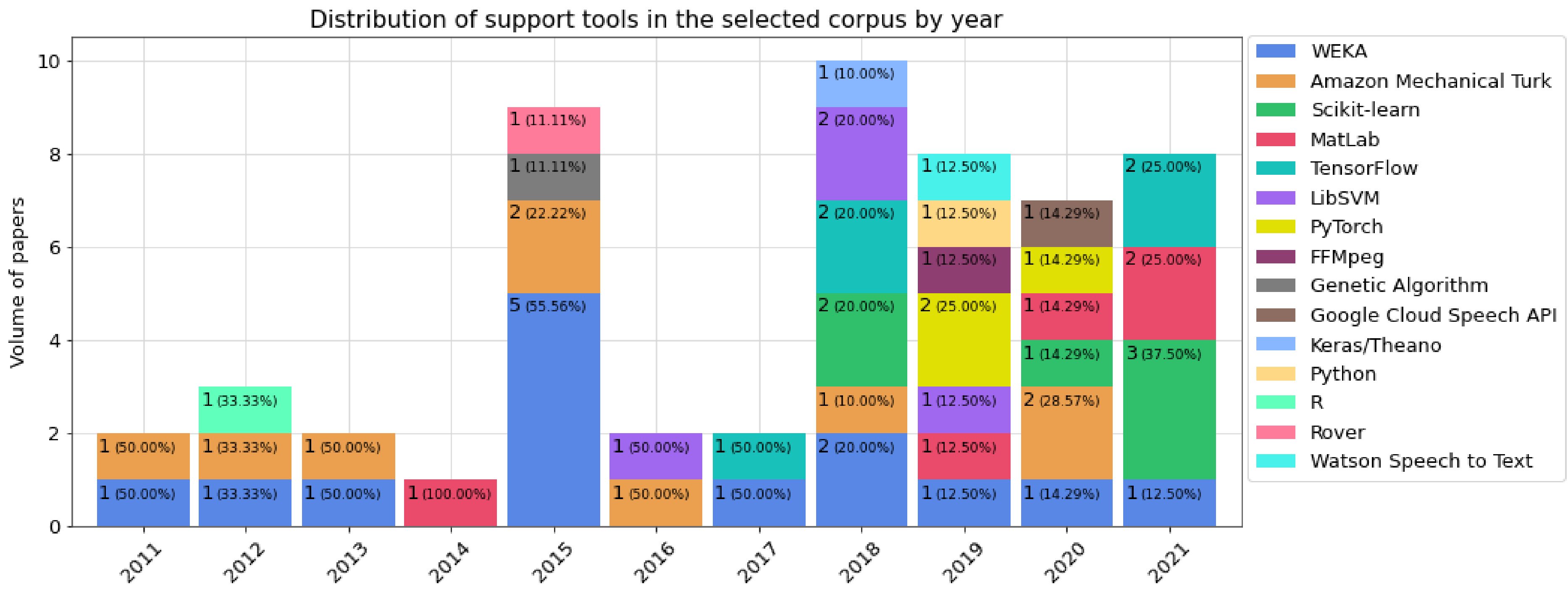

7. Dataset analysis

In this section we gather data extracted about datasets exploited in each selected study and present some analysis. What we seek here is to give a perception of what data were consumed to train models, which ones are available for future research, what data nature is stored in those datasets and how many times they were used in different papers.

At the end we present a dataset benchmark table that is intended to summarize all we could collect about the dataset used for research on Deception Detection.

7.2. Dataset origin analysis

Due to data scarcity on Deception Detection, many authors reported that they had to devise a data collection strategy. In such cases those datasets are named as "Author's collected dataset". The data existing in those datasets are labeled as "Mock" because the settings elaborated tried to create a circumstance where participants would be encouraged to lie so the desired features could be collected.

On the other hand, some authors produced or consumed datasets created from real-life situations, which means, datasets composed by features of people that were, supposedly, lying expontaneously. Those data are labeled "Real-life".

The next charts present a study on the volume of those two kinds of data origin and their distribution along the period of interest.

The conclusion is that Mock data is still the majority, but there is an increasing and persistent tendency to exploit Real-life data.

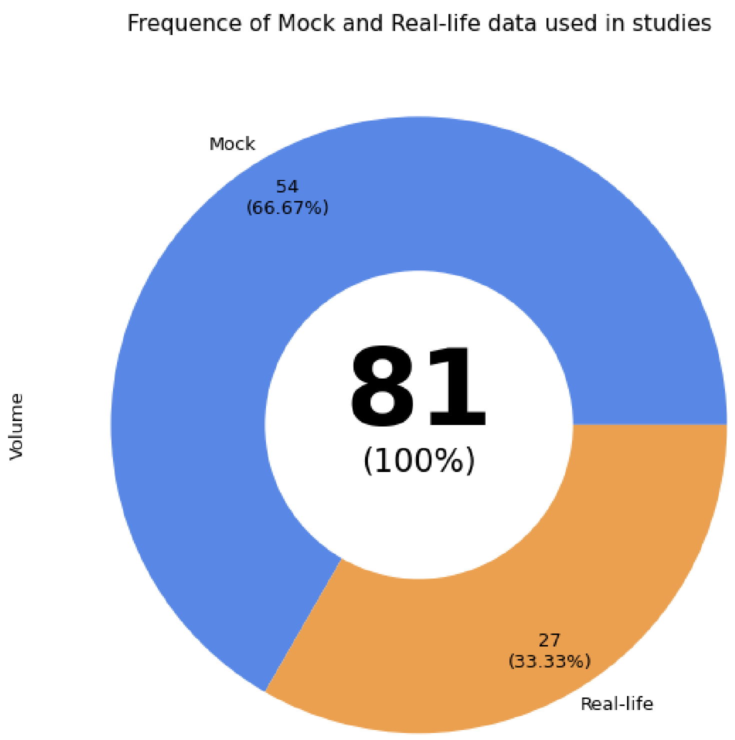

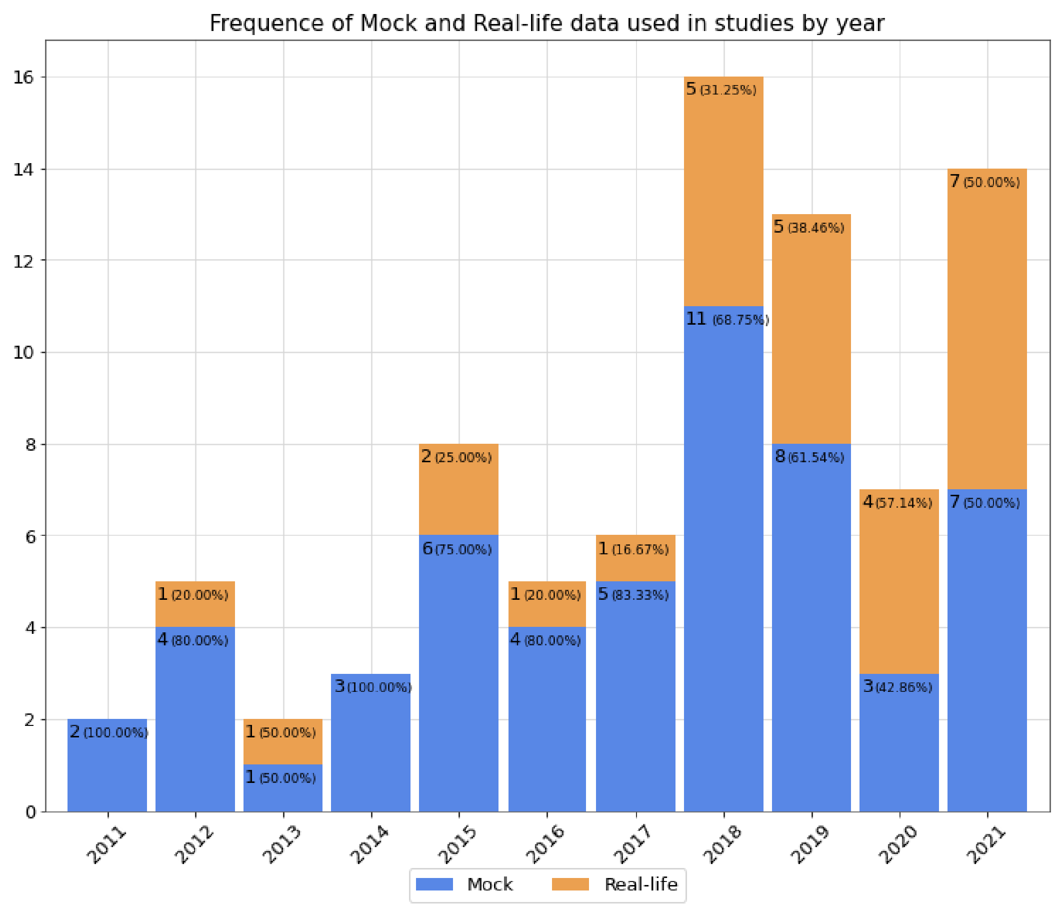

7.3. Dataset access

Not all datasets collected in studies were made available to community access.

The next charts present a study on public availability of datasets, their distribution along the period of interest, and their use by different papers.

The conclusion is that open access datasets are still the minority, but there is an increasing and persistent tendency to adopt those datasets. However, there is a strong recurrence of the Real-life Deception Detection Dataset.

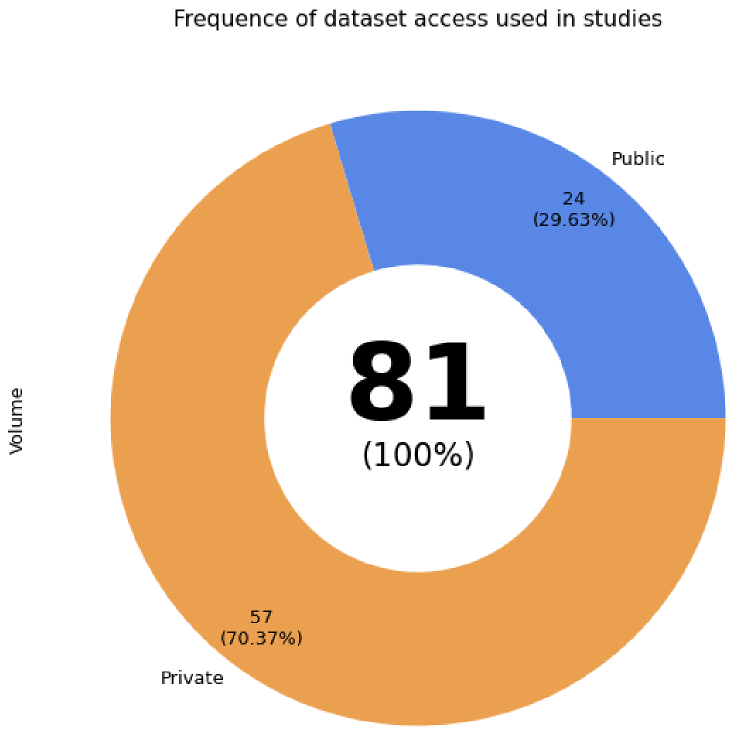

Frequence of dataset access used in studies
Volume
Public
24
(29.63%)
81
(100%)
57
(70.37%)
Private

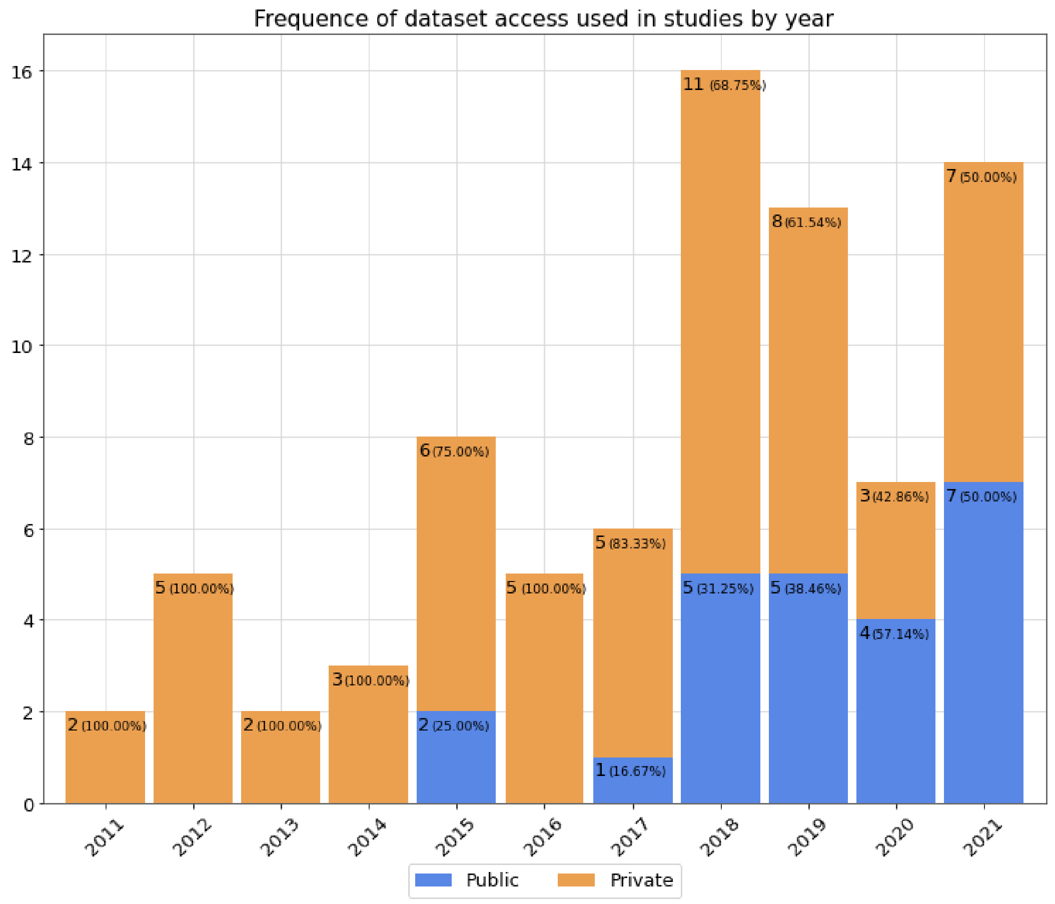

Frequence of dataset access used in studies by year
0
2
4
6
8
10
12
14
16
2 (100.00%)
5 (100.00%)
2 (100.00%)
3 (100.00%)
6 (75.00%)
2 (25.00%)
5 (100.00%)
5 (83.33%)
1 (16.67%)
11 (68.75%)
5 (31.25%)
8 (61.54%)
5 (38.46%)
3 (42.86%)
4 (57.14%)
7 (50.00%)
7 (50.00%)
2011
2012
2013
2014
2015
2016
2017
2018
2019
2020
2021
Public
Private

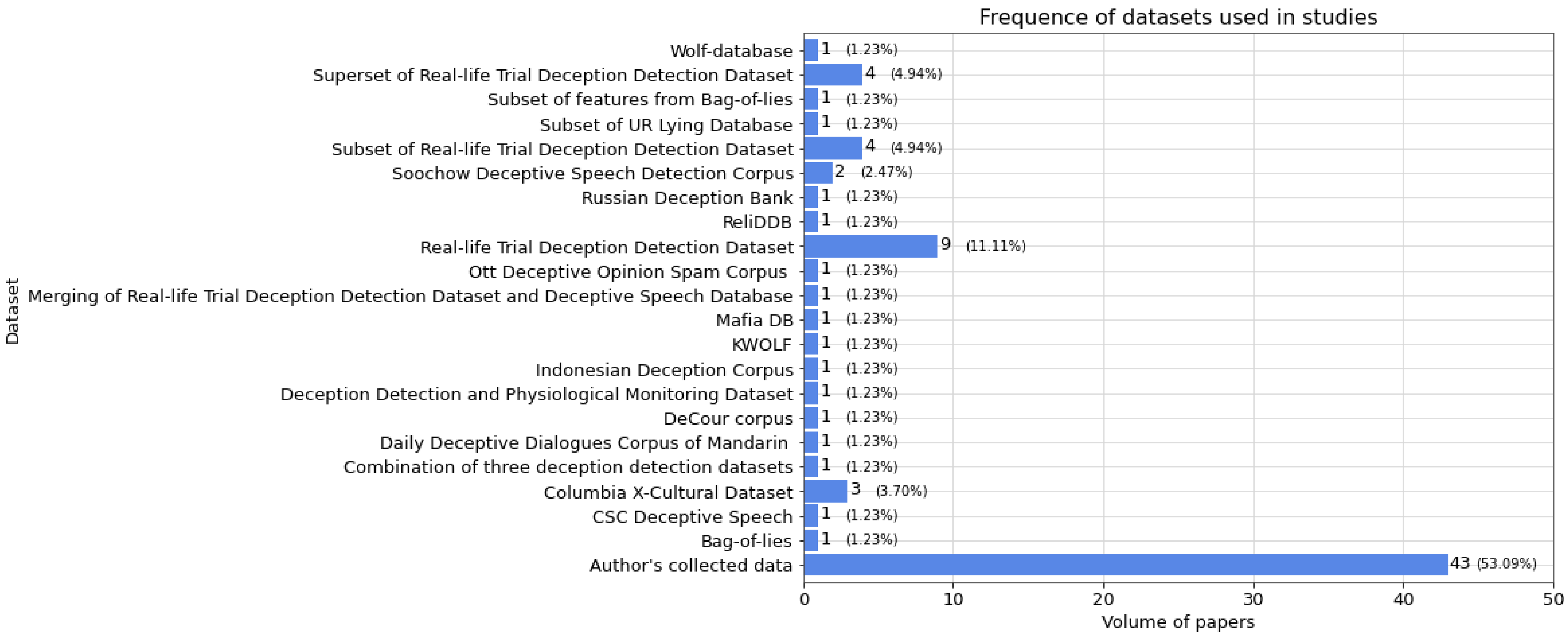

Frequence of datasets used in studies
Wolf-database
Superset of Real-life Trial Deception Detection Dataset
Subset of features from Bag-of-lies
Subset of UR Lying Database
Subset of Real-life Trial Deception Detection Dataset
Soochow Deceptive Speech Detection Corpus
Russian Deception Bank
ReliDDB
Real-life Trial Deception Detection Dataset
Ott Deceptive Opinion Spam Corpus
Merging of Real-life Trial Deception Detection Dataset and Deceptive Speech Database
Mafia DB
KWOLF
Indonesian Deception Corpus
Deception Detection and Physiological Monitoring Dataset
DeCour corpus
Daily Deceptive Dialogues Corpus of Mandarin
Combination of three deception detection datasets
Columbia X-Cultural Dataset
CSC Deceptive Speech
Bag-of-lies
Author's collected data
1 (1.23%)
4 (4.94%)
1 (1.23%)
1 (1.23%)
4 (4.94%)
2 (2.47%)
1 (1.23%)
1 (1.23%)
9 (11.11%)
1 (1.23%)
1 (1.23%)
1 (1.23%)
1 (1.23%)
1 (1.23%)
1 (1.23%)
1 (1.23%)
1 (1.23%)
1 (1.23%)
3 (3.70%)
1 (1.23%)
1 (1.23%)
43 (53.09%)
Dataset
0
10
20
30
40
50
Volume of papers

Frequence of open access datasets used in studies

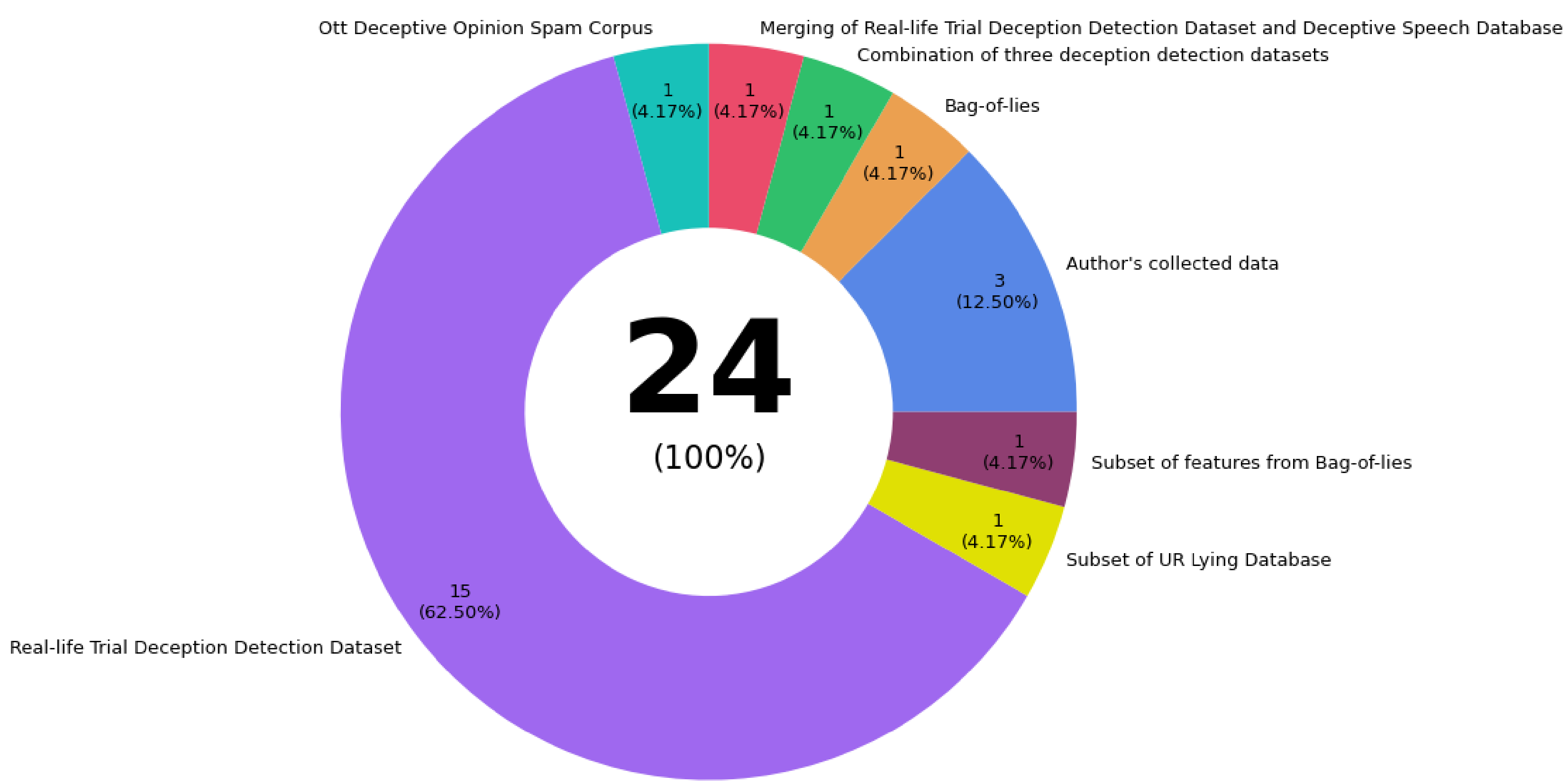

Summary of datasets used in studies in the selected corpus

	year	Paper	Author	Name	Origin	Public	Real-life	Cardinality	Modality combination	Features
**72**	2021	Affect-Aware Deep Belief Network Representations for Multimodal Unsupervised Deception Detection	Mathur, Leena, Matarić, Maja J	Subset of Real-life Trial Deception Detection Dataset	Real-life Trial Deception Detection Dataset	True	True	108	Visual + Vocal	Eye gaze, Facial arousal, Facial expressions, Facial valence, Head pose, MFCC, Prosody, Spectral parameters, Voice energy, Voice quality
**78**	2021	Automatic Detection of Deceptive and Truthful Paralinguistic Information in Speech using Two-	Velichko, A.N., Karpov, A.A.	Merging of Real-life Trial Deception	Real-life Trial Deception Detection Dataset and	True	True	1680	Vocal	INTERSPEECH 2009, INTERSPEECH 2013, INTERSPEECH 2016

		Level Machine Learning Model [Автоматическое определение ложной и истинной паралингвистической информации в речи человека с применением двухуровневой модели машинного обучения]		Detection Dataset and Deceptive Speech Database	Deceptive Speech Database					
**74**	2021	Deception Detection and Remote Physiological Monitoring: A Dataset and Baseline Experimental Results	Speth, Jeremy, Vance, Nathan, Czajka, Adam, Bowyer, Kevin W., Wright, Diane, Flynn, Patrick	Deception Detection and Physiological Monitoring Dataset	An interview scenario in which the interviewee attempts to deceive the interviewer on selected responses	False	True	1680	Physiological + Visual	Eye saccades, Facial micro-expressions, Head pose, Heart rate, Thermal video
**80**	2021	Deception detection in text and its relation to the cultural dimension of individualism/collectivism	Papantoniou, K., Papadakos, P., Patkos, T., Flouris, G., Androutsopoulos, I., Plexousakis, D.	Author's collected data	Eleven multidomain and multicultural datasets.	False	False	7024	Demographical + Textual	BERT embeddings, Culture, Language, Lexical measures, N-grams, Phonemes, Pronouns, Relativity, Sentiment, Syntax complexity
**76**	2021	Deception in the eyes of deceiver: A computer vision and machine learning based automated deception detection	Khan, Wasiq, Hussain, Abir, Crockett, Keeley, OShea, James, Khan, Bilal M.	Author's collected data	Video recordings of true and false declarations on an interview taken from volunteers.	False	False	255026	Demographical + Visual	Ethnicity, Eye saccades, Facial micro-expressions, Gender
**79**	2021	Detecting Lies is a Child (Robot)'s Play: Gaze-Based Lie Detection in HRI	Gonzalez-Billandon, J., Sciutti, A., Sandini, G., Rea, F., Pasquali, D., Aroyo, A.M.	Author's collected data	Pupil size measures taken during a card game with a iCub robot.	False	False	37	Visual	Pupil size
**69**	2021	Development of Spectral Speech Features for Deception Detection Using Neural Networks	Ullah, Muhammad S., Fernandes, Sinead V.	Author's collected data	Three sessions of a police interrogation on a suspect.	False	True	12	Vocal	Bark
**77**	2021	How humans impair automated deception detection performance	Kleinberg, Bennett, Verschuere, Bruno	Author's collected data	True and deceptive statements collected by a web application from volunteers.	False	False	1640	Textual	LIWC categories, POS tags
**68**	2021	Identity Unbiased Deception Detection by 2D-to-3D Face Reconstruction	Ngô, Lê Minh, Wang, Wei, Mandira, Burak, Karaoğlu, Sezer, Bouma, Henri, Dibeklioğlu, Hamdi, Gevers, Theo	Real-life Trial Deception Detection Dataset	Real-life Trial Deception Detection Dataset	True	True	121	Demographical + Visual	Age, Face image, Gender
**75**	2021	LieNet: A Deep Convolution Neural Networks Framework for Detecting Deception	Karnati, Mohan, Seal, Ayan, Yazidi, Anis, Krejcar, Ondrej	Combination of three deception detection datasets	Real-life Trial Deception Detection Dataset, Bag-of-lies and Miami University Deception Detection Database	True	False	766	Physiological +Visual + Vocal	EEG channels, Face image, Voice signal
**73**	2021	Multimodal Political Deception Detection	Abouelenien, Mohamed, Hessler, Christian, Kamboj, Manvi, Asnani, Priyanka, Riani, Kais	Author's collected data	Videos with political debates with deceptions checked by PolitiFact.org	True	True	180	Textual + Visual + Vocal	Eye gaze, Facial emotion, Facial expressions, GloVe embeddings, Head pose, INTERSPEECH 2009, INTERSPEECH 2013, LIWC categories, POS tags, Sentiment, Unigrams

**67**	2021	Non-invasive Deception Detection in Videos Using Machine Learning Techniques	Islam, Siam, Saha, Popin, Chowdhury, Touhidul, Sorowar, Asif, Rab, Raqeebir	Subset of features from Bag-of-lies	Bag-of-lies	True	False	325	Visual	Facial expressions
**71**	2021	Unsupervised Audio-Visual Subspace Alignment for High-Stakes Deception Detection	Mathur, Leena, Matarić, Maja J.	Subset of UR Lying Database	UR Lying Database	True	False	107	Visual + Vocal	Eye gaze, Facial expressions, Head pose, MFCC, Prosody, Statistical measures, Statistical measures, Voice quality, eGeMAPd
**70**	2021	Use of Machine Learning for Deception Detection From Spectral and Cepstral Features of Speech Signals	Ullah, Muhammad S., Fernandes, Sinead V.	Author's collected data	Three sessions of a police interrogation on a suspect.	False	True	12	Vocal	Delta cepstrum, Delta energy, Time difference cepstrum, Time difference energy
**10**	2020	Automated Deception Detection of Males and Females from Non-Verbal Facial Micro-Gestures	Crockett, K., OShea, J., Khan, W.	Author's collected data	Interview video recordings	False	False	86584	Demographical + Visual	Eye gaze, Facial micro-gestures, Gender, Head pose
**65**	2020	Building a Better Lie Detector with BERT: The Difference Between Truth and Lies	Barsever, D., Singh, S., Neftci, E.	Ott Deceptive Opinion Spam Corpus	Ott Deceptive Opinion Spam Corpus	True	True	1600	Textual	BERT embeddings
**42**	2020	Emotion Transformation Feature: Novel Feature For Deception Detection In Videos	Yang, J. -T., Liu, G. -M., Huang, S. C. . -H.	Superset of Real-life Trial Deception Detection Dataset	Real-life Trial Deception Detection Dataset	True	True	190	Emotional + Visual	Emotion Transformation, Eye gaze, Facial expressions, Hand motion
**26**	2020	Introducing Representations of Facial Affect in Automated Multimodal Deception Detection	Mathur, Leena, Mataric, Maja J.	Real-life Trial Deception Detection Dataset	Real-life Trial Deception Detection Dataset	True	True	121	Textual + Visual + Vocal	Eye gaze, Facial affect, Facial expressions, Head motion, LIWC categories, MFCC, Spectral parameters, Voice pitch, Voice quality
**20**	2020	Multilingual Deception Detection by Autonomous Agents	Azaria, Amos, Hershkovitch Neiterman, Evgeny, Bitan, Moshe	Author's collected data	Computer-based card game played by volunteers	False	False	637	Demographical + Vocal	Native language, Voice spectrogram
**43**	2 020	Multimodal Deception Detection using Real-Life Trial Data	Mihalcea, R., Abouelenien, M., Burzo, M., Sen, U. M., Perez-Rosas, V., Yanikoglu, B.	Subset of Real-life Trial Deception Detection Dataset	Real-life Trial Deception Detection Dataset	True	True	59	Demographical +Textual + Visual + Vocal	Eye gaze, Facial expressions, Gender, Hand motion, Head motion, LIWC categories, Silence gaps, Unigrams, Voice histogram, Voice pitch
**11**	2020	Your eyes never lie: A robot magician can tell if you are lying	Gonzalez-Billandon, J., Sciutti, A., Sandini, G., Rea, F., Pasquali, D., Aroyo, A.M.	Author's collected data	Card discovery game played by volunteers	False	False	126	Visual	Pupil dilation, Pupil dilation latency
**27**	2019	Automatic Deception Detection in RGB Videos Using Facial Action Units	Avola, Danilo, Cinque, Luigi, Foresti, Gian Luca, Pannone, Daniele	Real-life Trial Deception Detection Dataset	Real-life Trial Deception Detection Dataset	True	True	121	Visual	Eye gaze, Facial expressions, Head motion
**49**	2019	Automatic Long-Term Deception Detection in Group Interaction Videos	Bai, C., Wu, Z., Singh, B., Burgoon, J., Bolonkin, M., Chen, C., Dunbar, N., Subrahmanian, V. S.	Author's collected data	Online Resistance game	False	False	285	Visual + Vocal	Eye gaze, Facial expressions, Head motion, MFCC

**55**	2019	Bag-of-Lies: A Multimodal Dataset for Deception Detection	Gupta, V., Agarwal, M., Arora, M., Chakraborty, T., Singh, R., Vatsa, M.	Bag-of-lies	Picture description game envolving volunteers	True	False	325	Physiological + Visual + Vocal	EEG channels, Eye blinks, Eye gaze, MFCC, Pupil size, Spectral parameters, Zero-crossing
**0**	2019	Can a Robot Catch You Lying? A Machine Learning System to Detect Lies During Interactions	Gonzalez-Billandon, Jonas, Aroyo, Alexander M., Tonelli, Alessia, Pasquali, Dario, Sciutti, Alessandra, Gori, Monica, Sandini, Giulio, Rea, Francesco	Author's collected data	Questionnaire answered by volunteers	False	False	1054	Psychological + Visual	Eloquence, Eye blinks, Eye gaze, Histronic, NARS, NEO-FFI scores, Narcisistic Machiavellianism, Pupil dilation, Response time
**1**	2019	Detecting Concealed Information in Text and Speech	Hu, Shengli	Author's collected data	Blind wine-tasting game	False	False	12392	Demographical + Textual + Vocal	Gender, GloVe embeddings, INTERSPEECH 2009, LIWC categories, N-grams, POS tags, Syntax complexity, Voice energy, Voice pitch, Voice quality
**58**	2019	Detecting Deception in Political Debates Using Acoustic and Textual Features	Kopev, D., Ali, A., Koychev, I., Nakov, P.	Author's collected data	Political claims existing in the CT-FCC-18 corpus	False	True	286	Demographical + Textual + Vocal	BERT embeddings, Claim author, INTERSPEECH 2013, LIWC categories, N-grams, TF-IDF, i-vector features
**57**	2019	Face-Focused Cross-Stream Network for Deception Detection in Videos	Ding, M., Zhao, A., Lu, Z., Xiang, T., Wen, J.	Real-life Trial Deception Detection Dataset	Real-life Trial Deception Detection Dataset	True	True	121	Textual + Visual + Vocal	Facial expressions, Head motion, Spectral parameters, Unigrams
**45**	2 019	High-Level Features for Multimodal Deception Detection in Videos	Rill-García, R., Escalante, H. J., Villaseñor-Pineda, L., Reyes-Meza, V.	Real-life Trial Deception Detection Dataset	Real-life Trial Deception Detection Dataset	True	True	121	Textual + Visual + Vocal	Eye gaze, Facial expressions, Head pose, LIWC categories, MFCC, N-grams, POS tags, Syntax complexity, Voice pitch
**12**	2019	How smart your smartphone is in lie detection?	Mizanur Rahman, Md., Shome, A., Chellappan, S., Alim Al Islam, A.B.M.	Author's collected data	Questionnaire answered by recruts	False	False	121	Emotional	Hand shaking
**13**	2019	Improved semi-supervised autoencoder for deception detection	Fu, H., Lei, P., Tao, H., Zhao, L., Yang, J.	Author's collected data	Video recordings of Werewolf online game	True	False	987	Vocal	INTERSPEECH 2009
**47**	2019	Joint Learning of Conversational Temporal Dynamics and Acoustic Features for Speech Deception Detection in Dialog Games	Chou, H., Liu, Y., Lee, C.	Daily Deceptive Dialogues Corpus of Mandarin	Daily Deceptive Dialogues Corpus of Mandarin	False	False	7504	Dynamical + Vocal	Hesitation duration, MFCC, Silence count, Spectral parameters, Turn duration, Utterance duration, Voice energy, Voice pitch, Zero-crossing
**37**	2019	Robust Algorithm for Multimodal Deception Detection	Venkatesh, S., Ramachandra, R., Bours, P.	Real-life Trial Deception Detection Dataset	Real-life Trial Deception Detection Dataset	False	True	121	Textual + Visual + Vocal	Body motion, Facial micro-expressions, MFCC, N-grams
**38**	2019	Speech Deception Detection Algorithm Based on SVM and Acoustic Features	Fu, H., Lei, P., Tao, H., Wang, M., Wang, J.	KWOLF	Extracted from Werewolf online game sessions	False	False	388	Vocal	MFCC, Voice energy, Voice pitch, Zero-crossing

**5**	2018	A Multi-View Learning Approach To Deception Detection	Carissimi, Nicolo, Beyan, Cigdem, Murino, Vittorio	Superset of Real-life Trial Deception Detection Dataset	Real-life Trial Deception Detection Dataset	True	False	121	Textual + Visual	Bigrams, Eye gaze, Facial expressions, Hand motion, Head motion, Unigrams
**4**	2018	Acoustic-Prosodic Indicators of Deception and Trust in Interview Dialogues	Levitan, Sarah Ita, Maredia, Angel, Hirschberg, Julia	Columbia X-Cultural Dataset	Columbia X-Cultural Dataset	False	False	49106	Demographical + Vocal	Gender, Native language, Voice energy, Voice pitch, Voice quality
**36**	2018	An Empirical Study on Detecting Deception and Cybercrime Using Artificial Neural Networks	Mbaziira, Alex V., Murphy, Diane R.	Author's collected data	Combination of four publicly available dataset	False	True	300	Textual	Syntax complexity
**2**	2018	Automated verbal credibility assessment of intentions: The model statement technique and predictive modeling	Kleinberg, Bennett, van der Toolen, Yaloe, Vrij, Aldert, Arntz, Arnoud, Verschuere, Bruno	Author's collected data	Interviews on weekand plans collected from volunteers	False	False	147	Textual	LIWC categories, Named entities, Psychological processes
**3**	2018	Comparative Analysis of Classification Methods for Automatic Deception Detection in Speech	Velichko, Alena, Budkov, Viktor, Kagirov, Ildar, Karpov, Alexey	Superset of Real-life Trial Deception Detection Dataset	Real-life Trial Deception Detection Dataset	True	True	195	Vocal	INTERSPEECH 2013
**62**	2018	Construction of a Liar Corpus and Detection of Lying Situations	Takabatake, S., Shimada, K., Saitoh, T.	Author's collected data	Computer-based question-answer interview	False	False	540	Visual	Facial micro-expressions
**48**	2018	Convolutional Bidirectional Long Short-Term Memory for Deception Detection With Acoustic Features	Tao, H., Zhao, L., Xie, Y., Liang, R., Zhu, Y.	Author's collected data	Question-answer experiment answered by selected participants	False	False	7867	Vocal	Spectral parameters, Voice pitch
**53**	2018	Deception Detection and Analysis in Spoken Dialogues based on FastText	Hosomi, N., Sakti, S., Yoshino, K., Nakamura, S.	CSC Deceptive Speech	CSC Deceptive Speech	False	False	4100	Textual + Vocal	FastText embedding, INTERSPEECH 2009
**14**	2018	Deception detection in videos	Subrahmanian, V.S., Wu, Z., Singh, B., Davis, L.S.	Subset of Real-life Trial Deception Detection Dataset	Real-life Trial Deception Detection Dataset	True	True	104	Textual + Visual + Vocal	Facial micro-expressions, GloVe embeddings, MFCC
**40**	2018	Deception detection using artificial neural network and support vector machine	Srivastava, N., Dubey, S.	Author's collected data	15-question questionnaire answered by 50 people	False	False	750	Physiological + Vocal	Blood pressure, Heart rate, MFCC, Respiration rate, Voice energy, Voice pitch, Zero-crossing
**59**	2018	Detection of Deception Using Facial Expressions Based on Different Classification Algorithms	Thannoon, H. H., Ali, W. H., Hashim, I. A.	Author's collected data	Questionnaire answered by volunteers	False	False	448	Demographical + Visual	Facial expressions, Gender
**44**	2018	Intelligent Deception Detection through Machine Based Interviewing	Crockett, K., Khan, W., O'Shea, J., Kindynis, P., Antoniades, A., Boultadakis, G.	Author's collected data	Questionnaire answered by volunteers	False	False	400	Demographical +Visual	Ethnicity, Eye gaze, Facial expressions, Gender, Head pose
**22**	2018	Interpretable Multimodal Deception Detection in Videos	Karimi, Hamid	Real-life Trial Deception Detection Dataset	Real-life Trial Deception Detection Dataset	True	True	121	Visual + Vocal	Facial expressions, INTERSPEECH 2009, INTERSPEECH 2013
**63**	2018	Lie Detector With The Analysis Of The Change Of Diameter Pupil and The Eye Movement Use Method Gabor Wavelet Transform and Decision Tree	Labibah, Z., Nasrun, M., Setianingsih, C.	Author's collected data	Questionnaire answered by volunteers	False	False	40	Visual	Eye gaze, Pupil dilation

**15**	2018	Linguistic cues to deception and perceived deception in interview dialogues	Levitan, S.I., Maredia, A., Hirschberg, J.	Columbia X-Cultural Dataset	Columbia X-Cultural Dataset	False	False	4056	Demographical + Psychological + Textual	Gender, LIWC categories, Lexical measures, NEO-FFI scores, Native language, Pauses, Syntax complexity
**46**	2018	Toward End-to-End Deception Detection in Videos	Karimi, H., Tang, J., Li, Y.	Real-life Trial Deception Detection Dataset	Real-life Trial Deception Detection Dataset	True	True	121	Visual + Vocal	Facial expressions, INTERSPEECH 2009, INTERSPEECH 2013
**8**	2017	Construction and Analysis of Indonesian-Interviews Deception Corpus	Warnita, Tifani, Lestari, Dessi Puji	Indonesian Deception Corpus	Interviews on six themes with volunteers	False	False	5542	Textual + Vocal	INTERSPEECH 2010, LIWC categories, Silence gaps, Voice energy, Voice pitch
**16**	2017	Deception detection in Russian texts	Litvinova, O., Litvinova, T., Seredin, P., Lyell, J.	Russian Deception Bank	Texts written by volunteers	False	False	226	Demographical + Textual	Age, Education, Gender, LIWC categories, POS tags
**6**	2017	Deep Learning Driven Multimodal Fusion For Automated Deception Detection	Gogate, Mandar, Adeel, Ahsan, Hussain, Amir	Superset of Real-life Trial Deception Detection Dataset	Real-life Trial Deception Detection Dataset	True	True	121	Textual + Visual + Vocal	Facial expressions, GloVe embeddings, Hand motion, INTERSPEECH 2013
**54**	2017	Detecting Deceptive Behavior via Integration of Discriminative Features From Multiple Modalities	Pérez-Rosas, V., Mihalcea, R., Abouelenien, M., Burzo, M.	Author's collected data	Mock crime game and a questionnaire about two sensitive themes answered by 30 volunteers	False	False	149	Physiological + Textual + Thermal	Blood volume, Cheeks, Forehead, Heart rate, LIWC categories, Nose, POS tags, Periorbital region, Respiration volume, Skin conductance, Syntax complexity, TF-IDF
**21**	2017	Gender-Based Multimodal Deception Detection	Perez-Rosas, Veronica, Mihalcea, Rada, Abouelenien, Mohamed, Zhao, Bohan, Burzo, Mihai	Author's collected data	Mock crime game and a questionnaire about two sensitive themes answered by 104 volunteers	False	False	520	Demographical + Physiological + Textual + Thermal	Face region, Gender, Heart rate, LIWC categories, POS tags, Respiration rate, Skin conductance, Syntax complexity, Unigrams
**7**	2 017	Hybrid Acoustic-Lexical Deep Learning Approach for Deception Detection	Levitan, Sarah Ita, Hirschberg, Julia, Mendels, Gideon, Lee, Kai-Zhan	Columbia X-Cultural Dataset	Columbia X-Cultural Dataset	False	False	49106	Textual + Vocal	GloVe embeddings, INTERSPEECH 2009, INTERSPEECH 2013, MFCC, N-grams
**28**	2016	Analyzing Thermal and Visual Clues of Deception for a Non-Contact Deception Detection Approach	Mihalcea, Rada, Abouelenien, Mohamed, Burzo, Mihai	Author's collected data	A trivia game answered by volunteers	False	False	149	Thermal + Visual	Entire face, Eye blinks, Facial expressions, Head motion
**66**	2016	Automated detection of user deception in on-line questionnaires with focus on eye tracking use	Rybar, M., Bielikova, M.	Author's collected data	Gaze and pupil data collected during questionnaire answering by volunteers	False	False	50	Visual	Eye saccades, Pupil dilation, Response time
**60**	2016	Deceptive Speech Detection based on sparse representation	Fan, Cheng, Zhao, Heming, Chen, Xueqin, Fan, Xiaohe, Chen, Shuxi	Soochow Deceptive Speech Detection Corpus	Questionnaires answered by forty recruited native Chinese speakers	False	False	4143	Demographical + Vocal	Gender, MFCC, Spectral parameters, Zero-crossing
**41**	2016	ReLiDSS: Novel lie detection system from speech signal	Nasri, H., Ouarda, W., Alimi, A. M.	ReliDDB	Interviewing game answered by volunteers	False	False	137640	Vocal	MFCC, Voice pitch

**52**	2016	The Truth and Nothing But the Truth: Multimodal Analysis for Deception Detection	Jaiswal, M., Tabibu, S., Bajpai, R.	Subset of Real-life Trial Deception Detection Dataset	Real-life Trial Deception Detection Dataset	False	True	100	Textual + Visual + Vocal	Facial expressions, MFCC, POS tags, Prosody, Sentiment, Unigrams, Voice energy
**39**	2015	A comparison of features for automatic deception detection in synchronous computer-mediated communication	Pak, J., Zhou, L.	Author's collected data	Communications during sessions of the online mafia game	False	False	142	Textual	LIWC categories, Syntax complexity, Unigrams
**29**	2015	Cross-Cultural Production and Detection of Deception from Speech	Hirschberg, Julia, Mendels, Gideon, Levitan, Sarah I., An, Guzhen, Wang, Mandi, Levine, Michelle, Rosenberg, Andrew	Author's collected data	Statements provided by volunteers in a mock fake resume game	False	False	154	Demographical + Psychological + Vocal	Ethnicity, Gender, NEO-FFI scores, Native language, Speaking rate, Voice energy, Voice pitch, Voice quality
**24**	2015	Deception Detection Using Real-Life Trial Data	Perez-Rosas, Veronica, Mihalcea, Rada, Abouelenien, Mohamed, Burzo, Mihai	Real-life Trial Deception Detection Dataset	Real-life Trial Deception Detection Dataset	True	True	121	Textual + Visual	Bigrams, Body motion, Facial expressions, Head motion, Unigrams
**35**	2015	Detection of Deception in the Mafia Party Game	Demyanov, Sergey, Bailey, James, Ramamohanarao, Kotagiri, Leckie, Christopher	Mafia DB	Video recordings of the Russian Mafia TV Show	False	True	6733	Visual	Facial expressions
**9**	2015	Distinguishing Deception from Non-Deception in Chinese Speech	Fan, Cheng, Zhao, Heming, Chen, Xueqin, Fan, Xiaohe, Chen, Shuxi	Soochow Deceptive Speech Detection Corpus	Recordings taken from volunteers in a story-telling game	False	False	3787	Demographical + Vocal	Duration, Formant, Gender, Voice energy, Voice pitch
**17**	2015	Experiments in open domain deception detection	Pérez-Rosas, V., Mihalcea, R.	Author's collected data	Open domain sentences collected from volunteers	True	False	7168	Demographical + Textual	Age, Education, Gender, LIWC categories, Nationality, POS tags, Syntax complexity, Unigrams
**56**	2015	Is Interactional Dissynchrony a Clue to Deception? Insights From Automated Analysis of Nonverbal Visual Cues	Burgoon, J. K., Yu, X., Zhang, S., Yan, Z., Yang, F., Huang, J., Dunbar, N. E., Jensen, M. L., Metaxas, D. N.	Author's collected data	From a trivia game with volunteers	False	False	100	Visual	Facial expressions, Head motion, Interactional synchrony
**61**	2015	Perinasal indicators of deceptive behavior	Burgoon, J. K., Dcosta, M., Shastri, D., Vilalta, R., Pavlidis, I.	Author's collected data	From a mock crime game answered by volunteers	False	False	40	Thermal	Perinasal region
**64**	2014	Cues to Deception in Social Media Communications	Briscoe, E. J., Appling, D. S., Hayes, H.	Author's collected data	Statements provided by volunteers in a mock chat room	False	False	254	Textual	Emoticons, Informality, Sentiment, Syntax complexity
**23**	2014	Deception Detection Using a Multimodal Approach	Perez-Rosas, Veronica, Mihalcea, Rada, Abouelenien, Mohamed, Burzo, Mihai	Author's collected data	Statements from volunteers in an opinion game	False	False	120	Physiological + Textual + Thermal	Blood volume, Entire face, LIWC categories, Respiration volume, Skin conductance, Unigrams
**50**	2014	Thermal Facial Analysis for Deception Detection	Rajoub, B. A., Zwiggelaar, R.	Author's collected data	Statements from volunteers in an interview game	False	False	492	Thermal	Periorbital region

**32**	2013	Automatic Detection of Deceit in Verbal Communication	Perez-Rosas, Veronica, Mihalcea, Rada, Burzo, Mihai	Author's collected data	Video recordings from volunteers	False	False	140	Textual	Unigrams
**51**	2013	Deception detection in speech using bark band and perceptually significant energy features	Sanaullah, M., Gopalan, K.	Author's collected data	Utterances from recordings of police interrogations	False	True	6	Vocal	Bark, Significant energy
**18**	2012	Discerning truth from deception: Human judgments and automation efforts	Rubin, V.L., Conroy, N.	Author's collected data	Stories written by volunteers	False	False	90	Textual	LIWC categories, Lexical measures
**33**	2012	On the Use of Homogenous Sets of Subjects in Deceptive Language Analysis	Fornaciari, Tommaso, Poesio, Massimo	DeCour corpus	Utterances extracted from statements in real case transcriptions	False	True	3015	Textual	LIWC categories, Lexical measures, N-grams, POS tags
**31**	2012	Seeing through Deception: A Computational Approach to Deceit Detection in Written Communication	Almela, Angela, Valencia-Garcia, Rafael, Cantos, Pascual	Author's collected data	Opinions from volunteers on three themes	False	False	600	Textual	LIWC categories
**25**	2012	Syntactic Stylometry for Deception Detection	Feng, Song, Banerjee, Ritwik, Choi, Yejin	Author's collected data	Reviews of 35 Italian restaurants	False	False	2692	Textual	Bigrams, POS tags, Syntax complexity, Unigrams
**30**	2012	The Voice and Eye Gaze Behavior of an Imposter: Automated Interviewing and Detection for Rapid Screening at the Border	Elkins, Aaron C., Derrick, Douglas C., Gariup, Monica	Author's collected data	Participants of an experiment in UE border control	False	False	259	Thermal + Vocal	Eye gaze, Pupil dilation, Voice energy, Voice pitch, Voice quality
**19**	2011	Challenges in automated deception detection in computer-mediated communication	Conroy, N.J., Rubin, V.L.	Author's collected data	Stories written by volunteers	False	False	90	Textual	LIWC categories, Lexical measures
**34**	2011	Move, and i Will Tell You Who You Are: Detecting Deceptive Roles in Low-Quality Data	Raiman, Nimrod, Hung, Hayley, Englebienne, Gwenn	Wolf-database	Recordings of players of RPG Werewolf game	False	False	72	Visual + Vocal	Body motion, Non-silent

Summary of Real-life Trial Deception Detection Dataset used in studies in the selected corpus

	year	Paper	Author	Cardinality	Modality combination	Features
**72**	2021	Affect-Aware Deep Belief Network Representations for Multimodal Unsupervised Deception Detection	Mathur, Leena, Matarić, Maja J	108	Visual + Vocal	Eye gaze, Facial arousal, Facial expressions, Facial valence, Head pose, MFCC, Prosody, Spectral parameters, Voice energy, Voice quality
**68**	2021	Identity Unbiased Deception Detection by 2D-to-3D Face Reconstruction	Ngô, Lê Minh, Wang, Wei, Mandira, Burak, Karaoğlu, Sezer, Bouma, Henri, Dibeklioğlu, Hamdi, Gevers, Theo	121	Demographical + Visual	Age, Face image, Gender
**42**	2020	Emotion Transformation Feature: Novel Feature For Deception Detection In Videos	Yang, J. -T., Liu, G. -M., Huang, S. C. . -H.	190	Emotional + Visual	Emotion Transformation, Eye gaze, Facial expressions, Hand motion
**26**	2020	Introducing Representations of Facial Affect in Automated Multimodal Deception Detection	Mathur, Leena, Mataric, Maja J.	121	Textual + Visual + Vocal	Eye gaze, Facial affect, Facial expressions, Head motion, LIWC categories, MFCC, Spectral parameters, Voice pitch, Voice quality
**43**	2020	Multimodal Deception Detection using Real-Life Trial Data	Mihalcea, R., Abouelenien, M., Burzo, M., Sen, U. M., Perez-Rosas, V., Yanikoglu, B.	59	Demographical + Textual + Visual + Vocal	Eye gaze, Facial expressions, Gender, Hand motion, Head motion, LIWC categories, Silence gaps, Unigrams, Voice histogram, Voice pitch
**27**	2019	Automatic Deception Detection in RGB Videos Using Facial Action Units	Avola, Danilo, Cinque, Luigi, Foresti, Gian Luca, Pannone, Daniele	121	Visual	Eye gaze, Facial expressions, Head motion
**57**	2019	Face-Focused Cross-Stream Network for Deception Detection in Videos	Ding, M., Zhao, A., Lu, Z., Xiang, T., Wen, J.	121	Textual + Visual + Vocal	Facial expressions, Head motion, Spectral parameters, Unigrams
**45**	2019	High-Level Features for Multimodal Deception Detection in Videos	Rill-García, R., Escalante, H. J., Villaseñor-Pineda, L., Reyes-Meza, V.	121	Textual + Visual + Vocal	Eye gaze, Facial expressions, Head pose, LIWC categories, MFCC, N-grams, POS tags, Syntax complexity, Voice pitch

**37**	2019	Robust Algorithm for Multimodal Deception Detection	Venkatesh, S., Ramachandra, R., Bours, P.	121	Textual + Visual + Vocal	Body motion, Facial micro-expressions, MFCC, N-grams
**5**	2018	A Multi-View Learning Approach To Deception Detection	Carissimi, Nicolo, Beyan, Cigdem, Murino, Vittorio	121	Textual + Visual	Bigrams, Eye gaze, Facial expressions, Hand motion, Head motion, Unigrams
**3**	2018	Comparative Analysis of Classification Methods for Automatic Deception Detection in Speech	Velichko, Alena, Budkov, Viktor, Kagirov, Ildar, Karpov, Alexey	195	Vocal	INTERSPEECH 2013
**14**	2018	Deception detection in videos	Subrahmanian, V.S., Wu, Z., Singh, B., Davis, L.S.	104	Textual + Visual + Vocal	Facial micro-expressions, GloVe embeddings, MFCC
**22**	2018	Interpretable Multimodal Deception Detection in Videos	Karimi, Hamid	121	Visual + Vocal	Facial expressions, INTERSPEECH 2009, INTERSPEECH 2013
**46**	2018	Toward End-to-End Deception Detection in Videos	Karimi, H., Tang, J., Li, Y.	121	Visual + Vocal	Facial expressions, INTERSPEECH 2009, INTERSPEECH 2013
**6**	2017	Deep Learning Driven Multimodal Fusion For Automated Deception Detection	Gogate, Mandar, Adeel, Ahsan, Hussain, Amir	121	Textual + Visual + Vocal	Facial expressions, GloVe embeddings, Hand motion, INTERSPEECH 2013
**52**	2016	The Truth and Nothing But the Truth: Multimodal Analysis for Deception Detection	Jaiswal, M., Tabibu, S., Bajpai, R.	100	Textual + Visual + Vocal	Facial expressions, MFCC, POS tags, Prosody, Sentiment, Unigrams, Voice energy
**24**	2015	Deception Detection Using Real-Life Trial Data	Perez-Rosas, Veronica, Mihalcea, Rada, Abouelenien, Mohamed, Burzo, Mihai	121	Textual + Visual	Bigrams, Body motion, Facial expressions, Head motion, Unigrams

Summary of Columbia X-Cultural Dataset used in studies in the selected corpus

	year	Paper	Author	Cardinality	Modality combination	Features
**4**	2018	Acoustic-Prosodic Indicators of Deception and Trust in Interview Dialogues	Levitan, Sarah Ita, Maredia, Angel, Hirschberg, Julia	49106	Demographical + Vocal	Gender, Native language, Voice energy, Voice pitch, Voice quality
**15**	2018	Linguistic cues to deception and perceived deception in interview dialogues	Levitan, S.I., Maredia, A., Hirschberg, J.	4056	Demographical + Psychological + Textual	Gender, LIWC categories, Lexical measures, NEO-FFI scores, Native language, Pauses, Syntax complexity
**7**	2017	Hybrid Acoustic-Lexical Deep Learning Approach for Deception Detection	Levitan, Sarah Ita, Hirschberg, Julia, Mendels, Gideon, Lee, Kai-Zhan	49106	Textual + Vocal	GloVe embeddings, INTERSPEECH 2009, INTERSPEECH 2013, MFCC, N-grams

Summary of Soochow Deceptive Speech Detection Corpus used in studies in the selected corpus

	year	Paper	Author	Cardinality	Modality combination	Features
**60**	2016	Deceptive Speech Detection based on sparse representation	Fan, Cheng, Zhao, Heming, Chen, Xueqin, Fan, Xiaohe, Chen, Shuxi	4143	Demographical + Vocal	Gender, MFCC, Spectral parameters, Zero-crossing
**9**	2015	Distinguishing Deception from Non-Deception in Chinese Speech	Fan, Cheng, Zhao, Heming, Chen, Xueqin, Fan, Xiaohe, Chen, Shuxi	3787	Demographical + Vocal	Duration, Formant, Gender, Voice energy, Voice pitch

Summary of dataset features used in studies in the selected corpus

	Name	Origin	Public	Real-life	Cardinality	Demographical	Dynamical	Emotional	Physiological	Psychological	Textual	Thermal	Visual	Vocal	Algorithms
**79**	None	Pupil size measures taken during a card game with a iCub robot.	False	False	37	0	0	0	0	0	0	0	1	0	Random Forest
**61**	None	From a mock crime game	False	False	40	0	0	0	0	0	0	9	0	0	AdaBoost, Decision Tree,

		answered by volunteers													Naïve Bayes, Neural Network
**63**	None	Questionnaire answered by volunteers	False	False	40	0	0	0	0	0	0	0	2	0	Decision Tree
**66**	None	Gaze and pupil data collected during questionnaire answering by volunteers	False	False	50	0	0	0	0	0	0	0	6	0	SVM
**34**	Wolf-database	Recordings of players of RPG Werewolf game	False	False	72	0	0	0	0	0	0	0	6	1	RVM, SVM
**18**	None	Stories written by volunteers	False	False	90	0	0	0	0	0	13	0	0	0	Decision Tree, SMO
**19**	None	Stories written by volunteers	False	False	90	0	0	0	0	0	13	0	0	0	Decision Tree, SMO
**56**	None	From a trivia game with volunteers	False	False	100	0	0	0	0	0	0	0	Uninformed	0	SVM
**71**	Subset of UR Lying Database	UR Lying Database	True	False	107	0	0	0	0	0	0	0	372	696	KNN
**23**	None	Statements from volunteers in an opinion game	False	False	120	0	0	0	60	0	214	124	0	0	Decision Tree
**5**	Superset of Real-life Trial Deception Detection Dataset	Real-life Trial Deception Detection Dataset	True	False	121	0	0	0	0	0	1609	0	4152	0	Multi-view Learning
**12**	None	Questionnaire answered by recruts	False	False	121	0	0	5	0	0	0	0	0	0	Decision Tree, KNN, KStar, Random Committee, Random Forest
**11**	None	Card discovery game played by volunteers	False	False	126	0	0	0	0	0	0	0	13	0	Random Forest

**32**	None	Video recordings from volunteers	False	False	140	0	0	0	0	0	Uninformed	0	0	0	Naïve Bayes, SVM
**39**	None	Communications during sessions of the online mafia game	False	False	142	0	0	0	0	0	Uninformed	0	0	0	AdaBoost, Decision Tree, JRip, KNN, Naïve Bayes, Neural Network, SVM
**2**	None	Interviews on weekand plans collected from volunteers	False	False	147	0	0	0	0	0	132	0	0	0	SVM
**28**	None	A trivia game answered by volunteers	False	False	149	0	0	0	0	0	0	107	42	0	Decision Tree
**54**	None	Mock crime game and a questionnaire about two sensitive themes answered by 30 volunteers	False	False	149	0	0	0	60	0	6262	8	0	0	Decision Tree
**29**	None	Statements provided by volunteers in a mock fake resume game	False	False	154	3	0	0	0	5	0	0	0	14	Bagging, Decision Tree, Random Forest
**16**	Russian Deception Bank	Texts written by volunteers	False	False	226	3	0	0	0	0	104	0	0	0	Clustering
**64**	None	Statements provided by volunteers in a mock chat room	False	False	254	0	0	0	0	0	14	0	0	0	Gradient Boosting, Neural Network, Random Forest, SVM
**30**	None	Participants of an experiment in UE border control	False	False	259	0	0	0	0	0	0	2	0	6	Decision Tree
**49**	None	Online Resistance game	False	False	285	0	0	0	0	0	0	0	Uninformed	Uninformed	Combined methods
**55**	Bag-of-lies	Picture description game envolving volunteers	True	False	325	0	0	0	1000	0	0	0	64	26	Combined methods

**67**	Subset of features from Bag-of-lies	Bag-of-lies	True	False	325	0	0	0	0	0	0	0	5	0	Decision Tree, KNN, Logistic Regression, Random Forest, SVM
**38**	KWOLF	Extracted from Werewolf online game sessions	False	False	388	0	0	0	0	0	0	0	0	Uninformed	SVM
**44**	None	Questionnaire answered by volunteers	False	False	400	2	0	0	0	0	0	0	38	0	Neural Network
**59**	None	Questionnaire answered by volunteers	False	False	448	1	0	0	0	0	0	0	1606	0	KNN, Neural Network, SVM
**50**	None	Statements from volunteers in an interview game	False	False	492	0	0	0	0	0	0	102	0	0	KNN
**21**	None	Mock crime game and a questionnaire about two sensitive themes answered by 104 volunteers	False	False	520	1	0	0	59	0	Uninformed	20	0	0	Decision Tree
**62**	None	Computer-based question-answer interview	False	False	540	0	0	0	0	0	0	0	5	0	SVM
**31**	None	Opinions from volunteers on three themes	False	False	600	0	0	0	0	0	27	0	0	0	SVM
**20**	None	Computer-based card game played by volunteers	False	False	637	1	0	0	0	0	0	0	0	Uninformed	Neural Network
**40**	None	15-question questionnaire answered by 50 people	False	False	750	0	0	0	3	0	0	0	0	Uninformed	Neural Network, SVM
**75**	Combination of three deception detection datasets	Real-life Trial Deception Detection Dataset, Bag-of-lies and Miami University	True	False	766	0	0	0	13	0	0	0	Uninformed	Uninformed	Neural Network

		Deception Detection Database													
**13**	None	Video recordings of Werewolf online game	True	False	987	0	0	0	0	0	0	0	0	384	Neural Network
**0**	None	Questionnaire answered by volunteers	False	False	1054	0	0	0	0	5	0	0	5	0	Random Forest
**77**	None	True and deceptive statements collected by a web application from volunteers.	False	False	1640	0	0	0	0	0	110	0	0	0	Random Forest
**25**	None	Reviews of 35 Italian restaurants	False	False	2692	0	0	0	0	0	Uninformed	0	0	0	SVM
**9**	Soochow Deceptive Speech Detection Corpus	Recordings taken from volunteers in a story-telling game	False	False	3787	1	0	0	0	0	0	0	0	20	Decision Tree, Linear regression, Neural Network, SVM
**15**	Columbia X-Cultural Dataset	Columbia X-Cultural Dataset	False	False	4056	2	0	0	0	5	122	0	0	0	Random Forest
**53**	CSC Deceptive Speech	CSC Deceptive Speech	False	False	4100	0	0	0	0	0	30	0	0	384	Neural Network
**60**	Soochow Deceptive Speech Detection Corpus	Questionnaires answered by forty recruited native Chinese speakers	False	False	4143	1	0	0	0	0	0	0	0	25	SVM
**8**	Indonesian Deception Corpus	Interviews on six themes with volunteers	False	False	5542	0	0	0	0	0	Uninformed	0	0	1582	Random Forest
**80**	None	Eleven multidomain and multicultural datasets.	False	False	7024	2	0	0	0	0	49	0	0	0	Logistic Regression, Neural Network
**17**	None	Open domain sentences collected from volunteers	True	False	7168	4	0	0	0	0	96	0	0	0	SVM
**47**	Daily Deceptive Dialogues	Daily Deceptive Dialogues	False	False	7504	0	20	0	0	0	0	0	0	988	Neural Network

	Corpus of Mandarin	Corpus of Mandarin													
**48**	None	Question-answer experiment answered by selected participants	False	False	7867	0	0	0	0	0	0	0	0	60	Neural Network
**1**	None	Blind wine-tasting game	False	False	12392	1	0	0	0	0	103	0	0	6778	Logistic Regression, Neural Network, Random Forest
**4**	Columbia X-Cultural Dataset	Columbia X-Cultural Dataset	False	False	49106	2	0	0	0	0	0	0	0	8	Random Forest
**7**	Columbia X-Cultural Dataset	Columbia X-Cultural Dataset	False	False	49106	0	0	0	0	0	200	0	0	7157	Neural Network
**10**	None	Interview video recordings	False	False	86584	1	0	0	0	0	0	0	36	0	Decision Tree, Naïve Bayes, Neural Network, Random Forest, ZeroR
**41**	ReliDDB	Interviewing game answered by volunteers	False	False	137640	0	0	0	0	0	0	0	0	14	SVM
**76**	None	Video recordings of true and false declarations on an interview taken from volunteers.	False	False	255026	2	0	0	0	0	0	0	36	0	Neural Network, Random Forest, SVM
**51**	None	Utterances from recordings of police interrogations	False	True	6	0	0	0	0	0	0	0	0	42	Neural Network
**69**	None	Three sessions of a police interrogation on a suspect.	False	True	12	0	0	0	0	0	0	0	0	Uninformed	Neural Network
**70**	None	Three sessions of a police interrogation on a suspect.	False	True	12	0	0	0	0	0	0	0	0	4	Neural Network

**43**	Subset of Real-life Trial Deception Detection Dataset	Real-life Trial Deception Detection Dataset	True	True	59	1	0	0	0	0	234	0	39	104	Neural Network, Random Forest, SVM
**52**	Subset of Real-life Trial Deception Detection Dataset	Real-life Trial Deception Detection Dataset	False	True	100	0	0	0	0	0	Uninformed	0	18	28	SVM
**14**	Subset of Real-life Trial Deception Detection Dataset	Real-life Trial Deception Detection Dataset	True	True	104	0	0	0	0	0	Uninformed	0	396	Uninformed	AdaBoost, Decision Tree, Logistic Regression, Naïve Bayes, Random Forest, SVM
**72**	Subset of Real-life Trial Deception Detection Dataset	Real-life Trial Deception Detection Dataset	True	True	108	0	0	0	0	0	0	0	561	986	Neural Network
**27**	Real-life Trial Deception Detection Dataset	Real-life Trial Deception Detection Dataset	True	True	121	0	0	0	0	0	0	0	Uninformed	0	Logistic Regression, Random Forest, SVM
**24**	Real-life Trial Deception Detection Dataset	Real-life Trial Deception Detection Dataset	True	True	121	0	0	0	0	0	Uninformed	0	40	0	Decision Tree, Random Forest
**22**	Real-life Trial Deception Detection Dataset	Real-life Trial Deception Detection Dataset	True	True	121	0	0	0	0	0	0	0	18	6757	Neural Network
**37**	Real-life Trial Deception Detection Dataset	Real-life Trial Deception Detection Dataset	False	True	121	0	0	0	0	0	11906	0	39	14	AdaBoost, Combined methods, SKRDA, SVM
**68**	Real-life Trial Deception Detection Dataset	Real-life Trial Deception Detection Dataset	True	True	121	2	0	0	0	0	0	0	Uninformed	0	Neural Network

**45**	Real-life Trial Deception Detection Dataset	Real-life Trial Deception Detection Dataset	True	True	121	0	0	0	0	0	Uninformed	0	49	Uninformed	BSSD, Neural Network
**46**	Real-life Trial Deception Detection Dataset	Real-life Trial Deception Detection Dataset	True	True	121	0	0	0	0	0	0	0	18	6757	KNN
**26**	Real-life Trial Deception Detection Dataset	Real-life Trial Deception Detection Dataset	True	True	121	0	0	0	0	0	7	0	65	38	AdaBoost
**57**	Real-life Trial Deception Detection Dataset	Real-life Trial Deception Detection Dataset	True	True	121	0	0	0	0	0	300	0	256	Uninformed	Neural Network
**6**	Superset of Real-life Trial Deception Detection Dataset	Real-life Trial Deception Detection Dataset	True	True	121	0	0	0	0	0	300	0	5	6373	Neural Network
**73**	None	Videos with political debates with deceptions checked by PolitiFact.org	True	True	180	0	0	0	0	0	2255	0	179	6757	Decision Tree
**42**	Superset of Real-life Trial Deception Detection Dataset	Real-life Trial Deception Detection Dataset	True	True	190	0	0	49	0	0	0	0	Uninformed	0	Decision Tree, KNN, Random Forest, SVM
**3**	Superset of Real-life Trial Deception Detection Dataset	Real-life Trial Deception Detection Dataset	True	True	195	0	0	0	0	0	0	0	0	6373	AdaBoost, Decision Tree, KNN, OneR, PART, Random Forest, Random Tree, SGD, SMO
**58**	None	Political claims existing in the CT-FCC-18 corpus	False	True	286	1	0	0	0	0	Uninformed	0	0	62773	Neural Network
**36**	None	Combination of four publicly	False	True	300	0	0	0	0	0	27	0	0	0	KNN, Naïve Bayes,

		available dataset													Neural Network, SVM
**65**	Ott Deceptive Opinion Spam Corpus	Ott Deceptive Opinion Spam Corpus	True	True	1600	0	0	0	0	0	768	0	0	0	Neural Network
**74**	Deception Detection and Physiological Monitoring Dataset	An interview scenario in which the interviewee attempts to deceive the interviewer on selected responses	False	True	1680	0	0	0	Uninformed	0	0	0	Uninformed	0	SVM
**78**	Merging of Real-life Trial Deception Detection Dataset and Deceptive Speech Database	Real-life Trial Deception Detection Dataset and Deceptive Speech Database	True	True	1680	0	0	0	0	0	0	0	0	986	Combined methods
**33**	DeCour corpus	Utterances extracted from statements in real case transcriptions	False	True	3015	0	0	0	0	0	390	0	0	0	SVM
**35**	Mafia DB	Video recordings of the Russian Mafia TV Show	False	True	6733	0	0	0	0	0	0	0	79	0	Logistic Regression

8. Performance analysis

8.1. Performance metrics and levels

The next charts present the performance achieved by each study in the selected corpus. Studies presented the achieved performance in different measures. Most of them used **Accuracy** alone or combined with some other metric, but a few ones also used **Area Under the Curve**, **F1-score**, **Precision**, **Recall**, and **Unweighted Average Recall**.

When **Accuracy** and some other metric was used, **Accuracy** was accounted as the one for that study, since the threshold (90%) used to classify lie catcher as "Wizards of Deception Detection" by [1] is measured by accuracy.

[1] O'Sullivan M, Ekman P. The wizards of deception detection. The Detection of Deception in Forensic Contexts. 2004.

Summary of performances achieved by techniques exploited by studies in the selected corpus

	id	Year	Technique	Accuracy	Area Under the Curve	F1-score	Precision	Recall	Unweighted Average Recall	Modality cardinality	Modality combination	Dataset	Dataset cardinality	Features
**80**	675	2021	Logistic Regression	0.624000	0.620000	0.620000	0.626000	0.930000	0.000000	Bimodal	Demographical + Textual	Eleven multidomain and multicultural datasets.	7024	BERT embeddings, Culture, Language, Lexical measures, N-grams, Phonemes, Pronouns, Relativity, Sentiment, Syntax complexity
**73**	625	2021	Decision Tree	0.700000	0.000000	0.000000	0.690000	0.000000	0.000000	Multimodal	Textual + Visual + Vocal	Videos with political debates with deceptions checked by PolitiFact.org	180	Eye gaze, Facial emotion, Facial expressions, GloVe embeddings, Head pose, INTERSPEECH 2009, INTERSPEECH 2013, LIWC categories, POS tags, Sentiment, Unigrams
**67**	618	2021	SVM	0.615400	0.000000	0.628500	0.575800	0.697200	0.000000	Monomodal	Visual	Subset of features from Bag-of-lies	325	Facial expressions
**69**	621	2021	Neural Network	0.916700	0.000000	0.000000	0.000000	0.000000	0.000000	Monomodal	Vocal	Three sessions of a police interrogation on a suspect.	12	Bark
**70**	622	2021	Neural Network	1.000000	0.000000	0.000000	0.000000	0.000000	0.000000	Monomodal	Vocal	Three sessions of a police interrogation on a suspect.	12	Delta cepstrum, Delta energy, Time difference cepstrum, Time difference energy
**71**	623	2021	KNN	0.740000	0.750000	0.730000	0.000000	0.000000	0.000000	Bimodal	Visual + Vocal	Subset of UR Lying Database	107	Eye gaze, Facial expressions, Head pose, MFCC, Prosody, Statistical measures, Statistical measures, Voice quality, eGeMAPd
**72**	624	2021	Neural Network	0.700000	0.800000	0.000000	0.880000	0.000000	0.000000	Bimodal	Visual + Vocal	Subset of Real-life Trial Deception Detection Dataset	108	Eye gaze, Facial arousal, Facial expressions, Facial valence, Head pose, MFCC, Prosody,

														Spectral parameters, Voice energy, Voice quality
**68**	619	2021	Neural Network	0.680000	0.000000	0.000000	0.660000	0.720000	0.000000	Bimodal	Demographical + Visual	Real-life Trial Deception Detection Dataset	121	Age, Face image, Gender
**74**	626	2021	SVM	0.626000	0.000000	0.000000	0.000000	0.000000	0.000000	Bimodal	Physiological + Visual	Deception Detection and Physiological Monitoring Dataset	1680	Eye saccades, Facial micro-expressions, Head pose, Heart rate, Thermal video
**76**	638	2021	SVM	0.770000	0.000000	0.780000	0.840000	0.690000	0.000000	Bimodal	Demographical + Visual	Video recordings of true and false declarations on an interview taken from volunteers.	255026	Ethnicity, Eye saccades, Facial micro-expressions, Gender
**77**	639	2021	Random Forest	0.690000	0.750000	0.000000	0.600000	0.760000	0.000000	Monomodal	Textual	True and deceptive statements collected by a web application from volunteers.	1640	LIWC categories, POS tags
**78**	667	2021	Combined methods	0.000000	0.000000	0.856000	0.000000	0.000000	0.855000	Monomodal	Vocal	Merging of Real-life Trial Deception Detection Dataset and Deceptive Speech Database	1680	INTERSPEECH 2009, INTERSPEECH 2013, INTERSPEECH 2016
**79**	672	2021	Random Forest	0.000000	0.733000	0.711000	0.000000	0.000000	0.000000	Monomodal	Visual	Pupil size measures taken during a card game with a iCub robot.	37	Pupil size
**75**	629	2021	Neural Network	0.967375	0.000000	0.000000	0.000000	0.000000	0.000000	Multimodal	Physiological + Visual + Vocal	Combination of three deception detection datasets	766	EEG channels, Face image, Voice signal
**10**	52	2020	Random Forest	0.998000	0.000000	0.000000	0.000000	0.000000	0.000000	Bimodal	Demographical + Visual	Interview video recordings	86584	Eye gaze, Facial micro-gestures, Gender, Head pose
**11**	60	2020	Random Forest	0.000000	0.897000	0.000000	0.833000	0.833000	0.000000	Monomodal	Visual	Card discovery game played by volunteers	126	Pupil dilation, Pupil dilation latency
**65**	526	2020	Neural Network	0.936000	0.000000	0.000000	0.000000	0.000000	0.000000	Monomodal	Textual	Ott Deceptive Opinion Spam Corpus	1600	BERT embeddings
**42**	394	2020	SVM	0.875900	0.000000	0.000000	0.000000	0.000000	0.000000	Bimodal	Emotional + Visual	Superset of Real-life Trial Deception	190	Emotion Transformation, Eye gaze, Facial

												Detection Dataset		expressions, Hand motion
**20**	177	2020	Neural Network	0.600000	0.000000	0.000000	0.520000	0.420000	0.000000	Bimodal	Demographical + Vocal	Computer-based card game played by volunteers	637	Native language, Voice spectrogram
**26**	193	2020	AdaBoost	0.840000	0.910000	0.840000	0.000000	0.000000	0.000000	Multimodal	Textual + Visual + Vocal	Real-life Trial Deception Detection Dataset	121	Eye gaze, Facial affect, Facial expressions, Head motion, LIWC categories, MFCC, Spectral parameters, Voice pitch, Voice quality
**43**	397	2020	Neural Network	0.728800	0.000000	0.000000	0.000000	0.000000	0.000000	Multimodal	Demographical + Textual + Visual + Vocal	Subset of Real-life Trial Deception Detection Dataset	59	Eye gaze, Facial expressions, Gender, Hand motion, Head motion, LIWC categories, Silence gaps, Unigrams, Voice histogram, Voice pitch
**45**	401	2019	BSSD	0.000000	0.671000	0.000000	0.000000	0.000000	0.000000	Multimodal	Textual + Visual + Vocal	Real-life Trial Deception Detection Dataset	121	Eye gaze, Facial expressions, Head pose, LIWC categories, MFCC, N-grams, POS tags, Syntax complexity, Voice pitch
**1**	19	2019	Neural Network	0.000000	0.000000	0.656150	0.000000	0.000000	0.000000	Multimodal	Demographical + Textual + Vocal	Blind wine-tasting game	12392	Gender, GloVe embeddings, INTERSPEECH 2009, LIWC categories, N-grams, POS tags, Syntax complexity, Voice energy, Voice pitch, Voice quality
**57**	450	2019	Neural Network	0.970000	0.997800	0.000000	0.000000	0.000000	0.000000	Multimodal	Textual + Visual + Vocal	Real-life Trial Deception Detection Dataset	121	Facial expressions, Head motion, Spectral parameters, Unigrams
**47**	409	2019	Neural Network	0.000000	0.000000	0.000000	0.000000	0.000000	0.747100	Bimodal	Dynamical + Vocal	Daily Deceptive Dialogues Corpus of Mandarin	7504	Hesitation duration, MFCC, Silence count, Spectral parameters, Turn duration, Utterance duration, Voice

														energy, Voice pitch, Zero-crossing
**49**	421	2019	Combined methods	0.000000	0.705000	0.466000	0.666000	0.379000	0.000000	Bimodal	Visual + Vocal	Online Resistance game	285	Eye gaze, Facial expressions, Head motion, MFCC
**55**	438	2019	Combined methods	0.661700	0.000000	0.000000	0.000000	0.000000	0.000000	Multimodal	Physiological + Visual + Vocal	Bag-of-lies	325	EEG channels, Eye blinks, Eye gaze, MFCC, Pupil size, Spectral parameters, Zero-crossing
**58**	457	2019	Neural Network	0.510400	0.000000	0.450700	0.000000	0.000000	0.000000	Multimodal	Demographical + Textual + Vocal	Political claims existing in the CT-FCC-18 corpus	286	BERT embeddings, Claim author, INTERSPEECH 2013, LIWC categories, N-grams, TF-IDF, i-vector features
**37**	383	2019	Combined methods	0.970000	0.000000	0.000000	0.000000	0.000000	0.000000	Multimodal	Textual + Visual + Vocal	Real-life Trial Deception Detection Dataset	121	Body motion, Facial micro-expressions, MFCC, N-grams
**38**	384	2019	SVM	0.824700	0.000000	0.000000	0.000000	0.000000	0.000000	Monomodal	Vocal	KWOLF	388	MFCC, Voice energy, Voice pitch, Zero-crossing
**0**	13	2019	Random Forest	0.630000	0.740000	0.000000	0.000000	0.000000	0.000000	Bimodal	Psychological + Visual	Questionnaire answered by volunteers	1054	Eloquence, Eye blinks, Eye gaze, Histronic, NARS, NEO-FFI scores, Narcisistic Machiavellianism, Pupil dilation, Response time
**12**	75	2019	KNN	0.810000	0.000000	0.830000	0.950000	0.760000	0.000000	Monomodal	Emotional	Questionnaire answered by recruts	121	Hand shaking
**13**	80	2019	Neural Network	0.627800	0.000000	0.000000	0.000000	0.000000	0.000000	Monomodal	Vocal	Video recordings of Werewolf online game	987	INTERSPEECH 2009
**27**	198	2019	SVM	0.768400	0.000000	0.000000	0.000000	0.000000	0.000000	Monomodal	Visual	Real-life Trial Deception Detection Dataset	121	Eye gaze, Facial expressions, Head motion
**44**	400	2018	Neural Network	0.746050	0.000000	0.000000	0.000000	0.000000	0.000000	Bimodal	Demographical + Visual	Questionnaire answered by volunteers	400	Ethnicity, Eye gaze, Facial expressions, Gender, Head pose
**59**	466	2018	Neural Network	0.840000	0.000000	0.000000	0.000000	0.000000	0.000000	Bimodal	Demographical + Visual	Questionnaire answered by volunteers	448	Facial expressions, Gender

**15**	118	2018	Random Forest	0.716350	0.000000	0.716000	0.716850	0.716050	0.000000	Multimodal	Demographical + Psychological + Textual	Columbia X-Cultural Dataset	4056	Gender, LIWC categories, Lexical measures, NEO-FFI scores, Native language, Pauses, Syntax complexity
**53**	433	2018	Neural Network	0.640000	0.000000	0.609000	0.667000	0.560000	0.000000	Bimodal	Textual + Vocal	CSC Deceptive Speech	4100	FastText embedding, INTERSPEECH 2009
**14**	115	2018	Logistic Regression	0.000000	0.922100	0.000000	0.000000	0.000000	0.000000	Multimodal	Textual + Visual + Vocal	Subset of Real-life Trial Deception Detection Dataset	104	Facial micro-expressions, GloVe embeddings, MFCC
**22**	179	2018	Neural Network	0.841600	0.000000	0.000000	0.000000	0.000000	0.000000	Bimodal	Visual + Vocal	Real-life Trial Deception Detection Dataset	121	Facial expressions, INTERSPEECH 2009, INTERSPEECH 2013
**48**	414	2018	Neural Network	0.748700	0.000000	0.000000	0.000000	0.000000	0.000000	Monomodal	Vocal	Question-answer experiment answered by selected participants	7867	Spectral parameters, Voice pitch
**63**	500	2018	Decision Tree	0.950000	0.000000	0.000000	0.970000	0.940000	0.000000	Monomodal	Visual	Questionnaire answered by volunteers	40	Eye gaze, Pupil dilation
**46**	402	2018	KNN	0.841600	0.000000	0.000000	0.000000	0.000000	0.000000	Bimodal	Visual + Vocal	Real-life Trial Deception Detection Dataset	121	Facial expressions, INTERSPEECH 2009, INTERSPEECH 2013
**40**	388	2018	SVM	1.000000	0.000000	0.000000	0.000000	0.000000	0.000000	Bimodal	Physiological + Vocal	15-question questionnaire answered by 50 people	750	Blood pressure, Heart rate, MFCC, Respiration rate, Voice energy, Voice pitch, Zero-crossing
**4**	28	2018	Random Forest	0.745300	0.000000	0.716850	0.783700	0.660450	0.000000	Bimodal	Demographical + Vocal	Columbia X-Cultural Dataset	49106	Gender, Native language, Voice energy, Voice pitch, Voice quality
**5**	31	2018	Multi-view Learning	0.980000	0.000000	0.000000	0.000000	0.000000	0.000000	Bimodal	Textual + Visual	Superset of Real-life Trial Deception Detection Dataset	121	Bigrams, Eye gaze, Facial expressions, Hand motion, Head motion, Unigrams
**2**	24	2018	SVM	0.774200	0.000000	0.000000	0.000000	0.000000	0.000000	Monomodal	Textual	Interviews on weekand plans	147	LIWC categories, Named entities,

												collected from volunteers		Psychological processes
**3**	26	2018	Decision Tree	0.696000	0.000000	0.000000	0.000000	0.000000	0.795000	Monomodal	Vocal	Superset of Real-life Trial Deception Detection Dataset	195	INTERSPEECH 2013
**36**	249	2018	Neural Network	0.633333	0.800000	0.733333	0.733333	0.733333	0.000000	Monomodal	Textual	Combination of four publicly available dataset	300	Syntax complexity
**62**	492	2018	SVM	0.551600	0.000000	0.000000	0.551550	0.551450	0.000000	Monomodal	Visual	Computer-based question-answer interview	540	Facial micro-expressions
**6**	33	2017	Neural Network	0.964000	0.000000	0.950000	0.960000	0.950000	0.000000	Multimodal	Textual + Visual + Vocal	Superset of Real-life Trial Deception Detection Dataset	121	Facial expressions, GloVe embeddings, Hand motion, INTERSPEECH 2013
**7**	37	2017	Neural Network	0.000000	0.000000	0.639000	0.000000	0.000000	0.000000	Bimodal	Textual + Vocal	Columbia X-Cultural Dataset	49106	GloVe embeddings, INTERSPEECH 2009, INTERSPEECH 2013, MFCC, N-grams
**54**	435	2017	Decision Tree	0.892600	0.000000	0.000000	0.000000	0.892300	0.000000	Multimodal	Physiological + Textual + Thermal	Mock crime game and a questionnaire about two sensitive themes answered by 30 volunteers	149	Blood volume, Cheeks, Forehead, Heart rate, LIWC categories, Nose, POS tags, Periorbital region, Respiration volume, Skin conductance, Syntax complexity, TF-IDF
**21**	178	2017	Decision Tree	0.664000	0.000000	0.000000	0.000000	0.000000	0.000000	Multimodal	Demographical + Physiological + Textual + Thermal	Mock crime game and a questionnaire about two sensitive themes answered by 104 volunteers	520	Face region, Gender, Heart rate, LIWC categories, POS tags, Respiration rate, Skin conductance, Syntax complexity, Unigrams
**16**	129	2017	Clustering	0.683000	0.000000	0.000000	0.000000	0.000000	0.000000	Bimodal	Demographical + Textual	Russian Deception Bank	226	Age, Education, Gender, LIWC categories, POS tags
**8**	38	2017	Random Forest	0.612600	0.000000	0.613000	0.000000	0.000000	0.000000	Bimodal	Textual + Vocal	Indonesian Deception Corpus	5542	INTERSPEECH 2010, LIWC categories,

														Silence gaps, Voice energy, Voice pitch
**52**	431	2016	SVM	0.789500	0.000000	0.000000	0.000000	0.000000	0.000000	Multimodal	Textual + Visual + Vocal	Subset of Real-life Trial Deception Detection Dataset	100	Facial expressions, MFCC, POS tags, Prosody, Sentiment, Unigrams, Voice energy
**28**	203	2016	Decision Tree	0.617400	0.000000	0.000000	0.000000	0.000000	0.000000	Bimodal	Thermal + Visual	A trivia game answered by volunteers	149	Entire face, Eye blinks, Facial expressions, Head motion
**66**	545	2016	SVM	0.620000	0.000000	0.000000	0.640000	0.630000	0.000000	Monomodal	Visual	Gaze and pupil data collected during questionnaire answering by volunteers	50	Eye saccades, Pupil dilation, Response time
**41**	393	2016	SVM	0.863750	0.000000	0.000000	0.000000	0.000000	0.000000	Monomodal	Vocal	ReliDDB	137640	MFCC, Voice pitch
**60**	478	2016	SVM	0.729500	0.000000	0.000000	0.000000	0.000000	0.000000	Bimodal	Demographical + Vocal	Soochow Deceptive Speech Detection Corpus	4143	Gender, MFCC, Spectral parameters, Zero-crossing
**9**	40	2015	Decision Tree	0.000000	0.000000	0.809500	0.793400	0.835550	0.000000	Bimodal	Demographical + Vocal	Soochow Deceptive Speech Detection Corpus	3787	Duration, Formant, Gender, Voice energy, Voice pitch
**17**	148	2015	SVM	0.695000	0.000000	0.000000	0.000000	0.000000	0.000000	Bimodal	Demographical + Textual	Open domain sentences collected from volunteers	7168	Age, Education, Gender, LIWC categories, Nationality, POS tags, Syntax complexity, Unigrams
**56**	447	2015	SVM	0.000000	0.000000	0.000000	0.668000	0.659000	0.000000	Monomodal	Visual	From a trivia game with volunteers	100	Facial expressions, Head motion, Interactional synchrony
**24**	183	2015	Random Forest	0.735500	0.000000	0.000000	0.000000	0.000000	0.000000	Bimodal	Textual + Visual	Real-life Trial Deception Detection Dataset	121	Bigrams, Body motion, Facial expressions, Head motion, Unigrams
**35**	244	2015	Logistic Regression	0.702600	0.639100	0.000000	0.000000	0.000000	0.000000	Monomodal	Visual	Mafia DB	6733	Facial expressions
**29**	207	2015	Random Forest	0.658900	0.000000	0.000000	0.000000	0.000000	0.000000	Multimodal	Demographical + Psychological + Vocal	Statements provided by volunteers in a mock fake resume game	154	Ethnicity, Gender, NEO-FFI scores, Native language, Speaking rate, Voice energy,

														Voice pitch, Voice quality
**39**	387	2015	Decision Tree	0.980000	0.000000	0.000000	0.000000	0.000000	0.000000	Monomodal	Textual	Communications during sessions of the online mafia game	142	LIWC categories, Syntax complexity, Unigrams
**61**	482	2015	Neural Network	0.800000	0.000000	0.000000	0.000000	0.000000	0.000000	Monomodal	Thermal	From a mock crime game answered by volunteers	40	Perinasal region
**23**	180	2014	Decision Tree	0.701000	0.000000	0.000000	0.000000	0.000000	0.000000	Multimodal	Physiological + Textual + Thermal	Statements from volunteers in an opinion game	120	Blood volume, Entire face, LIWC categories, Respiration volume, Skin conductance, Unigrams
**64**	503	2014	Gradient Boosting	0.910000	0.000000	0.000000	0.000000	0.000000	0.000000	Monomodal	Textual	Statements provided by volunteers in a mock chat room	254	Emoticons, Informality, Sentiment, Syntax complexity
**50**	422	2014	KNN	0.868800	0.000000	0.000000	0.000000	0.000000	0.000000	Monomodal	Thermal	Statements from volunteers in an interview game	492	Periorbital region
**51**	425	2013	Neural Network	0.833300	0.000000	0.000000	0.000000	0.000000	0.000000	Monomodal	Vocal	Utterances from recordings of police interrogations	6	Bark, Significant energy
**32**	220	2013	SVM	0.737000	0.000000	0.000000	0.000000	0.000000	0.000000	Monomodal	Textual	Video recordings from volunteers	140	Unigrams
**25**	188	2012	SVM	0.912000	0.000000	0.000000	0.000000	0.000000	0.000000	Monomodal	Textual	Reviews of 35 Italian restaurants	2692	Bigrams, POS tags, Syntax complexity, Unigrams
**30**	213	2012	Decision Tree	0.944700	0.000000	0.000000	0.000000	0.000000	0.000000	Bimodal	Thermal + Vocal	Participants of an experiment in UE border control	259	Eye gaze, Pupil dilation, Voice energy, Voice pitch, Voice quality
**18**	171	2012	Decision Tree	0.650000	0.000000	0.000000	0.000000	0.000000	0.000000	Monomodal	Textual	Stories written by volunteers	90	LIWC categories, Lexical measures
**33**	223	2012	SVM	0.659600	0.000000	0.601200	0.718500	0.625600	0.000000	Monomodal	Textual	DeCour corpus	3015	LIWC categories, Lexical measures, N-grams, POS tags
**31**	217	2012	SVM	0.000000	0.000000	0.702000	0.000000	0.000000	0.000000	Monomodal	Textual	Opinions from volunteers on three themes	600	LIWC categories
**19**	173	2011	SMO	0.650000	0.000000	0.000000	0.000000	0.000000	0.000000	Monomodal	Textual	Stories written by volunteers	90	LIWC categories, Lexical measures
**34**	233	2011	SVM	0.000000	0.000000	0.760000	0.000000	0.000000	0.000000	Bimodal	Visual + Vocal	Wolf-database	72	Body motion, Non-silent

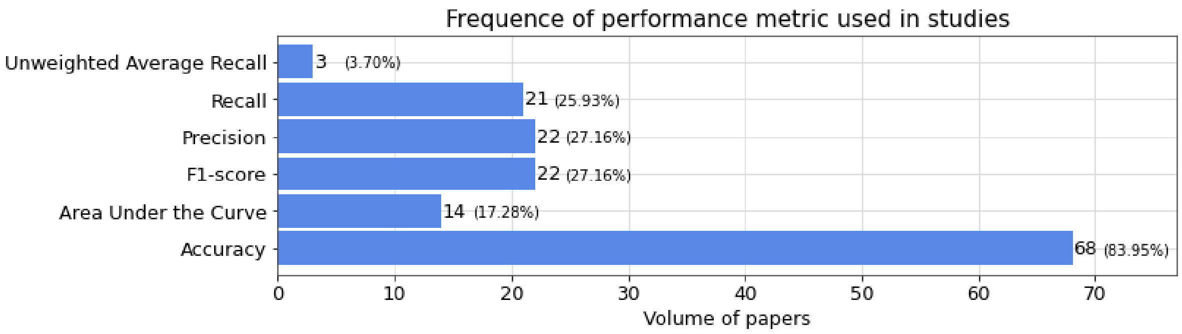

8.2. Performance and modality

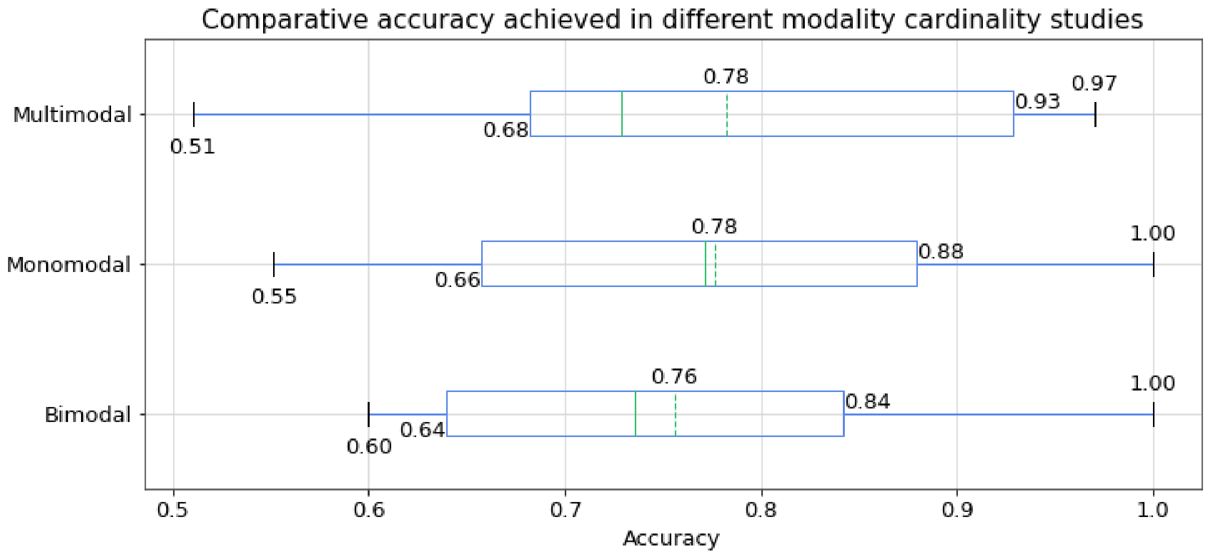

8.3. Performance in Monomodal approaches

Accuracies achieved by Monomodal Texual studies

	Year	Authors	Title	Accuracy	Technique	Features	Dataset	Dataset cardinality
**39**	2015	Pak, J.; Zhou, L.	A comparison of features for automatic deception detection in synchronous computer-mediated communication	0.980000	Decision Tree	LIWC categories, Syntax complexity, Unigrams	Communications during sessions of the online mafia game	142
**65**	2020	Barsever, D.; Singh, S.; Neftci, E.	Building a Better Lie Detector with BERT: The Difference Between Truth and Lies	0.936000	Neural Network	BERT embeddings	Ott Deceptive Opinion Spam Corpus	1600

**25**	2012	Feng, Song; Banerjee, Ritwik; Choi, Yejin	Syntactic Stylometry for Deception Detection	0.912000	SVM	Bigrams, POS tags, Syntax complexity, Unigrams	Reviews of 35 Italian restaurants	2692
**64**	2014	Briscoe, E. J.; Appling, D. S.; Hayes, H.	Cues to Deception in Social Media Communications	0.910000	Gradient Boosting	Emoticons, Informality, Sentiment, Syntax complexity	Statements provided by volunteers in a mock chat room	254
**2**	2018	Kleinberg, Bennett; van der Toolen, Yaloe; Vrij, Aldert; Arntz, Arnoud; Verschuere, Bruno	Automated verbal credibility assessment of intentions: The model statement technique and predictive modeling	0.774200	SVM	LIWC categories, Named entities, Psychological processes	Interviews on weekand plans collected from volunteers	147
**32**	2013	Perez-Rosas, Veronica; Mihalcea, Rada; Burzo, Mihai	Automatic Detection of Deceit in Verbal Communication	0.737000	SVM	Unigrams	Video recordings from volunteers	140
**77**	2021	Kleinberg, Bennett; Verschuere, Bruno	How humans impair automated deception detection performance	0.690000	Random Forest	LIWC categories, POS tags	True and deceptive statements collected by a web application from volunteers.	1640
**33**	2012	Fornaciari, Tommaso; Poesio, Massimo	On the Use of Homogenous Sets of Subjects in Deceptive Language Analysis	0.659600	SVM	LIWC categories, Lexical measures, N-grams, POS tags	DeCour corpus	3015
**18**	2012	Rubin, V.L.; Conroy, N.	Discerning truth from deception: Human judgments and automation efforts	0.650000	Decision Tree	LIWC categories, Lexical measures	Stories written by volunteers	90
**19**	2011	Conroy, N.J.; Rubin, V.L.	Challenges in automated deception detection in computer-mediated communication	0.650000	SMO	LIWC categories, Lexical measures	Stories written by volunteers	90
**36**	2018	Mbaziira, Alex V.; Murphy, Diane R.	An Empirical Study on Detecting Deception and Cybercrime Using Artificial Neural Networks	0.633333	Neural Network	Syntax complexity	Combination of four publicly available dataset	300

Accuracies achieved by Monomodal Visual studies

	Year	Authors	Title	Accuracy	Technique	Features	Dataset	Dataset cardinality
**63**	2018	Labibah, Z.; Nasrun, M.; Setianingsih, C.	Lie Detector With The Analysis Of The Change Of Diameter Pupil and The Eye Movement Use Method Gabor Wavelet Transform and Decision Tree	0.950000	Decision Tree	Eye gaze, Pupil dilation	Questionnaire answered by volunteers	40
**27**	2019	Avola, Danilo; Cinque, Luigi; Foresti, Gian Luca; Pannone, Daniele	Automatic Deception Detection in RGB Videos Using Facial Action Units	0.768400	SVM	Eye gaze, Facial expressions, Head motion	Real-life Trial Deception Detection Dataset	121
**35**	2015	Demyanov, Sergey; Bailey, James; Ramamohanarao, Kotagiri; Leckie, Christopher	Detection of Deception in the Mafia Party Game	0.702600	Logistic Regression	Facial expressions	Mafia DB	6733
**66**	2016	Rybar, M.; Bielikova, M.	Automated detection of user deception in on-line questionnaires with focus on eye tracking use	0.620000	SVM	Eye saccades, Pupil dilation, Response time	Gaze and pupil data collected during questionnaire answering by volunteers	50
**67**	2021	Islam, Siam; Saha, Popin; Chowdhury, Touhidul; Sorowar, Asif; Rab, Raqeebir	Non-invasive Deception Detection in Videos Using Machine Learning Techniques	0.615400	SVM	Facial expressions	Subset of features from Bag-of-lies	325
**62**	2018	Takabatake, S.; Shimada, K.; Saitoh, T.	Construction of a Liar Corpus and Detection of Lying Situations	0.551600	SVM	Facial micro-expressions	Computer-based question-answer interview	540

Accuracies achieved by Monomodal Vocal studies

	Year	Authors	Title	Accuracy	Technique	Features	Dataset	Dataset cardinality

**70**	2021	Ullah, Muhammad S.; Fernandes, Sinead V.	Use of Machine Learning for Deception Detection From Spectral and Cepstral Features of Speech Signals	1.000000	Neural Network	Delta cepstrum, Delta energy, Time difference cepstrum, Time difference energy	Three sessions of a police interrogation on a suspect.	12
**69**	2021	Ullah, Muhammad S.; Fernandes, Sinead V.	Development of Spectral Speech Features for Deception Detection Using Neural Networks	0.916700	Neural Network	Bark	Three sessions of a police interrogation on a suspect.	12
**41**	2016	Nasri, H.; Ouarda, W.; Alimi, A. M.	ReLiDSS: Novel lie detection system from speech signal	0.863750	SVM	MFCC, Voice pitch	ReliDDB	137640
**51**	2013	Sanaullah, M.; Gopalan, K.	Deception detection in speech using bark band and perceptually significant energy features	0.833300	Neural Network	Bark, Significant energy	Utterances from recordings of police interrogations	6
**38**	2019	Fu, H.; Lei, P.; Tao, H.; Wang, M.; Wang, J.	Speech Deception Detection Algorithm Based on SVM and Acoustic Features	0.824700	SVM	MFCC, Voice energy, Voice pitch, Zero-crossing	KWOLF	388
**48**	2018	Tao, H.; Zhao, L.; Xie, Y.; Liang, R.; Zhu, Y.	Convolutional Bidirectional Long Short-Term Memory for Deception Detection With Acoustic Features	0.748700	Neural Network	Spectral parameters, Voice pitch	Question-answer experiment answered by selected participants	7867
**3**	2018	Velichko, Alena; Budkov, Viktor; Kagirov, Ildar; Karpov, Alexey	Comparative Analysis of Classification Methods for Automatic Deception Detection in Speech	0.696000	Decision Tree	INTERSPEECH 2013	Superset of Real-life Trial Deception Detection Dataset	195
**13**	2019	Fu, H.; Lei, P.; Tao, H.; Zhao, L.; Yang, J.	Improved semi-supervised autoencoder for deception detection	0.627800	Neural Network	INTERSPEECH 2009	Video recordings of Werewolf online game	987

Accuracies achieved by Monomodal Thermal studies

	Year	Authors	Title	Accuracy	Technique	Features	Dataset	Dataset cardinality
**50**	2014	Rajoub, B. A.; Zwiggelaar, R.	Thermal Facial Analysis for Deception Detection	0.868800	KNN	Periorbital region	Statements from volunteers in an interview game	492
**61**	2015	Burgoon, J. K.; Dcosta, M.; Shastri, D.; Vilalta, R.; Pavlidis, I.	Perinasal indicators of deceptive behavior	0.800000	Neural Network	Perinasal region	From a mock crime game answered by volunteers	40

Accuracies achieved by Monomodal Emotional studies

	Year	Authors	Title	Accuracy	Technique	Features	Dataset	Dataset cardinality
**12**	2019	Mizanur Rahman, Md.; Shome, A.; Chellappan, S.; Alim Al Islam, A.B.M.	How smart your smartphone is in lie detection?	0.810000	KNN	Hand shaking	Questionnaire answered by recruts	121

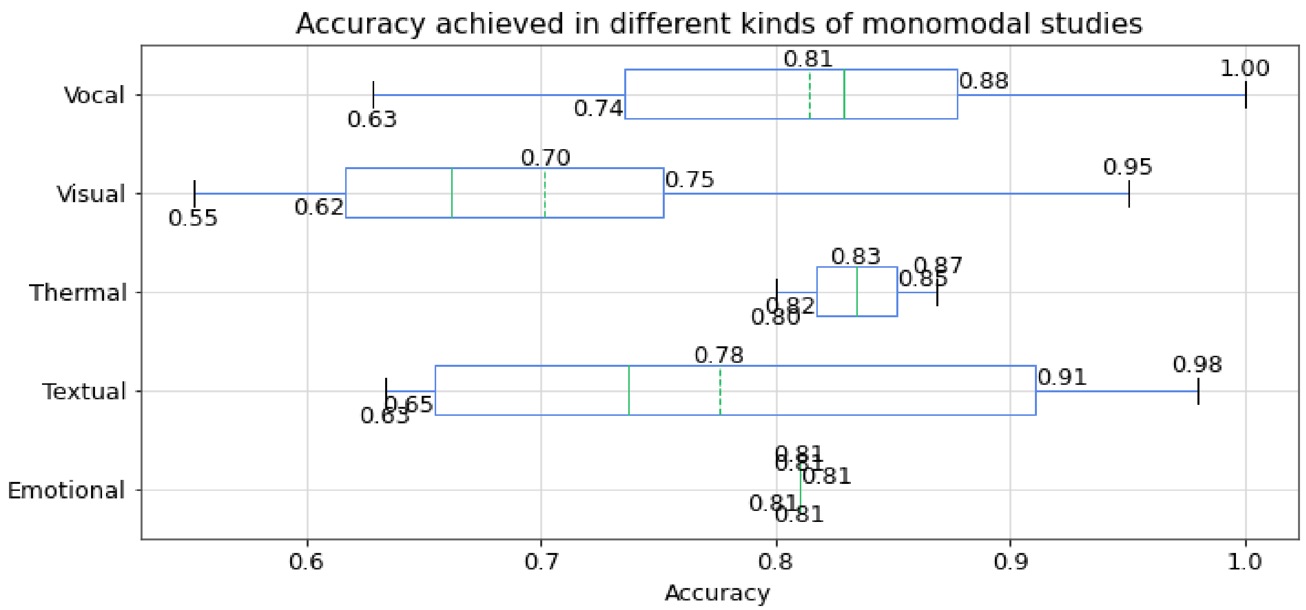

8.4. Performance in Bimodal approaches

Accuracies achieved by Bimodal Demographical + Visual studies

	Year	Authors	Title	Accuracy	Technique	Features	Dataset	Dataset cardinality
**10**	2020	Crockett, K.; OShea, J.; Khan, W.	Automated Deception Detection of Males and Females from Non-Verbal Facial Micro-Gestures	0.998000	Random Forest	Eye gaze, Facial micro-gestures, Gender, Head pose	Interview video recordings	86584
**59**	2018	Thannoon, H. H.; Ali, W. H.; Hashim, I. A.	Detection of Deception Using Facial Expressions Based on Different Classification Algorithms	0.840000	Neural Network	Facial expressions, Gender	Questionnaire answered by volunteers	448
**76**	2021	Khan, Wasiq; Hussain, Abir; Crockett, Keeley; OShea, James; Khan, Bilal M.	Deception in the eyes of deceiver: A computer vision and machine learning based automated deception detection	0.770000	SVM	Ethnicity, Eye saccades, Facial micro-expressions, Gender	Video recordings of true and false declarations on an interview taken from volunteers.	255026
**44**	2018	Crockett, K.; Khan, W.; O'Shea, J.; Kindynis, P.; Antoniades, A.; Boultadakis, G.	Intelligent Deception Detection through Machine Based Interviewing	0.746050	Neural Network	Ethnicity, Eye gaze, Facial expressions, Gender, Head pose	Questionnaire answered by volunteers	400
**68**	2021	Ngô, Lê Minh; Wang, Wei; Mandira, Burak; Karaoğlu, Sezer; Bouma, Henri; Dibeklioğlu, Hamdi; Gevers, Theo	Identity Unbiased Deception Detection by 2D-to-3D Face Reconstruction	0.680000	Neural Network	Age, Face image, Gender	Real-life Trial Deception Detection Dataset	121

Accuracies achieved by Bimodal Textual + Visual studies

	Year	Authors	Title	Accuracy	Technique	Features	Dataset	Dataset cardinality

**5**	2018	Carissimi, Nicolo; Beyan, Cigdem; Murino, Vittorio	A Multi-View Learning Approach To Deception Detection	0.980000	Multi-view Learning	Bigrams, Eye gaze, Facial expressions, Hand motion, Head motion, Unigrams	Superset of Real-life Trial Deception Detection Dataset	121
**24**	2015	Perez-Rosas, Veronica; Mihalcea, Rada; Abouelenien, Mohamed; Burzo, Mihai	Deception Detection Using Real-Life Trial Data	0.735500	Random Forest	Bigrams, Body motion, Facial expressions, Head motion, Unigrams	Real-life Trial Deception Detection Dataset	121

Accuracies achieved by Bimodal Visual + Vocal studies

	Year	Authors	Title	Accuracy	Technique	Features	Dataset	Dataset cardinality
**46**	2018	Karimi, H.; Tang, J.; Li, Y.	Toward End-to-End Deception Detection in Videos	0.841600	KNN	Facial expressions, INTERSPEECH 2009, INTERSPEECH 2013	Real-life Trial Deception Detection Dataset	121
**22**	2018	Karimi, Hamid	Interpretable Multimodal Deception Detection in Videos	0.841600	Neural Network	Facial expressions, INTERSPEECH 2009, INTERSPEECH 2013	Real-life Trial Deception Detection Dataset	121
**71**	2021	Mathur, Leena; Matarić, Maja J.	Unsupervised Audio-Visual Subspace Alignment for High-Stakes Deception Detection	0.740000	KNN	Eye gaze, Facial expressions, Head pose, MFCC, Prosody, Statistical measures, Statistical measures, Voice quality, eGeMAPd	Subset of UR Lying Database	107
**72**	2021	Mathur, Leena; Matarić, Maja J	Affect-Aware Deep Belief Network Representations for Multimodal Unsupervised Deception Detection	0.700000	Neural Network	Eye gaze, Facial arousal, Facial expressions, Facial valence, Head pose, MFCC, Prosody, Spectral parameters, Voice energy, Voice quality	Subset of Real-life Trial Deception Detection Dataset	108

Accuracies achieved by Bimodal Demographical + Vocal studies

	Year	Authors	Title	Accuracy	Technique	Features	Dataset	Dataset cardinality
**4**	2018	Levitan, Sarah Ita; Maredia, Angel; Hirschberg, Julia	Acoustic-Prosodic Indicators of Deception and Trust in Interview Dialogues	0.745300	Random Forest	Gender, Native language, Voice energy, Voice pitch, Voice quality	Columbia X-Cultural Dataset	49106
**60**	2016	Fan, Cheng; Zhao, Heming; Chen, Xueqin; Fan, Xiaohe; Chen, Shuxi	Deceptive Speech Detection based on sparse representation	0.729500	SVM	Gender, MFCC, Spectral parameters, Zero-crossing	Soochow Deceptive Speech Detection Corpus	4143
**20**	2020	Azaria, Amos; Hershkovitch Neiterman, Evgeny; Bitan, Moshe	Multilingual Deception Detection by Autonomous Agents	0.600000	Neural Network	Native language, Voice spectrogram	Computer-based card game played by volunteers	637

Accuracies achieved by Bimodal Demographical + Textual studies

	Year	Authors	Title	Accuracy	Technique	Features	Dataset	Dataset cardinality
**17**	2015	Pérez-Rosas, V.; Mihalcea, R.	Experiments in open domain deception detection	0.695000	SVM	Age, Education, Gender, LIWC categories, Nationality, POS tags, Syntax complexity, Unigrams	Open domain sentences collected from volunteers	7168
**16**	2017	Litvinova, O.; Litvinova, T.; Seredin, P.; Lyell, J.	Deception detection in Russian texts	0.683000	Clustering	Age, Education, Gender, LIWC categories, POS tags	Russian Deception Bank	226
**80**	2021	Papantoniou, K.; Papadakos, P.; Patkos, T.; Flouris, G.; Androutsopoulos, I.; Plexousakis, D.	Deception detection in text and its relation to the cultural dimension of individualism/collectivism	0.624000	Logistic Regression	BERT embeddings, Culture, Language, Lexical measures, N-grams, Phonemes, Pronouns, Relativity, Sentiment, Syntax complexity	Eleven multidomain and multicultural datasets.	7024

Accuracies achieved by Bimodal Textual + Vocal studies

	Year	Authors	Title	Accuracy	Technique	Features	Dataset	Dataset cardinality
**53**	2018	Hosomi, N.; Sakti, S.; Yoshino, K.; Nakamura, S.	Deception Detection and Analysis in Spoken Dialogues based on FastText	0.640000	Neural Network	FastText embedding, INTERSPEECH 2009	CSC Deceptive Speech	4100
**8**	2017	Warnita, Tifani; Lestari, Dessi Puji	Construction and Analysis of Indonesian-Interviews Deception Corpus	0.612600	Random Forest	INTERSPEECH 2010, LIWC categories, Silence gaps, Voice energy, Voice pitch	Indonesian Deception Corpus	5542

Accuracies achieved by Bimodal Physiological + Vocal studies

	Year	Authors	Title	Accuracy	Technique	Features	Dataset	Dataset cardinality
**40**	2018	Srivastava, N.; Dubey, S.	Deception detection using artificial neural network and support vector machine	1.000000	SVM	Blood pressure, Heart rate, MFCC, Respiration rate, Voice energy, Voice pitch, Zero-crossing	15-question questionnaire answered by 50 people	750

Accuracies achieved by Bimodal Psychological + Visual studies

	Year	Authors	Title	Accuracy	Technique	Features	Dataset	Dataset cardinality
**0**	2019	Gonzalez-Billandon, Jonas; Aroyo, Alexander M.; Tonelli, Alessia; Pasquali, Dario; Sciutti, Alessandra; Gori, Monica; Sandini, Giulio; Rea, Francesco	Can a Robot Catch You Lying? A Machine Learning System to Detect Lies During Interactions	0.630000	Random Forest	Eloquence, Eye blinks, Eye gaze, Histronic, NARS, NEO-FFI scores, Narcisistic Machiavellianism, Pupil dilation, Response time	Questionnaire answered by volunteers	1054

Accuracies achieved by Bimodal Thermal + Visual studies

	Year	Authors	Title	Accuracy	Technique	Features	Dataset	Dataset cardinality
**28**	2016	Mihalcea, Rada; Abouelenien, Mohamed; Burzo, Mihai	Analyzing Thermal and Visual Clues of Deception for a Non-Contact Deception Detection Approach	0.617400	Decision Tree	Entire face, Eye blinks, Facial expressions, Head motion	A trivia game answered by volunteers	149

Accuracies achieved by Bimodal Thermal + Vocal studies

	Year	Authors	Title	Accuracy	Technique	Features	Dataset	Dataset cardinality
**30**	2012	Elkins, Aaron C.; Derrick, Douglas C.; Gariup, Monica	The Voice and Eye Gaze Behavior of an Imposter: Automated Interviewing and Detection for Rapid Screening at the Border	0.944700	Decision Tree	Eye gaze, Pupil dilation, Voice energy, Voice pitch, Voice quality	Participants of an experiment in UE border control	259

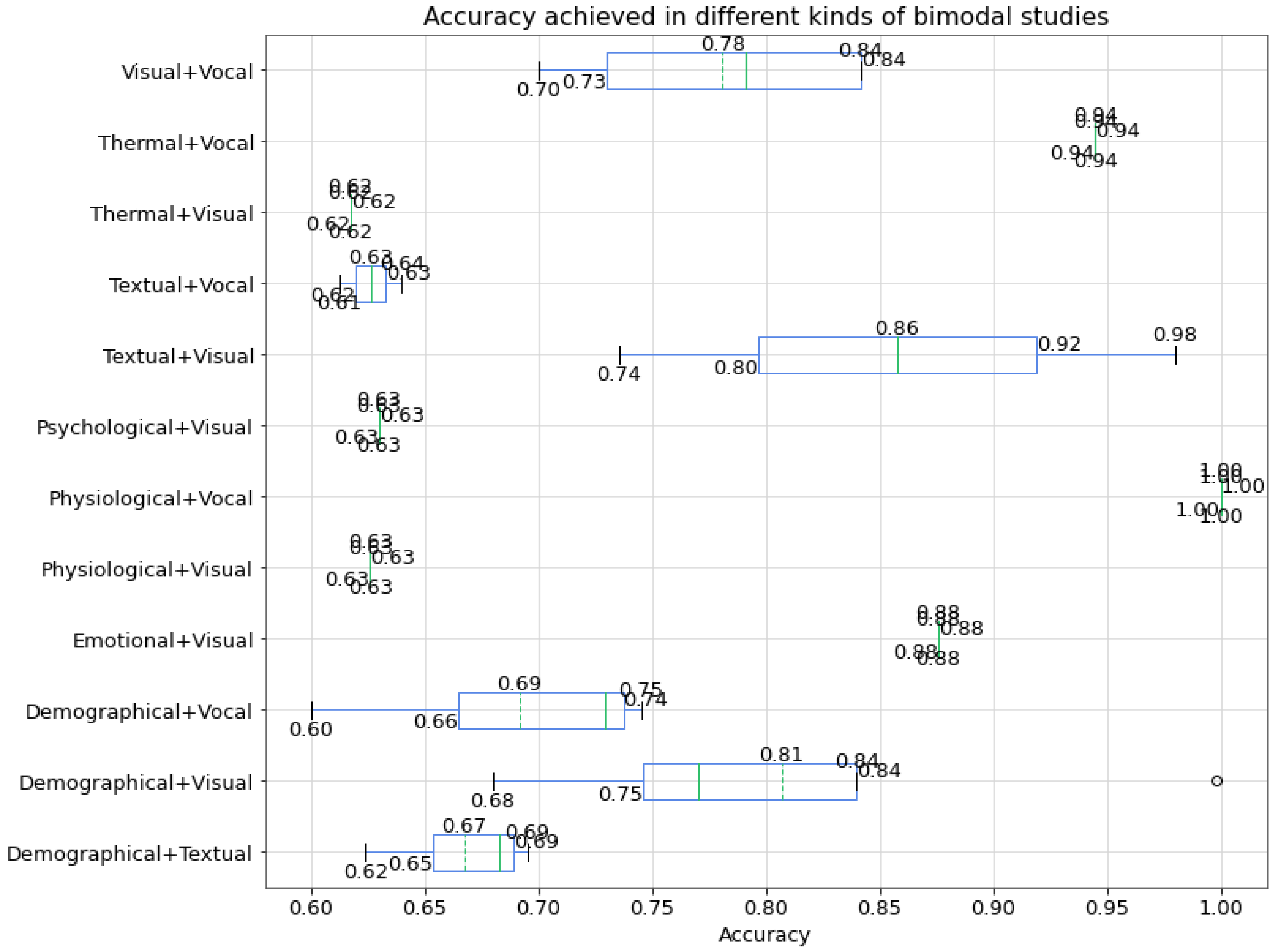

8.5. Performance in Multimodal approaches

Accuracies achieved by Multimodal Textual + Visual + Vocal studies								
	Year	Authors	Title	Accuracy	Technique	Features	Dataset	Dataset cardinality
**37**	2019	Venkatesh, S.; Ramachandra, R.; Bours, P.	Robust Algorithm for Multimodal Deception Detection	0.970000	Combined methods	Body motion, Facial micro-expressions, MFCC, N-grams	Real-life Trial Deception Detection Dataset	121

**57**	2019	Ding, M.; Zhao, A.; Lu, Z.; Xiang, T.; Wen, J.	Face-Focused Cross-Stream Network for Deception Detection in Videos	0.970000	Neural Network	Facial expressions, Head motion, Spectral parameters, Unigrams	Real-life Trial Deception Detection Dataset	121
**6**	2017	Gogate, Mandar; Adeel, Ahsan; Hussain, Amir	Deep Learning Driven Multimodal Fusion For Automated Deception Detection	0.964000	Neural Network	Facial expressions, GloVe embeddings, Hand motion, INTERSPEECH 2013	Superset of Real-life Trial Deception Detection Dataset	121
**26**	2020	Mathur, Leena; Mataric, Maja J.	Introducing Representations of Facial Affect in Automated Multimodal Deception Detection	0.840000	AdaBoost	Eye gaze, Facial affect, Facial expressions, Head motion, LIWC categories, MFCC, Spectral parameters, Voice pitch, Voice quality	Real-life Trial Deception Detection Dataset	121
**52**	2016	Jaiswal, M.; Tabibu, S.; Bajpai, R.	The Truth and Nothing But the Truth: Multimodal Analysis for Deception Detection	0.789500	SVM	Facial expressions, MFCC, POS tags, Prosody, Sentiment, Unigrams, Voice energy	Subset of Real-life Trial Deception Detection Dataset	100
**73**	2021	Abouelenien, Mohamed; Hessler, Christian; Kamboj, Manvi; Asnani, Priyanka; Riani, Kais	Multimodal Political Deception Detection	0.700000	Decision Tree	Eye gaze, Facial emotion, Facial expressions, GloVe embeddings, Head pose, INTERSPEECH 2009, INTERSPEECH 2013, LIWC categories, POS tags, Sentiment, Unigrams	Videos with political debates with deceptions checked by PolitiFact.org	180

Accuracies achieved by Multimodal Physiological + Textual + Thermal studies

	Year	Authors	Title	Accuracy	Technique	Features	Dataset	Dataset cardinality
**54**	2017	Pérez-Rosas, V.; Mihalcea, R.; Abouelenien, M.; Burzo, M.	Detecting Deceptive Behavior via Integration of Discriminative Features From Multiple Modalities	0.892600	Decision Tree	Blood volume, Cheeks, Forehead, Heart rate, LIWC categories, Nose, POS tags, Periorbital region, Respiration volume, Skin conductance, Syntax complexity, TF-IDF	Mock crime game and a questionnaire about two sensitive themes answered by 30 volunteers	149
**23**	2014	Perez-Rosas, Veronica; Mihalcea, Rada; Abouelenien, Mohamed; Burzo, Mihai	Deception Detection Using a Multimodal Approach	0.701000	Decision Tree	Blood volume, Entire face, LIWC categories, Respiration volume, Skin conductance, Unigrams	Statements from volunteers in an opinion game	120

Accuracies achieved by Multimodal Demographical + Textual + Visual + Vocal studies

	Year	Authors	Title	Accuracy	Technique	Features	Dataset	Dataset cardinality
**43**	2020	Mihalcea, R.; Abouelenien, M.; Burzo, M.; Sen, U. M.; Perez-Rosas, V.; Yanikoglu, B.	Multimodal Deception Detection using Real-Life Trial Data	0.728800	Neural Network	Eye gaze, Facial expressions, Gender, Hand motion, Head motion, LIWC categories, Silence gaps, Unigrams, Voice histogram, Voice pitch	Subset of Real-life Trial Deception Detection Dataset	59

Accuracies achieved by Multimodal Demographical + Physiological + Textual + Thermal studies

	Year	Authors	Title	Accuracy	Technique	Features	Dataset	Dataset cardinality
**21**	2017	Perez-Rosas, Veronica; Mihalcea, Rada; Abouelenien, Mohamed; Zhao, Bohan; Burzo, Mihai	Gender-Based Multimodal Deception Detection	0.664000	Decision Tree	Face region, Gender, Heart rate, LIWC categories, POS tags, Respiration rate, Skin conductance, Syntax complexity, Unigrams	Mock crime game and a questionnaire about two sensitive themes answered by 104 volunteers	520

Accuracies achieved by Multimodal Physiological + Visual + Vocal studies

	Year	Authors	Title	Accuracy	Technique	Features	Dataset	Dataset cardinality
**75**	2021	Karnati, Mohan; Seal, Ayan; Yazidi, Anis; Krejcar, Ondrej	LieNet: A Deep Convolution Neural Networks Framework for Detecting Deception	0.967375	Neural Network	EEG channels, Face image, Voice signal	Combination of three deception detection datasets	766

**55**	2019	Gupta, V.; Agarwal, M.; Arora, M.; Chakraborty, T.; Singh, R.; Vatsa, M.	Bag-of-Lies: A Multimodal Dataset for Deception Detection	0.661700	Combined methods	EEG channels, Eye blinks, Eye gaze, MFCC, Pupil size, Spectral parameters, Zero-crossing	Bag-of-lies	325

Accuracies achieved by Multimodal Demographical + Psychological + Vocal studies

	Year	Authors	Title	Accuracy	Technique	Features	Dataset	Dataset cardinality
**29**	2015	Hirschberg, Julia; Mendels, Gideon; Levitan, Sarah I.; An, Guzhen; Wang, Mandi; Levine, Michelle; Rosenberg, Andrew	Cross-Cultural Production and Detection of Deception from Speech	0.658900	Random Forest	Ethnicity, Gender, NEO-FFI scores, Native language, Speaking rate, Voice energy, Voice pitch, Voice quality	Statements provided by volunteers in a mock fake resume game	154

Accuracies achieved by Multimodal Demographical + Textual + Vocal studies

	Year	Authors	Title	Accuracy	Technique	Features	Dataset	Dataset cardinality
**58**	2019	Kopev, D.; Ali, A.; Koychev, I.; Nakov, P.	Detecting Deception in Political Debates Using Acoustic and Textual Features	0.510400	Neural Network	BERT embeddings, Claim author, INTERSPEECH 2013, LIWC categories, N-grams, TF-IDF, i-vector features	Political claims existing in the CT-FCC-18 corpus	286

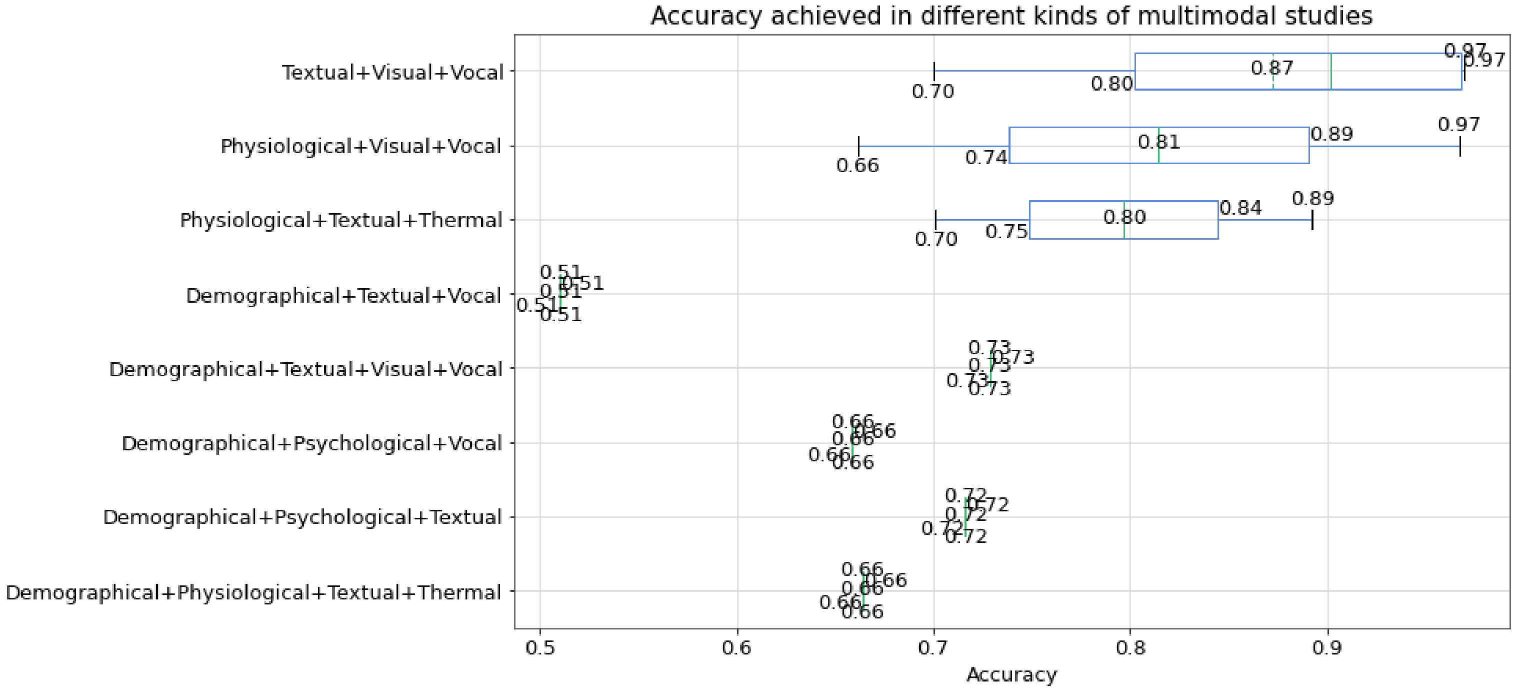

8.6. Accuracy by Technique analysis

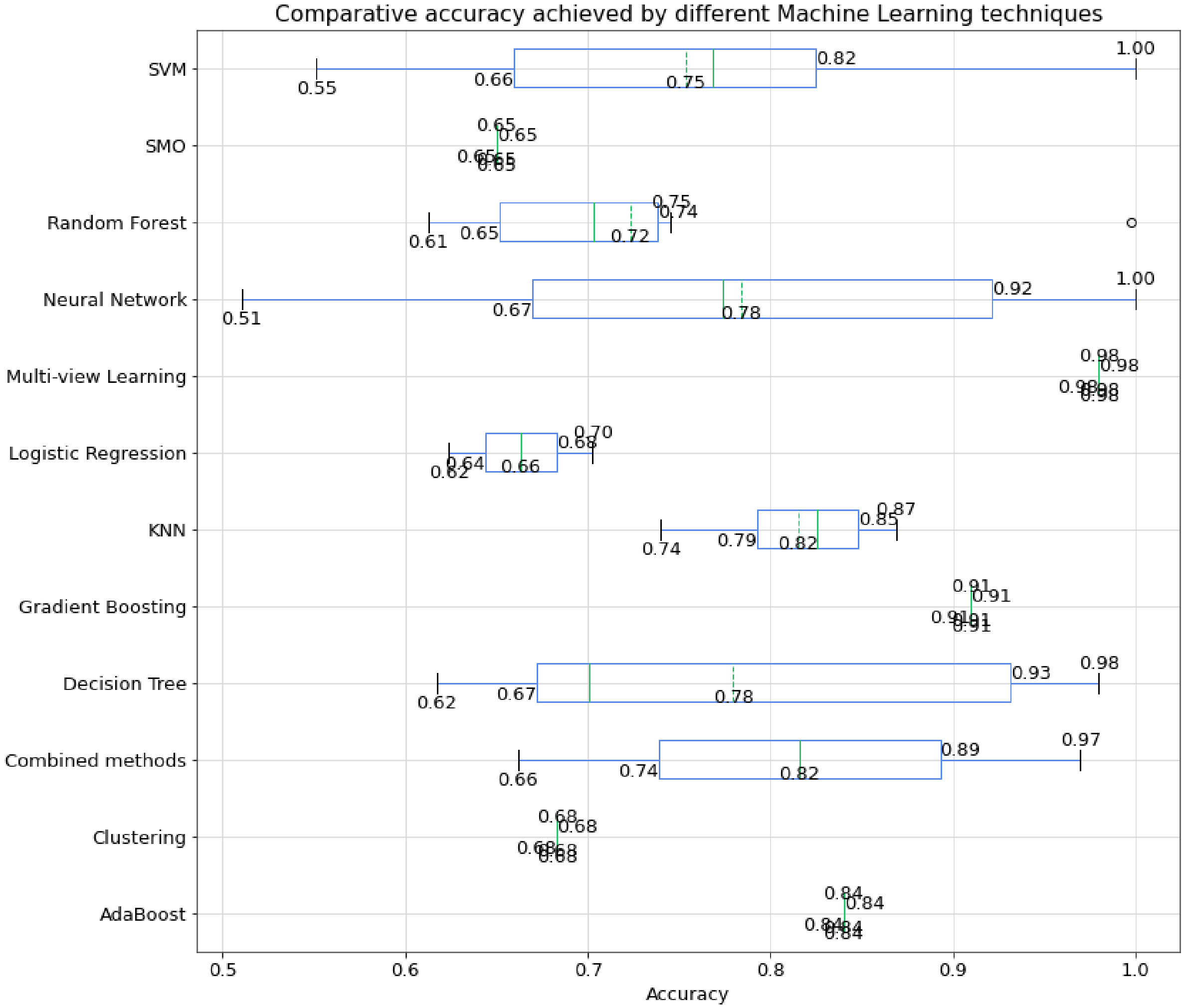

Comparative accuracy achieved by different Machine Learning techniques
SVM
SMO
Random Forest
Neural Network
Multi-view Learning
Logistic Regression
KNN
Gradient Boosting
Decision Tree
Combined methods
Clustering
AdaBoost
Accuracy
0.5
0.6
0.7
0.8
0.9
1.0

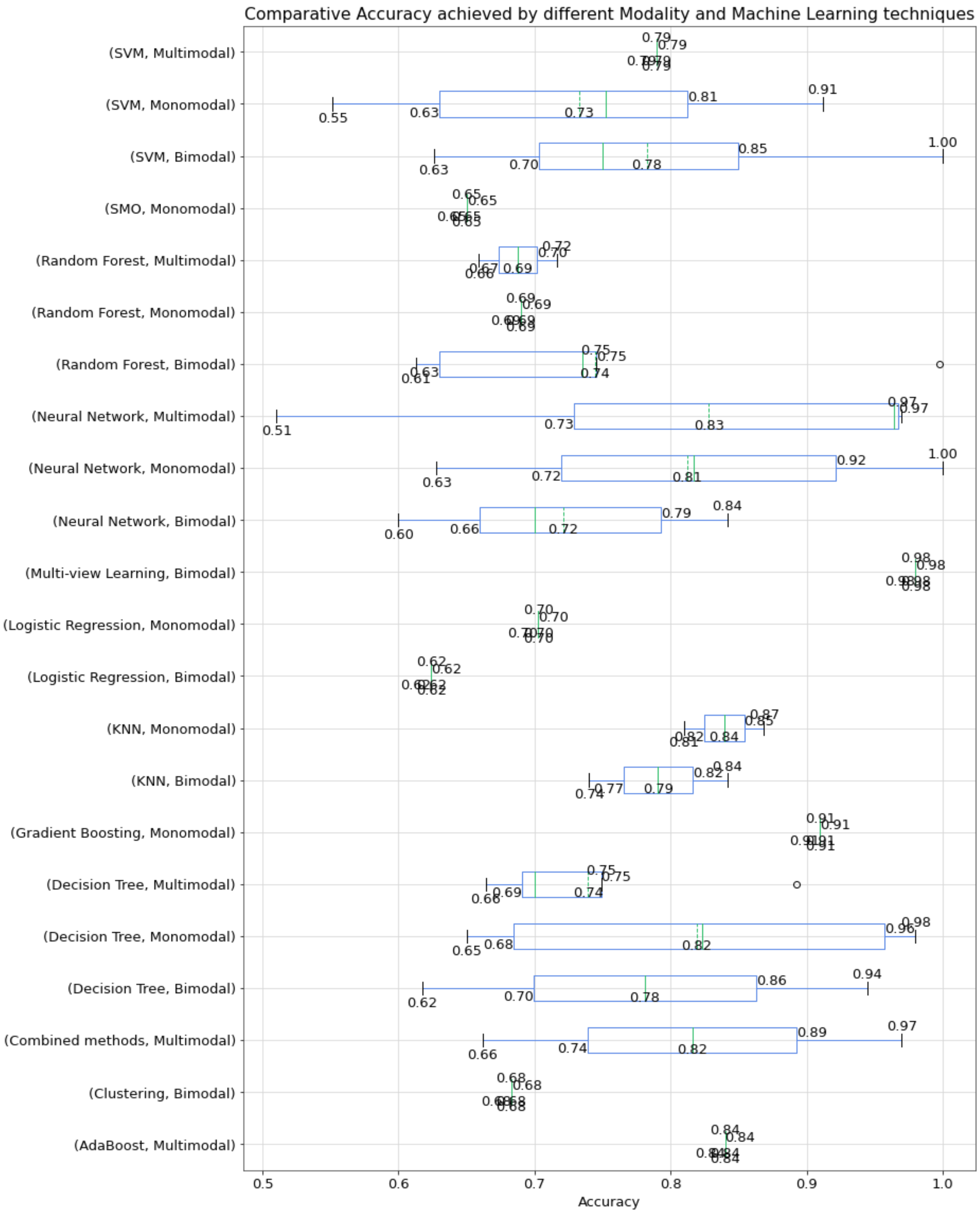
Comparative Accuracy achieved by different Modality and Machine Learning techniques
(SVM, Multimodal)
(SVM, Monomodal)
(SVM, Bimodal)
(SMO, Monomodal)
(Random Forest, Multimodal)
(Random Forest, Monomodal)
(Random Forest, Bimodal)
(Neural Network, Multimodal)
(Neural Network, Monomodal)
(Neural Network, Bimodal)
(Multi-view Learning, Bimodal)
(Logistic Regression, Monomodal)
(Logistic Regression, Bimodal)
(KNN, Monomodal)
(KNN, Bimodal)
(Gradient Boosting, Monomodal)
(Decision Tree, Multimodal)
(Decision Tree, Monomodal)
(Decision Tree, Bimodal)
(Combined methods, Multimodal)
(Clustering, Bimodal)
(AdaBoost, Multimodal)
0.5
0.6
0.7
0.8
0.9
1.0
Accuracy

8.7. Best results in studies (Accuracy ≥ 90%)

We decided to use the 90% accuracy criteria because it is the one used in [1].

[1] O'Sullivan M, Ekman P. **The wizards of deception detection**. The Detection of Deception in Forensic Contexts. 2004.

Studies that achieved accuracy greater than or equals to 0.9

	Year	Study	Modality	Acc/Tech	Features	Dataset
**40**	2018	Deception detection using artificial neural network and support vector machine (Srivastava, N.; Dubey, S.)	Bimodal (Physiological+Vocal)	1.0 / SVM	Blood pressure, Heart rate, MFCC, Respiration rate, Voice energy, Voice pitch, Zero-crossing	15-question questionnaire answered by 50 people (750 rows)
**70**	2021	Use of Machine Learning for Deception Detection From Spectral and Cepstral Features of Speech Signals (Ullah, Muhammad S.; Fernandes, Sinead V.)	Monomodal (Vocal)	1.0 / Neural Network	Delta cepstrum, Delta energy, Time difference cepstrum, Time difference energy	Three sessions of a police interrogation on a suspect. (12 rows)
**10**	2020	Automated Deception Detection of Males and Females from Non-Verbal Facial Micro-Gestures (Crockett, K.; OShea, J.; Khan, W.)	Bimodal (Demographical+Visual)	0.998 / Random Forest	Eye gaze, Facial micro-gestures, Gender, Head pose	Interview video recordings (86584 rows)
**5**	2018	A Multi-View Learning Approach To Deception Detection (Carissimi, Nicolo; Beyan, Cigdem; Murino, Vittorio)	Bimodal (Textual+Visual)	0.98 / Multi-view Learning	Bigrams, Eye gaze, Facial expressions, Hand motion, Head motion, Unigrams	Superset of Real-life Trial Deception Detection Dataset (121 rows)
**39**	2015	A comparison of features for automatic deception detection in synchronous computer-mediated communication (Pak, J.; Zhou, L.)	Monomodal (Textual)	0.98 / Decision Tree	LIWC categories, Syntax complexity, Unigrams	Communications during sessions of the online mafia game (142 rows)
**37**	2019	Robust Algorithm for Multimodal Deception Detection (Venkatesh, S.; Ramachandra, R.; Bours, P.)	Multimodal (Textual+Visual+Vocal)	0.97 / Combined methods	Body motion, Facial micro-expressions, MFCC, N-grams	Real-life Trial Deception Detection Dataset (121 rows)
**57**	2019	Face-Focused Cross-Stream Network for Deception Detection in Videos (Ding, M.; Zhao, A.; Lu, Z.; Xiang, T.; Wen, J.)	Multimodal (Textual+Visual+Vocal)	0.97 / Neural Network	Facial expressions, Head motion, Spectral parameters, Unigrams	Real-life Trial Deception Detection Dataset (121 rows)
**75**	2021	LieNet: A Deep Convolution Neural Networks Framework for Detecting Deception (Karnati, Mohan; Seal, Ayan; Yazidi, Anis; Krejcar, Ondrej)	Multimodal (Physiological+Visual+Vocal)	0.967375 / Neural Network	EEG channels, Face image, Voice signal	Combination of three deception detection datasets (766 rows)
**6**	2017	Deep Learning Driven Multimodal Fusion For Automated Deception Detection (Gogate, Mandar; Adeel, Ahsan; Hussain, Amir)	Multimodal (Textual+Visual+Vocal)	0.964 / Neural Network	Facial expressions, GloVe embeddings, Hand motion, INTERSPEECH 2013	Superset of Real-life Trial Deception Detection Dataset (121 rows)
**63**	2018	Lie Detector With The Analysis Of The Change Of Diameter Pupil and The Eye Movement Use Method Gabor Wavelet Transform and Decision Tree (Labibah, Z.; Nasrun, M.; Setianingsih, C.)	Monomodal (Visual)	0.95 / Decision Tree	Eye gaze, Pupil dilation	Questionnaire answered by volunteers (40 rows)
**30**	2012	The Voice and Eye Gaze Behavior of an Imposter: Automated Interviewing and Detection for Rapid Screening at the Border (Elkins, Aaron C.; Derrick, Douglas C.; Gariup, Monica)	Bimodal (Thermal+Vocal)	0.9447 / Decision Tree	Eye gaze, Pupil dilation, Voice energy, Voice pitch, Voice quality	Participants of an experiment in UE border control (259 rows)
**65**	2020	Building a Better Lie Detector with BERT: The Difference Between Truth and Lies (Barsever, D.; Singh, S.; Neftci, E.)	Monomodal (Textual)	0.936 / Neural Network	BERT embeddings	Ott Deceptive Opinion Spam Corpus (1600 rows)
**69**	2021	Development of Spectral Speech Features for Deception Detection Using Neural Networks (Ullah, Muhammad S.; Fernandes, Sinead V.)	Monomodal (Vocal)	0.9167 / Neural Network	Bark	Three sessions of a police interrogation on a suspect. (12 rows)
**25**	2012	Syntactic Stylometry for Deception Detection (Feng, Song; Banerjee, Ritwik; Choi, Yejin)	Monomodal (Textual)	0.912 / SVM	Bigrams, POS tags, Syntax complexity, Unigrams	Reviews of 35 Italian restaurants (2692 rows)
**64**	2014	Cues to Deception in Social Media Communications (Briscoe, E. J.; Appling, D. S.; Hayes, H.)	Monomodal (Textual)	0.91 / Gradient Boosting	Emoticons, Informality, Sentiment, Syntax complexity	Statements provided by volunteers in a mock chat room (254 rows)
