# Supplementary material for: Deception detection with machine learning: A systematic review and statistical analysis
Source: PLoS One. 2023 Feb 9;18(2):e0281323. doi: 10.1371/journal.pone.0281323 (PMC9910662; doi:10.1371/journal.pone.0281323)
Supplement: S7 File — Source: The authors (2022). (PDF) [file pone.0281323.s007.pdf]

Deception Detection supported by Machine Learning

Literature Review - Mind maps

This notebook is devoted to show all the mind maps built as a summary of all the selected studies.

Mind maps are ascendingly sorted by publishing year and title. Each section shows the article title, the bibliographic reference and the mind map extracted during the full text reading.

1. Move, and I will tell you who you are: Detecting deceptive roles in low-quality data

Raiman N, Hung H, Englebienne G. Move, and I will tell you who you are: Detecting deceptive roles in low-quality data. ICMI'11 - Proc 2011 ACM Int Conf Multimodal Interact. 2011; 201-4.

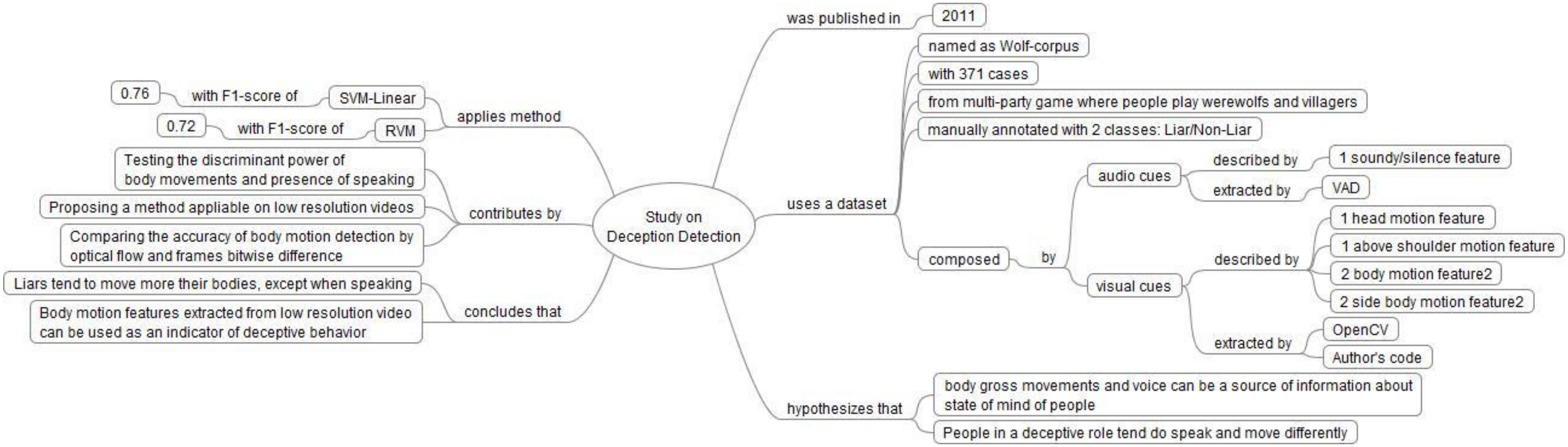

2. Challenges in automated deception detection in computer-mediated communication

Rubin V. L., Conroy N. J. Challenges in automated deception detection in computer-mediated communication. Proc ASIST Annu Meet. 2011; 48.

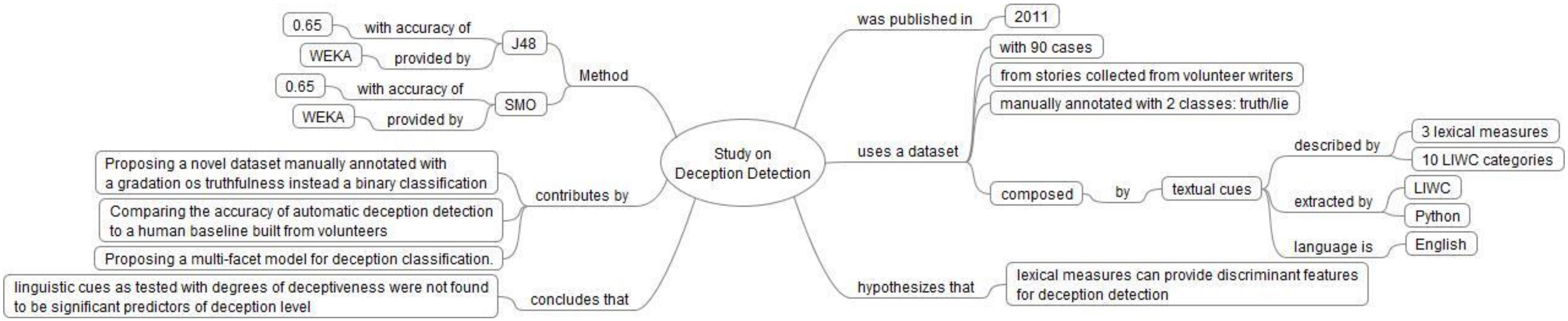

3. The Voice and Eye Gaze Behavior of an Imposter: Automated Interviewing and Detection for Rapid Screening at the Border

Elkins A. C. , Derrick D. C. , Gariup M. The Voice and Eye Gaze Behavior of an Imposter: Automated Interviewing and Detection for Rapid Screening at the Border. Conf Eur Chapter Assoc Comput Linguist. 2012; 49–54.

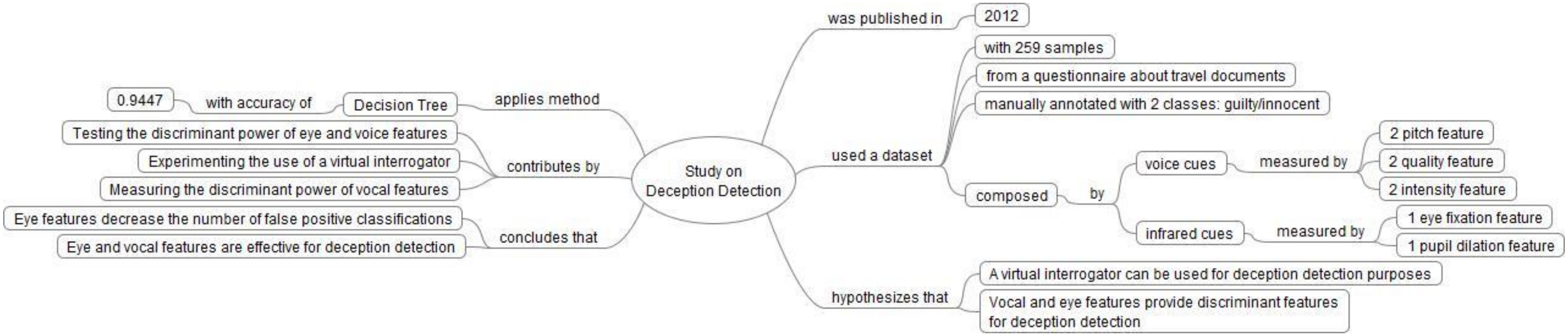

4. Syntactic Stylometry for Deception Detection

Feng S., Banerjee R., Choi Y. Syntactic stylometry for deception detection. 50th Annu Meet Assoc Comput Linguist ACL 2012 - Proc Conf. 2012; 2(July):171–5.

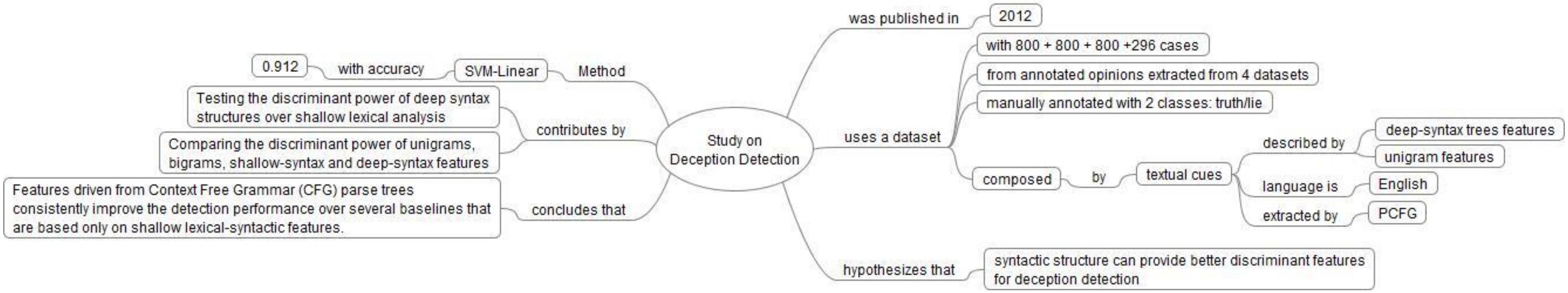

5. On the Use of Homogenous Sets of Subjects in Deceptive Language Analysis

Fornaciari T., Poesio M. On the use of homogenous sets of subjects in deceptive language analysis. Comput Linguist Proc Work Comput Approaches to Decept Detect. 2012; 39–47.

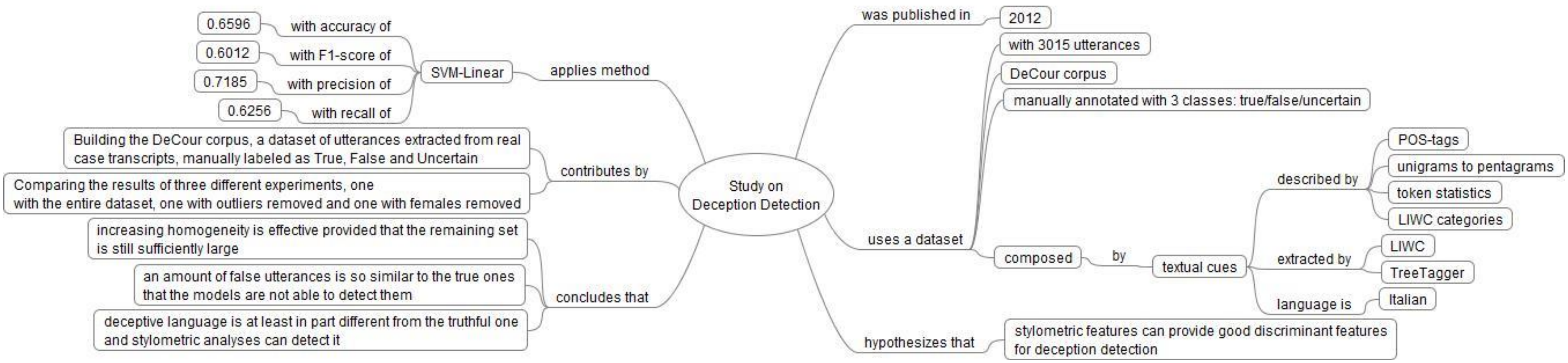

6. Discerning truth from deception: Human judgments and automation efforts

Rubin V. L., Conroy N. Discerning truth from deception: Human judgments and automation efforts. First Monday. 2012; 17(3).

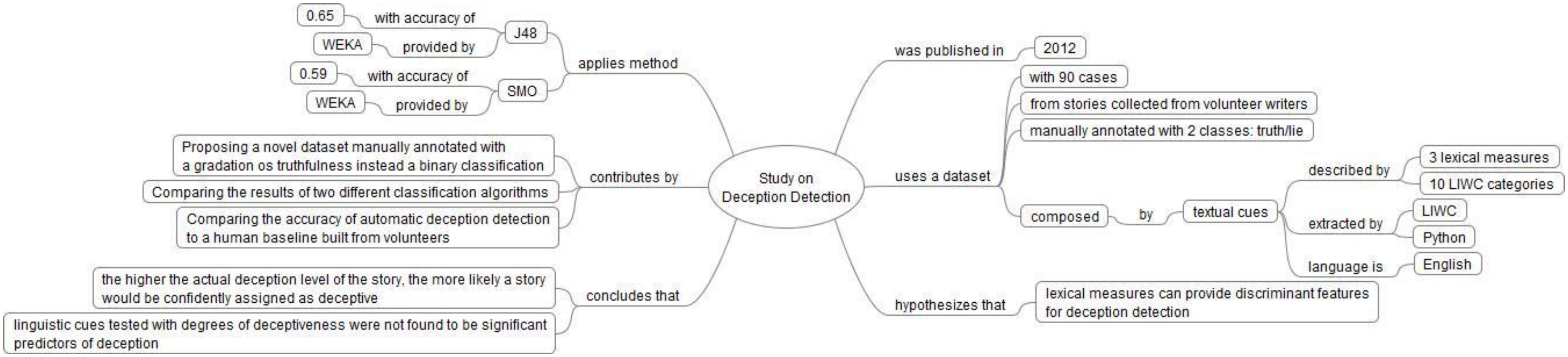

7. Seeing through Deception: A Computational Approach to Deceit Detection in Spanish Written Communication

Almela Á., Valencia-García R., Cantos P. Seeing through Deception: A Computational Approach to Deceit Detection in Spanish Written Communication. Linguist Evid Secur Law Intell. 2013; 1(1):3–12.

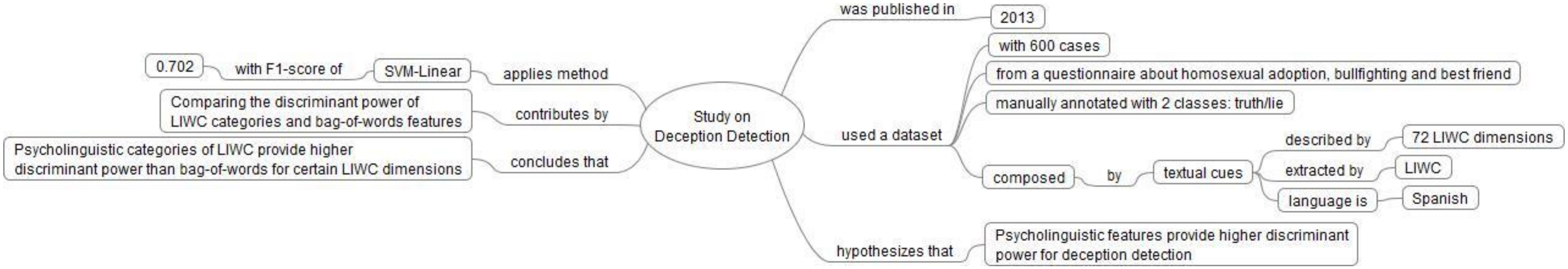

8. Automatic detection of deceit in verbal communication

Mihalcea R., Pérez-Rosas V., Burzo M. Automatic detection of deceit in verbal communication. ICMI 2013 - Proc 2013 ACM Int Conf Multimodal Interact. 2013; 131–4.

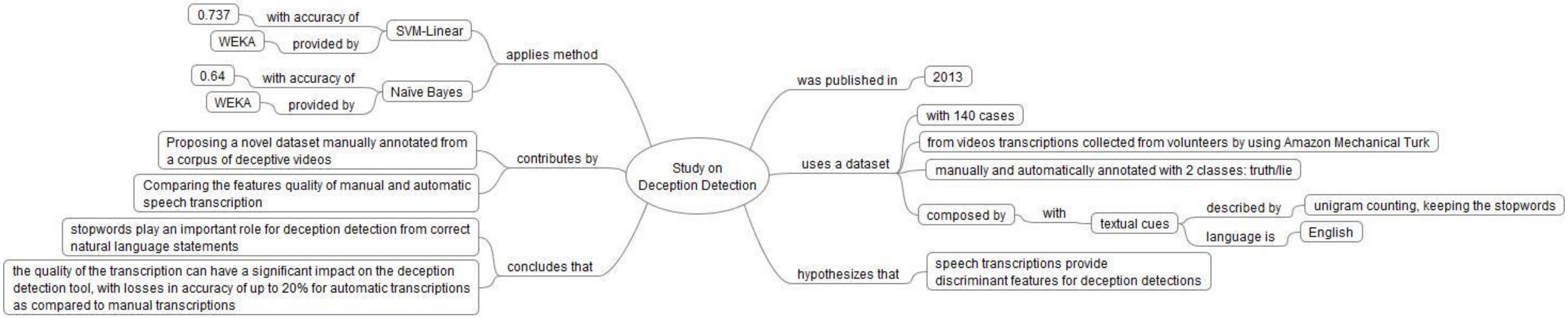

9. Deception detection in speech using bark band and perceptually significant energy features

Sanaullah M, Gopalan K. Deception detection in speech using bark band and perceptually significant energy features. Midwest Symp Circuits Syst. 2013; 1212–5.

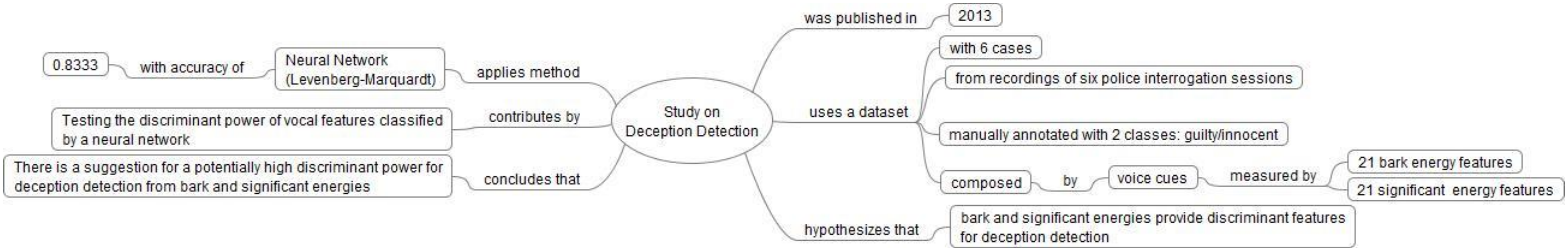

10. Deception Detection Using a Multimodal Approach

Abouelenien M., Pérez-Rosas V., Mihalcea R., Burzo M. Deception detection using a multimodal approach. ICMI 2014 - Proc 2014 Int Conf Multimodal Interact. 2014; 58–65.

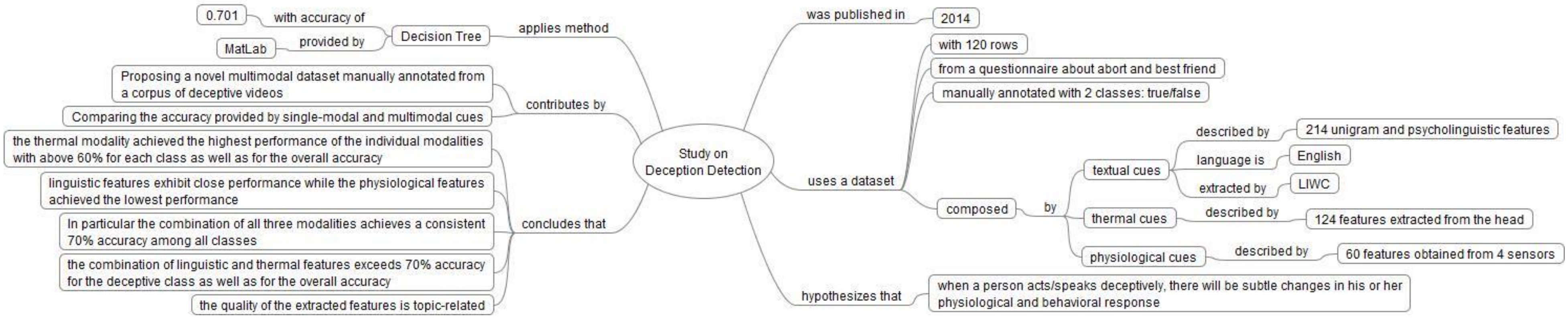

11. Cues to deception in social media communications

Briscoe E. J., Appling D. S., Hayes H. Cues to deception in social media communications. Proc Annu Hawaii Int Conf Syst Sci. 2014; 1435-43.

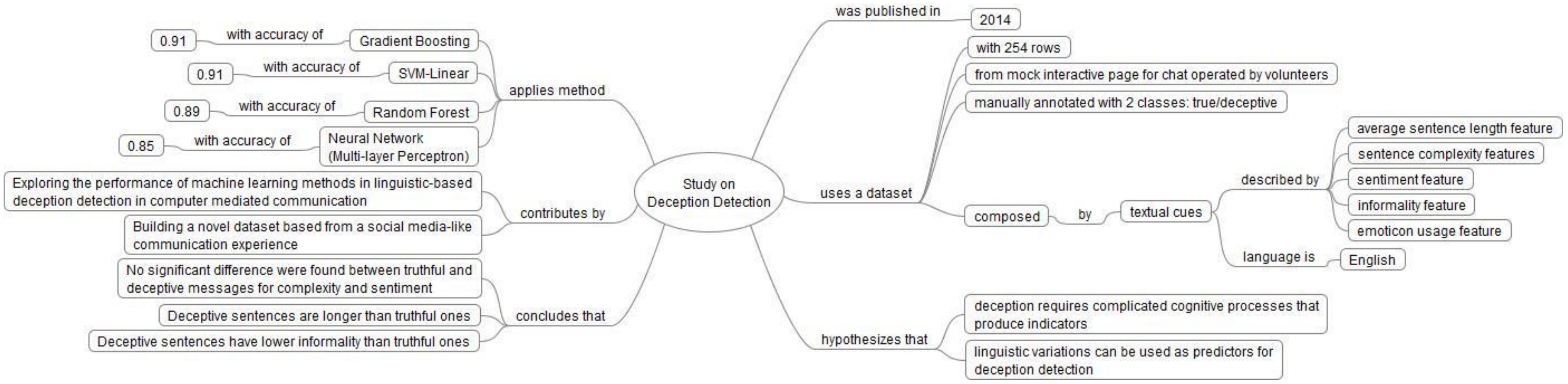

12. Thermal Facial Analysis for Deception Detection

Rajoub B. A., Zwiggelaar R. Thermal Facial Analysis for Deception Detection. IEEE Trans Inf Forensics Secur. 2014; 9(6):1015-23.

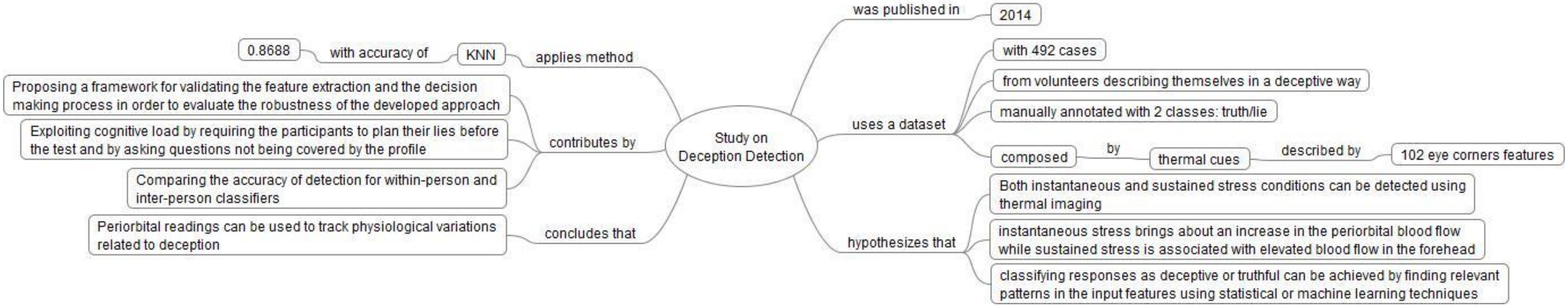

13. Detection of deception in the Mafia party game

Bailey J., Demyanov S., Ramamohanarao K., Leckie C. Detection of deception in the Mafia party game. ICMI 2015 - Proc 2015 ACM Int Conf Multimodal Interact. 2015; 335-42.

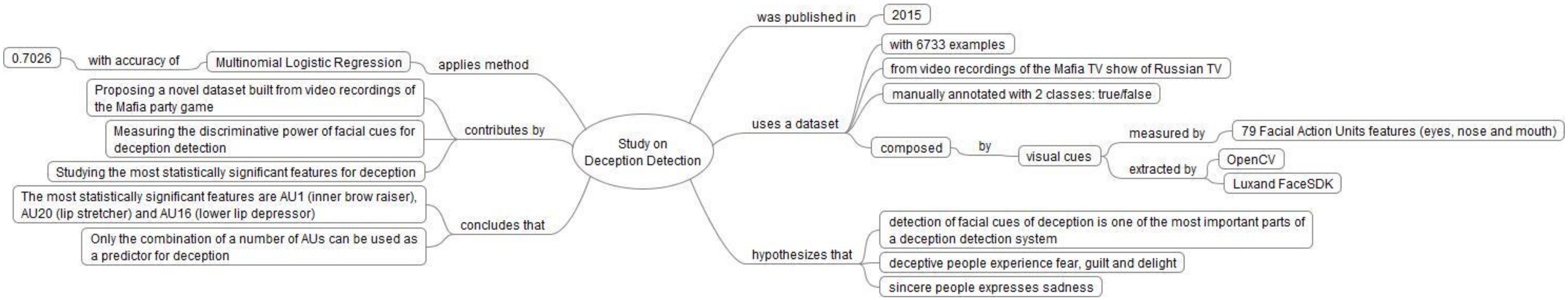

14. Perinasal indicators of deceptive behavior

Dcosta M., Shastri D., Vilalta R., Burgoon J. K., Pavlidis I. Perinasal indicators of deceptive behavior. 2015 11th IEEE Int Conf Work Autom Face Gesture Recognition, FG 2015. 2015;

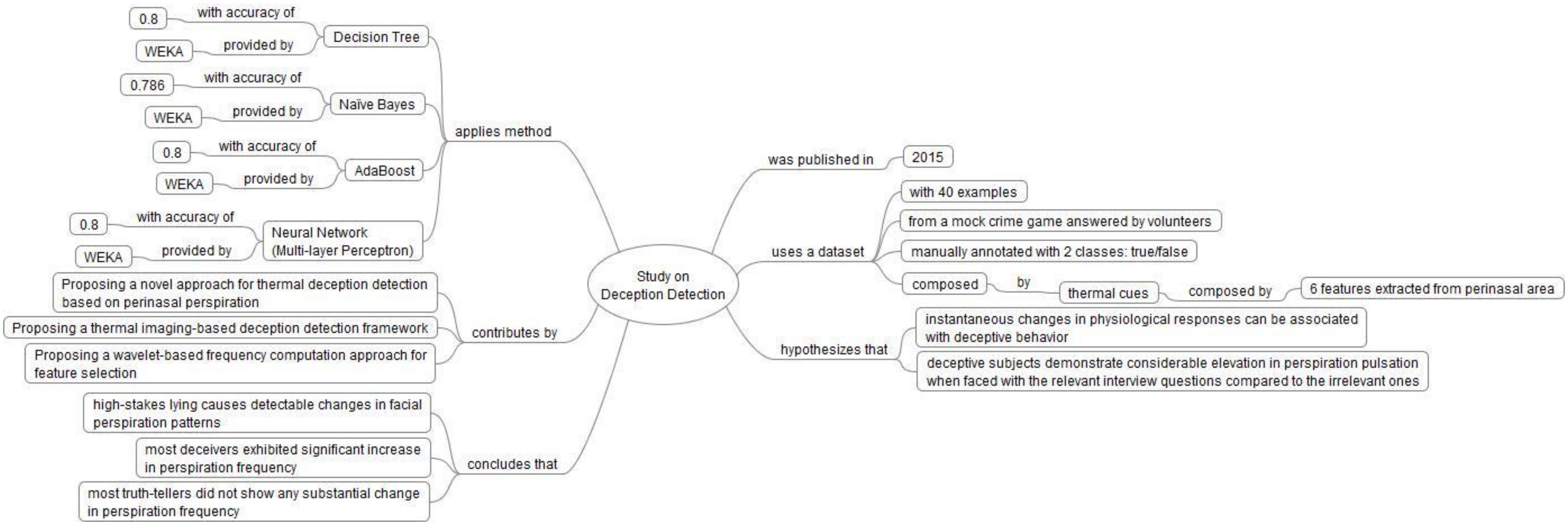

15. Distinguishing deception from non-deception in Chinese speech

Fan C., Zhao H., Chen X., Fan X., Chen S. Distinguishing deception from non-deception in Chinese speech. Proc 6th Int Conf Intell Control Inf Process ICICIP 2015. 2016; 268–73.

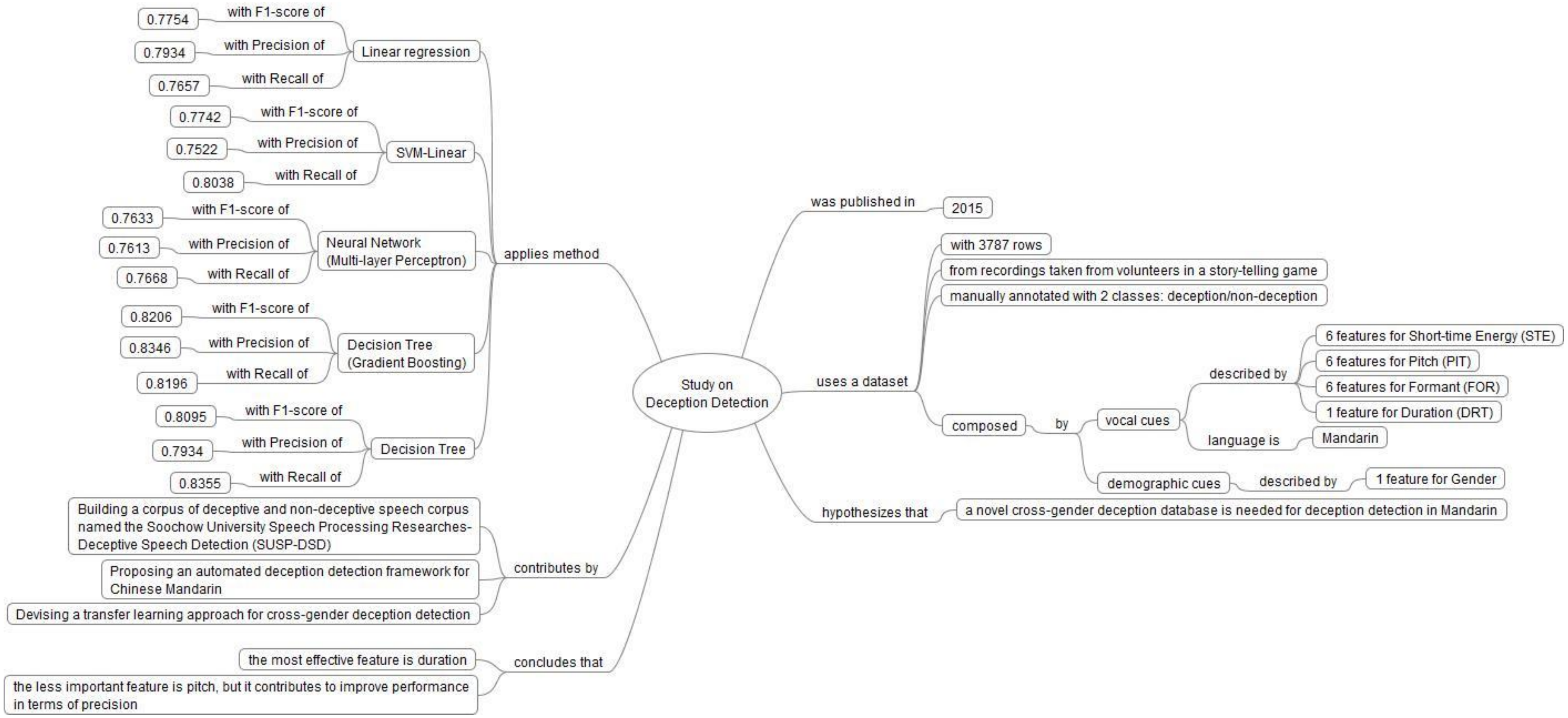

16. Cross-cultural production and detection of deception from speech

Levitan S. I., An G., Wang M., Mendels G., Hirschberg J., Levine M., et al. Cross-cultural production and detection of deception from speech. WMDD 2015 - Proc ACM Work Multimodal Decept Detect co-located with ICMI 2015. 2015; 1–8.

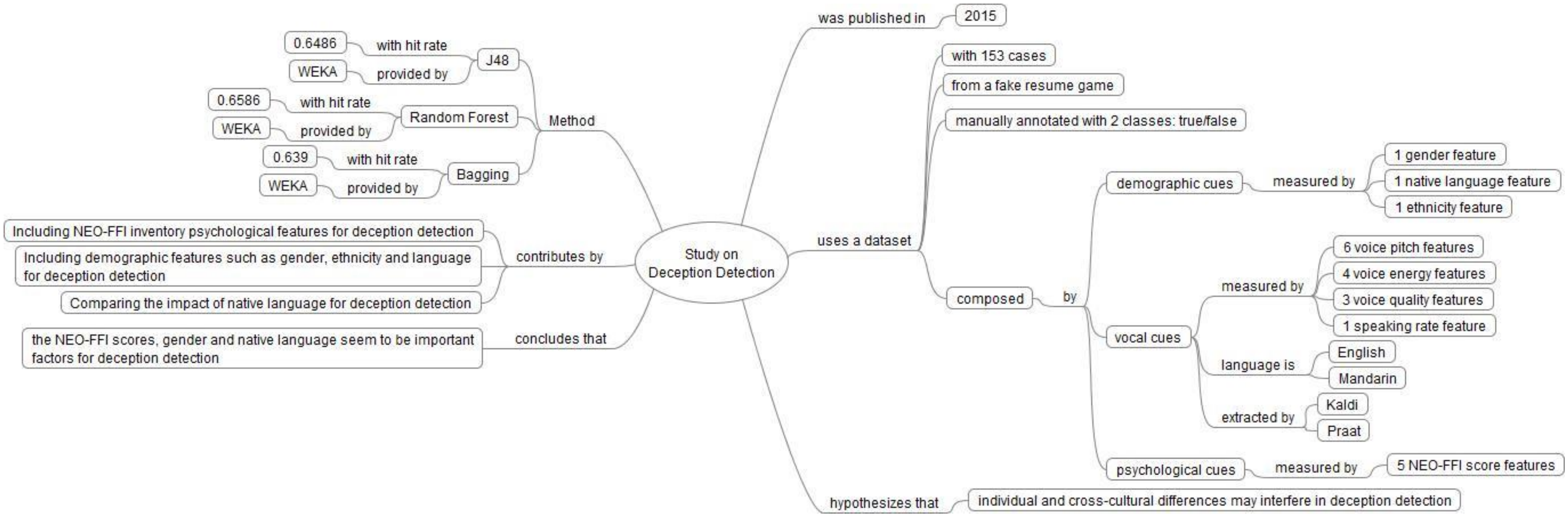

17. A comparison of features for automatic deception detection in synchronous computer-mediated communication

Pak J., Zhou L. A comparison of features for automatic deception detection in synchronous computer-mediated communication. 2015 IEEE Int Conf Intell Secur Informatics Secur World through an Alignment Technol Intell Humans Organ ISI 2015. 2015; 141–3.

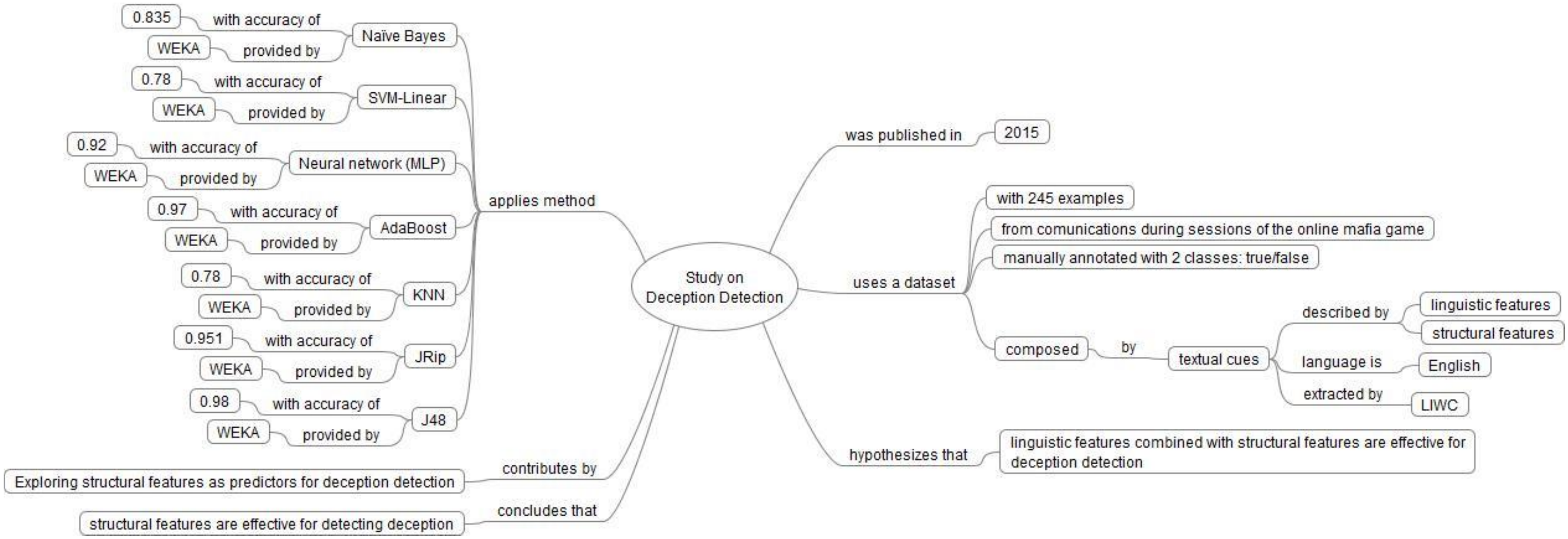

18. Deception detection using real-life trial data

Pérez-Rosas V., Abouelenien M., Mihalcea R., Burzo M. Deception detection using real-life trial data. ICMI 2015 - Proc 2015 ACM Int Conf Multimodal Interact. 2015; 59-66.

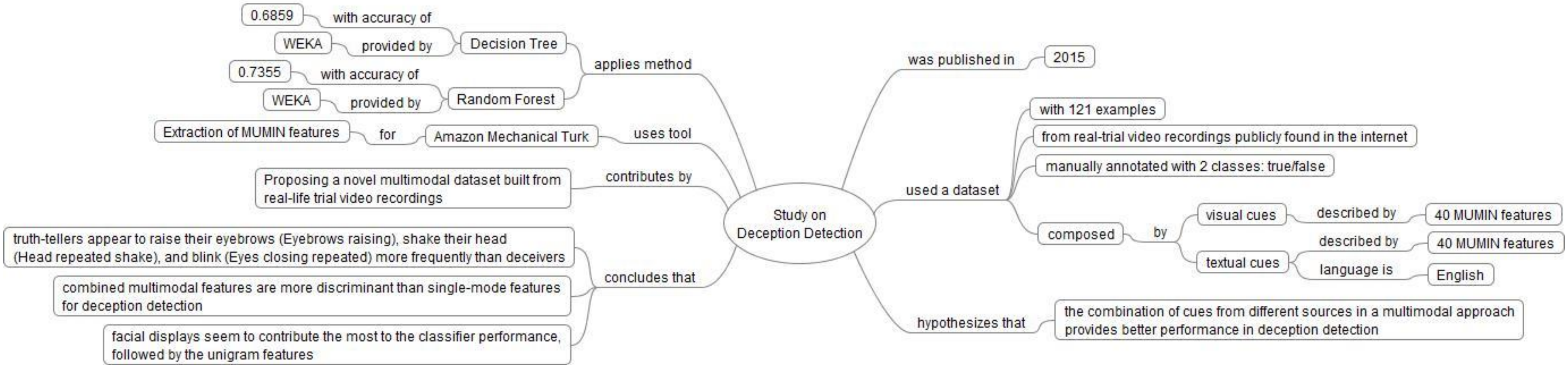

19. Experiments in open domain deception detection

Pérez-Rosas V., Mihalcea R. Experiments in open domain deception detection. Conf Proc - EMNLP 2015 Conf Empir Methods Nat Lang Process. 2015; (September):1120-5.

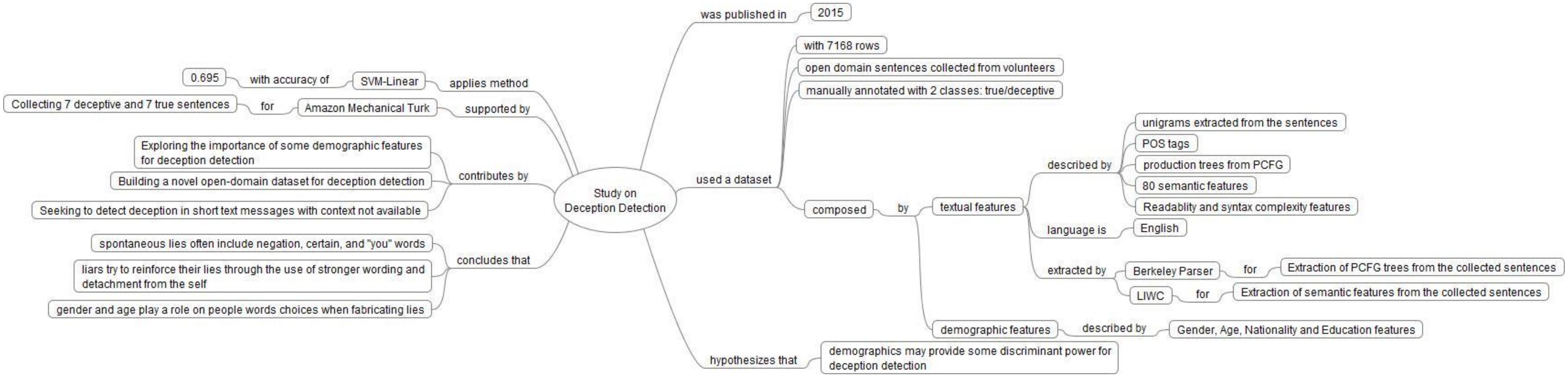

20. Is interactional dissynchrony a clue to deception? Insights from automated analysis of nonverbal visual cues

Yu X., Zhang S., Yan Z., Yang F., Huang J., Dunbar N. E., et al. Is interactional dissynchrony a clue to deception? Insights from automated analysis of nonverbal visual cues. IEEE Trans Cybern. 2015; 45(3):492-506.

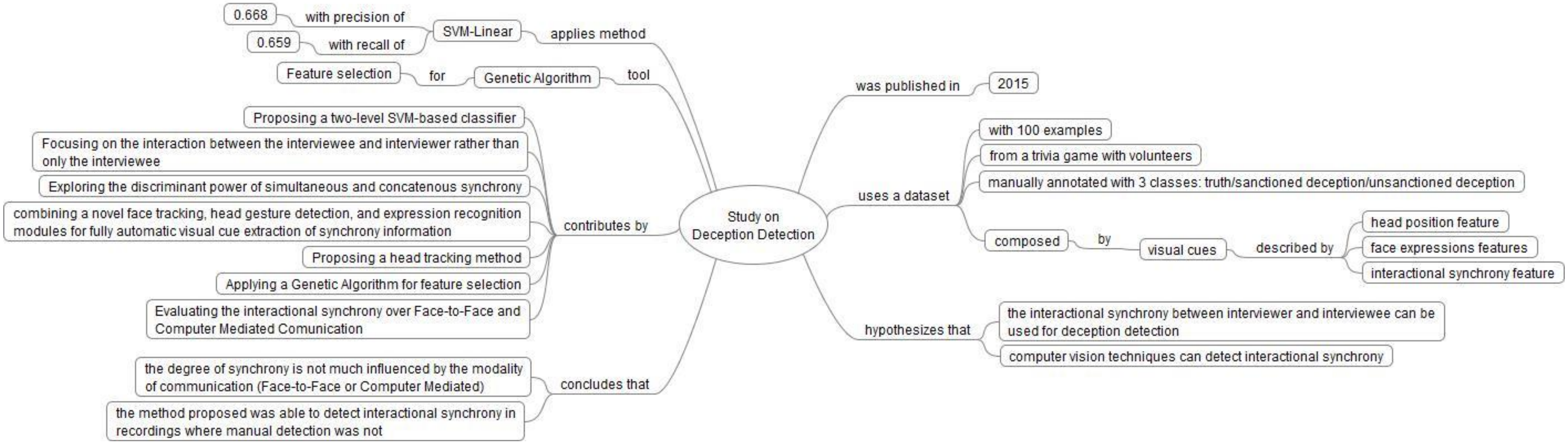

21. Analyzing thermal and visual clues of deception for a non-contact deception detection approach

Abouelenien M., Mihalcea R., Burzo M. Analyzing thermal and visual clues of deception for a non-contact deception detection approach. ACM Int Conf Proceeding Ser. 2016; 29-June-20:1–4.

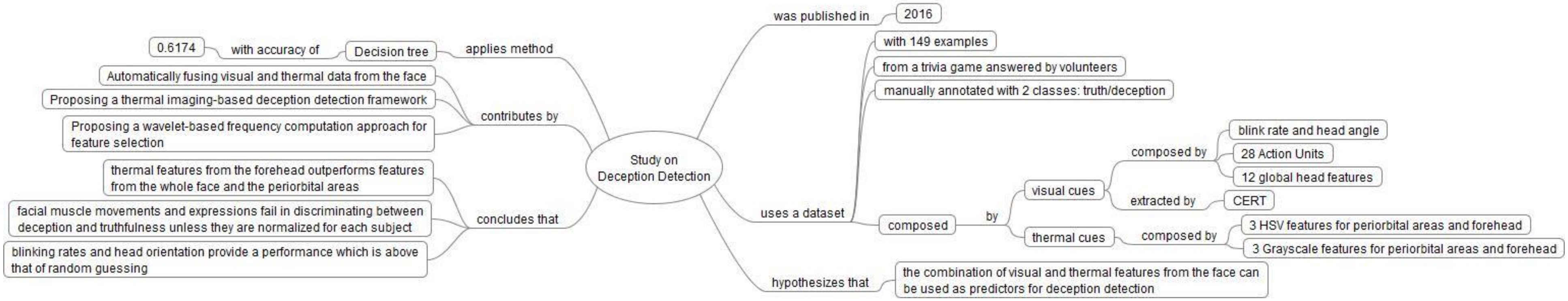

22. Deceptive Speech Detection based on sparse representation

Fan X., Zhao H., Chen X., Fan C., Chen S. Deceptive Speech Detection based on sparse representation. Proceeding - 2016 IEEE 12th Int Colloq Signal Process its Appl CSPA 2016. 2016; (March):7–11.

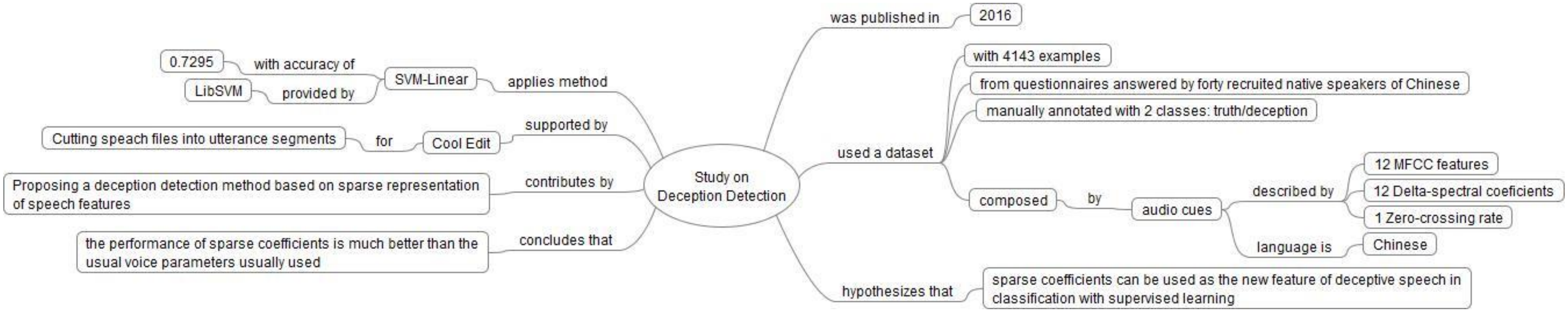

23. The Truth and Nothing but the Truth: Multimodal Analysis for Deception Detection

Jaiswal M., Tabibu S., Bajpai R. The Truth and Nothing but the Truth: Multimodal Analysis for Deception Detection. IEEE Int Conf Data Min Work ICDMW. 2016; 0:938-43.

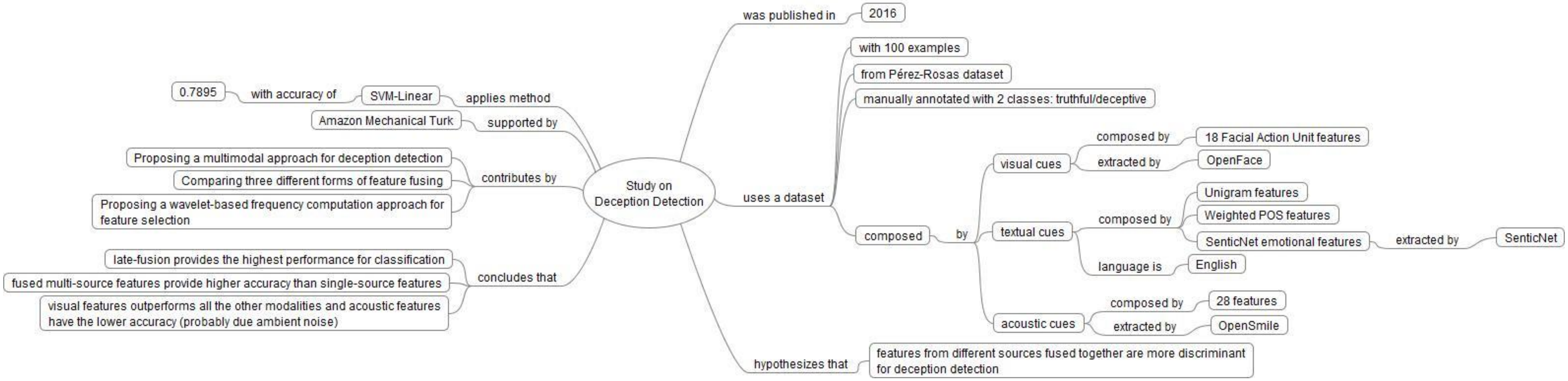

24. ReLiDSS: Novel lie detection system from speech signal

Nasri H., Ouarda W., Alimi A.M. ReLiDSS: Novel lie detection system from speech signal. Proc IEEE/ACS Int Conf Comput Syst Appl AICCSA. 2016.

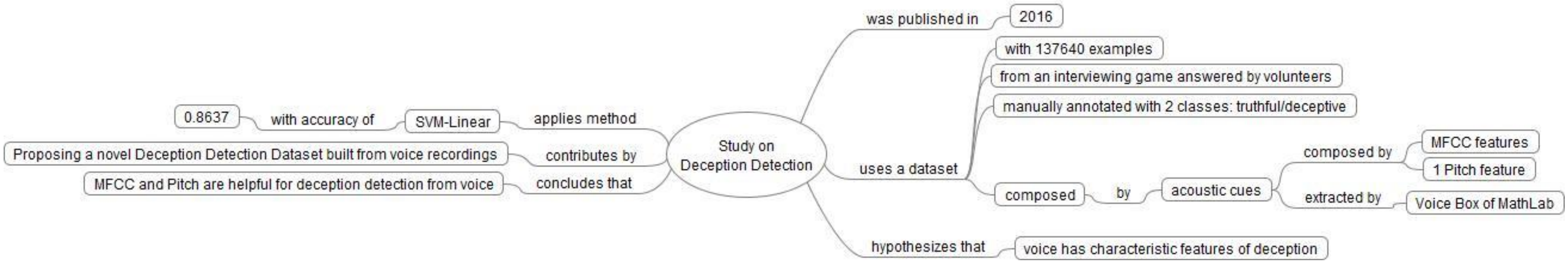

25. Automated detection of user deception in on-line questionnaires with focus on eye tracking use

Rybar M., Bielikova M. Automated detection of user deception in on-line questionnaires with focus on eye tracking use. Proc - 11th Int Work Semant Soc Media Adapt Pers SMAP 2016. 2016; (i):24–8.

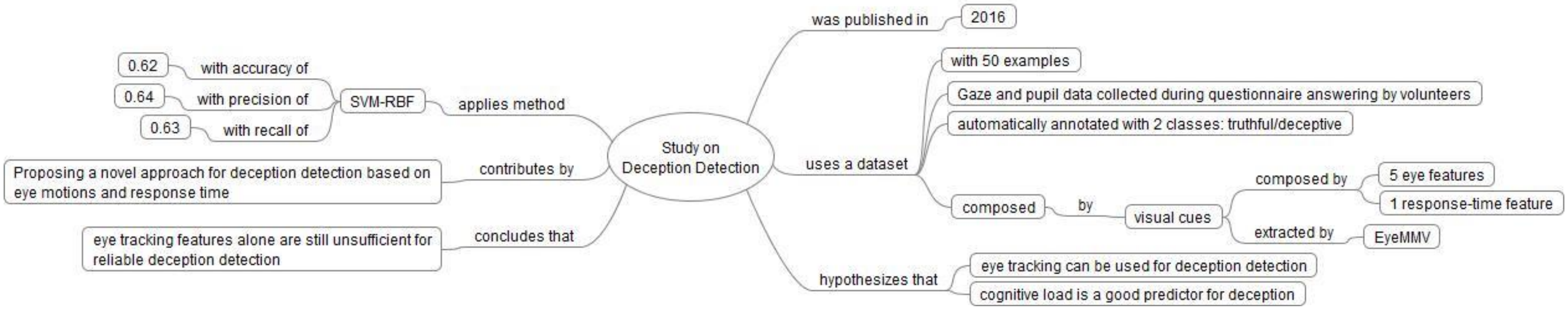

26. Detecting Deceptive Behavior via Integration of Discriminative Features from Multiple Modalities

Abouelenien M., Pérez-Rosas V., Mihalcea R., Burzo M. Detecting Deceptive Behavior via Integration of Discriminative Features from Multiple Modalities. IEEE Trans Inf Forensics Secur. 2017; 12(5):1042–55.

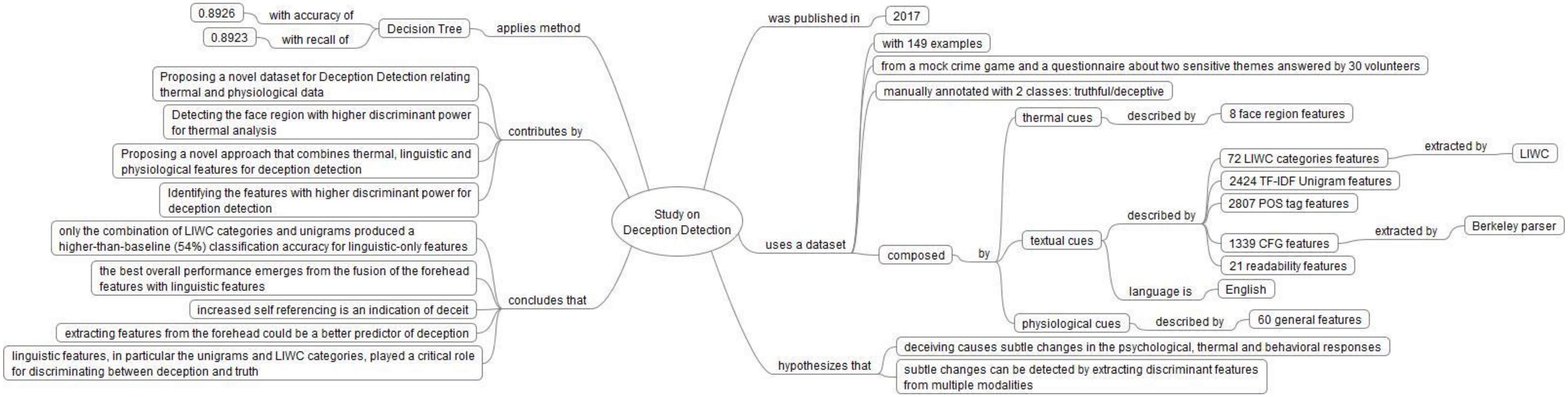

27. Gender-based multimodal deception detection

Abouelenien M., Pérez-Rosas V., Zhao B., Mihalcea R., Burzo M. Gender-based multimodal deception detection. Proc ACM Symp Appl Comput. 2017; Part F1280:137–44.

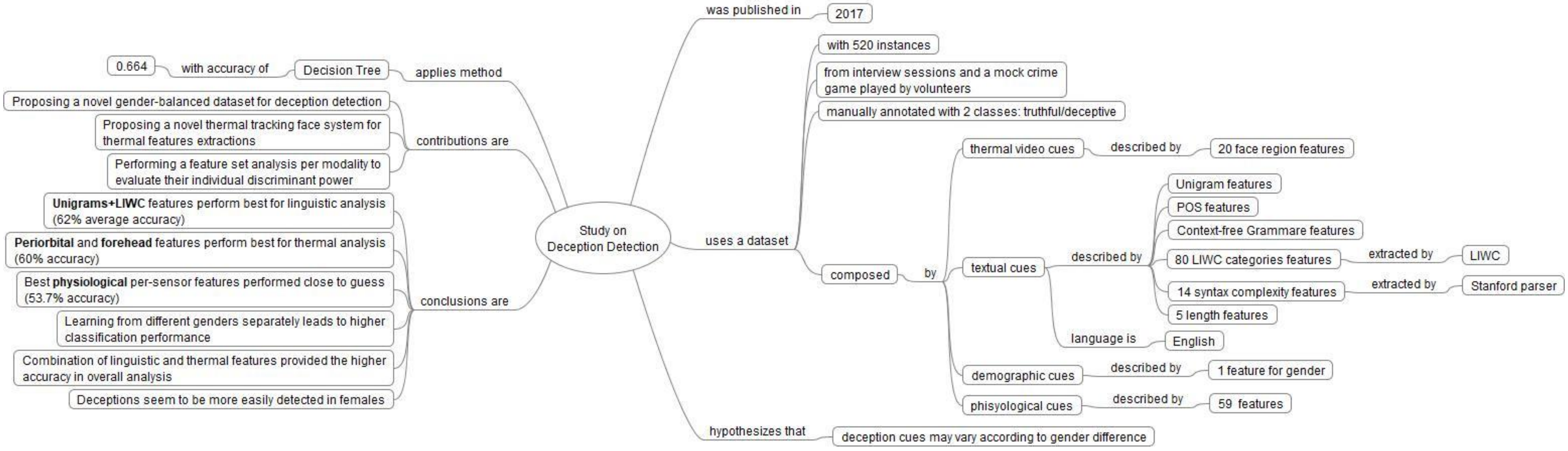

28. Deep learning driven multimodal fusion for automated deception detection

Gogate M., Adeel A., Hussain A. Deep learning driven multimodal fusion for automated deception detection. 2017 IEEE Symp Ser Comput Intell SSCI 2017 - Proc. 2018; 2018-Janua:1-6.

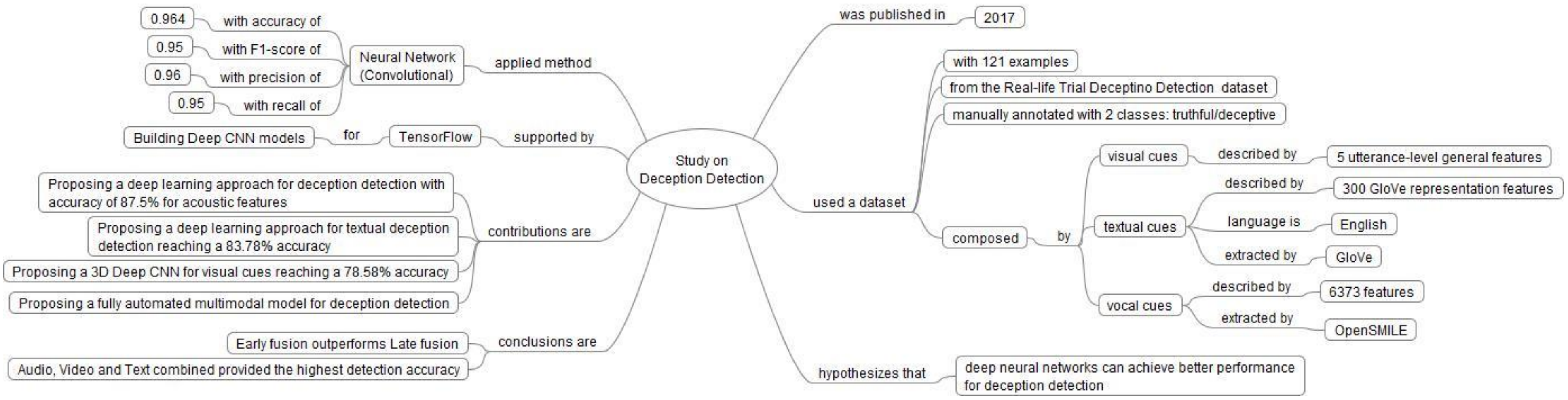

29. Deception detection in Russian texts

Litvinova O., Litvinova T., Seredin P., Lyell J. Deception detection in Russian texts. 15th Conf Eur Chapter Assoc Comput Linguist EACL 2017 - Proc Student Res Work. 2017; 43-52.

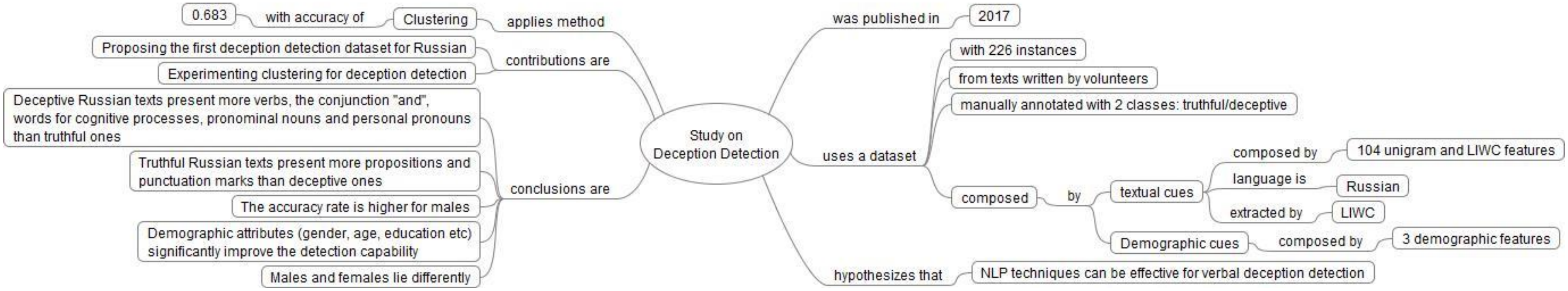

30. Hybrid acoustic-lexical deep learning approach for deception detection

Mendels G., Levitan S. I., Lee K. Z., Hirschberg J. Hybrid acoustic-lexical deep learning approach for deception detection. Proc Annu Conf Int Speech Commun Assoc INTERSPEECH. 2017; 2017-Augus:1472–6.

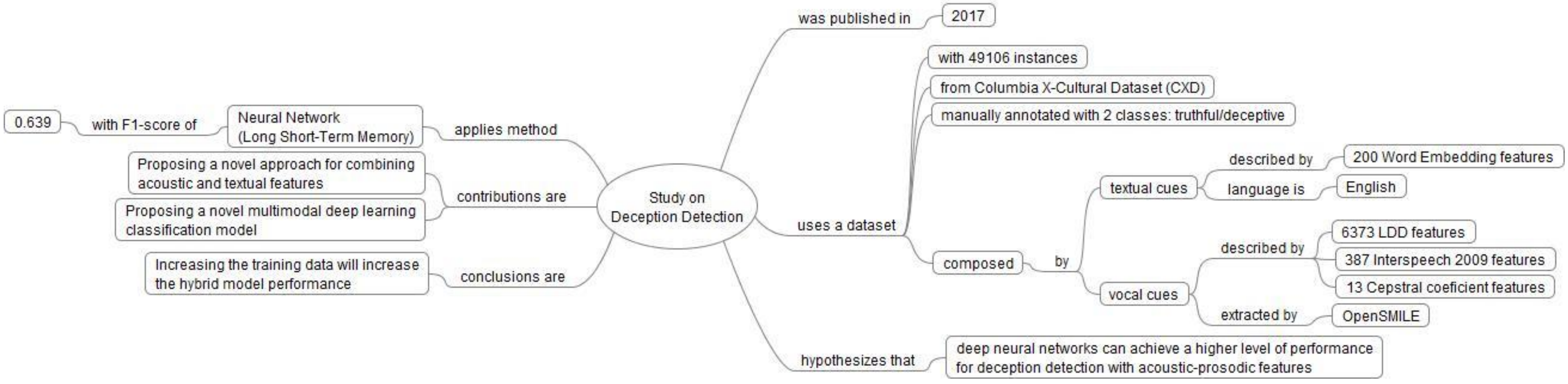

31. Construction and analysis of Indonesian-interviews deception corpus

Warnita T., Lestari D. P. Construction and analysis of Indonesian-interviews deception corpus. 2017 20th Conf Orient Chapter Int Comm Coord Stand Speech Databases Assess Tech O-COCOSDA 2017. 2018; (November):1–6.

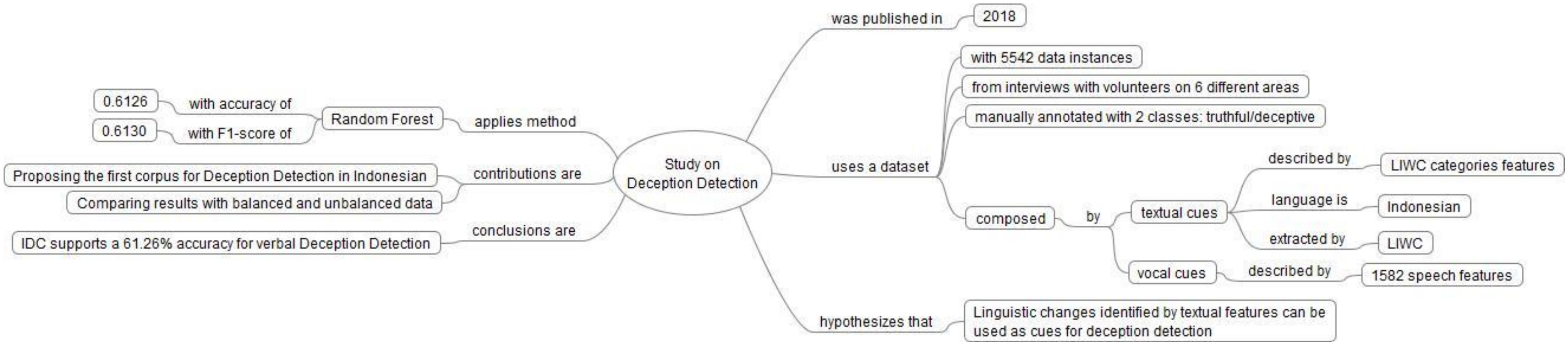

32. A multi-view learning approach to deception detection

Carissimi N., Beyan C., Murino V. A multi-view learning approach to deception detection. Proc - 13th IEEE Int Conf Autom Face Gesture Recognition, FG 2018. 2018; 599–606.

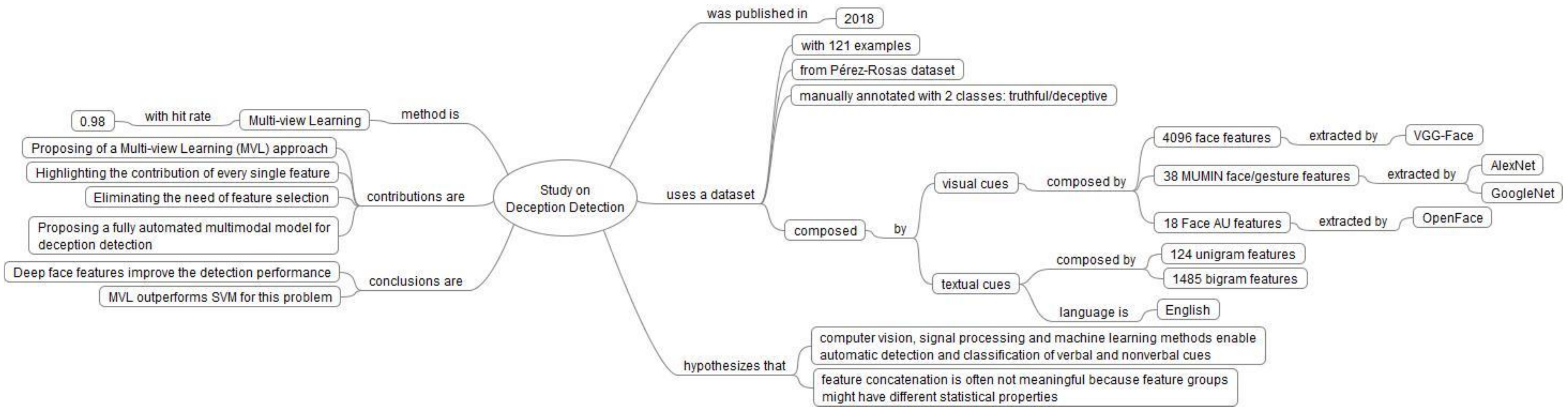

33. Deception Detection and Analysis in Spoken Dialogues based on FastText

Hosomi N., Sakti S., Yoshino K., Nakamura S. Deception Detection and Analysis in Spoken Dialogues based on FastText. 2018 Asia-Pacific Signal Inf Process Assoc Annu Summit Conf APSIPA ASC 2018 - Proc. 2019; (November):139–42.

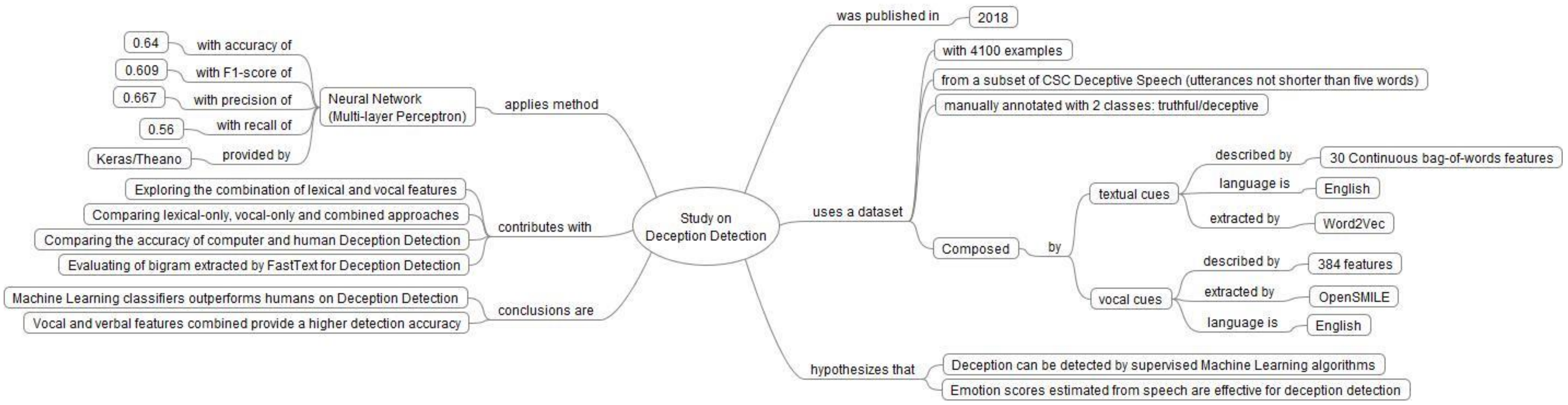

34. Interpretable multimodal deception detection in videos

Karimi H. Interpretable multimodal deception detection in videos. ICMI 2018 - Proc 2018 Int Conf Multimodal Interact. 2018; 511-5.

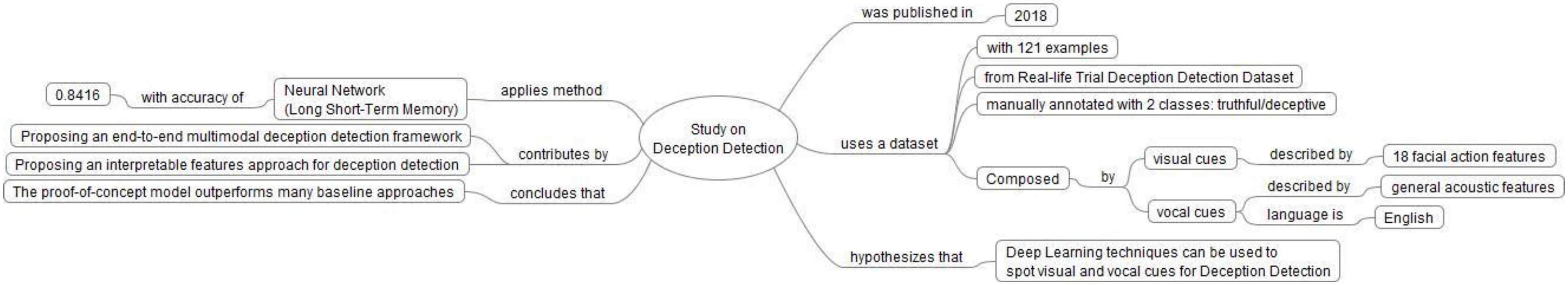

35. Toward End-to-End Deception Detection in Videos

Karimi H., Tang J., Li Y. Toward End-to-End Deception Detection in Videos. Proc - 2018 IEEE Int Conf Big Data, Big Data 2018. 2019; (c):1278-83.

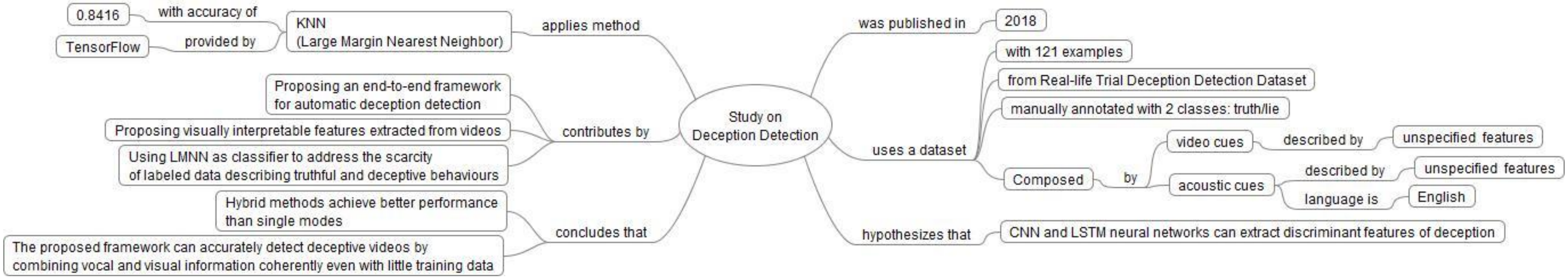

36. Automated verbal credibility assessment of intentions: The model statement technique and predictive modeling

Kleinberg B., van der Toolen Y., Vrij A., Arntz A., Verschuere B. Automated verbal credibility assessment of intentions: The model statement technique and predictive modeling. Appl Cogn Psychol. 2018; 32(3):354-66.

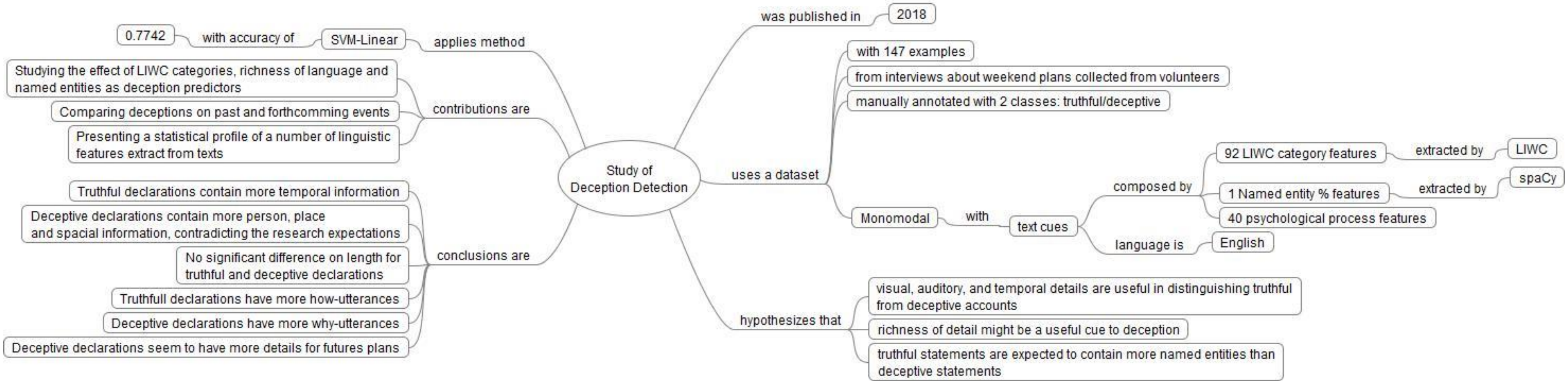

37. Lie Detector With The Analysis Of The Change Of Diameter Pupil and The Eye Movement

Labibah Z., Nasrun M., Setianingsih C. Lie Detector With The Analysis Of The Change Of Diameter Pupil and The Eye Movement. 2018;214–20.

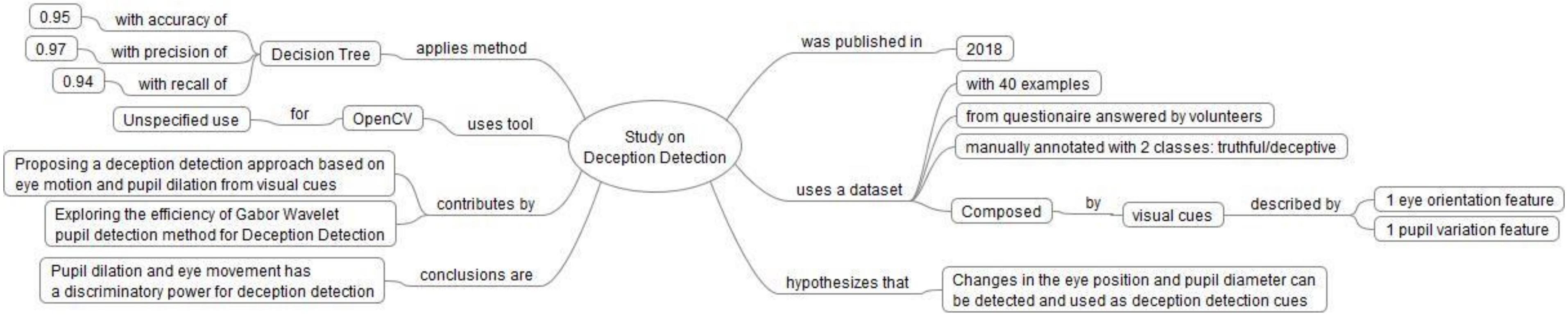

38. Acoustic-prosodic indicators of deception and trust in interview dialogues

Levitan S. I., Maredia A., Hirschberg J. Acoustic-prosodic indicators of deception and trust in interview dialogues. Proc Annu Conf Int Speech Commun Assoc INTERSPEECH. 2018; 2018-Septe:416–20.

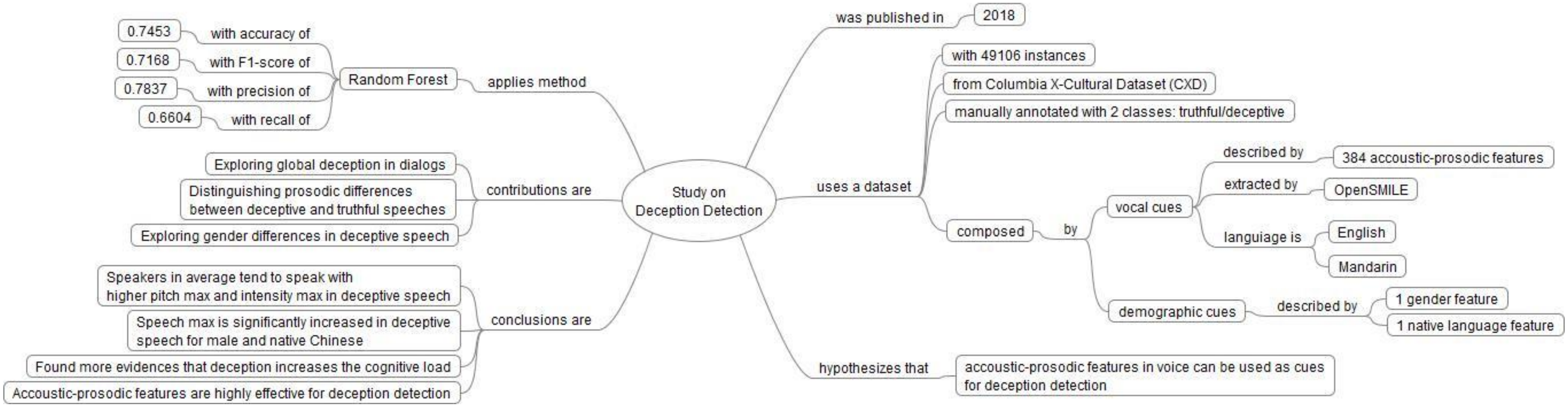

39. Linguistic cues to deception and perceived deception in interview dialogues

Levitan S. I., Maredia A., Hirschberg J. Linguistic cues to deception and perceived deception in interview dialogues. NAACL HLT 2018 - 2018 Conf North Am Chapter Assoc Comput Linguist Hum Lang Technol - Proc Conf. 2018; 1:1941–50.

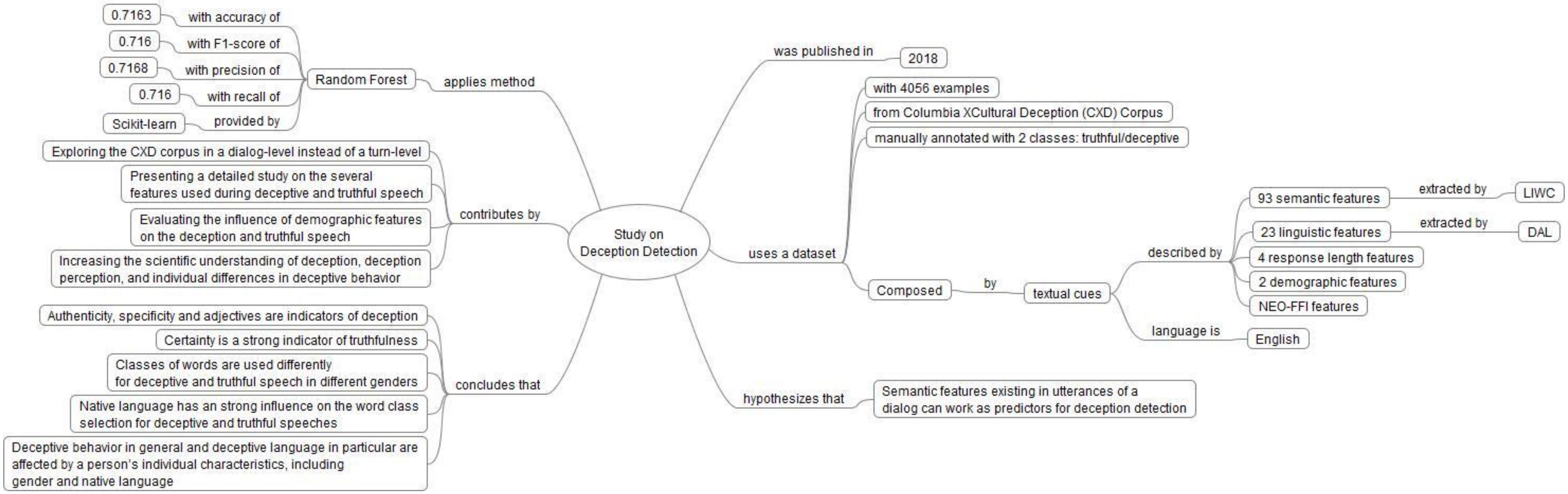

40. An empirical study on detecting deception and cybercrime using artificial neural networks

Mbaziira A. V., Murphy D. R. An empirical study on detecting deception and cybercrime using artificial neural networks. ACM Int Conf Proceeding Ser. 2018; 42–6.

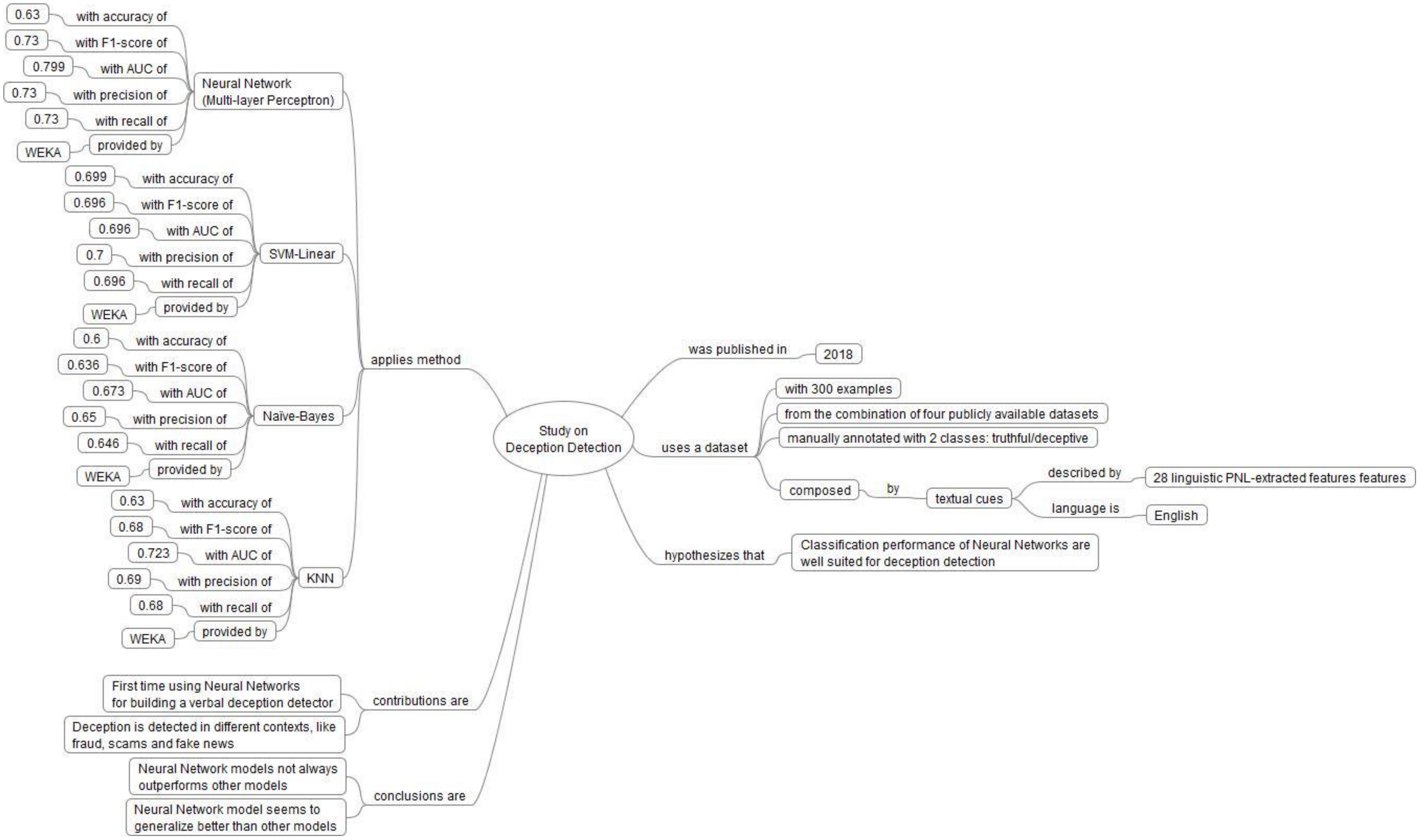

41. Intelligent Deception Detection through Machine Based Interviewing

Orshea J., Crockett K., Khan W., Kindynis P., Antoniades A., Boultadakis G. Intelligent Deception Detection through Machine Based Interviewing. Proc Int Jt Conf Neural Networks. 2018; 2018-July.

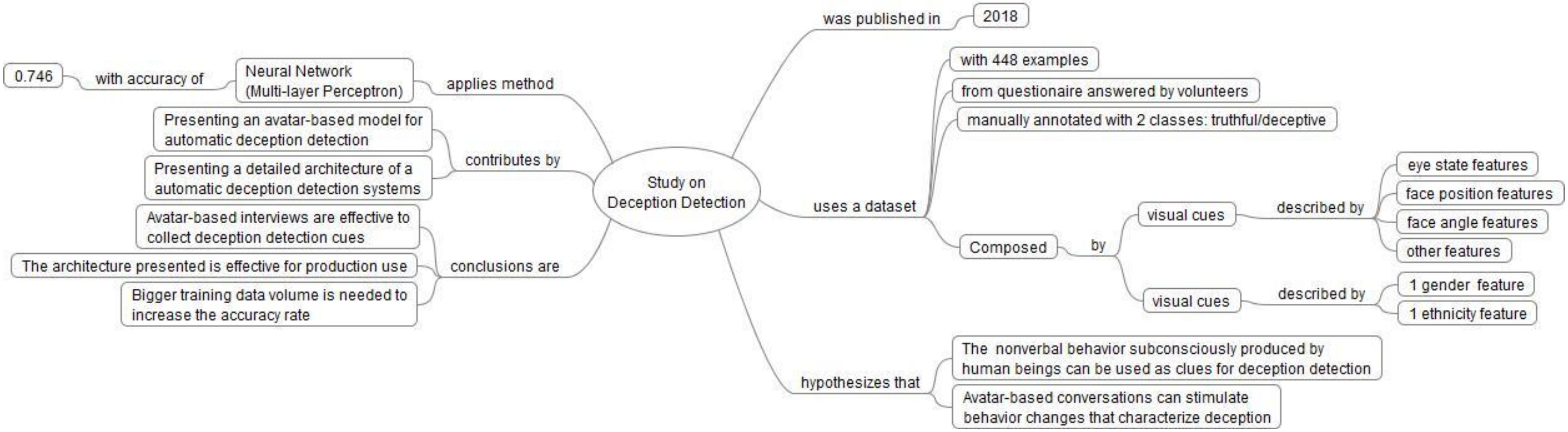

42. Deception detection using artificial neural network and support vector machine

Srivastava N., Dubey S. Deception detection using artificial neural network and support vector machine. Proc 2nd Int Conf Electron Commun Aerosp Technol ICECA 2018. 2018; (Iceca):1205–8.

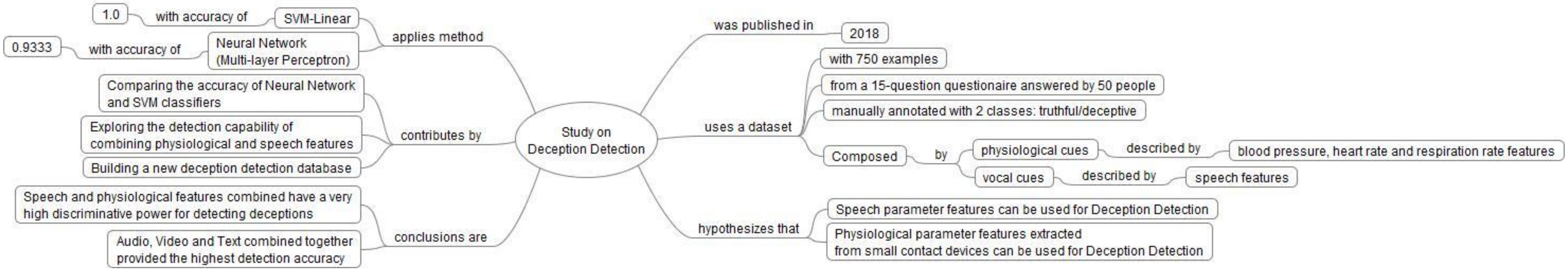

43. Construction of a liar corpus and detection of lying situations

Takabatake S., Shimada K., Saitoh T. Construction of a liar corpus and detection of lying situations. Proc - 2018 Jt 10th Int Conf Soft Comput Intell Syst 19th Int Symp Adv Intell Syst SCIS-ISIS 2018. 2018; 971–6.

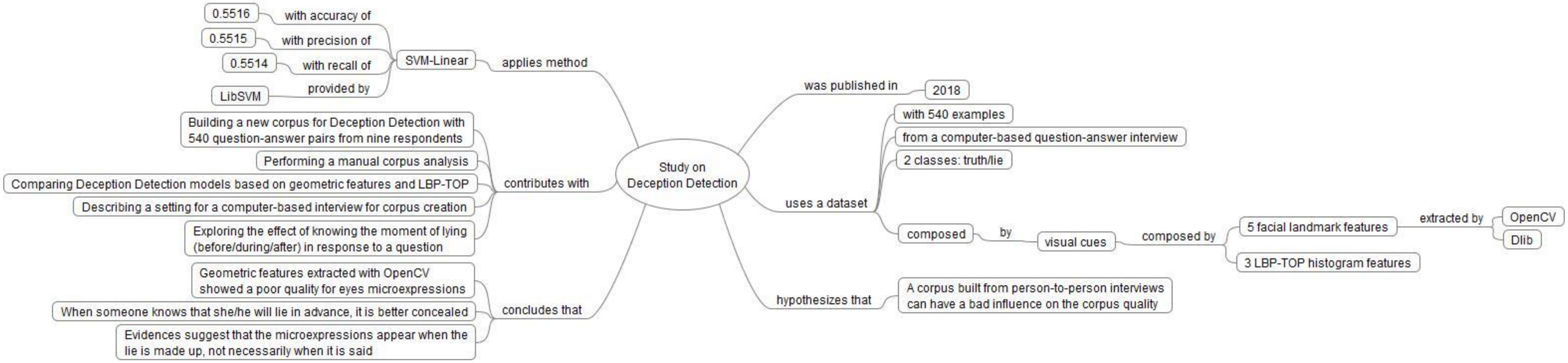

44. Detection of Deception Using Facial Expressions Based on Different Classification Algorithms

Thannoon H. H., Ali W. H., Hashim I. A. Detection of Deception Using Facial Expressions Based on Different Classification Algorithms. 2018 3rd Sci Conf Electr Eng SCEE 2018. 2018; 51–6.

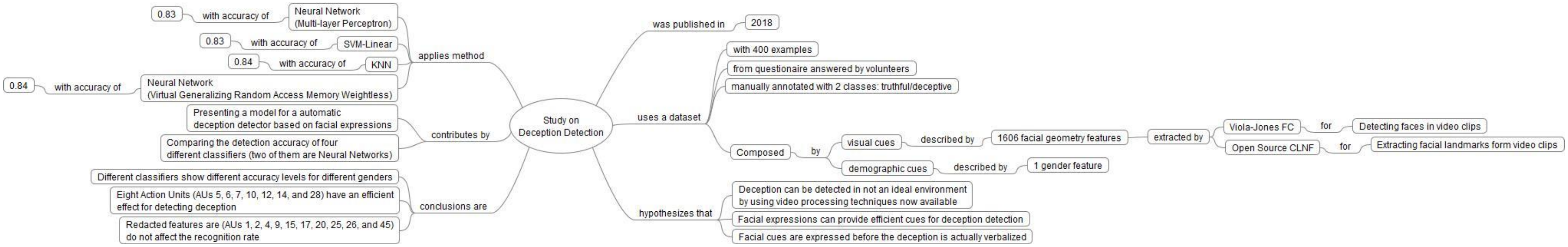

45. Comparative Analysis of Classification Methods for Automatic Deception Detection in Speech

Velichko A., Budkov V., Kagirov I., Karpov A. Comparative Analysis of Classification Methods for Automatic Deception Detection in Speech. In: Karpov A, Jokisch O, Potapova R, editors. Speech and Computer. Cham: Springer International Publishing; 2018. p. 737–46.

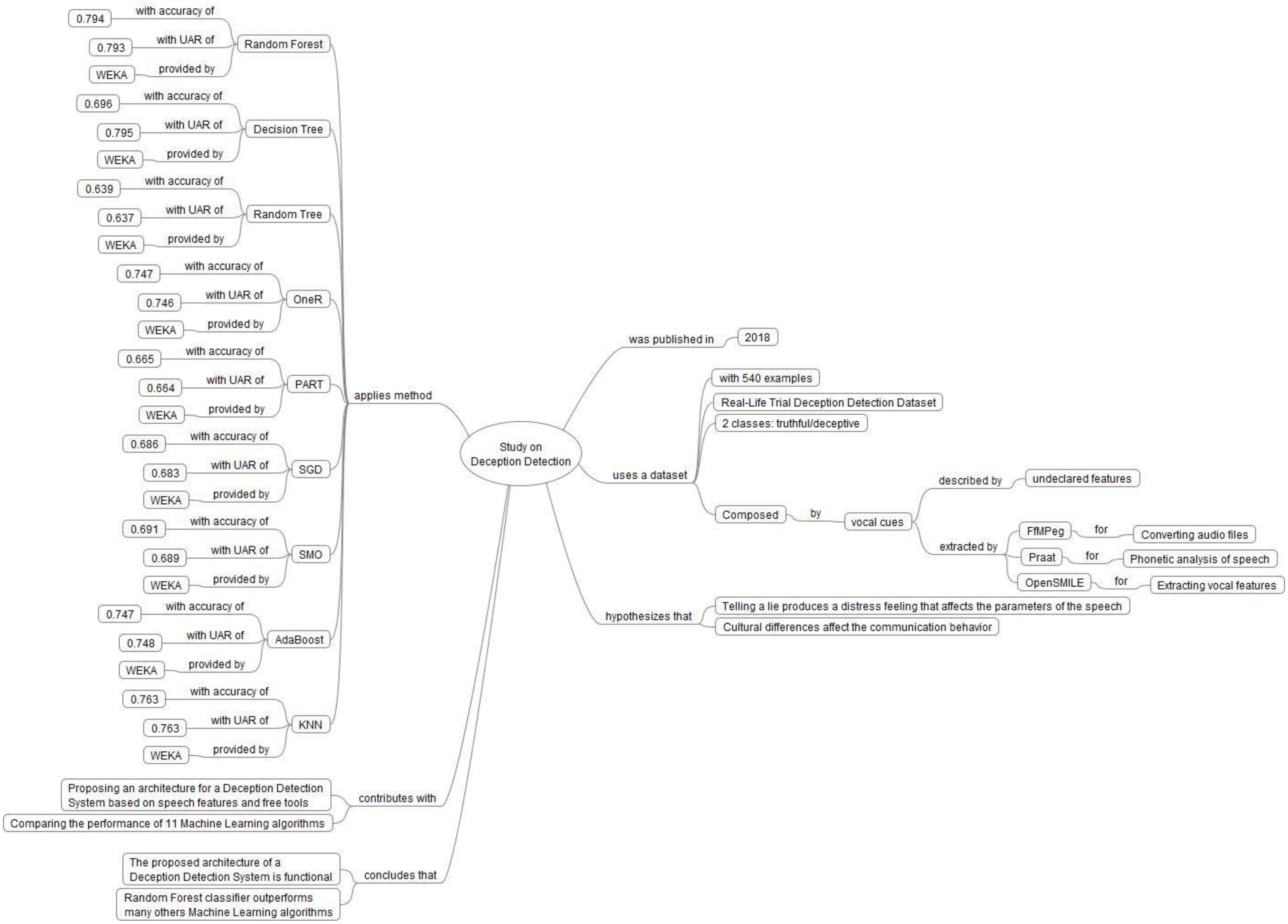
Study on Deception Detection
applies method
Random Forest
with accuracy of
0.794
with UAR of
0.793
provided by
WEKA
Decision Tree
with accuracy of
0.696
with UAR of
0.795
provided by
WEKA
Random Tree
with accuracy of
0.639
with UAR of
0.637
provided by
WEKA
OneR
with accuracy of
0.747
with UAR of
0.746
provided by
WEKA
PART
with accuracy of
0.665
with UAR of
0.664
provided by
WEKA
SGD
with accuracy of
0.686
with UAR of
0.683
provided by
WEKA
SMO
with accuracy of
0.691
with UAR of
0.689
provided by
WEKA
AdaBoost
with accuracy of
0.747
with UAR of
0.748
provided by
WEKA
KNN
with accuracy of
0.763
with UAR of
0.763
provided by
WEKA
contributes with
Proposing an architecture for a Deception Detection System based on speech features and free tools
Comparing the performance of 11 Machine Learning algorithms
concludes that
The proposed architecture of a Deception Detection System is functional
Random Forest classifier outperforms many others Machine Learning algorithms
was published in
2018
uses a dataset
with 540 examples
Real-Life Trial Deception Detection Dataset
2 classes: truthful/deceptive
Composed
by
vocal cues
described by
undeclared features
extracted by
FfMPeg
for
Converting audio files
Praat
for
Phonetic analysis of speech
OpenSMILE
for
Extracting vocal features
hypothesizes that
Telling a lie produces a distress feeling that affects the parameters of the speech
Cultural differences affect the communication behavior

46. Deception detection in videos

Wu Z., Singh B., Davis L. S., Subrahmanian V. S. Deception detection in videos. 32nd AAAI Conf Artif Intell AAAI 2018. 2018; 1695–702.

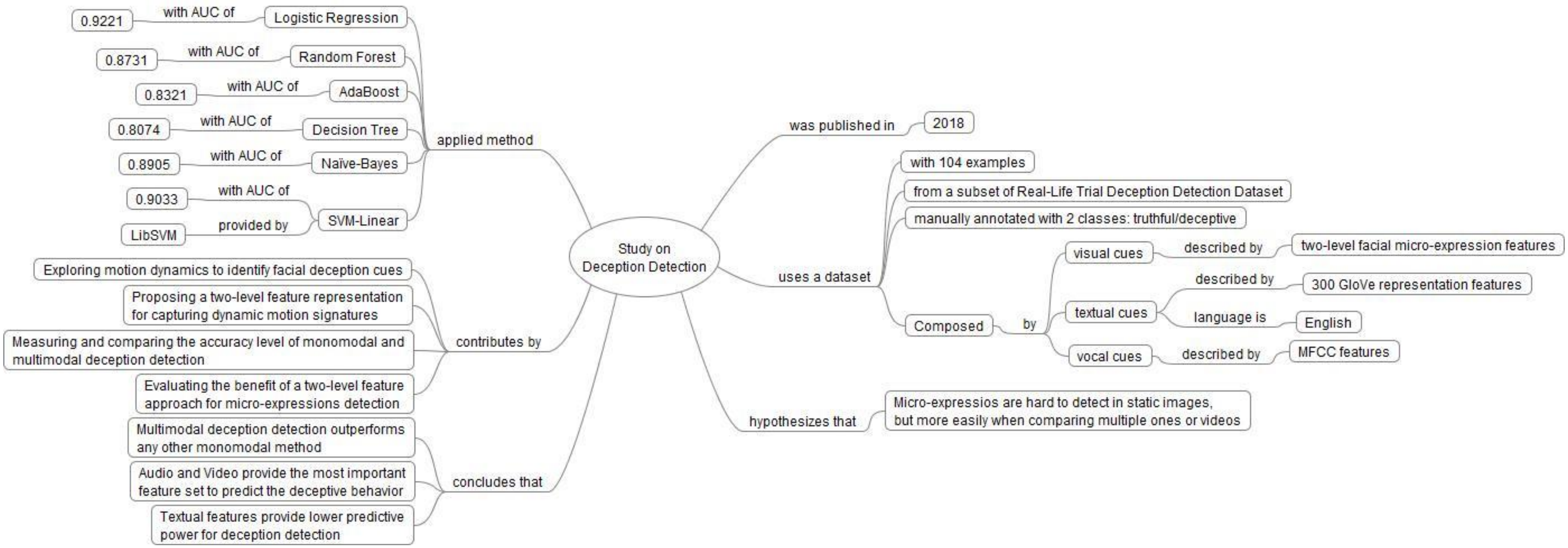

47. Convolutional bidirectional long short-term memory for deception detection with acoustic features

Xie Y., Liang R., Tao H., Zhu Y., Zhao L. Convolutional bidirectional long short-term memory for deception detection with acoustic features. IEEE Access. 2018; 6:76527–34.

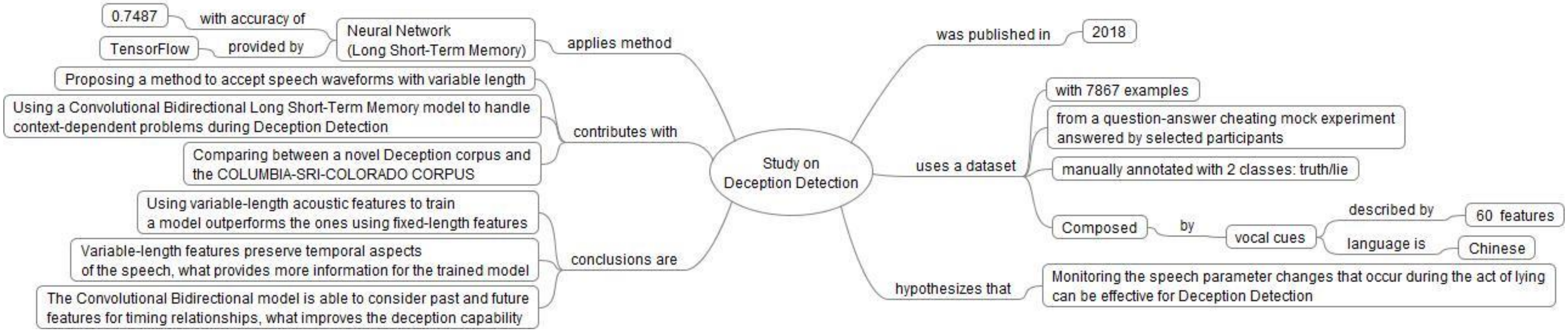

48. Automatic deception detection in RGB videos using facial action units

Avola D., Foresti G. L., Cinque L., Pannone D. Automatic deception detection in RGB videos using facial action units. ACM Int Conf Proceeding Ser. 2019;

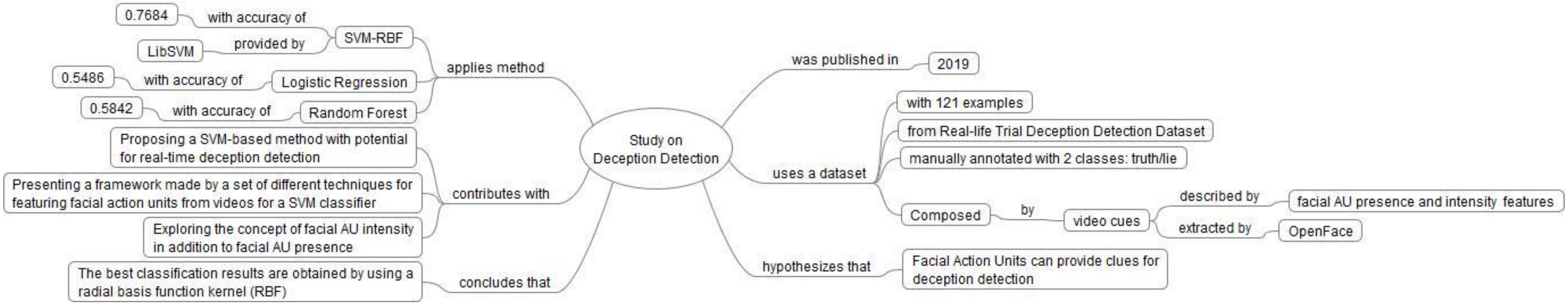

49. Automatic long-term deception detection in group interaction videos

Bai C., Bolonkin M., Burgoon J., Chen C., Dunbar N., Singh B., et al. Automatic long-term deception detection in group interaction videos. Proc - IEEE Int Conf Multimed Expo. 2019; 2019-July:1600–5.

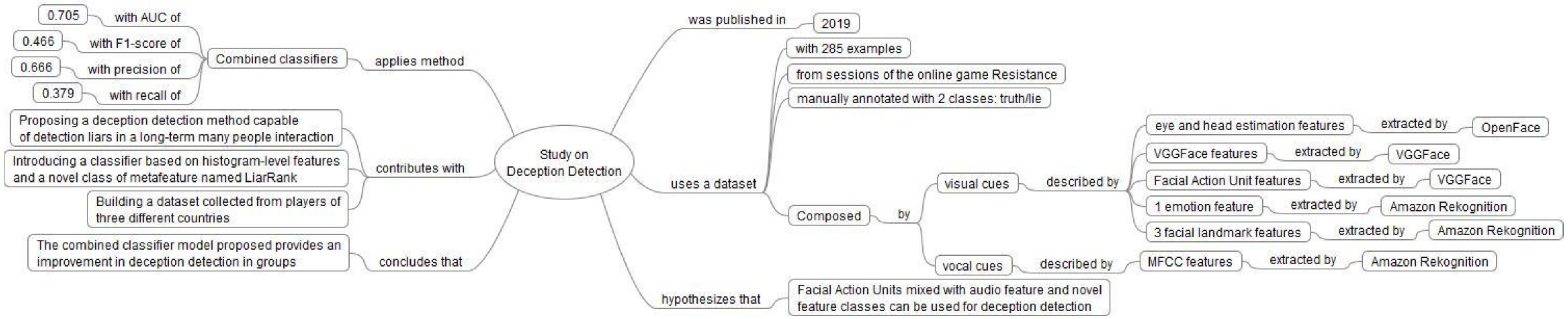

50. Joint learning of conversational temporal dynamics and acoustic features for speech deception detection in dialog games

Chou H. C., Liu Y. W., Lee C. C. Joint learning of conversational temporal dynamics and acoustic features for speech deception detection in dialog games. 2019 Asia-Pacific Signal Inf Process Assoc Annu Summit Conf APSIPA ASC 2019. 2019;(November):1044–50.

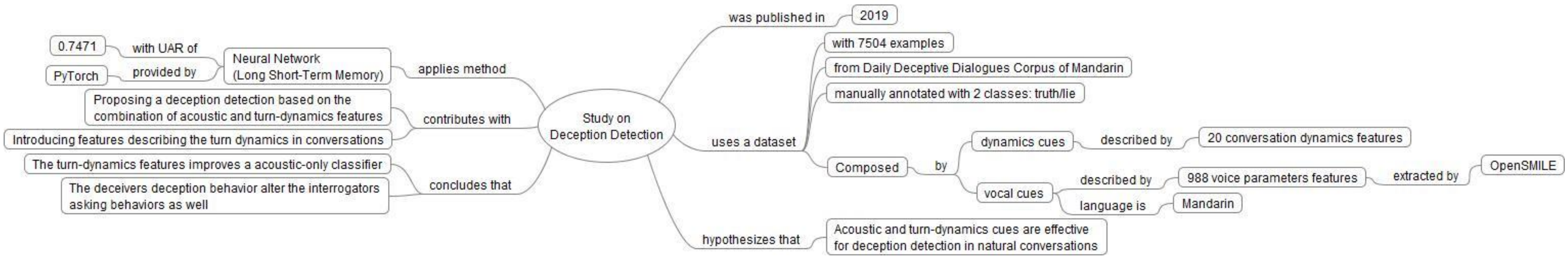

51. Face-focused cross-stream network for deception detection in videos

Ding M., Zhao A., Lu Z., Xiang T., Wen J. R. Face-focused cross-stream network for deception detection in videos. Proc IEEE Comput Soc Conf Comput Vis Pattern Recognit. 2019; 2019-June(2):7794–803.

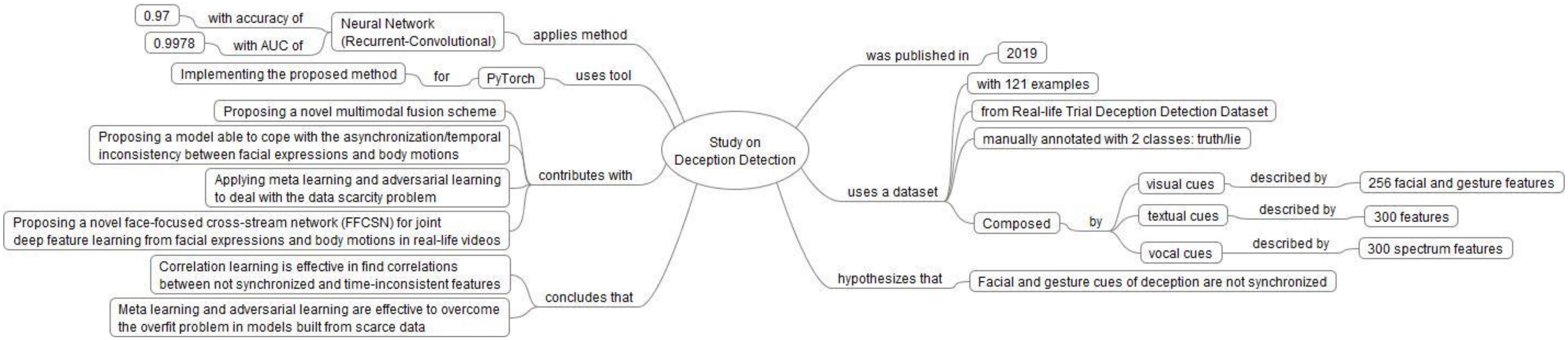

52. Improved semi-supervised autoencoder for deception detection

Fu H., Lei P., Tao H., Zhao L., Yang J. Improved semi-supervised autoencoder for deception detection. PLoS One [Internet]. 2019; 14(10):1-13. Available from: http://dx.doi.org/10.1371/journal.pone.0223361

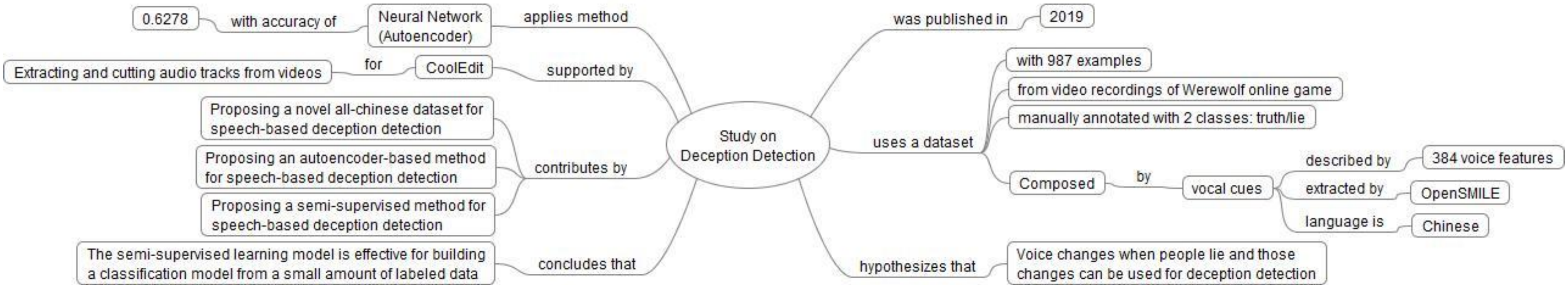

53. Can a Robot Catch You Lying? A Machine Learning System to Detect Lies During Interactions

Gonzalez-Billandon J., Aroyo A. M., Tonelli A., Pasquali D., Sciutti A., Gori M., et al. Can a Robot Catch You Lying? A Machine Learning System to Detect Lies During Interactions. Front Robot AI. 2019; 6(July):1-12.

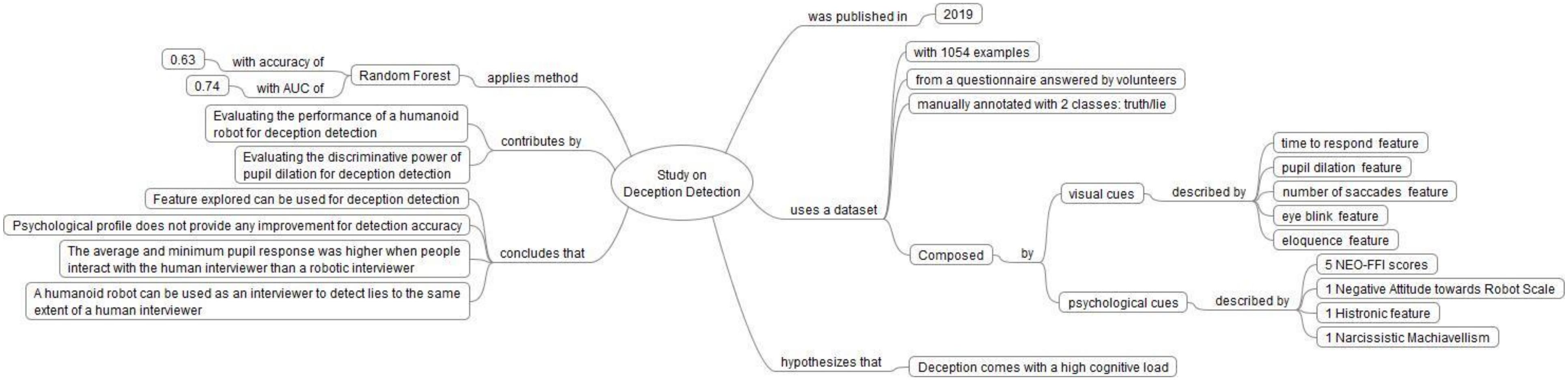

54. Bag-of-lies: A multimodal dataset for deception detection

Gupta V., Agarwal M., Arora M., Chakraborty T., Singh R., Vatsa M. Bag-of-lies: A multimodal dataset for deception detection. IEEE Comput Soc Conf Comput Vis Pattern Recognit Work. 2019; 2019-June:83–90.

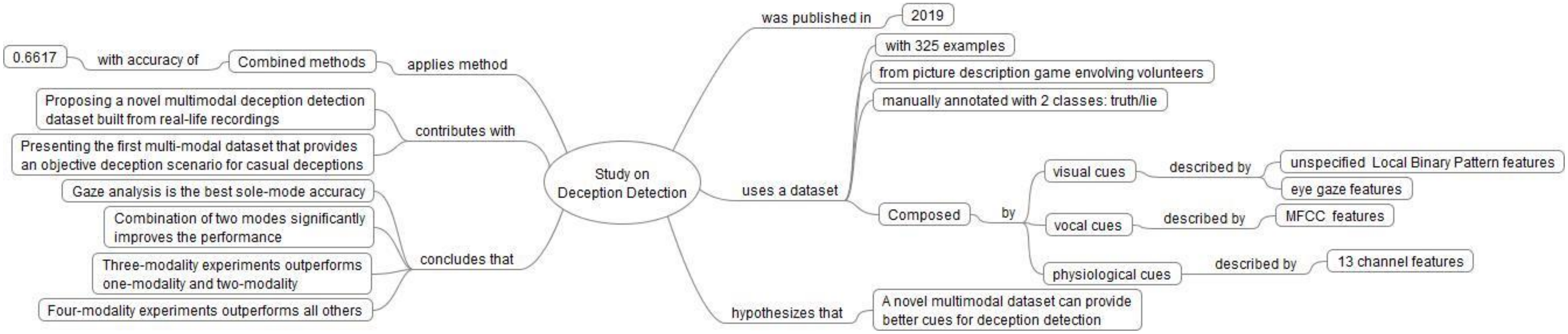

55. Detecting concealed information in text and speech

Hu S. Detecting concealed information in text and speech. ACL 2019 - 57th Annu Meet Assoc Comput Linguist Proc Conf. 2020; 402–12.

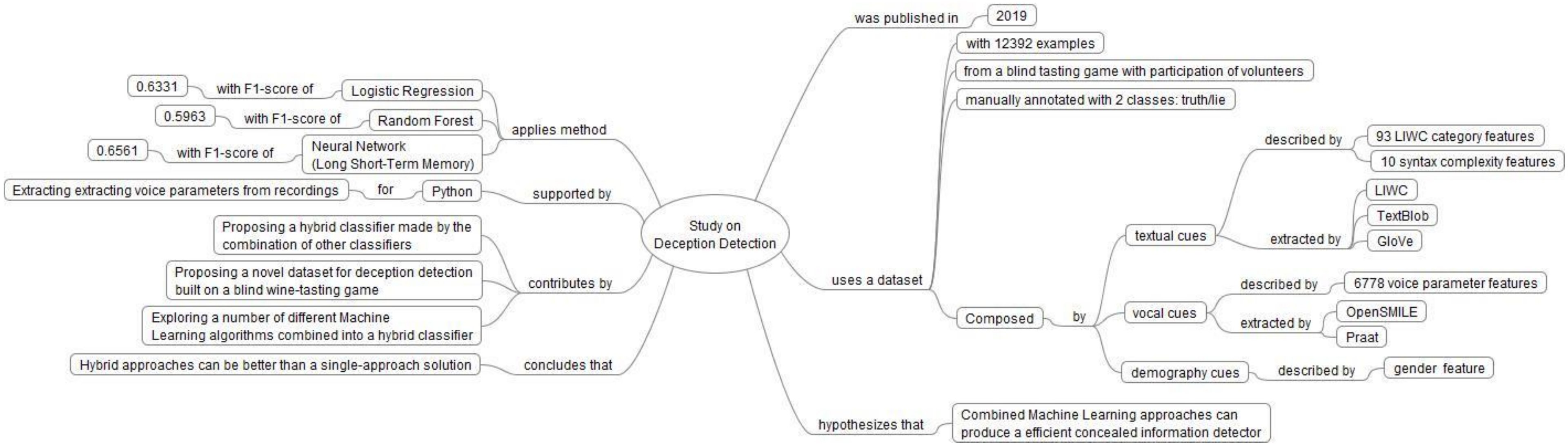

56. Detecting Deception in Political Debates Using Acoustic and Textual Features

Kopev D., Ali A., Koychev I., Nakov P. Detecting Deception in Political Debates Using Acoustic and Textual Features. 2019 IEEE Autom Speech Recognit Underst Work ASRU 2019 - Proc. 2019; 652-9.

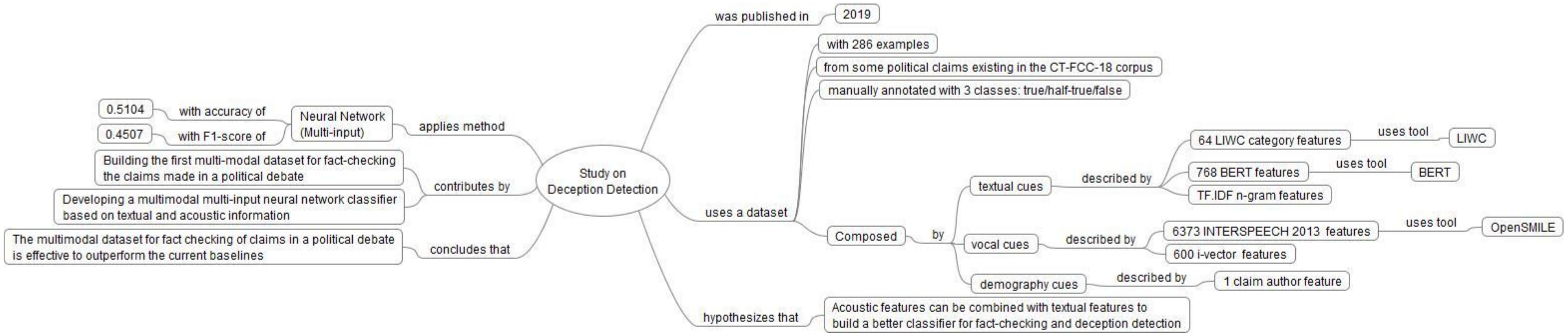

57. How smart your smartphone is in lie detection?

Mizanur Rahman M., Shome A., Chellappan S., Alim Al Islam A. B. M. How smart your smartphone is in lie detection? ACM Int Conf Proceeding Ser. 2019; 338-47.

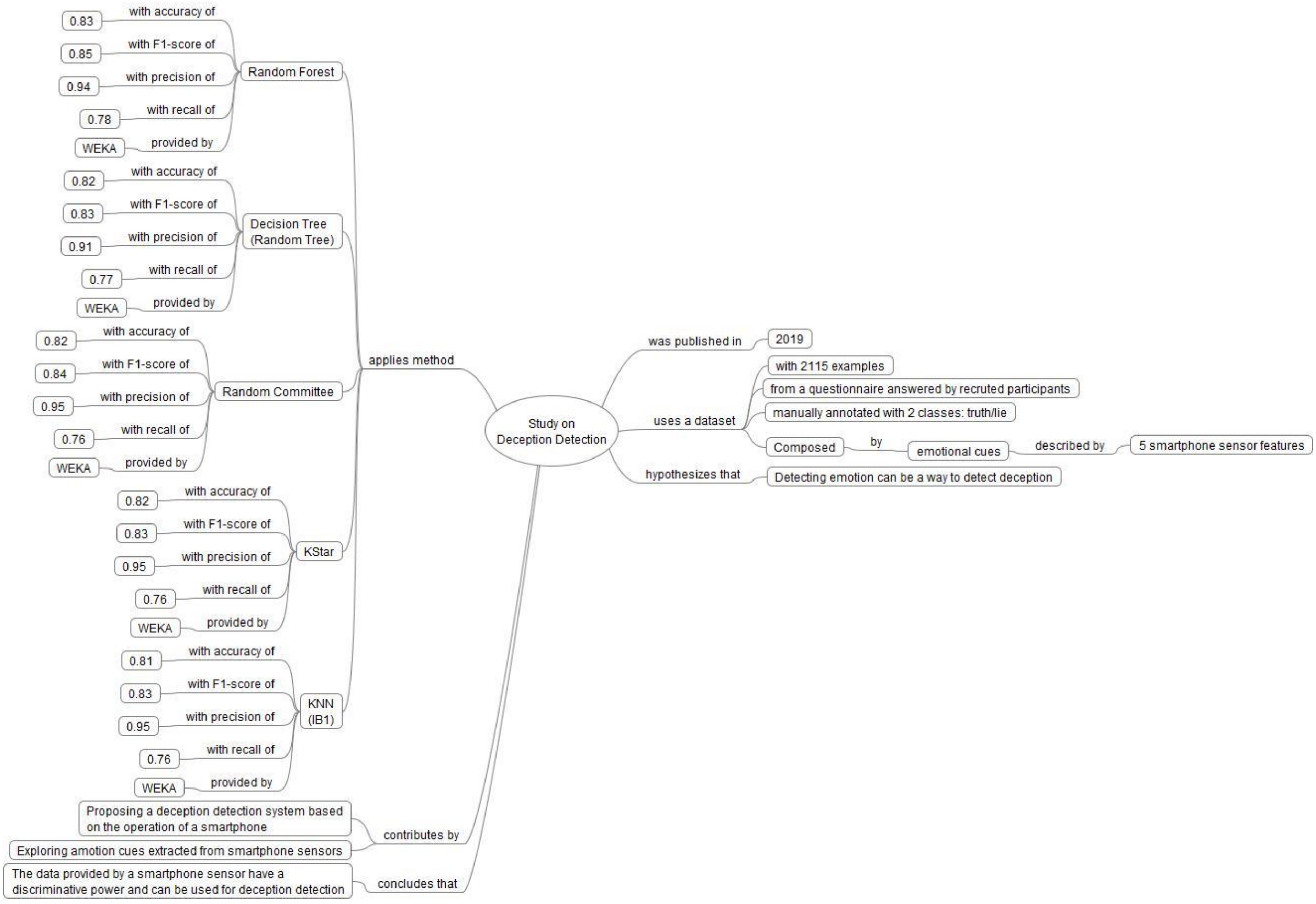

58. High-level features for multimodal deception detection in videos

Rill-Garcia R., Escalante H. J., Villasenor-Pineda L., Reyes-Meza V. High-level features for multimodal deception detection in videos. IEEE Comput Soc Conf Comput Vis Pattern Recognit Work. 2019; 2019-June:1565–73.

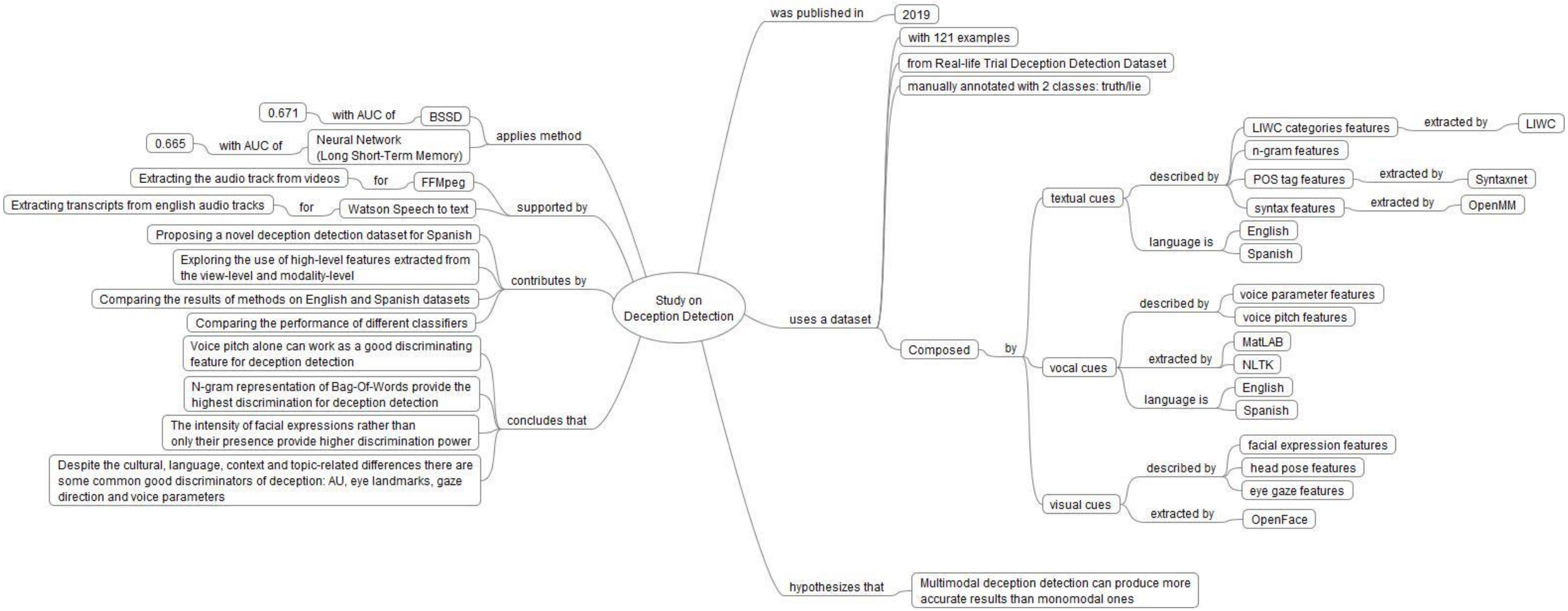

59. Speech Deception Detection Algorithm Based on SVM and Acoustic Features

Tao H., Lei P., Wang M., Wang J., Fu H. Speech Deception Detection Algorithm Based on SVM and Acoustic Features. Proc IEEE 7th Int Conf Comput Sci Netw Technol ICCSNT 2019. 2019;31–3.

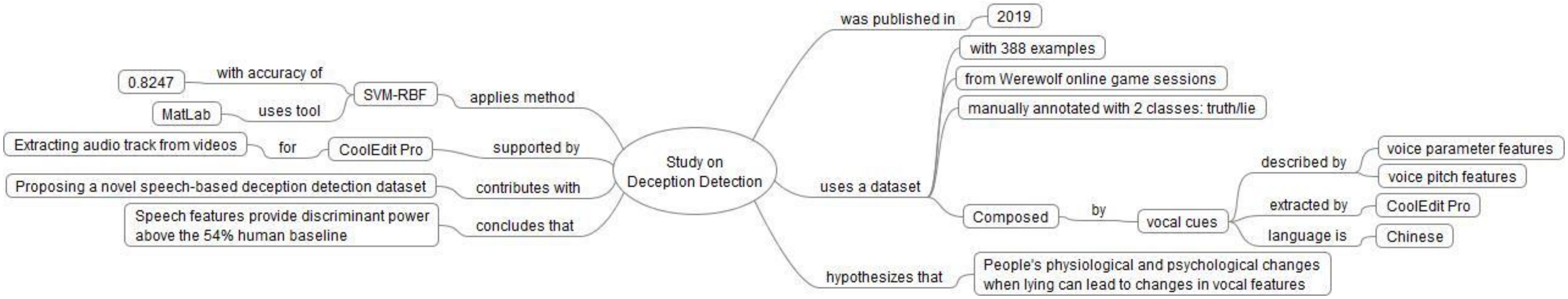

60. Robust Algorithm for Multimodal Deception Detection

Venkatesh S., Ramachandra R., Bours P. Robust Algorithm for Multimodal Deception Detection. Proc - 2nd Int Conf Multimed Inf Process Retrieval, MIPR 2019. 2019; 534–7.

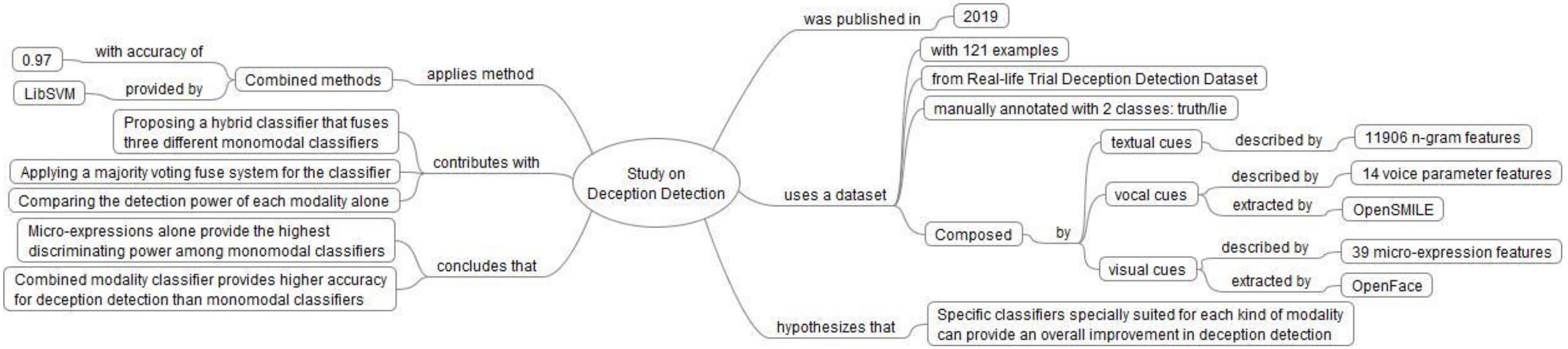

61. Building a Better Lie Detector with BERT: The Difference between Truth and Lies

Barsever D., Singh S., Neftci E. Building a Better Lie Detector with BERT: The Difference between Truth and Lies. Proc Int Jt Conf Neural Networks. 2020;

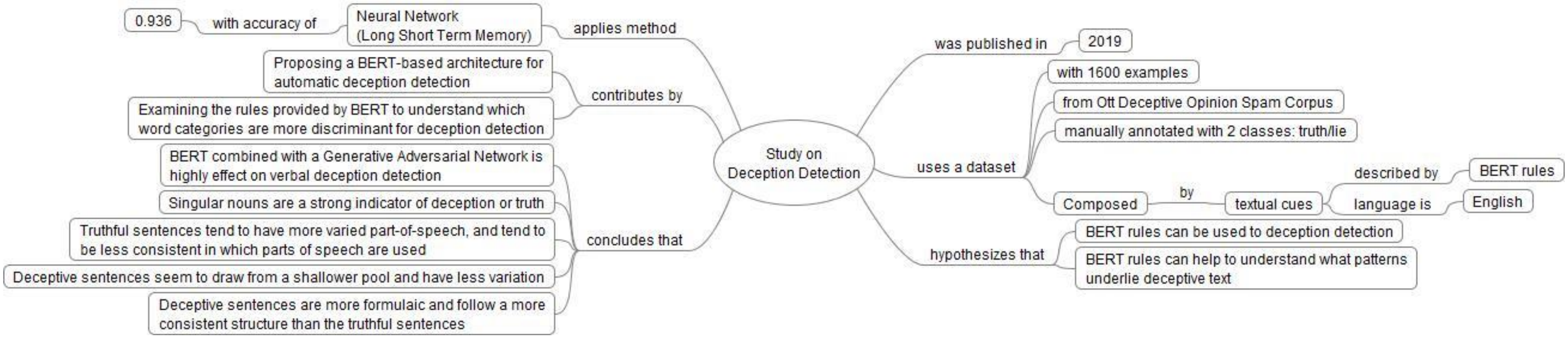

62. Automated Deception Detection of Males and Females from Non-Verbal Facial Micro-Gestures

Crockett K., O’Shea J., Khan W. Automated Deception Detection of Males and Females from Non-Verbal Facial Micro-Gestures. Proc Int Jt Conf Neural Networks. 2020;

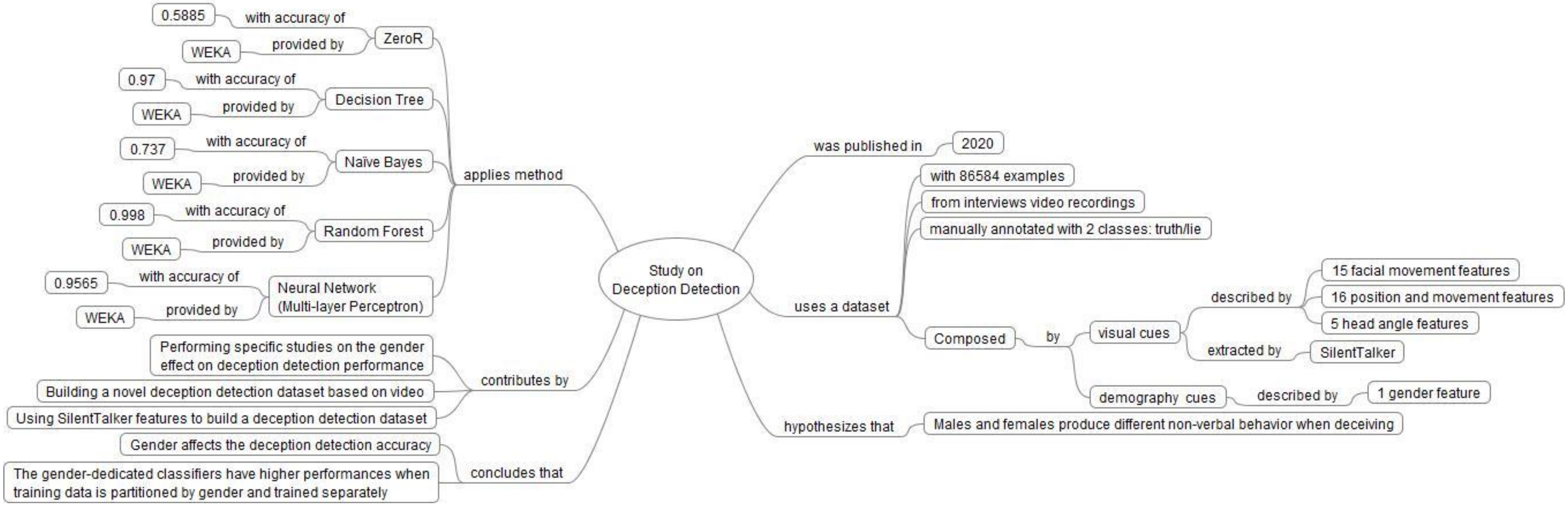

63. Introducing Representations of Facial Affect in Automated Multimodal Deception Detection

Mathur L., Matarić M. J. Introducing Representations of Facial Affect in Automated Multimodal Deception Detection. ICMI 2020 - Proc 2020 Int Conf Multimodal Interact. 2020; 305–14.

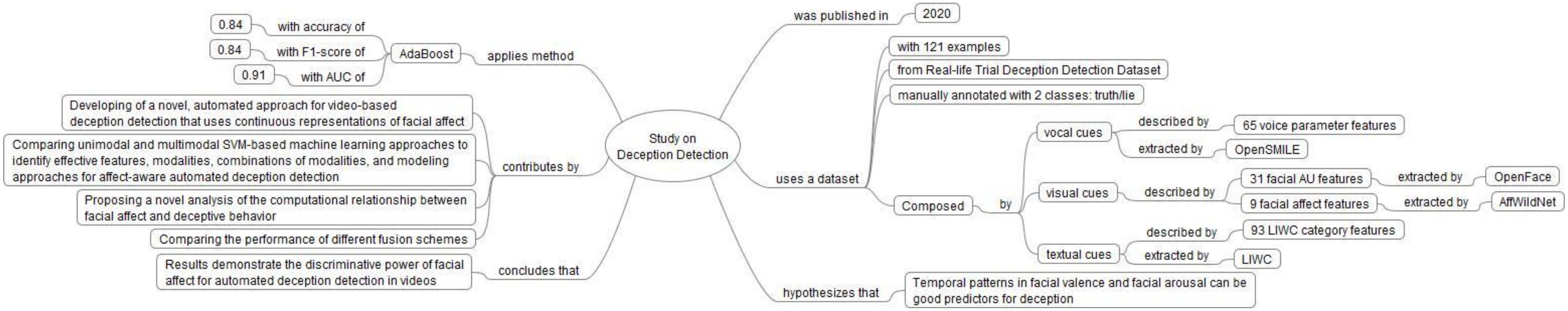

64. Multilingual Deception Detection by Autonomous Agents

Hershkovitch Neiterman E., Bitan M., Azaria A. Multilingual Deception Detection by Autonomous Agents. Web Conf 2020 - Companion World Wide Web Conf WWW 2020. 2020; 480–4.

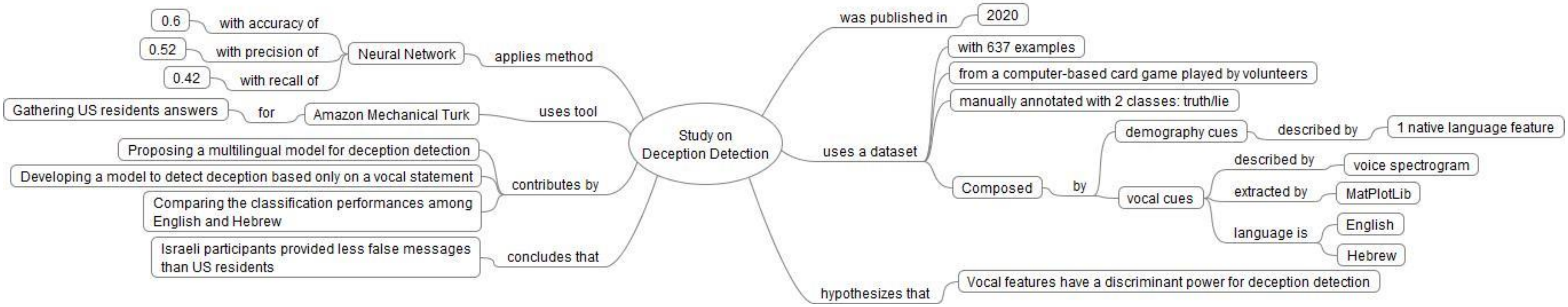

65. Your eyes never lie: A robot magician can tell if you are lying

Pasquali D., Aroyo A. M., Gonzalez-Billandon J., Rea F., Sandini G., Sciutti A. Your eyes never lie: A robot magician can tell if you are lying. ACM/IEEE Int Conf Human-Robot Interact. 2020;392–4.

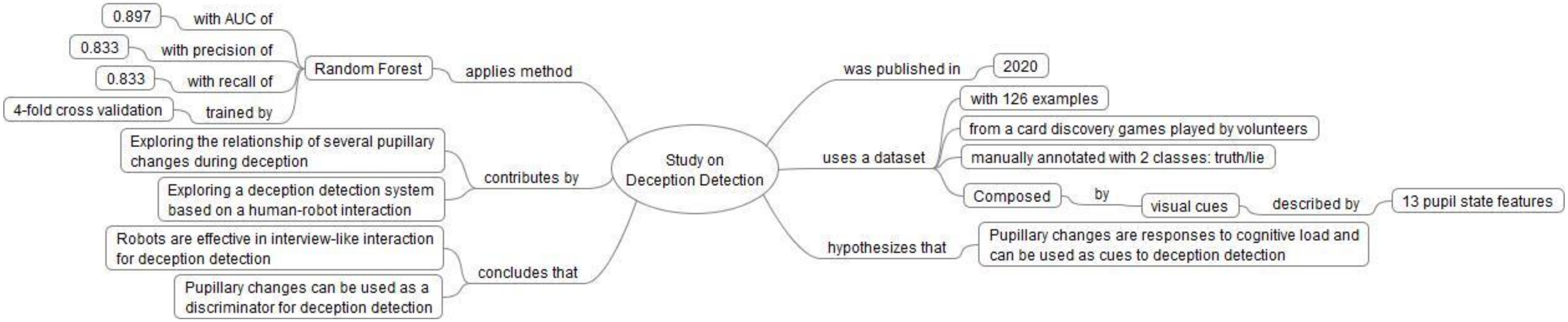

66. Multimodal Deception Detection using Real-Life Trial Data

Sen U. M., Pérez-Rosas V., Yanikoglu B., Abouelenien M., Burzo M., Mihalcea R. Multimodal Deception Detection using Real-Life Trial Data. IEEE Trans Affect Comput. 2020; 3045(c):1–14.

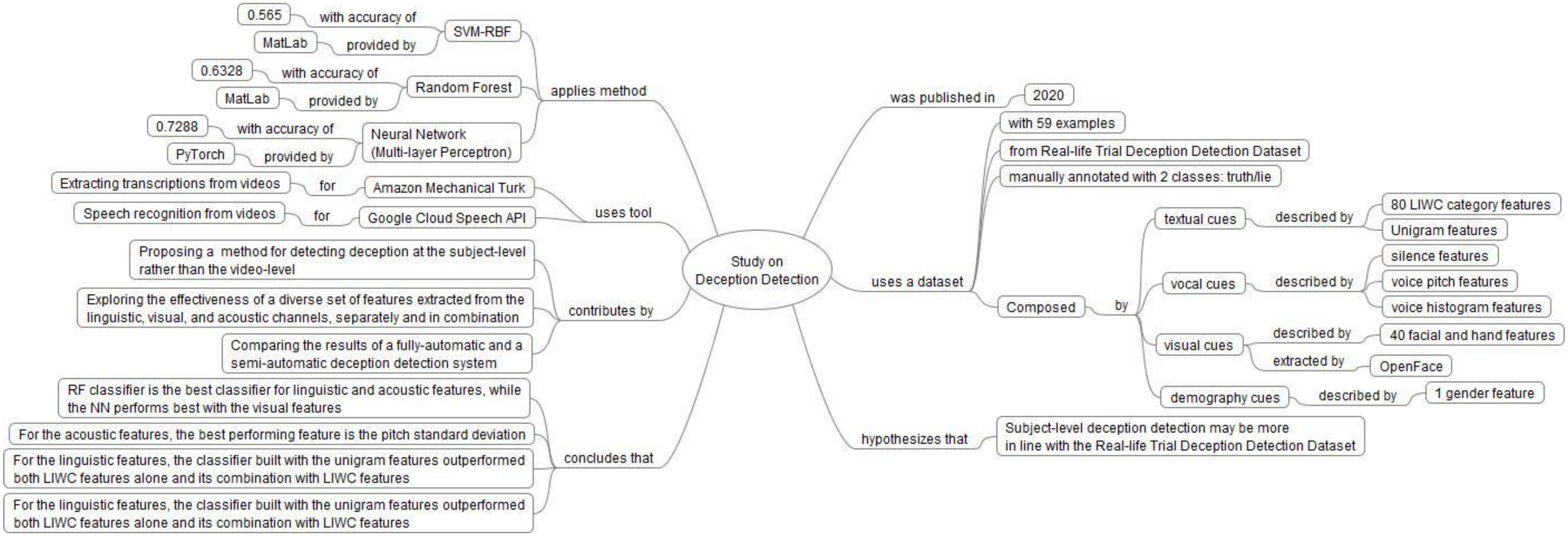

67. Emotion Transformation Feature: Novel Feature for Deception Detection in Videos

Yang J. T., Liu G. M., Huang S. C. H. Emotion Transformation Feature: Novel Feature for Deception Detection in Videos.

In: Proceedings - International Conference on Image Processing, ICIP. 2020. p. 1726-30.

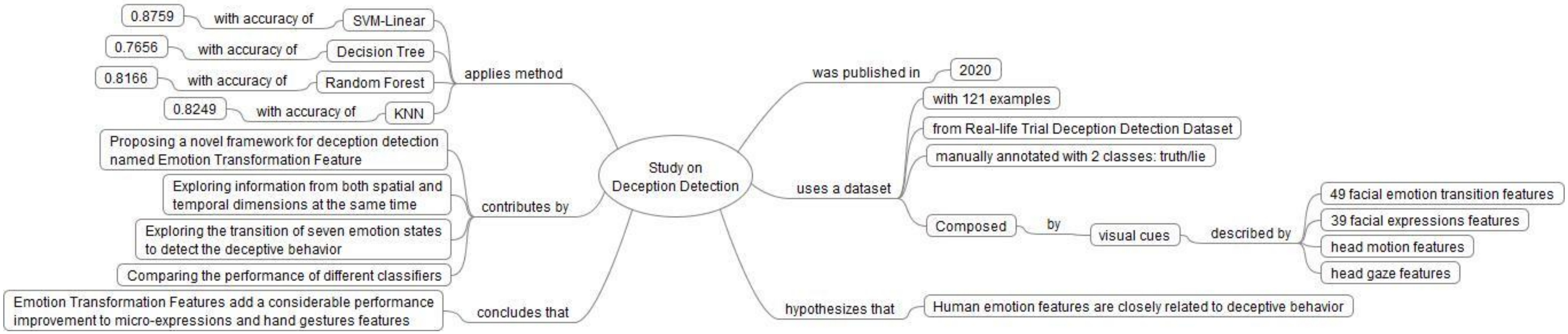

68. Development of Spectral Speech Features for Deception Detection Using Neural Networks

FERNANDES, Sinead V.; ULLAH, Muhammad S. Development of Spectral Speech Features for Deception Detection Using Neural Networks. 2021 IEEE 12th Annual Information Technology, Electronics and Mobile Communication Conference, IEMCON 2021, [S. l.], p. 198-203, 2021. b. DOI: 10.1109/IEMCON53756.2021.9623077.

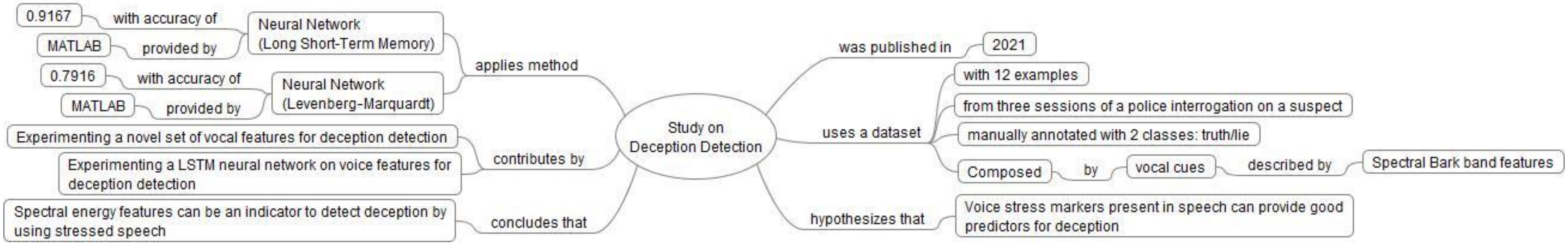

69. Use of Machine Learning for Deception Detection from Spectral and Cepstral Features of Speech Signals

FERNANDES, Sinead V.; ULLAH, Muhammad S. Use of Machine Learning for Deception Detection from Spectral and Cepstral Features of Speech Signals. IEEE Access, [S. l.], v. 9, p. 78925-78935, 2021. a. DOI: 10.1109/ACCESS.2021.3084200.

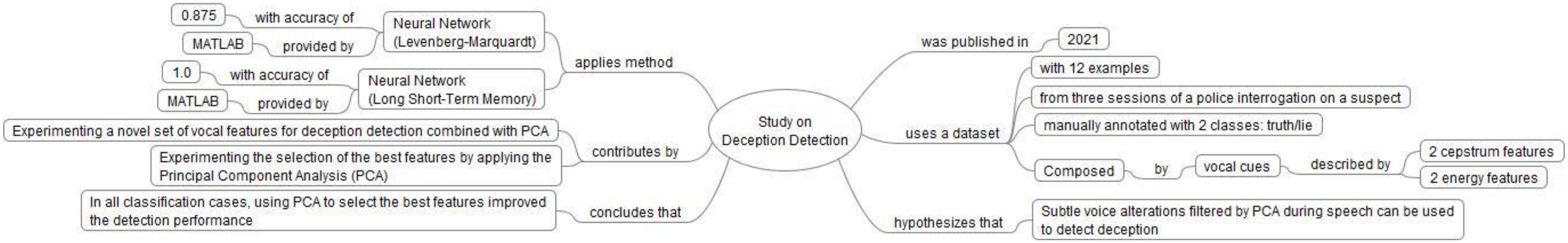

70. Non-invasive Deception Detection in Videos Using Machine Learning Techniques

ISLAM, Siam; SAHA, Popin; CHOWDHURY, Touhidul; SOROWAR, Asif; RAB, Raqeebir. Non-invasive Deception Detection in Videos Using Machine Learning Techniques. 2021 5th International Conference on Electrical Engineering and Information and Communication Technology, ICEEICT 2021, [S. l.], 2021. DOI: 10.1109/ICEEICT53905.2021.9667928.

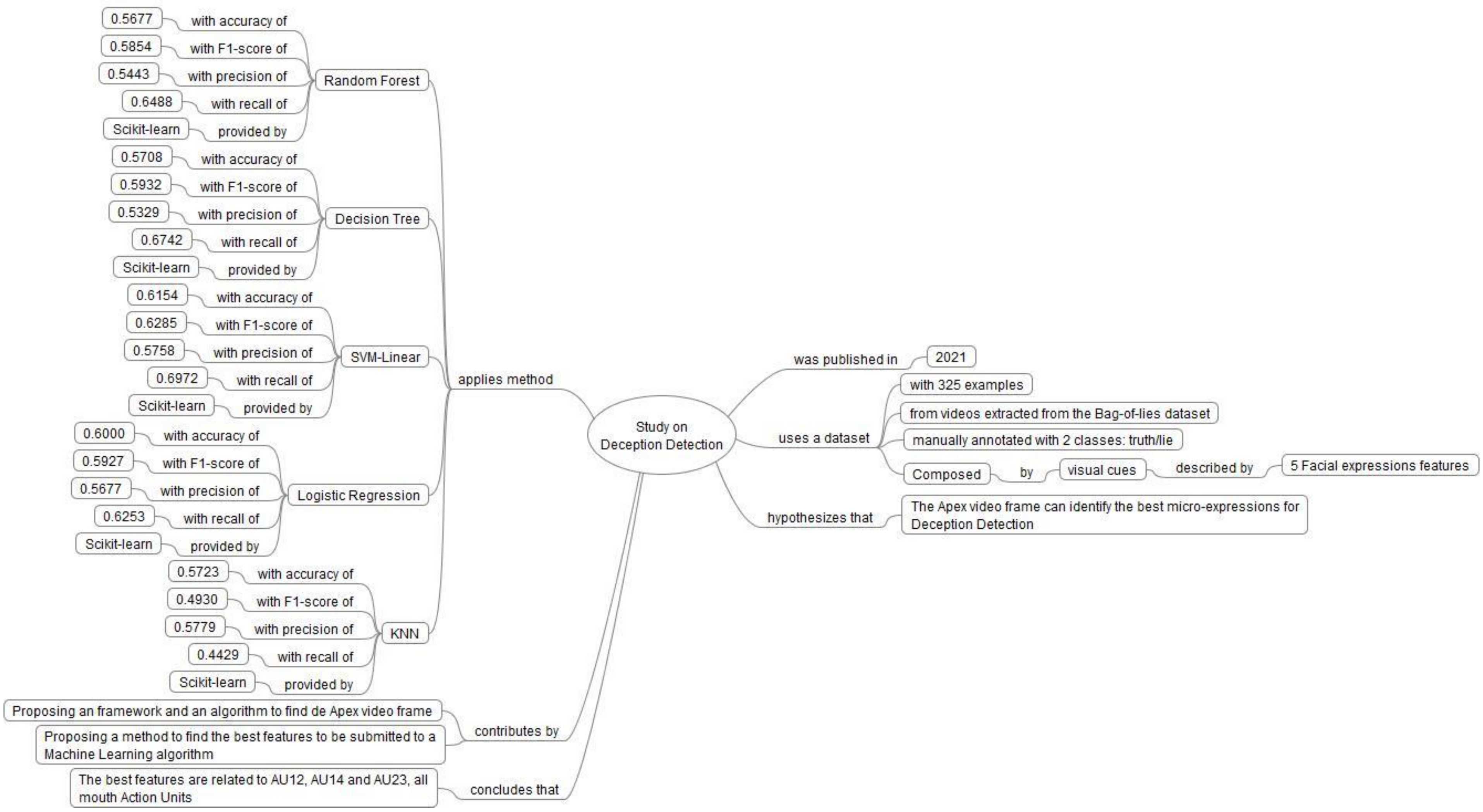

71. Multimodal Political Deception Detection

KAMBOJ, Manvi; HESSLER, Christian; ASNANI, Priyanka; RIANI, Kais; ABOUELENIEN, Mohamed. Multimodal Political Deception Detection. IEEE Multimedia, [S. l.], v. 28, n. 1, p. 94-102, 2021. DOI: 10.1109/MMUL.2020.3048044.

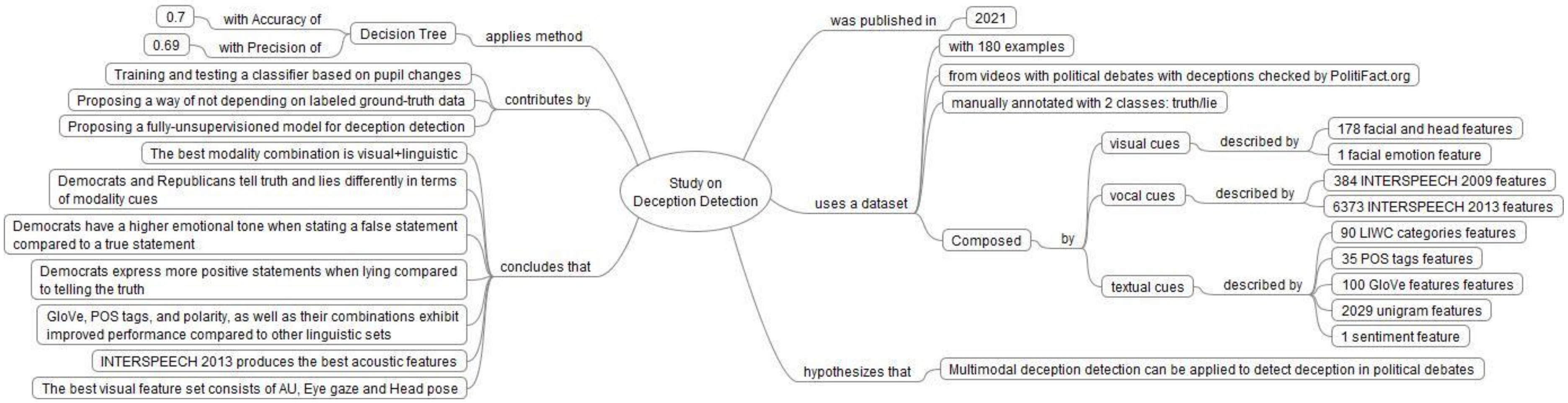

72. LieNet: A Deep Convolution Neural Networks Framework for Detecting Deception

KARNATI, Mohan; SEAL, Ayan; YAZIDI, Anis; KREJCAR, Ondrej. LieNet: A Deep Convolution Neural Networks Framework for Detecting Deception. IEEE Transactions on Cognitive and Developmental Systems, [S. l.], v. 8920, n. c, p. 1–15, 2021. DOI: 10.1109/TCDS.2021.3086011.

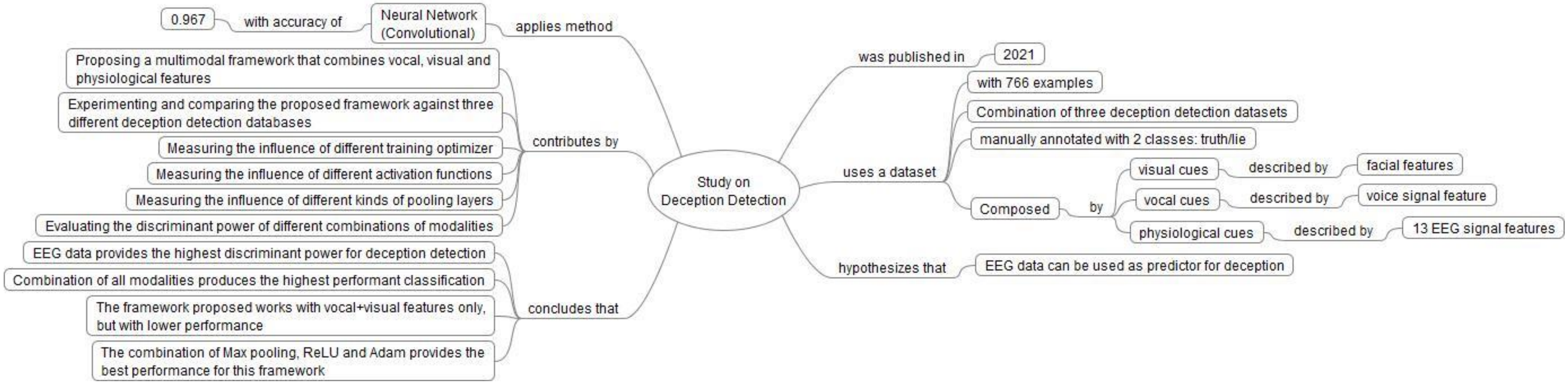

73. Deception in the eyes of deceiver: A computer vision and machine learning based automated deception detection

KHAN, Wasiq; CROCKETT, Keeley; O'SHEA, James; HUSSAIN, Abir; KHAN, Bilal M. Deception in the eyes of deceiver: A computer vision and machine learning based automated deception detection. Expert Systems with Applications, [S. l.], v.

169, n. February 2020, p. 114341, 2021. DOI: 10.1016/j.eswa.2020.114341. Disponível em: https://doi.org/10.1016/j.eswa.2020.114341.

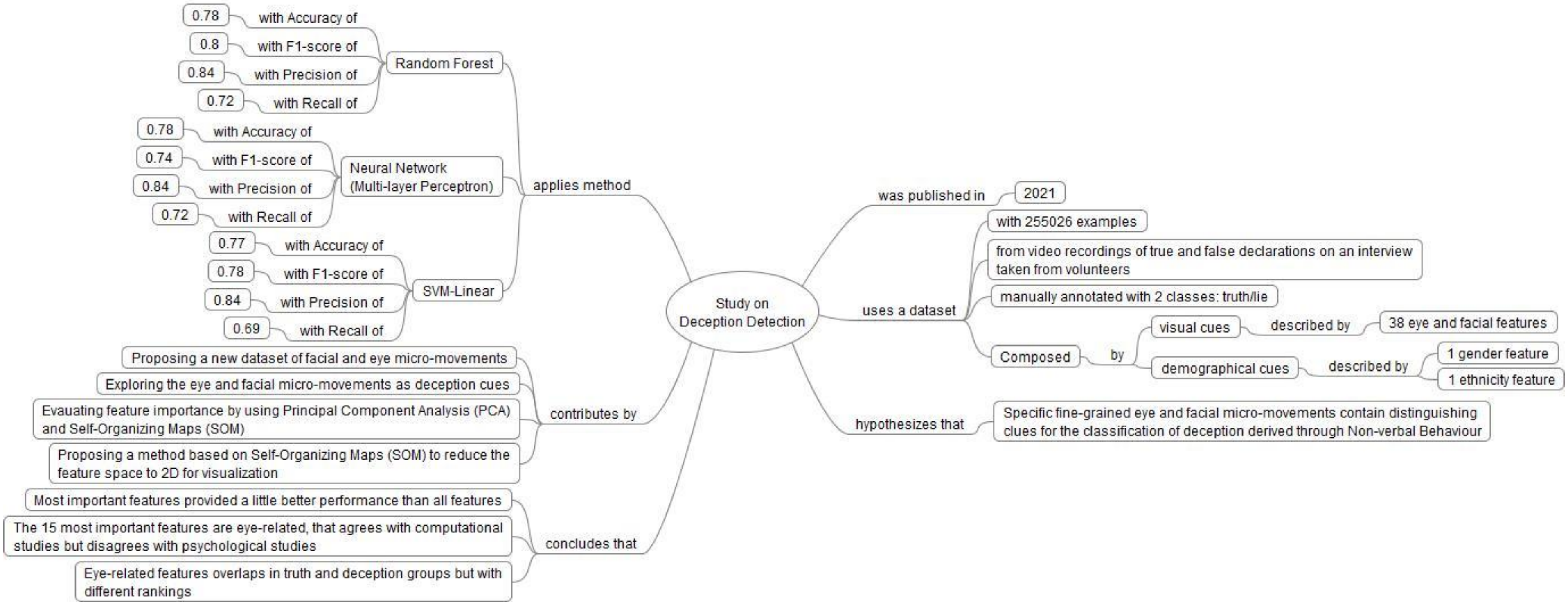

74. How humans impair automated deception detection performance

KLEINBERG, Bennett; VERSCHUERE, Bruno. How humans impair automated deception detection performance. Acta Psychologica, [S. l.], v. 213, n. March 2020, p. 103250, 2021. DOI: 10.1016/j.actpsy.2020.103250. Available in: https://doi.org/10.1016/j.actpsy.2020.103250.

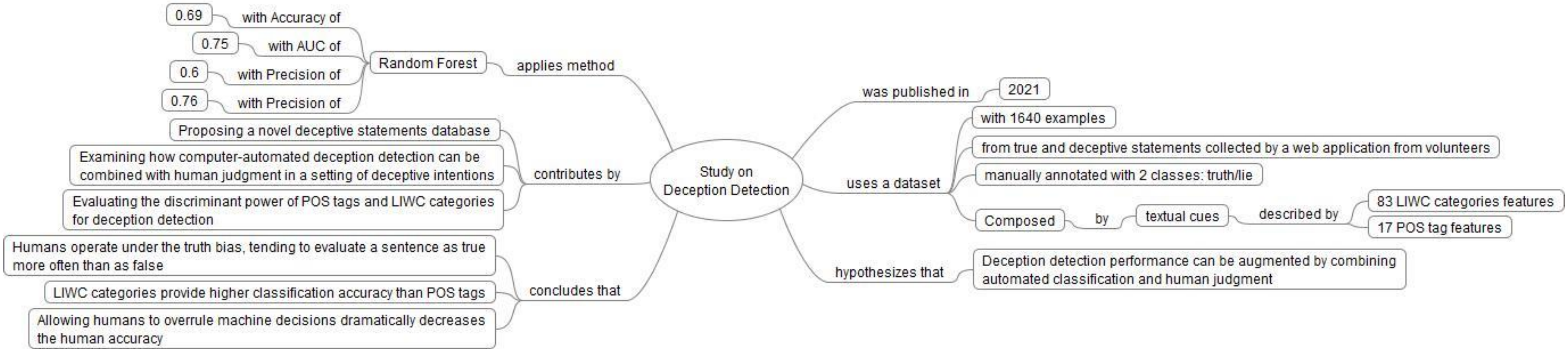

75. Affect-Aware Deep Belief Network Representations for Multimodal Unsupervised Deception Detection

MATHUR, Leena; MATARIC, Maja J. Affect-Aware Deep Belief Network Representations for Multimodal Unsupervised Deception Detection. Proceedings - 2021 16th IEEE International Conference on Automatic Face and Gesture Recognition, FG 2021, [S. l.], 2021. DOI: 10.1109/FG52635.2021.9667050.

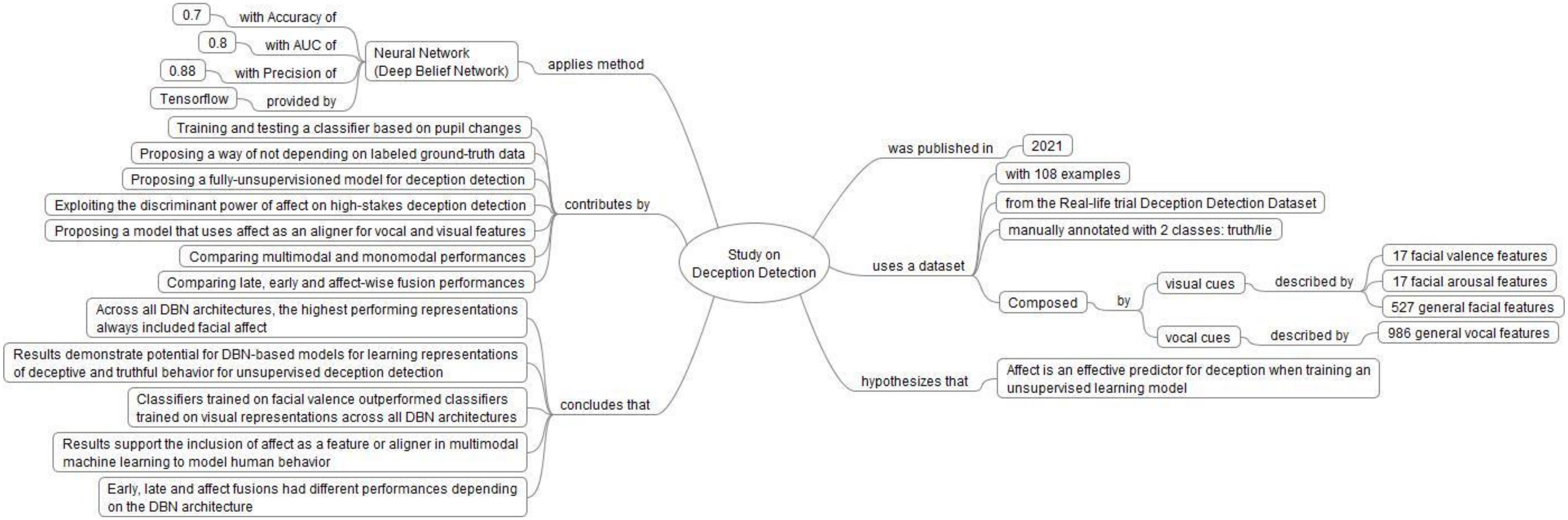

76. Unsupervised Audio-Visual Subspace Alignment for High-Stakes Deception Detection

MATHUR, Leena; MATARIĆ, Maja J. Unsupervised Audio-Visual Subspace Alignment for High-Stakes Deception Detection. Proceedings - 2021 IEEE International Conference on Acoustics, Speech and Signal Processing, ICASSP 2021, [S. l.], p. 2255–2259, 2021. DOI: 10.1109/ICASSP39728.2021.9413550.

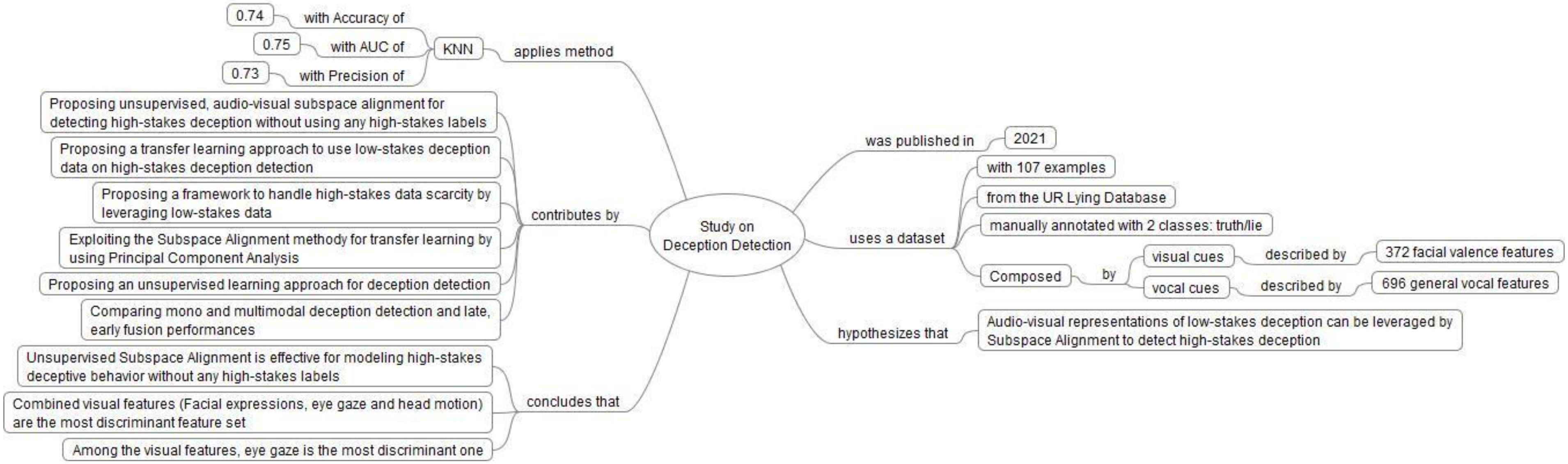

77. Identity unbiased deception detection by 2d-to-3d face reconstruction

NGO, Le Minh; WANG, Wei; MANDIRA, Burak; KARAOGLU, Sezer; BOUMA, Henri; DIBEKLIOGLU, Hamdi; GEVERS, Theo. Identity unbiased deception detection by 2d-to-3d face reconstruction. Proceedings - 2021 IEEE Winter Conference on Applications of Computer Vision, WACV 2021, [S. l.], p. 145–154, 2021. DOI: 10.1109/WACV48630.2021.00019.

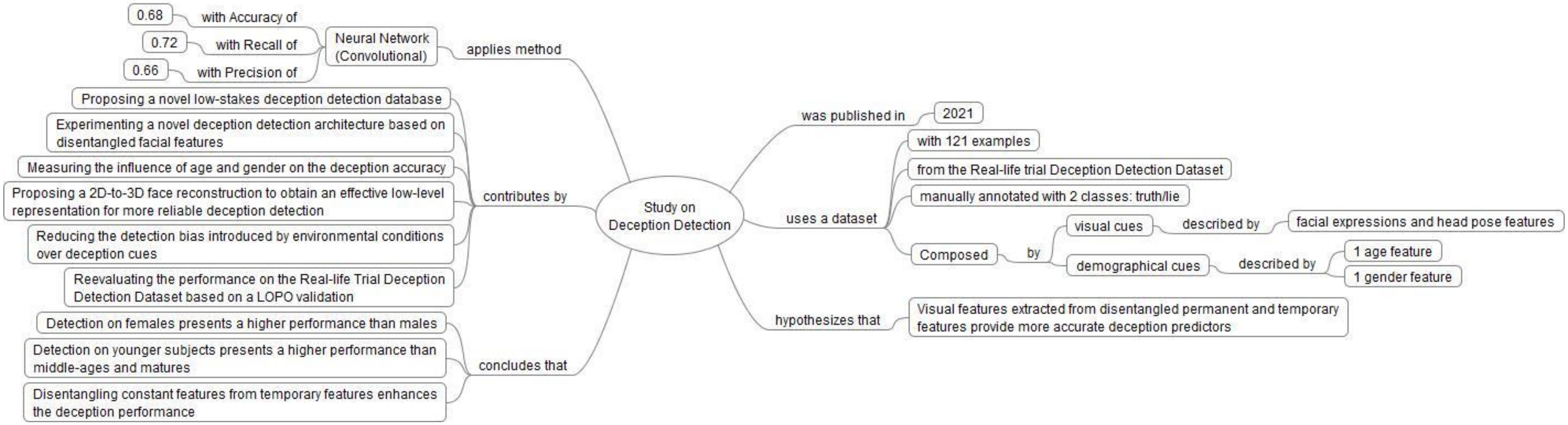

78. Deception detection in text and its relation to the cultural dimension of individualism/collectivism

PAPANTONIOU, Katerina; PAPADAKOS, Panagiotis; PATKOS, Theodore; FLOURIS, George; ANDROUTSOPOULOS, Ion; PLEXOUSAKIS, Dimitris. Deception detection in text and its relation to the cultural dimension of individualism/collectivism. Natural Language Engineering, [S. l.], p. 1–62, 2021. DOI: 10.1017/S1351324921000152.

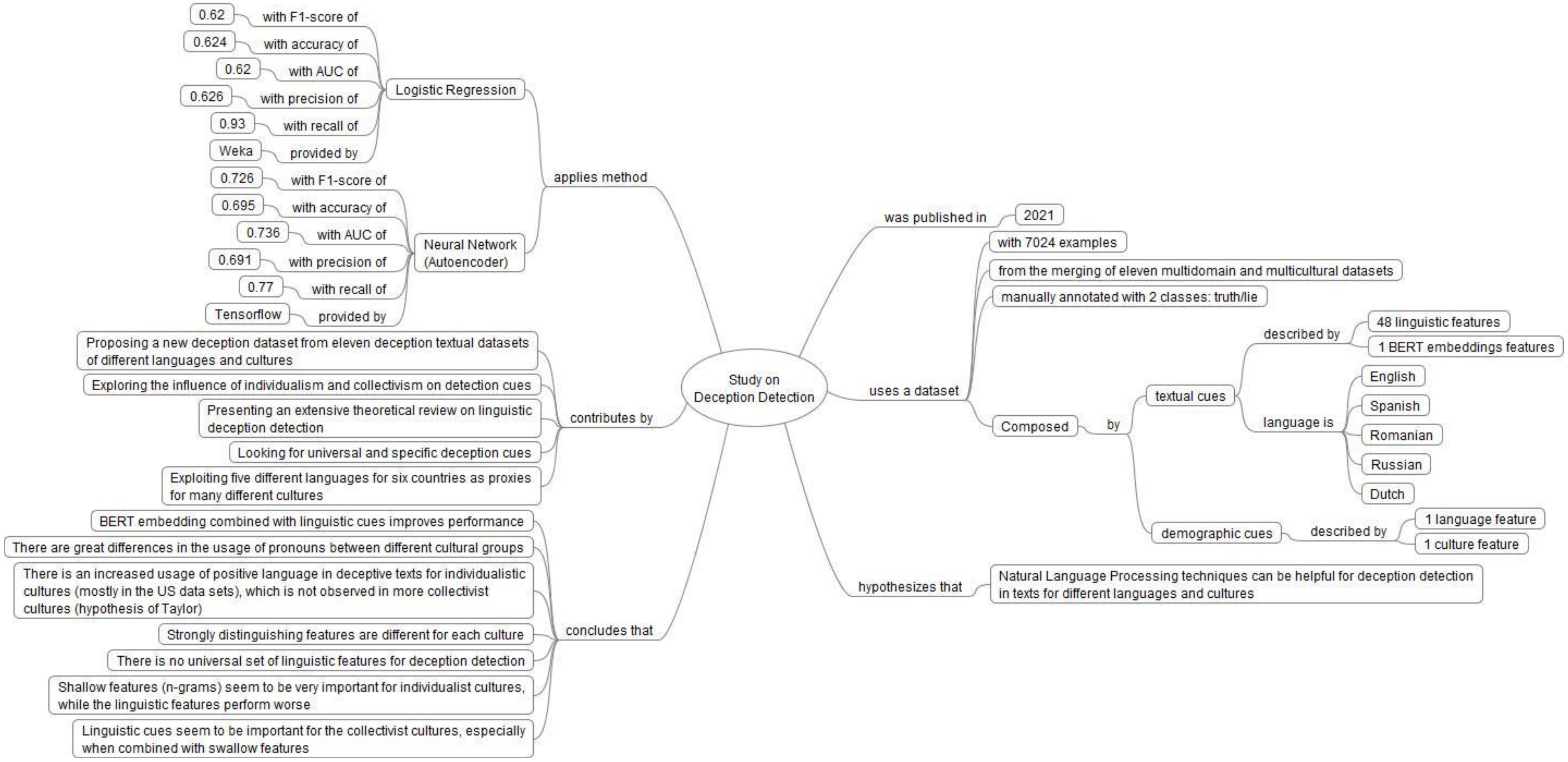

79. Detecting Lies is a Child (Robot)'s Play: Gaze-Based Lie Detection in HRI

PASQUALI, Dario; GONZALEZ-BILLANDON, Jonas; AROYO, Alexander Mois; SANDINI, Giulio; SCIUTTI, Alessandra; REA, Francesco. Detecting Lies is a Child (Robot)'s Play: Gaze-Based Lie Detection in HRI. International Journal of Social Robotics, [S. l.], 2021. DOI: 10.1007/s12369-021-00822-5. Available in: https://doi.org/10.1007/s12369-021-00822-5.

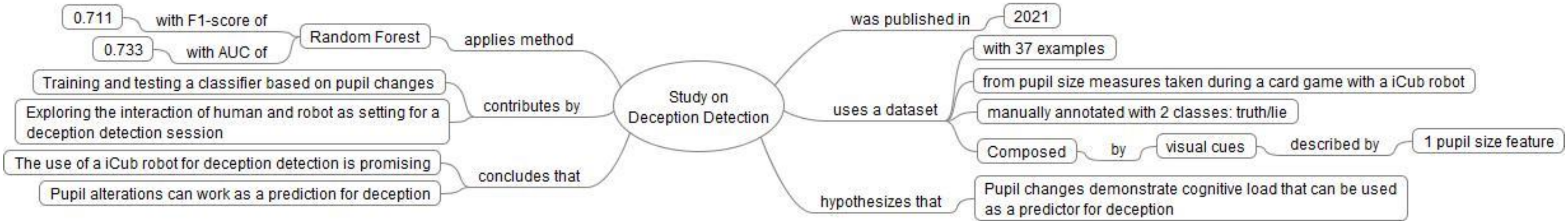

80. Deception detection and remote physiological monitoring: A dataset and baseline experimental results

SPETH, Jeremy; VANCE, Nathan; CZAJKA, Adam; BOWYER, Kevin W.; WRIGHT, Diane; FLYNN, Patrick. Deception detection and remote physiological monitoring: A dataset and baseline experimental results. 2021 IEEE International Joint Conference on Biometrics, IJCB 2021, [S. l.], 2021. DOI: 10.1109/IJCB52358.2021.9484409.

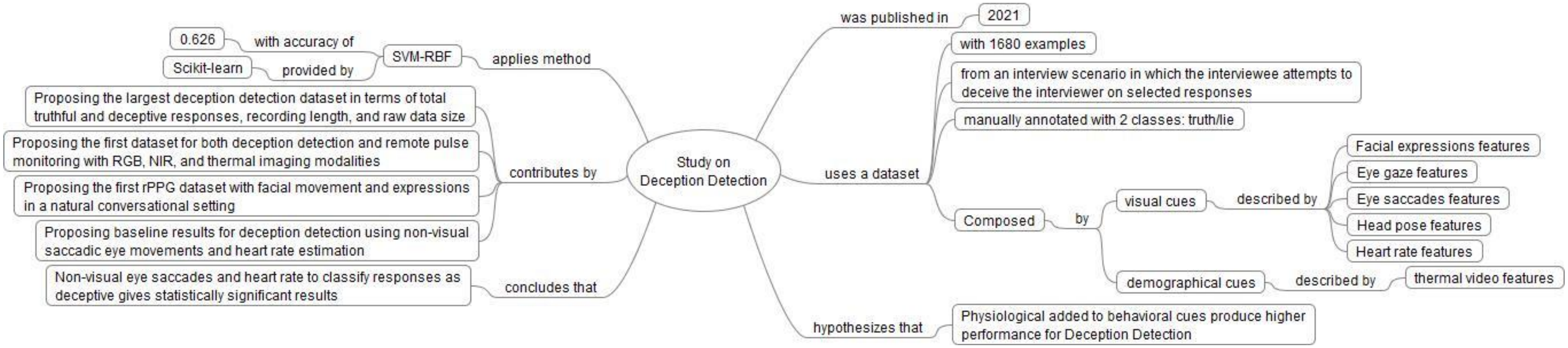

81. Automatic Detection of Deceptive and Truthful Paralinguistic Information in Speech using Two-Level Machine Learning Model

VELICHKO, A. N.; KARPOV, A. A. Automatic Detection of Deceptive and Truthful Paralinguistic Information in Speech using Two-Level Machine Learning Model. Komp’juternaja Lingvistika i Intellektual’nye Tehnologii, [S. l.], v. 2021-June, n. 20, p. 698–704, 2021. DOI: 10.28995/20757182202120698704.

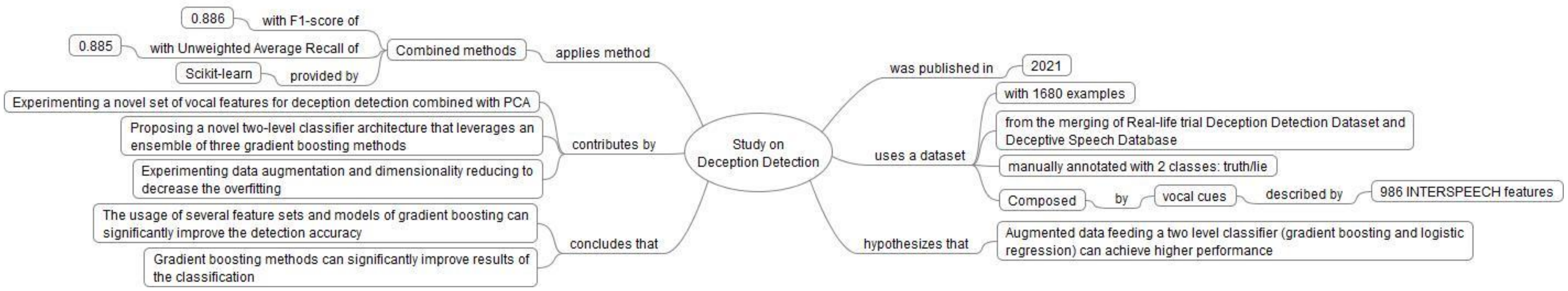
Study on Deception Detection
applies method
Combined methods
with F1-score of
0.886
with Unweighted Average Recall of
0.885
provided by
Scikit-learn
contributes by
Experimenting a novel set of vocal features for deception detection combined with PCA
Proposing a novel two-level classifier architecture that leverages an ensemble of three gradient boosting methods
Experimenting data augmentation and dimensionality reducing to decrease the overfitting
concludes that
The usage of several feature sets and models of gradient boosting can significantly improve the detection accuracy
Gradient boosting methods can significantly improve results of the classification
was published in
2021
uses a dataset
with 1680 examples
from the merging of Real-life trial Deception Detection Dataset and Deceptive Speech Database
manually annotated with 2 classes: truth/lie
Composed
by
vocal cues
described by
986 INTERSPEECH features
hypothesizes that
Augmented data feeding a two level classifier (gradient boosting and logistic regression) can achieve higher performance
